# Pharmacologically induced weight loss is associated with distinct gut microbiome changes in obese rats

**DOI:** 10.1186/s12866-022-02494-1

**Published:** 2022-04-07

**Authors:** Silvia Raineri, Julia A. Sherriff, Kevin S. J. Thompson, Huw Jones, Paul T. Pfluger, Nicholas E. Ilott, Jane Mellor

**Affiliations:** 1 Department of Biochemistry, University of Oxford, Oxford, OX1 3QU UK; 2 Chronos Therapeutics Ltd., Magdalen Centre, The Oxford Science Park, Oxford, OX4 4GA UK; 3 Research Unit Neurobiology of Diabetes, Helmholtz Zentrum München, Ingolstädter Landstrasse, 1D-85764 Neuherberg, Germany; 4 Oxford Centre for Microbiome Studies, Kennedy Institute of Rheumatology, Roosevelt Drive, Oxford, OX2 7FY UK

**Keywords:** Shotgun metagenomics, Faecal microbiome, Obese female Wistar rats, Obesity, Weight loss, Weight loss drugs, Sibutramine, Bupropion, Naltrexone, Tacrolimus/FK506

## Abstract

**Background:**

Obesity, metabolic disease and some psychiatric conditions are associated with changes to relative abundance of bacterial species and specific genes in the faecal microbiome. Little is known about the impact of pharmacologically induced weight loss on distinct microbiome species and their respective gene programs in obese individuals.

**Methodology:**

Using shotgun metagenomics, the composition of the microbiome was obtained for two cohorts of obese female Wistar rats (*n* = 10–12, total of 82) maintained on a high fat diet before and after a 42-day treatment with a panel of four investigatory or approved anti-obesity drugs (tacrolimus/FK506, bupropion, naltrexone and sibutramine), alone or in combination.

**Results:**

Only sibutramine treatment induced consistent weight loss and improved glycaemic control in the obese rats. Weight loss was associated with reduced food intake and changes to the faecal microbiome in multiple microbial taxa, genes, and pathways. These include increased β-diversity, increased relative abundance of multiple *Bacteroides* species, increased Bacteroides/Firmicutes ratio and changes to abundance of genes and species associated with obesity-induced inflammation, particularly those encoding components of the flagellum and its assembly.

**Conclusions:**

Sibutramine-induced weight loss in obese rats is associated with improved metabolic health, and changes to the faecal microbiome consistent with a reduction in obesity-induced bacterially-driven inflammation.

**Supplementary Information:**

The online version contains supplementary material available at 10.1186/s12866-022-02494-1.

## Background

The gut microbiome and human metabolism are tightly connected, as it is estimated that up to 10% of blood-circulating metabolites are products of reactions carried out by the microbiome [1]. Alterations to the microbiome have been linked to a wide variety of diseases, including obesity, inflammatory bowel disease and psychiatric conditions [2–4], but it is not clear whether disruption of the host microbiome is simply associated with a disease or is playing a causative role. In obesity, a pandemic affecting over 600 million adults worldwide [5–9], also associated with insulin resistance, type 2 diabetes and high blood glucose levels [8], one focus is on finding common patterns of microbiota alterations. The development of sequencing technologies such as 16S rRNA amplicon sequencing and shotgun metagenomics have allowed for descriptive analyses of the differences between the microbiota of obese and lean individuals, although progress is limited by high individual variability, and a lack of guidelines for study design and bioinformatics analysis. Nevertheless, a few common patterns have emerged. Obese patients show a less diverse microbiome, as both their α diversity, the number of different microbial species found within a subject, and their β diversity, the degree of species diversity between individuals, is different compared to healthy controls [3]. Obesity is associated with higher ratio of Firmicutes to Bacteroidetes phyla, although some studies reported little or no change [10–13]. Several lines of evidence suggest that in obese individuals, microbes belonging to Firmicutes phylum are generally enriched compared to Bacteroidetes, thus giving a lower ratio of Bacteroidetes to Firmicutes [10, 14, 15]. Microbes from the Firmicutes phylum are more efficient at digesting dietary polysaccharides, promoting the increased storage of triglycerides within adipocytes, leading to obesity [14, 15]. Furthermore, an increased presence of members of the Proteobacteria phylum, such as *Desulfovibrionaceae* and *Enterobacteriaceae*, may contribute to the obesity-induced inflammation state in patients [16, 17]. Finally, faecal microbiota transplantation (FMT) studies provide evidence of a link between obesity and an altered microbiome [18].

Many studies have focused on describing the changes in the microbiome of obese patients after surgical procedures, such as bariatric surgery or Roux-en-Y gastric bypass, but less is known about the consequences of pharmacological treatment (Additional Table 1) [11, 17, 19]. One therapeutic option is based on the combined treatment with bupropion and naltrexone, a dopamine uptake inhibitor and opioid receptor antagonist, respectively, both acting through suppression of appetite [6, 20]. This has been approved by the FDA and is currently used in the clinic as a treatment for obesity [8]. There is also anecdotal evidence for other compounds such as Tacrolimus, formally known as and from here onwards referred to as FK506, in inducing weight loss [21, 22]. High doses of FK506 act as an immunosuppressant, but its repeated administration at lower doses induces weight loss in rats (Additional Fig. 1), and thus FK506 has the potential to be re-purposed as a weight loss drug [21, 23]. Sibutramine is an oral anorexiant and while still available in some countries, in many others it has been withdrawn from the market following its association with adverse effects [20]. Many of these drugs are also mechanistically associated with immunosuppression, amelioration of alcohol, opiate or nicotine dependence, and treatment of major depressive disorders (Additional Table 1).

In the present study, the effect of a 42-day treatment with the panel of weight-loss associated drugs on weight, food intake, glucose tolerance and the microbiome of obese female Wistar rats was assessed, so that any changes to the microbiome could be related to physiology. Choosing shotgun metagenomics, a technique that allows the sequencing of all the genes in the faecal sample, as opposed to amplifying only the 16 s rRNAs, allowed the identification of not only the species composition for each sample, but the individual genes as well, adding information regarding the functional potential across rats and between different treatment conditions. A large metagenomics dataset is presented that will inform direct and indirect effects of these treatments on the composition of the faecal microbiome and the functional role of each species.

## Methods

### Animals and in vivo experimental design

One hundred and six female Wistar rats (weight range 200–250 g) were ordered from Charles River, Margate, Kent to arrive in the Bio Support Unit of the University of Nottingham Medical School in a study managed by RenaSci Ltd. (Nottingham, United Kingdom), on behalf of Chronos Therapeutics Ltd. Female animals were chosen over males, as they are a well-established model for obesity [23], and not as prone as males to behavioural issues when housed together. Female rats can be used to assess the ability of drugs to reduce body weight, as opposed to reduce weight gain as in males (See Additional Report 1). Dietary-induced obese female Wistar rates display hyperphagia, impaired glucose tolerance and significantly elevated basal leptin and insulin levels compared to lean animals, and have been shown to have excellent predictive validity for weight loss in the clinic [23]. Animals were inspected every day and removed from the study if necessary. Methods and protocols were designed by RenaSci Ltd., specifically for metabolic studies on rodents. Details of the study protocol, animal handling procedures and results are given in Additional Report 1.

**Fig. 1:**
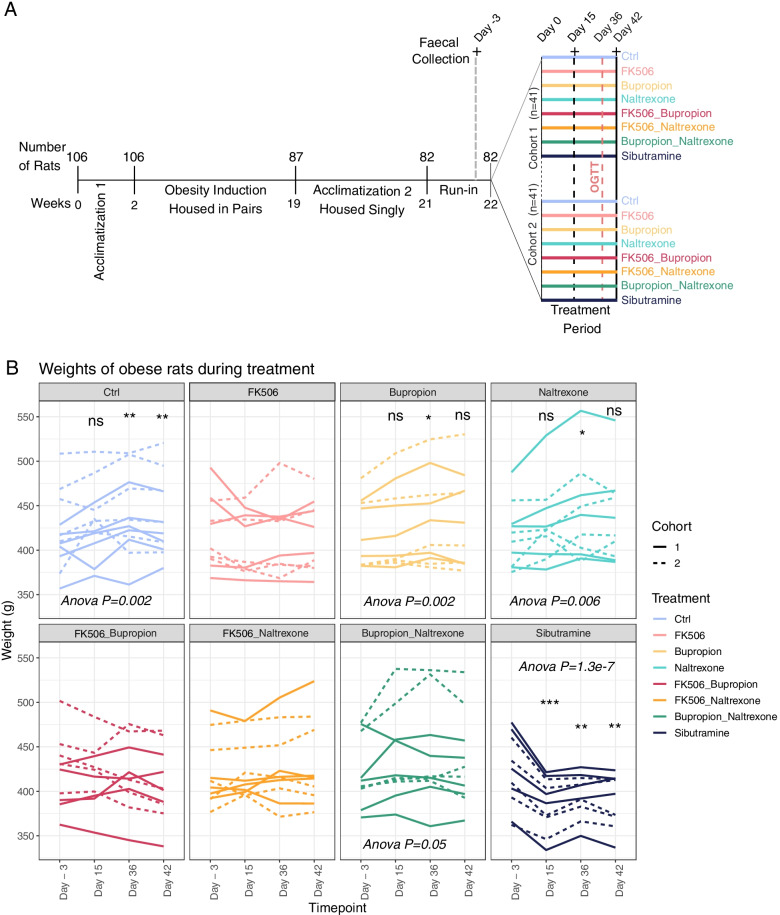
Experimental design and weights of rats treated with anti-obesity drugs. **A** Schematic outlining the experimental design. **B** Line plot showing the weight (g) of rats at day − 3, 15, 36 and 42 treated as indicated above each panel. Repeated measures ANOVA and paired T-tests reveal statistical significance for weight gain or loss over time: ns *P* > 0.05; * *P* < 0.05; ** *P* < 0.01; *** *P* < 0.00 (Control (Ctrl): *N* = 12; All other treatments: *N* = 10 rats per group)

In order to induce obesity and mimic a Western “cafeteria diet”, animals had free access to powdered high fat diet (VRF1 plus 20% lard; Special Diet Service, UK), ground chocolate, ground peanuts and tap water at all times. One hundred six rats were housed in pairs for 17 weeks for acclimatization and induction of obesity (Fig. 1A). Based on the total amount of weight gained during the obesity induction phase, 87 animals were then selected for further experiments and housed singly in polypropylene cages with wire grid floors to enable the food intake of each rat to be recorded and faecal samples to be obtained. Before starting the drug treatment programme, the 87 rats underwent a two-week acclimatization period, followed by a 7-day baseline run-in period. During the 7-day baseline run-in period, rats were weighed (to the nearest 0.1 g using a top-pan balance) and dosed once a day orally (per os, po) and intra-peritoneally (ip) with vehicle starting at 08.30 h. Based on body weights on Day − 3 and average food and water intake during the first 4 days of the baseline period, 82 of the 87 animals were allocated into 8 treatment groups, further separated in two distinct cohorts of 41 animals each (total *n* = 12 for the control group and *n* = 10 for 7 drug-treated groups, Table 1). The separation of the animals into the two cohorts was done to enable best practice in animal handling. The 5 spare rats continued to be dosed with vehicle in case they were required before the start of drug treatment.

**Table 1:** Treatment groups. Dosages and routes for drug administration of sibutramine and FK506 were based upon previous studies (Additional Fig. 1), while bupropion and naltrexone dosages were adapted from the literature [24, 25]. Each animal received drug and/or vehicle in a total volume of 4 ml/kg po (treatment 1) and 2 ml/kg ip (treatment 2)

Group	Treatment 1	Treatment 2	Ref	n
A	vehicle (castor oil/dehydrated alcohol po, 4 ml/kg po)	vehicle (saline, 2 ml/kg ip) (Fig. S1)	Fig S1	12
B	FK506 (0.6 mg/kg po)	vehicle (saline, 2 ml/kg ip) (Fig. S1)	Fig S1	10
C	vehicle (castor oil/dehydrated alcohol, 4 ml/kg po)	bupropion (20 mg/kg ip)	[24]	10
D	vehicle (castor oil/dehydrated alcohol, 4 ml/kg po)	naltrexone (1 mg/kg ip)	[26]	10
E	FK506 (0.6 mg/kg po)	bupropion (20 mg/kg ip)	Fig S1 [24]	10
F	FK506 (0.6 mg/kg po)	naltrexone (1 mg/kg ip)	Fig S1 [26]	10
G	vehicle (castor oil/dehydrated alcohol, 4 ml/kg po)	bupropion (20 mg/kg ip) plus naltrexone (1 mg/kg ip)	[26]	10
H	sibutramine (5 mg/kg po)	vehicle (saline, 2 ml/kg ip)	Fig S1	10

### Drug treatment

The panel of weight-loss drugs used includes FK506, bupropion, naltrexone either alone or in combination, and sibutramine. Routes and doses for the respective drugs were based on published pre-clinical studies in rodents/rats and preliminary experiments (Additional Fig. 1) [24, 26].

Overall food intake (kJ/g) in response to the drug treatments was calculated as the difference in weight of the chocolate, peanuts and High Fat Diet (HFD) pots in the rats’ cages between a certain day and the day before, then multiplied by the food energy values 20.79 kJ/g (HFD), 23.44 kJ/g (chocolate) and 30.34 kJ/g (ground peanuts). The sum of these three values is the combined food intake for a particular day. Throughout the duration of the study, animals were maintained on a reverse phase light-dark cycle (lights off for 8 h from 09.30–17.30 h) during which time the room was illuminated by red light. Following blood-sampling for PK on Day 42, animals were killed to a timed schedule (by rising CO_2_ concentration to minimise any fluid loss) and a terminal blood sample taken (Additional Report 1). A brief post-mortem was then performed on the animals and any unusual observations were recorded, including an examination of the pancreas and its weight.

### Oral Glucose Tolerance Test (OGTT)

An oral glucose tolerance test [27] (OGTT) was performed on Day 37. On the day preceding the OGTT, rats were deprived of food overnight but allowed free access to water. The following day, animals were cannulated and a first blood sample was taken (Timepoint − 60). Animals were then dosed with vehicle or drug as appropriate (60 min before the glucose challenge). Four minutes before the glucose challenge, an additional blood sample was taken (Timepoint 0). Thus, baseline blood samples were taken both before vehicle/drug treatment and before the glucose challenge at 0 min. Animals were then dosed orally with D-glucose (2 g/kg) and further blood samples were taken 10, 20, 30, 45, 60 and 120 min later [27]. Between blood-sampling animals were returned to the home cage with free access to water but not food. At all time-points, blood samples (~ 90 μl) were collected, and plasma separated by centrifugation (2400 g for 5 min at 4 °C) to produce a single aliquot of plasma (~ 45 μl) which was frozen (− 80 °C) and subsequently assayed for glucose and insulin as described below. After the final tail vein bleed, the cannulae were removed and feeding jars were returned to the animals. All cages were then moved back to the holding room (reverse-phase lighting).

### Measurement of plasma glucose and insulin

Plasma glucose was determined using a clinical glucose assay reagent (Thermo Electron Infinity stable reagent TR15421, Thermofisher) in a 96-well format, performed according to the manufacturer’s instructions. Plasma samples were diluted in saline prior to analysis (10x) and all determinations were performed in duplicate. Optical density at 340 nm and 400 nm (correcting wavelength) was determined using a SpectraMax 340PC384 microplate reader (Molecular Devices, USA). Glucose concentration (mM) was calculated following the manufacturer’s instructions.

Insulin was determined using the ultrasensitive rat insulin ELISA kit (cat. No 10–1251-01, Mercodia, Sweden) according to the manufacturer’s instructions, with additional insulin standards (1.50 and 1.75 ng/ml) to extend the range of the assay. Plasma samples were diluted 10x in saline solution prior to analysis, then assayed as single replicates, whilst standards were in duplicate. A five-parameter logistic curve of optical density against concentration was used to create standard curves using statistical analysis software (SAS®), then sample concentrations were estimated from the curves. The plasma glucose and insulin values of time point 0 from the OGTT was applied to an homeostasis model assessment (HOMA) of insulin resistance (HOMA-IR) and pancreatic beta cell function (HOMA-β) [28, 29].

### Faecal collection protocol

Faecal samples from the 82 rats were collected at three timepoints during the in vivo experiment. On the mornings of Days − 3, 15 and 42 immediately prior to dosing, a fresh cage pad was placed on the tray beneath the wire grid floor of the cage. The following morning (i.e. 24 h later) approximately 15 faecal pellets were removed, placed into a sterile 50 ml tube and stored at − 80 °C until DNA extraction was performed.

### Faecal DNA extraction

Faecal DNA was extracted from randomized samples using the QIAmp PowerFecal DNA kit (Qiagen, Hilden, Germany), following the manufacturer’s instructions. DNA was quantified on a Nanodrop (Thermofisher, Massachusetts, USA), then diluted to a final concentration of 10 ng/μl. To get 200 base-pairs (bp) fragments, samples were sonicated (Bioruptor, Diagenode, Belgium) with the following settings: 70 mins, 30″ on/30″ off, Low followed by 20 mins, 30″ on/30″ off, Medium. DNA was purified using a QIAQuick PCR Clean up and Purification kit (Qiagen) and quantified using Qubit (Thermofisher).

### Shotgun metagenomics sequencing

Library preparation was performed with 150 ng of starting material using NEBNext Fast DNA Library Prep Set for Ion Torrent (New England Biolabs, Massachusetts, USA) and following the manufacturer’s instructions. A barcode adapter (Ion Xpress Barcode Adapters 1–16, Thermofisher) was ligated to each library to allow for multiplexing (Table 2). DNA fragments of around 250–300 bp were size-selected on an E-Gel™ SizeSelect™ II Agarose Gels 2% (Thermofisher) as an intermediate step of the library preparation. Final concentration of each single-end library was assessed on a High Sensitivity DNA Chip for Bioanalyser (Agilent, California, USA). Shotgun metagenomics sequencing was performed on an Ion Proton sequencer (Ion Torrent, Thermofisher). Eight to ten DNA libraries with different barcodes were pooled together at a final concentration of 250 pM. Pooled libraries were loaded on an Ion PI v3 sequencing chip using the Ion Chef System (Thermofisher). A single sequencing run was performed for each pooled library.

**Table 2:** Primers and Barcodes used for library preparation and sequencing

Primer	Sequence
Ion Torrent Adapter P1	ATCACCGACTGCCCATAGAGAGGCTGAGAC
Barcode 1	CTAAGGTAAC
Barcode 2	TAAGGAGAAC
Barcode 3	AAGAGGATTC
Barcode 4	TACCAAGATC
Barcode 5	CAGAAGGAAC
Barcode 6	CTGCAAGTTC
Barcode 7	TTCGTGATTC
Barcode 8	TTCCGATAAC
Barcode 9	TGAGCGGAAC
Barcode 10	CTGACCGAAC
Barcode 11	TCCTCGAATC
Barcode 12	TAGGTGGTTC
Barcode 13	TCTAACGGAC
Barcode 14	TTGGAGTGTC
Barcode 15	TCTAGAGGTC
Barcode 16	TCTGGATGAC
Fragment Library Adapter A	TTCCATCTCATCCCTGCGTGTCTCCGACTCAG
Fragment Library Adapter A Rev	AAGGTAGAGTAGGGACGCACAGAGGCTGAGTC

### Bioinformatics analysis

#### Preprocessing files

Sequencing fastq files were loaded on Galaxy [30] (https://usegalaxy.org) for quality control. FASTQC (Galaxy Version 0.72) [31] was used to assess sequencing quality and evaluate the presence of adapters or other contaminating sequences. Sequences were trimmed using Trimmomatic (galaxy version 0.38.0) [32], using the SLIDINGWINDOW and MINLEN features to discard sequences below 30 bp long or whose average quality across a 4-nucleotide window drops below a defined threshold of 20. Newly trimmed sequences were then re-run on FASTQC to assess final quality. Host-derived sequences were removed from files through alignment with Bowtie2 (Galaxy Version 2.3.4.1) [33] against the rat canonical genome (Rn4). Unaligned sequences were written in a separate fasta file, which has been used as input for further analyses.

#### Taxonomic and functional profiling

Reads were processed using a cgat-core based pipeline (https://github.com/microbialman/MetaSequencing) to assemble reads into contigs, annotate those contigs with taxonomic and functional information and produce counts tables for each category of annotation [34]. Within this framework, reads were assembled into contigs using megahit with default parameters (v1.1.3) [35], which produced 44,789 (± 29,626) contigs of greater than 200 bp in length. Open reading frames were identified in contigs using prodigal (v2.6.3) [36] with default parameters. Open reading frames were annotated with functional information using eggnog mapper (emapper v2.0.0) [37]. The Kyoto Encyclopedia of Genes and Genomes (KEGG) was used for downstream analyses. Contigs were annotated with taxonomic information using kraken2 [38] against the kraken2-microbial-fatfree database available from https://lomanlab.github.io/mockcommunity/mc_databases.html that contains “representative” or “complete” genomes across bacteria, archaea, fungi, protozoa, virus and UniVec_Core (i.e. potential vector contaminants in genome assemblies) domains.

Reads from each sample were aligned to the corresponding assembled contigs using bowtie2 (v2.3.4.1 with default parameters) [33], and counts tables were produced using eggNOG mapper- and kraken2-derived features (KEGG orthologous groups and taxonomy) using featureCounts (v1.6.0 with default parameters, [39]). Counts across samples were combined based on feature identifiers with a sample given a count of zero if the feature was not present. The resulting tables contained 6391 unique KEGG ko features, 54 phyla, 122 classes, 254 orders, 559 families, 1621 genera and 4850 species. To functionally characterize genes, we performed gene set enrichment analysis (GSEA). This method aims at finding enrichment of entire gene sets rather than single genes, thus providing insight into the biological pathways contributing to a particular phenotype [40]. Statistical analyses have been carried out using a combination of the stats, rstatix and car R packages.

#### Microbiome composition analysis

Taxonomic profiling resulted in a table collecting microbiome composition for each sample at the species level. Diversity analyses were carried out using the R/Bioconductor package Phyloseq [41]. α diversity was calculated using Simpson’s diversity index through the estimate_richness function within the Phyloseq package, while β diversity was assessed using Bray-Curtis dissimilarity index to obtain a distance matrix (vegan, R package) [42], then visualized in a two dimensional ordination (PCA, base R). As sibutramine treatment displayed the greatest effect on weight loss, this condition was prioritized for downstream microbiome analysis. Differentially abundant genes or enriched species between sibutramine-treated rats at Day − 3 compared to Day 42 were assessed using DESeq2 [43], with the following linear model: ~ rat id + Day. Heatmaps representing differentially abundant genes or enriched species were produced with the Heatmap3 Bioconductor package [44]. Analysis of species contribution to differentially abundant genes of interest were performed using a ‘genes by species’ table obtained after taxonomic profiling, then absolute raw counts were transformed into relative counts. The top 10 contributing species were chosen for further representation, while the remainder was collapsed into the “other” category. KEGGREST Bioconductor package was used to decode KEGG-based KO gene identifiers into each gene name [45]. Statistical analysis was conducted using a combination of the stats, ggpubr and tidyverse R packages [46, 47].

### Statistical analysis of animal experiments and sequencing data

**Fig. 2:**
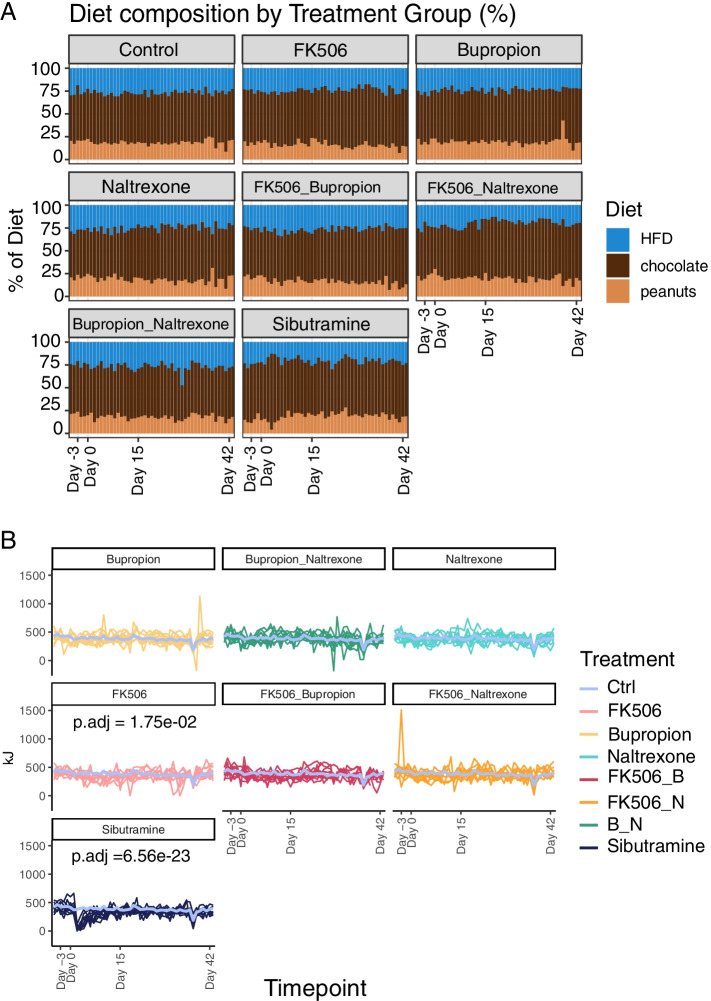
Food intake in obese rats treated with anti-obesity drugs. **A** Bar plot representing the percentage of high fat diet (HFD), chocolate or peanuts consumed by each rat throughout the experiment. **B** Combined food intake (kJ/g) for each rat throughout the duration of each treatment. A violet line represents the average food intake for rats in the control (Ctrl) group. Significant adjusted *p*-values for the day by group statistic are reported on the appropriate panels

**Fig. 3:**
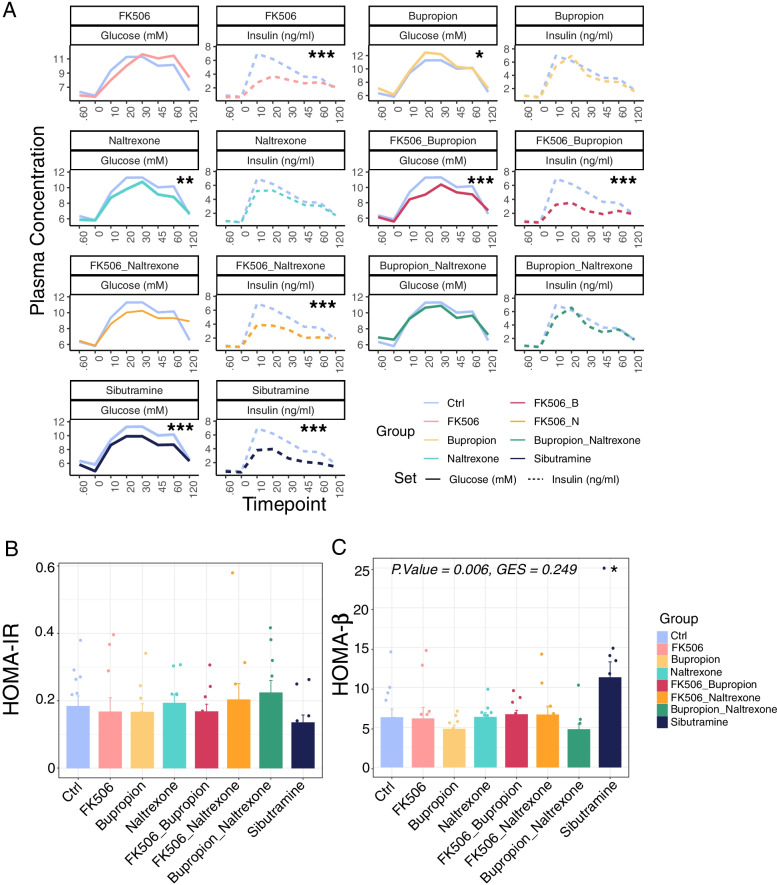
Assessment of glucose homeostasis in obese rats treated with anti-obesity drugs. **A** Line plot showing average values for plasma glucose and insulin throughout the OGTT for each treatment. A violet line represents glucose and insulin levels in the control (Ctrl). Statistical significance was assessed by ANOVA. Adjusted *p*.values: FK506, Glucose = 9.69e-04, Insulin = 1.98e-04; FK506_Buproprion, Glucose = 0.022, Insulin =4.17e-04; FK506_naltrexone, Glucose = 4.46e-04, Insulin = 7.41e-03; sibutramine, Insulin = 0.0256). Statistically significant results have been printed on the corresponding panels. Statistical significance levels = ns *P* > 0.05; * *P* < 0.05; ** *P* < 0.01; *** *P* < 0.001 (Control: *N* = 12; Treatment Groups: *N* = 10 rats per group). **B** Average HOMA-IR values for each treatment group. Fasting insulin and blood glucose measurements were taken from the 0 timepoint of the OGTT test, then HOMA-IR values were calculated according to the formula: (fasting insulin [ng/mL] × fasting blood glucose [mg/dL])/405 [28]. Data represented as mean ± SEM. Statistical significance was assessed by one-way ANOVA (*P*.value = 0.685, GES = 0.062, n.s). **C** Bar plot representing the values of HOMA-β, as function of insulin secretion and β cells activity, calculated with the formula: HOMA-β = (20× fasting insulin [μU/ml])/(fasting glucose [mmol/L] –3.5 )[29]. Data was taken from the 0 timepoint in the OGTT and is represented as mean ± SEM. Statistical significance was assessed by one-way ANOVA (Adjusted *P*.value = 0.006, GES = 0.249, **), followed by Tukey’s post-hoc test, which indicated the Ctrl vs. sibutramine comparison as statistically significant (Adjusted *P*.value = 0.0243, *). Statistical significance levels = ns *P* > 0.05; * *P* < 0.05; ** *P* < 0.01; *** *P* < 0.001 (Control: *N* = 12; Treatment Groups: *N* = 10 rats per group)

**Fig. 4:**
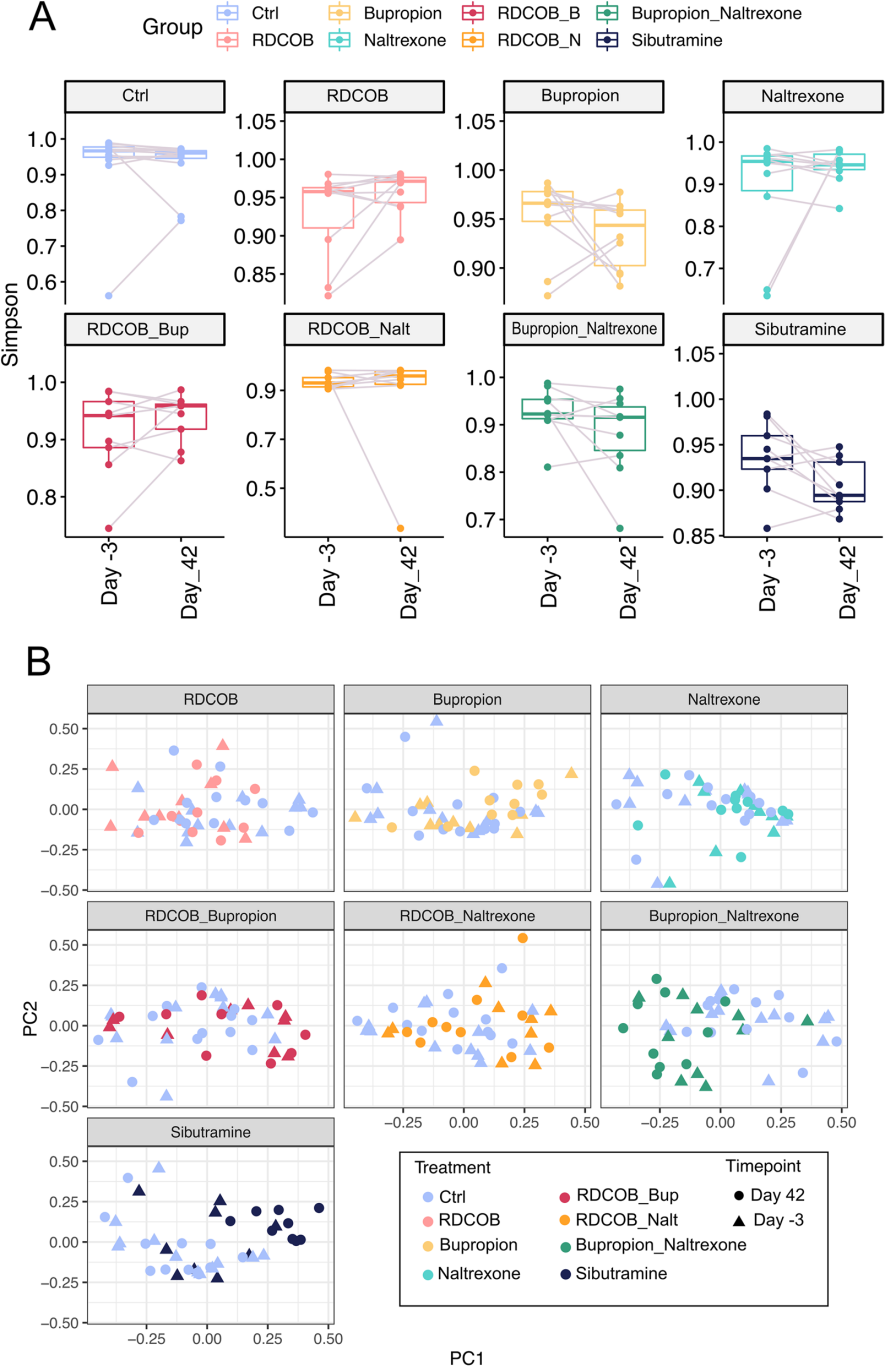
Diversity analyses on all treatment groups reveal no clear alteration of alpha diversity but some differences in beta diversity. **A** Boxplots of Simpson’s index to estimate α diversity changes, using Phyloseq’s estimate richness function, in each treatment group between the start (Day − 3) and the end of the treatment (Day 42). The higher the alpha diversity, the lower the Simpson index value. None of the comparisons were statistically significant. **B** PCA plots showing β diversity amongst samples, calculated on relative abundances by Bray-Curtis distance between each sample, using the phyloseq R/Bioconductor package [41]. The combination of bupropion and naltrexone, as well as sibutramine, were statistically significant and explained a consistent proportion of the variance (as explained by the value of *R*^2^), bupropion and naltrexone: *P*.value = 0.001, *R*^2^ = 0.13, sibutramine: *P*.value = 0.001, *R*^2^ = 0.23

**Fig. 5:**
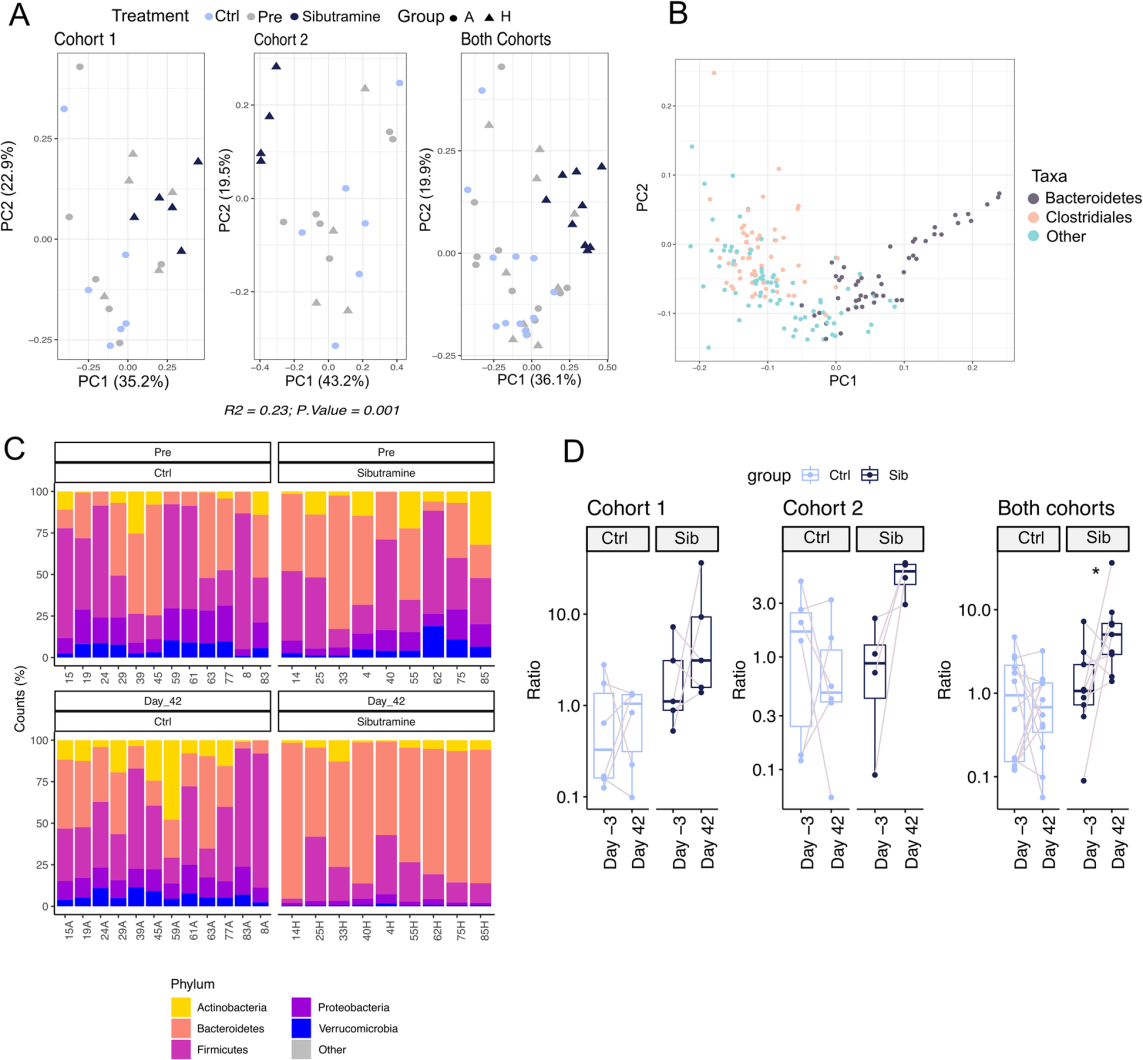
Sibutramine treatment impacts on the microbiome. **A** PCA of β diversity amongst sibutramine-treated (group H, triangles) and control (group A, circles) samples at day − 3 (pre) and day 42, calculated on relative abundances by Bray-Curtis distance between each sample, using the phyloseq R/Bioconductor package [41]. **B** PCA of β diversity amongst taxa, calculated as in A. **C** Bar plot summarizing the percent composition of the top 5 phyla across control (left panel) or sibutramine (right panel) samples, at the start (pre, top) or the end (Day 42, bottom) of the treatment. **D** Boxplots of the comparison of Ratio of Bacteroidetes/Firmicutes between Day − 3 and Day 42 of Ctrl and sibutramine (Sib)-treated group. Statistical significance was assessed by T-test. Statistical significance levels: ns *P* > 0.05; * *P* < 0.05; ** *P* < 0.01; *** *P* < 0.00 (Control: *N* = 12; sibutramine: *N* = 10 rats per group)

**Fig. 6:**
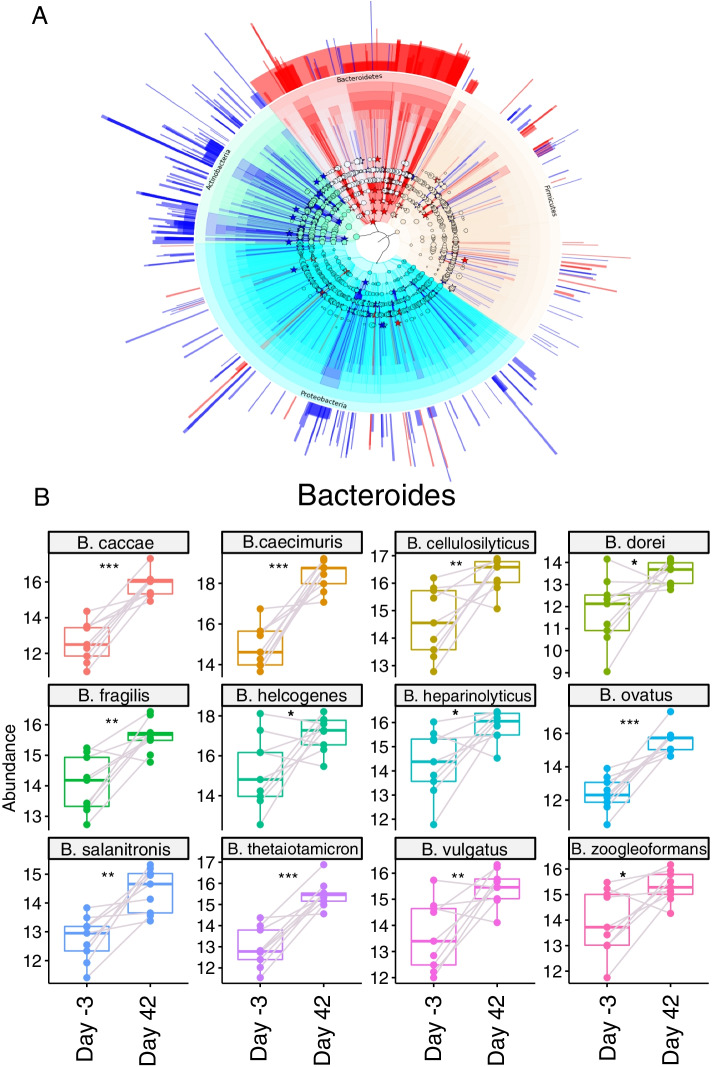
Differentially enriched species between rats before (Day − 3) and after (Day 42) sibutramine treatment. **A** Phylogenetic tree representation of differentially expressed taxa between samples at the start and end of the treatment. Differential enrichment at every classification level (Phylum, Class, Order, Family, Genus and Species) was calculated with R package DESeq2 using a linear model (~ Day + rat number). *P* values were adjusted by Benjamini-Hochberg correction and only species with P.adj < = 0.05 were considered significantly different, then tables with log2 and library size normalized counts (rlog) for every taxon were extracted [44, 49]. These were fed into GraPhlAn for phylogenetic tree visualization. External annotation corresponds to Log2 fold-change values for every differentially expressed species. Species of interest were indicated in the circular tree by stars and coloured according to their Log2 Fold change (Log2 fold-change < 0, blue; Log2 fold-change > 0, red). Concentric circles each represent a taxonomical level, starting from Phylum (Proteobacteria, light blue; Firmicutes, yellow; Bacteroidetes, red; Actinobacteria, green), then proceeding outwards to Class, Order, Family, Genus and Species. Notably, Bacteroidetes as a whole are significantly increased by the end of the treatment (see red star in the innermost circle). **B** Boxplots showing levels of differentially enriched Bacteroides species, before and after sibutramine treatment. Grey lines connect dots corresponding to the same rat. Counts used were log2 transformed and normalized by library size [49]

**Fig. 7:**
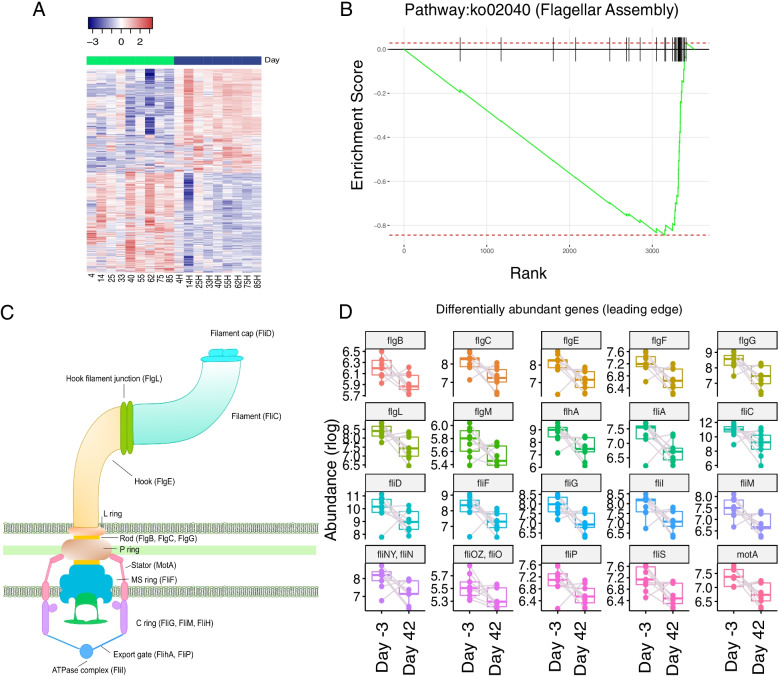
Gene Set Enrichment Analysis highlights decrease of genes related to flagellar assembly. **A** Differential gene abundance between sibutramine-treated rats at the start (day − 3) and end (day 42) of the treatment. Heatmap representing log2-transformed and normalized read counts for each of the 1047 differentially abundant genes. As with th species in Fig. 6, only genes with adjusted *P* value <= 0. 05 were considered statistically significant. Top bar shows which columns correspond to Day − 3 (green) or Day 42 (blue) samples. **B** Enrichment plot for the pathway “Flagellar Assembly” (ko:02040). Briefly, genes have been ranked by log2 fold change multiplied by the -log10(p.adjusted), then gsea was performed with the fgsea R/Bioconductor package [50]. “Flagellar Assembly” was the only statistically significant result (normalized Enrichment Score: − 1.7, p.adjusted = 0.03). **C** Schematic representation of the bacterial flagellum and its protein components. Genes detected in our analysis are indicated [51]. **D** Boxplots of leading edge genes belonging to the bacterial flagellum pathway, all of which are differentially abundant in sibutramine samples at Day 42 compared to Day − 3. Boxplots were obtained by plotting the log2-normalized expression values for each gene

Statistical tests on the raw data were performed using the Bioconductor stats R packages [48]. The code for each statistical test in included in brackets. The following tests were used: A repeated measures ANOVA (function anova_test; model: weight ~ Timepoint + Rat number + Cohort) and significance determined using a paired Student T-test comparing day − 3 with day 15, 36 and day 42 (Fig. 1B); A repeated measure ANOVA (food intake value ~ Day + Treatment Group + Day*Treatment Group + Sample; wid = sample, within = day, between = treatment, dv = food intake value) to assess food intake (Fig. 2B) followed by Tukey’s post-hoc test (Additional Fig. 2); A repeated measure ANOVA (value ~ Treatment Group + timepoint + timepoint * Treatment group + sample; wid = sample, within = Day, between = treatment, dv = value) for the OGTT (Fig. 3A); A one-way ANOVA for the HOMA-IR (HOMA-IR ~ group + Cohort) (Fig. 3B); A one-way ANOVA followed by a Tukey’s post-hoc test for the HOMA-β (HOMA-β ~ Group + Cohort) (Fig. 3C); A paired Student t-test was used to determine α diversity (Fig. 4A); The adonis test (PERMANOVA) (Treatment * Group + cohort) was used to determine statistical significance of β diversity (Fig. 4B); The adonis test (PERMANOVA) (Treatment * Group + cohort) was used to determine β diversity (Fig. 5A); the Student T-test was used to compare ratios of species (Fig. 5D); DESeq2 was used to determine differential enrichment of taxa (Fig. 6A); DESeq2 was used to determine differential enrichment of genes (Fig. 7A); A repeated measure ANOVA followed by Tukey’s post-hoc test (Additional Fig. 1); Tukey post-hoc test (Additional Fig. 2); A one-way AVOVA test and a Student T-test (Additional Fig. 3A,B); DESeq2 (Additional Fig. 5); A one-way ANOVA and Tukey’s post-hoc on pancreatic insulin levels (Additional Table 2). For all tests, *p*-values were adjusted using the Benjamini-Hochberg method.

## Results

### Sibutramine induces significant weight loss in obese female rats maintained on an HFD

Weight was monitored in female obese Wistar rats undergoing drug treatment (Fig. 1B, Table 3). The data from the two cohorts was pooled. When comparing average weight at day − 3 and day 42, the control (Ctrl) and naltrexone-treated rats showed a significantly higher final weight: Benjamini-Hochberg corrected *p*.value, padj = 0.005; T-statistic = − 4.31 (Ctrl), and padj = 0.04; T-statistic = − 2.9 (naltrexone). By contrast, no significant weight change was observed for FK506 (padj = 0.52), bupropion (padj = 0.06), FK506 and bupropion (padj = 0.14), FK506 and naltrexone (padj = 0.17) or bupropion and naltrexone (padj = 0.22). The sibutramine-treated rats showed a significantly lower final weight (padj = 0.005; T-statistic = 4.06) and had the smallest standard deviation when comparing weight at the start and end of the treatment period (Table 3). This was mainly due to a significant decrease in body weight within the first 15 days, that was maintained throughout the duration of the study (ANOVA *P*.value = 1.3e-7) (Fig. 1B).

**Table 3:** Average weight before (Day − 3) or at the end of the experiment (Day 42) for each treatment group. The standard deviations are indicated for each value

Treatment Group	Average Weight (Day − 3)	Standard Deviation (Day − 3)	Average Weight (Day 42)	Standard Deviation (Day 42)
Ctrl	420.30	41.6	435.69	42.7
FK506	420.6	39.9	416.27	38.9
bupropion	417.45	37.8	431.56	52.6
naltrexone	416.38	35.8	436.76	49.3
FK506 & bupropion	421.61	39.8	408.54	40.7
FK506 & naltrexone	420.05	38.0	428.79	47.6
bupropion & naltrexone	421.03	38.9	433.39	50.9
sibutramine	420.12	40.8	391.83	29.3

As treatment with sibutramine, a known oral anorexiant and appetite suppressant, reduces weight in obese rats, the preference and overall consumption of food in response to the various drug treatments was investigated.

### Food intake, but not the composition of the diet, is influenced by treatment with weight-loss associated drugs

Rats were granted ad libitum access to three different pots containing chocolate, peanuts or powdered High Fat Diet (HFD). All groups showed a comparable preference for chocolate, making up to 40–50% of their daily intake, followed by HFD and peanuts (Fig. 2A). Food consumption (kJ/g) was monitored daily (Fig. 2B). As expected, food consumption dropped for all treatment groups when the rats were fasted in preparation for the oral glucose tolerance test (OGTT) (Additional Fig. 2 and Fig. 2B). Only two treatments, FK506 and sibutramine, decreased food consumption. FK506 treatment led to a moderate but significant decrease in food consumption (P.adjusted = 2.62e-03, Fig. 2B). The decrease in overall food intake compared to vehicle was more profound for sibutramine, and mainly driven by a temporary decrease within the first week of treatment (P.adjusted = 1.05e-25, Fig. 2B, Additional Fig. 2). Both sibutramine and FK506 have a significant association of Day by Treatment, with sibutramine explaining 19% of the variance observed and FK506 explaining 7% (See Table 4, Generalized Effect Size (GES). Next, the effect of the drug treatments on general metabolic health, particularly fluctuations (excursions) in blood glucose levels after feeding and associated insulin secretion, was explored using an oral glucose tolerance test (OGTT).

**Table 4:** ANOVA results for combined food intake consumption. Statistical significance for differences in food intake was computed with ANOVA using the model: value ~ Day + Treatment + Day*Treatment + sample within the Rstatix R/Bioconductor package. Resulting *p*-values were corrected into adjusted *p*-values (p.adj) using the Benjamini-Hochberg correction. Adjusted *P*-values are shown for every comparison in the model, together with generalized effect sizes (GES), representing the proportion of variance explained

ANOVA	Day		Treatment		Day by Treatment	
	P.adj	GES	P.adj	GES	P.adj	GES
FK506	2.33E-21	0.18	3.40E-05	0.01	1.75E-02	0.07
bupropion	5.50E-20	0.18	0.21	0.002	0.88	0.036
naltrexone	7.84E-24	0.2	0.35	0.001	0.61	0.045
FK506 & bupropion	2.11E-21	0.19	0.003	0.01	0.24	0.05
FK506 & naltrexone	1.18E-16	0.16	0.48	0.0009	0.65	0.044
bupropion & naltrexone	6.63E-23	0.19	0.65	0.0002	0.65	0.04
sibutramine	1.16E-42	0.28	4.06E-27	0.11	6.56E-23	0.19

### FK506 does not improve insulin secretion or blood glucose levels in obese rats

Improved glucose tolerance in response to FK506 alone, or in combination with bupropion or naltrexone, compared to vehicle controls, are supported by the significantly (*p* < 0.001) decreased excursions of insulin in the OGTT (Fig. 3A). However, this is not matched by an equivalent decrease in glucose excursions, as for FK506, or FK506 in combination with naltrexone, these are significantly (*p* < 0.001) increased compared to the control rats. A significant (*p* < 0.05) reduction in glucose excursions is only seen with the FK506 in combination with bupropion.

To further delineate the impact of FK506 on blood glucose control, either alone or in combination with bupropion or naltrexone, the fasting plasma glucose and insulin values at time point 0 from the OGTT were applied to a homeostasis model assessment (HOMA) of insulin resistance (HOMA-IR) and pancreatic beta cell function (HOMA-β) (Fig. 3B,C). In addition, the post-mortem pancreatic weights and insulin content in rats at Day 42 were assessed (Additional Fig. 3). Both the HOMA-IR index values (Fig. 3B) and the HOMA-β values (Fig. 3C) indicate no improvement in β cell function and insulin secretion. In rats treated with FK506, either alone or in combination, pancreatic weights are unchanged but insulin production is reduced and blood glucose is increased (Additional Fig. 3). As FK506 does not improve metabolic health, next the impact of sibutramine on metabolic health was assessed.

### Sibutramine improves pancreatic β cell function and insulin sensitivity in obese rats

The results during the OGTT in sibutramine-treated rats indicate improved glucose tolerance and insulin sensitivity (Fig. 3A). This was confirmed by the trend towards increased insulin sensitivity in the HOMA-IR values (Fig. 3B) and the significantly higher HOMA-β value indicating improved β cell function and insulin secretion compared to the control (Fig. 3C). Sibutramine leads to an overall reduction in weight in the cohort and improved insulin sensitivity in the OGTT assay. Next we determined how the faecal microbiome was influenced by the treatments.

### Sibutramine and a combination of bupropion and naltrexone influence the diversity of species in the faecal microbiome

To assess whether the different drug treatments affected the microbiome, a diversity analysis was conducted on the sequences obtained. There was no statistically significant change in α diversity, a measure of the diversity of microbial species within an individual, at Day 42 vs. Day − 3 for any of the treatments investigated (Fig. 4A). Notably, the higher the α diversity, the lower the Simpson Index will be. β diversity assesses the diversity in species composition between samples and was altered by sibutramine (*R*^2^ = 0.23, *p*.value = 0.001, an indication of how much the variance is explained by the treatment), or a combination of bupropion and naltrexone (*R*^2^ = 0.13, *p*.value = 0.001), but no other drug or combination (Fig. 4B).

### A weight-loss independent influence of a combination of bupropion and naltrexone on the microbiome

Sequence analysis of the faecal microbiome in bupropion and naltrexone treated rats revealed a significant increase in members of the Bacteroidetes phylum (Additional Fig. 4A) with individual species showing a trend towards enrichment at day 42 (Additional Fig. 4B). Several pathways are significantly associated with the changes in the microbiome including ABC transporters (decreased), oxidative phosphorylation (enriched) and methane metabolism (decreased) (Additional Fig. 4C). In addition to the Bacteroidetes, a number of genes (Additional Fig. 5A) and species (Additional Fig. 5B) also showed differential enrichment after the combined bupropion and naltrexone treatment, although the significance of these changes remains to be determined. Next the impact of sibutramine on the microbiome was examined.

### Sibutramine treatment has an impact on the faecal microbiome

A principal component analysis (PCA) of β diversity from the two cohorts of sibutramine-treated rats showed control samples clustering with samples taken before the start of the treatment (Pre), while samples from rats treated with sibutramine for 42 days fell into a separate cluster (Fig. 5A). Furthermore, ordination analysis of the taxa present in the dataset highlighted a group of Bacteroidetes species separating along the first principal component (PC1), while a smaller cluster of the Clostridiales family (Firmicutes) is spread across PC2 (Fig. 5B). Next the Firmicutes to Bacteroidetes ratio was determined, as a high ratio is a marker of obesity.

### Sibutramine treatment increases the Bacteroidetes to Firmicutes ratio

Upon weight loss, in both cohorts, sibutramine treated rats had an increased Bacteroidetes to Firmicutes ratio at day 42 vs. day − 3. For the first cohort of rats, the mean ratio at day − 3 in the pre-treated was 3.04 rising to 12.23 after 42 days treatment with sibutramine, a fold increase of 4.02. The second cohort showed a similar fold increase of 5.20. When the data for cohorts 1 and 2 was combined the mean fold increase was 4.32 (Fig. 5C and D).

### Sibutramine treatment leads to changes in the abundance of multiple species

The 192 species that showed differential abundance (P.adj < = 0.05) between day − 3 and 42 days of treatment with sibutramine were classified into a phylogenetic tree (Fig. 6A). Concentric circles reveal differential enrichment at every classification level from phylum (inside) to species (outside) (Fig. 6A). Actinobacteria species detected showed a significant decrease by the end of the treatment. Moreover, a redistribution of several probiotic species is evident. Five members of Bifidobacteriaceae, such as *B.pseudolongum, B.animalis, B.catenulatum, B.dentium* and *B.scardovii* were significantly decreased, while two Lactobacillales, *L.reuteri and L ruminis*, increased by the end of the treatment. At the phylum level, Firmicutes did not show overall significant changes, as some of their species increase, such as *Flavonifractor plautii*, while others were decrease in abundance. Bacteroidetes were significantly increased by Day 42, with 13 species significantly increased by sibutramine treatment; *B.thetaiotamicron, B.caecimuris, B.heparinolyticus, B. cellulosilyticus, B. fragilis, B.caccae, B.vulgatus, B.salanitronis, B.ovatus, B.helcogenes, B.zoogleoformans, B.dorei* (Fig. 6B).

### Functional analysis of microbiomes in sibutramine-treated obese rats reveals a significant decrease of “Flagellar assembly” related genes

Single species changes might not explain all the differences in microbiome composition before or after sibutramine treatment. Analysis of differentially abundant genes revealed statistically significant changes in 1047 genes, of which 536 were increased and 509 decreased in sibutramine-treated rats (Fig. 7A). Gene set enrichment analysis (GSEA) revealed a significant enrichment of genes from the “Flagellar Assembly” set at the lower end of the ranked list, indicating their decrease in sibutramine-treated samples (Normalized enrichment score (NES) = − 1.71, p.adjusted = 0.03, Fig. 7B). In bacteria, the flagellum is a hair-like structure used for locomotion (Fig. 7C) and has been shown to induce inflammation due to stimulation of interleukin 8 (IL8) release from intestinal epithelial cells. Further analysis of genes associated with this pathway highlighted a panel of 20 genes, all of which are differentially abundant and significantly decreased by the end of sibutramine treatment (Fig. 7D).

Genes in this panel encode for each component of the flagellum. *FliD*, *fliC*, *flgL* and *flgE* gene products make up for the extracellular portion of the flagellum, encoding the filament cap, the filament itself, the hook junction and the hook, respectively. *FlgB*, *flgC*, *flgF* and *flgG* encode for the rod, while *motA* and *fliF* are representative of the membrane-spanning basal body, encoding for a part of the rotor and the MS ring, which provides the base for flagellar assembly. Finally, five genes, *fliM*, *fliN*, *flhA* and *fliP*, encode for proteins making up the export apparatus on the cytosolic side (Fig. 7C).

**Fig. 8:**
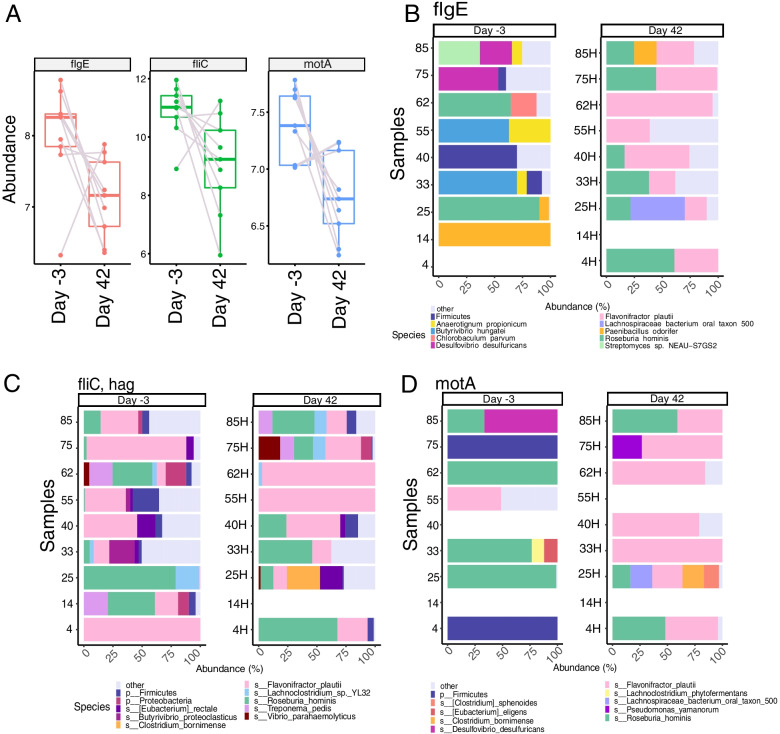
Species composition of three representative genes from the “Flagellar Assembly” pathway. **A** Boxplots showing expression levels of three representative members of the “Flagellar Assembly” pathway. Expression values have been log2 normalized as in Fig. 7. **B-D** Species contribution to the levels of *flgE* (**B***), fliC* (**C**) and *motA* (**D**) in the faecal microbiome. Gene counts for each species are expressed as a percentage and only the top 10 species for each gene are represented, whilst the remainder have been collapsed under the “other” category. Empty rows correspond to samples that either had no reads annotated to the gene (*motA*, 55H and 14H; *flgE* 4 and 14H) or whose reads were entirely annotated to “k_unassigned” (Kingdom unassigned), (*fliC*, 14H; *motA*, 14 and 40). Notably, every sample had a certain proportion of reads annotated to “k_unassigned” and thus lacked a reliable annotation. These reads have been excluded from the dataset prior to plotting of *fliC*, *flgE* and *motA*. The percentage of unassigned reads for each gene, within every sample, is described in Additional Table 3

Further analysis of the species that are likely to contribute to the enrichment of three representative genes, one for each major flagellum component, *flgE*, *fliC* and *motA* (Fig. 8A), indicated *Flavonifractor plautii* and *Roseburia hominis* as the two major sources of the respective genes (Fig. 8B-D). Interestingly, while *F.plautii* is differentially enriched at Day 42, *R.hominis* levels do not significantly change over time (*F.plautii*: Log2-fold change = 1.86, p.adjusted = 0.03; *R. hominis*: Log2 fold change = 0.93, p.adjusted = 0.49). *R.hominis* is a butyrate-producing bacterium from the Firmicutes phylum, and while its impact on *flgE* counts seems to be increasing by Day 42, in both *motA* and *fliC* it remains constant throughout the treatment (Fig. 8B). *F.plautii* is a flavonoid-metabolizing species from the Clostridiales family (Firmicutes). Its contribution to gene counts of *flgE* and *motA* increases in sibutramine-treated rats by Day 42 (Fig. 8B and D), while it is always present in the counts of *fliC* (Fig. 8C).

## Discussion

This study reveals how a range of drug treatments influence weight, food intake, metabolic health and the composition of the microbiome in obese female rats maintained on a high fat diet. Unexpectedly, we observed considerable variability in the weight gain or loss between and within the intervention groups. Reasons for this discrepancy remain elusive but may in part be based on our choice of using obese rats, their sex and/or our dietary feeding regimen. Despite their association with weight loss, most treatments led to an increase in weight during the period of study with the exception of sibutramine. For sibutramine, the weight loss in obese rats can, at least in part, be explained by a reduction in food consumption without any change in food preference. Interestingly, naltrexone and bupropion, currently used in the clinic to treat obesity and linked to suppression of appetite [24], failed to induce significant weight loss, with naltrexone-treated rats gaining weight by Day 42.

This study also highlights other physiological changes to the obese rats that appear unrelated to weight loss or changes to the microbiome composition that might warrant further investigation, for example the impact of FK506 combined with bupropion or naltrexone on insulin sensitivity and blood glucose levels. In non-obese rats, FK506 is associated with maintenance of a healthy weight compared to untreated controls [21] (see Additional Fig. 1). In obese rats, FK506 elicited at best modest effects on body weight and food intake but induced a profound decrease in insulin secretion upon a glucose challenge when given either alone or in combination with naltrexone or bupropion. This is likely to reflect reduced pancreatic beta cell function, as pancreatic weight is not changed. Overall, our data suggest that FK506 has a restricted therapeutic window as obese patients often show insulin resistance and are at high risk of developing diabetes [28, 52].

Multiple reports suggested a functional impact of faecal microbiome perturbations on the development of obesity and comorbid sequelae [18, 53]. Similarly, numerous studies have revealed that weight loss by dietary or surgical interventions was able to reverse perturbations in the faecal microbiome [17, 54, 55]. However, few studies to date have analysed the specific impact of pharmacological weight loss agents on faecal microbiome diversity, and even fewer study this in the context of obesity. Despite not reducing weight or improving blood glucose handling in the obese rats, the combined treatment with bupropion and naltrexone altered the microbiome composition. Changes include improving the abundance of members of the Bacteroidetes phylum which is notable as obese individuals often show a reduced proportion of Bacteroidetes in their faecal microbiome. However, the significance of this and other changes to the microbiome to the mode of action of these drugs remains to be determined.

Treatment of obese female rats with the appetite suppressant sibutramine leads to concomitant weight loss, improved glucose tolerance and insulin sensitivity, and changes to the microbiome. These include a trend towards increased α diversity, a distinct β diversity pattern and a shift in the Bacteroidetes to Firmicutes ratio towards a higher proportion of Bacteroidetes. The decrease in the overall relative abundance of genes related to flagellar assembly, previously associated with obesity-induced inflammation, as well as a higher contribution of anti-inflammatory species, such as *F.plautii*, suggests a shift towards a decreased inflammatory state in response to sibutramine-induced weight loss.

Sibutramine is a known inhibitor of serotonin reuptake, and more than 90% of serotonin production occurs in the gut, regulated by several microbial species [56–58]. Our analysis shows an influence of sibutramine treatment on the microbiome of obese rats, but it cannot define whether this change is causing weight loss, or whether weight loss induced by sibutramine is causing the changes in the faecal microbiome. Faecal microbiota transplantation (FMT) from sibutramine-treated rats to obese rats could answer this open question and reveal the functional role of such sibutramine-induced faecal microbiome changes on obesity. Such studies should further entail the study of gut serotonin production as a potential mediator of this effect.

The most striking alteration in the faecal microbiome in response to sibutramine treatment were linked to the decrease in the abundance of genes related to flagellar assembly. Flagella are rod-like structures composed by flagellin and used by bacteria for locomotion [59, 60]. Genes encoding flagella components are enriched in the microbiome of obese patients, as well as in patients with metabolic syndrome [61, 62]. The host toll like receptor 5 (TLR5) recognises flagellin and is able to activate an immune response. Thus, the increased presence of motile bacteria in the gut microbiota has been liked to a pro-inflammatory state in a range of diseases, including metabolic syndrome [63, 64]. A decrease of genes coding for this protein, along with other components of the flagellum might be indicative of a reduced inflammation in the gut of sibutramine treated rats. As suggested by the significant decrease of genes involved in flagellar assembly, differentially abundant genes hint at positive changes to the faecal microbiome, although results must be interpreted carefully. Indeed, while recent research in the field has highlighted microbial patterns associated with obesity at higher classification levels, such as the ratio of Bacteroidetes to Firmicutes and a lower α diversity [52], many of the species significantly changing are still largely unknown. Another complication is that, while over time metagenomic databases have expanded, there is still a consistent portion of reads that remain unassigned. For these reasons, our results remain largely descriptive, and with some contradictions, but mainly pointing toward the specific association of certain species, genes or pathways involved in flagellar assembly with weight loss induced by sibutramine treatment.

Between the beginning and the end of sibutramine treatment, a significant increase in 13 Bacteroides species, and in the Bacteroidetes phylum as a whole, is evident. In the context of obesity, Bacteroides species such as *B. thetaiotamicron,* were shown to be decreased in obese individuals where they contribute to glutamate fermentation and short chain fatty acid production [17]. Several studies linked an increased presence of Firmicutes species in obese individuals with improved efficiencies in extracting energy from nutrients and excessive storage [14, 65]. Overall, our data in sibutramine-treated rats are thus consistent with the reported, beneficial shift from Firmicutes to Bacteroidetes, including *B. thetaiotamicron.* However this is not universal, as some Firmicutes such as *F.plautii*, a flavonoid-metabolizing species from the Clostridiales family linked to anti-inflammatory activities, are enriched in sibutamine-treated rats and are found in the microbiome of lean individuals [66, 67].

Microbiome research remains a growing and controversial field, with many species remaining largely uncharacterised. Recent advances in sequencing techniques may help bypass the limitation of culturing species in the lab and thus overcome our lack in knowledge on the functional impact of distinct species. Our complete shotgun metagenomics dataset will serve as a resource and step in that direction, to ultimately support the study of functional roles of specific microbial species and genes in metabolic diseases such as obesity.

## Conclusions

Shotgun metagenomics analysis of the faecal microbiome from obese female rats treated with a panel of weight-loss drugs revealed that common pharmacological treatments for obesity, such as bupropion and naltrexone, did not induce consistent weight loss, while sibutramine successfully reduced the weight of the rats by decreasing their appetite. Analysis of the rats’ faecal microbiome highlighted a potentially beneficial impact of sibutramine treatment, as their microbiome composition by the end of the treatment is significantly different both compared to the beginning of the treatment and to control rats. Differential analysis revealed a significant increase in the abundance of species belonging to the Bacteroidetes phylum, and a concomitant shift of the Bacteroidetes to Firmicutes ratio towards the former, while in obese patients the ratio favours Firmicutes. Functional analysis showed a significant decrease in the abundance of genes coding for proteins of the flagellum, a bacterial component that has been linked to inflammation, further suggesting a beneficial impact of the treatment on the gut microbiome of obese rats.

These results lay the foundation for further investigation of the ways in which the transformed microbiome is affecting the host metabolism. To this end, a metabolomic analysis of the blood of sibutramine-treated rats would shed light on the kind of metabolites that the microbiome is producing, or sibutramine is inhibiting, leading to a better understanding of the connection between the gut microbiota and its host metabolism, in the context of obesity.

Furthermore, the entire shotgun metagenomic dataset is made publicly available as a resource for the field. As more species in the rat microbiome are identified and as the identity of more species is the microbiome become more refined, this data set will continue to produce new insights for the field.

## Supplementary Information

**Additional file 1: Additional Figure 1.** Long-term treatment of adult rats maintained on a high fat diet with different dosages of FK506 induces weight loss and promotes maintenance of a healthier weight. **Additional Figure 2.** Tukey’s post-hoc test following ANOVA statistical test on timepoint reveals early but temporary anorexigenic effects of sibutramine, and significant changes due to fasting on Day 36. **Additional Figure 3.** Pancreatic Insulin levels at Day 42. **Additional Figure 4.** Combination treatment of bupropion and naltrexone impacts on the host gut microbiome. **Additional Figure 5.** Differentially abundant genes and species upon bupropion and naltrexone combination treatment. **Additional Table 2.** Current pharmacotherapies for obesity. **Additional Table 2.** Tukey’s post-hoc test significant comparisons following ANOVA on pancreatic insulin levels at Day 42. **Additional Table 3.** Percentage of unassigned reads per sample for representative genes fliC, flgE and motA.

**Additional file 2: Additional report 1**. This contains the full details of the study showing the effects of Sibutramine and FK506 (RDC5), bupropion and naltrexone, alone and in combination, on body weight, food and water intake, glycaemic control, pancreatic insulin levels and fat pad, liver and pancreas weights in dietary-induced obese, female Wistar rats.

**Additional file 3.** eARRIVE Compliance Questionnaire.

## Data Availability

Raw fastq files are available from the European Nucleotide Archive (ENA), under the accession number PRJEB40767: https://www.ebi.ac.uk/ena/browser/text-search?query=PRJEB40767. All scripts are available from a dedicated github page at the URL: https://github.com/SilviaRaineri/Microbiome_PRJEB40767 . Processed data is uploaded on figshare and linked to the same github page.

## Abbreviations

HFD: High fat diet
OGTT: Oral glucose tolerance test
GES: General effect size
PCA: Principal component analysis
GSEA: Gene set enrichment analysis
NES: Normalised enrichment score
FMT: Faecal microbiota transplant
FDA: Food and Drug Administration
PK: Pharmacokinetics
p.adj.: Adjusted *p* value
ENA: European Nucleotide Archive
