# Supplementary material for: Pharmacologically induced weight loss is associated with distinct gut microbiome changes in obese rats
Source: BMC Microbiol. 2022 Apr 7;22:91. doi: 10.1186/s12866-022-02494-1 (PMC8988407; doi:10.1186/s12866-022-02494-1)
Supplement: Supplementary file 1 — Additional file 1: Additional Figure 1. Long-term treatment of adult rats maintained on a high fat diet with different dosages of FK506 induces weight loss and promotes maintenance of a healthier weight. Additional Figure 2. Tukey’s post-hoc test following ANOVA statistical test on timepoint reveals early but temporary anorexigenic effects of sibutramine, and significant changes due to fasting on Day 36. Additional Figure 3. Pancreatic Insulin levels at Day 42. Additional Figure 4. Combination treatment of bupropion and naltrexone impacts on the host gut microbiome. Additional Figure 5. Differentially abundant genes and species upon bupropion and naltrexone combination treatment. Additional Table 2. Current pharmacotherapies for obesity. Additional Table 2. Tukey’s post-hoc test significant comparisons following ANOVA on pancreatic insulin levels at Day 42. Additional Table 3. Percentage of unassigned reads per sample for representative genes fliC, flgE and motA. [file 12866_2022_2494_MOESM1_ESM.docx]

# Additional Figures and Tables


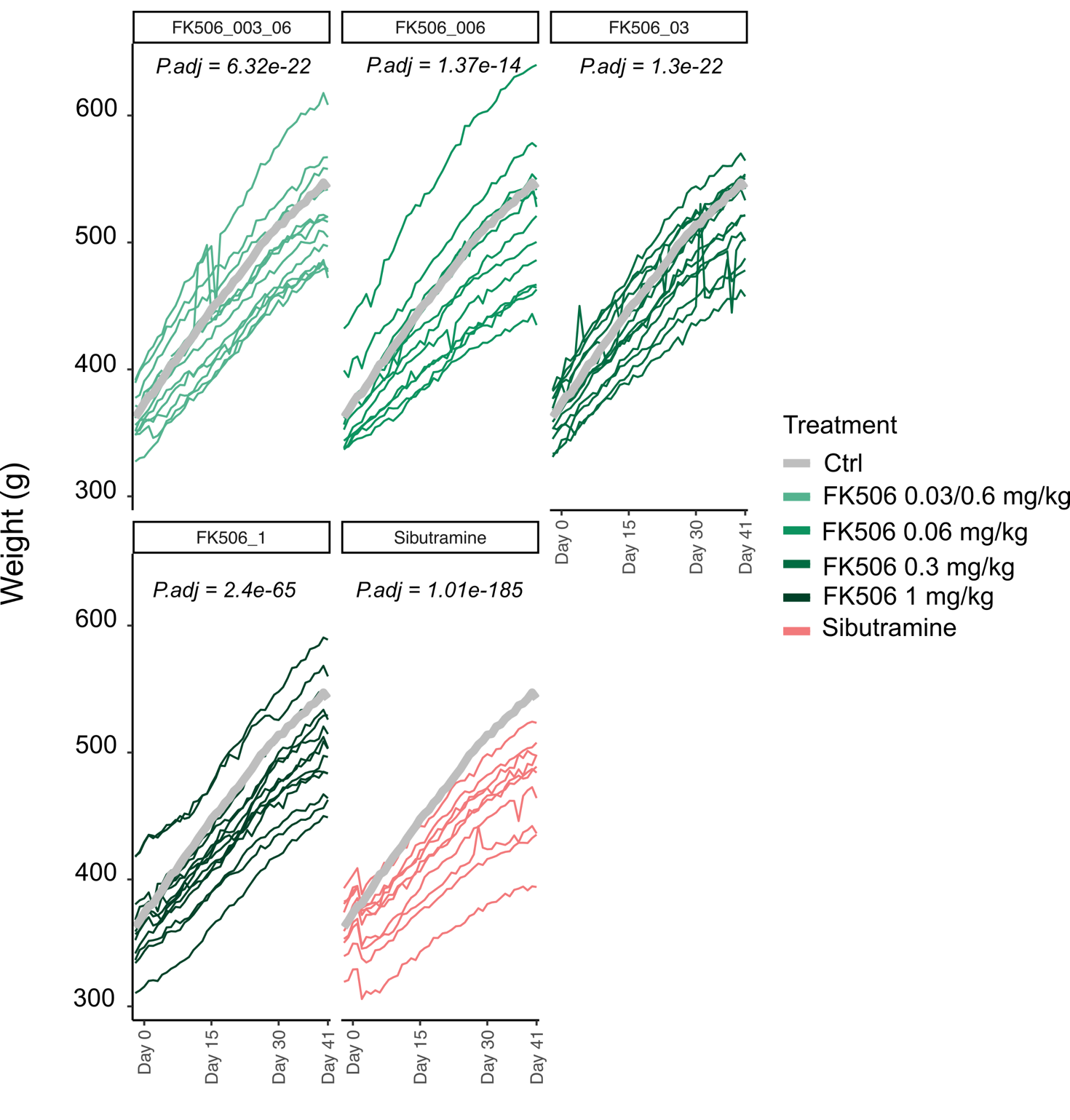


**Additional Figure 1. Long-term treatment of adult rats maintained on a high fat diet with different dosages of FK506 induces weight loss and promotes maintenance of a healthier weight.** FK506 therapeutic potential was assessed by treating 75 adult, male Sprague-Dawley rats maintained on a high fat diet for 40 days at different concentrations of drug. Rats treated with FK506 showed decreased body weight compared to untreated controls. Statistical significance was assessed by ANOVA with the model: weight ~ Day + Treatment Group + Day* Treatment Group + sample, (function anova_test within the rstatix R package, parameters: wid = sample, within = Day, between = treatment, dv = weight) [46], and significant adjusted p-values for the day by treatment group statistic have been reported on the appropriate panels. Generalized effect sizes for the Day*Treatment Group comparison were as follows: FK506 0.03/0.6 mg/kg =0.18, FK506 0.06 mg/kg = 0.1, FK506 0.3 mg/kg = 0.18, FK506 1 mg/kg = 0.34, sibutramine = 0.70.


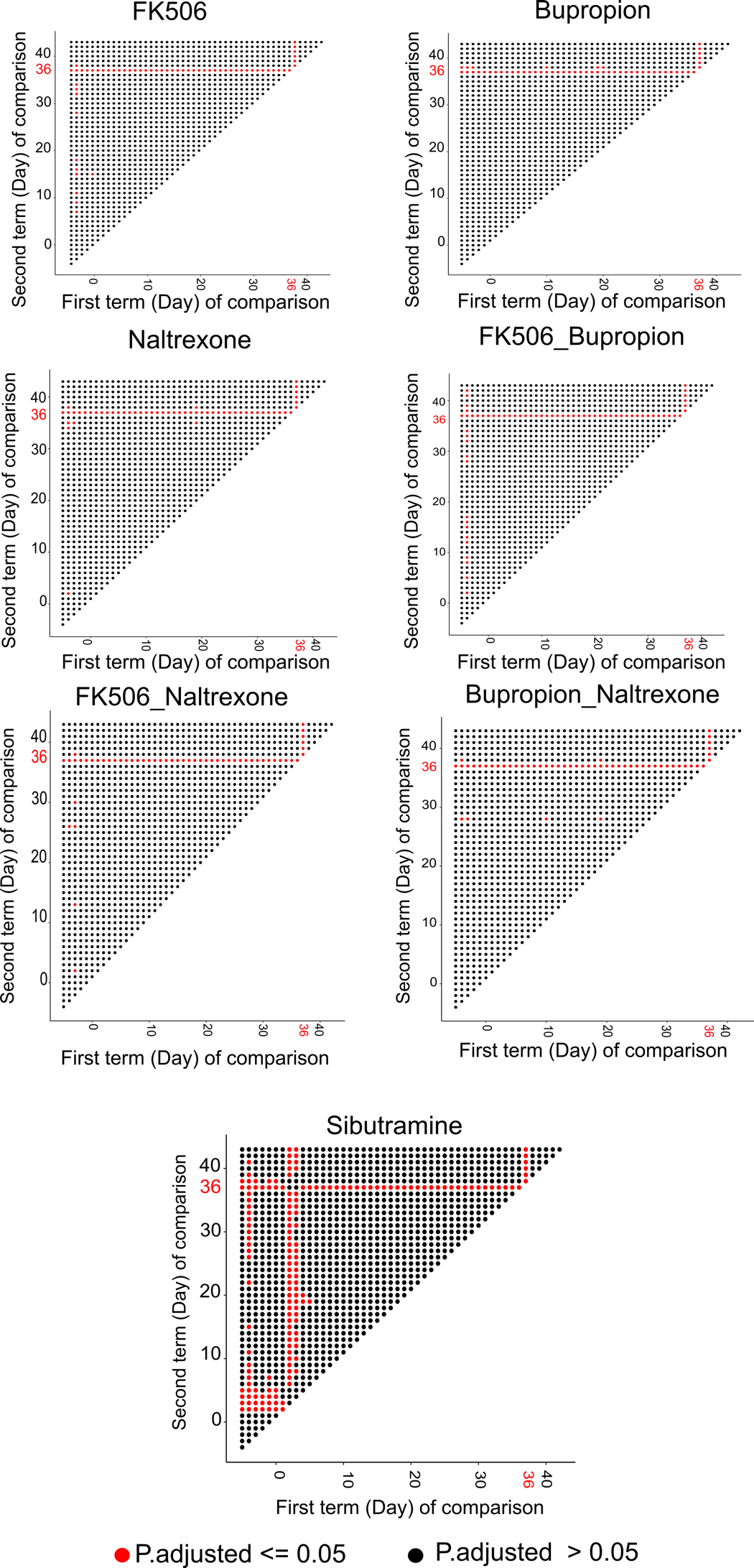


Additional Figure 2. Tukey's post-hoc test following ANOVA statistical test on timepoint reveals early but temporary anorexigenic effects of sibutramine, and significant changes due to fasting on Day 36. Visualization of the p.values (P.adj <= 0.05, red; P.adj > 0.05, black) in the Tukey’s post hoc test following ANOVA statistical test on timepoint. Food intake on a given day (first term of comparison, x axis) was compared to another (y axis) in the Tukey’s post-hoc test. The statistical test performed gives a significant p.value, indicating which days are contributing to the significance of the timepoint comparison. Closer examination of the plots for most of the treatment groups, except sibutramine, reveals that the significant term in the ANOVA test occurred on Day 36, when the rats were fasted in preparation for the OGTT test.


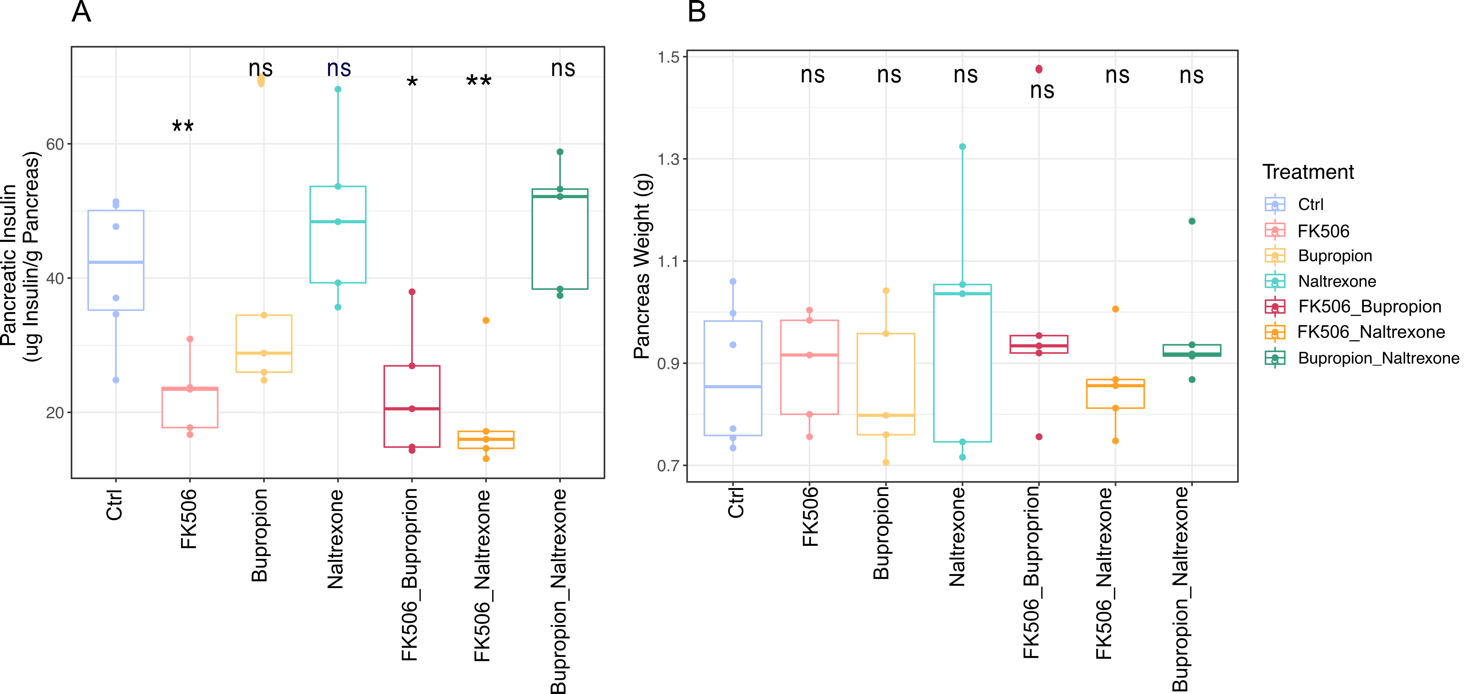


Additional Figure 3. Pancreatic Insulin levels at Day 42. A) Boxplots representing pancreatic insulin levels at Day 42. Statistical significance was assessed both by Student’s T-test of each treatment group against Controls and by ANOVA. T-test detected a significant decrease in pancreatic insulin in all FK506-treated samples, either alone or in combination (P.adj: FK506 = 0.019, FK506_bupropion = 0.03, FK506_naltrexone= 0.019). Statistical significance was confirmed by ANOVA, calculated with the formula: pancreatic insulin ~ Treatment Group + Cohort (P.adj. = 0.001 (Treatment Group), 0.99 (Cohort)), but Tukey’s post-hoc test indicated no significance when comparing any treatment group against control. Significant comparisons have been described in Additional Table 2. B) Boxplots of pancreas weight at Day 42. Statistical significance was assessed both by Student’s T-test and ANOVA (pancreas weight ~ Treatment Group + Cohort), but no statistical difference between controls and any of the treatment groups was detected.


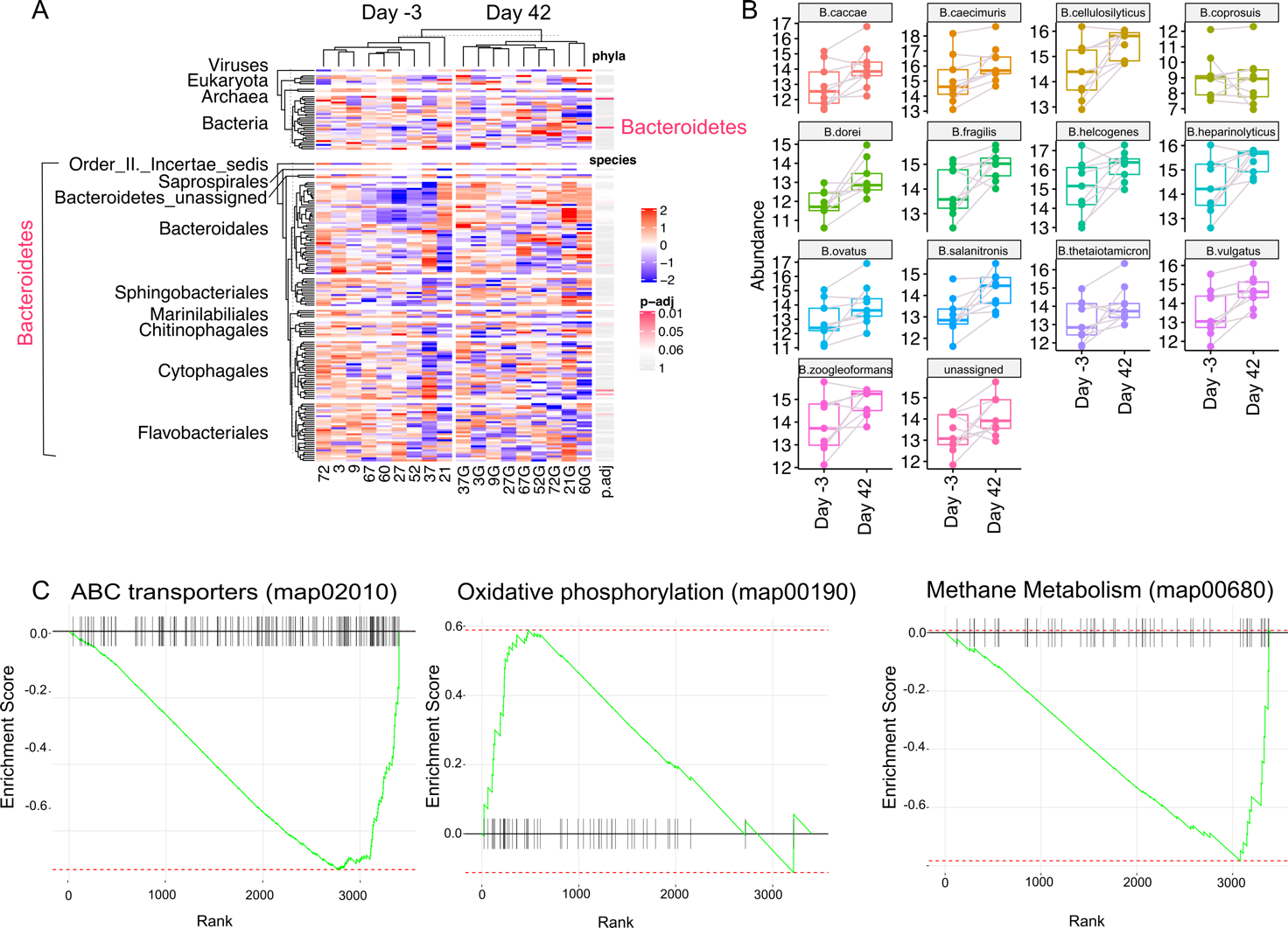


Additional Figure 4. Combination treatment of bupropion and naltrexone impacts on the host gut microbiome. A) Top: Heatmap of Phyla detected in bupropion and naltrexone-treated rats. A pink bar indicates a statistically significant change in abundance by the end of the treatment (p.adj <= 0.05). Notably, abundance of members of the Bacteroidetes phylum were significantly increased at Day 42. To further visualize this increase, all Bacteroidetes members were visualized in the bottom Heatmap, with rows split by Order. Notably, while the Phylum as a whole is significantly increased, only a handful of species are statistically significant (pink bars), but a cluster of Bacteroidales shows a trend towards increasing by the end of the treatment. This heatmap was plotted using ComplexHeatmap, using both phylum and species data [72]. B) Boxplots representing abundance levels of species belonging to the Bacteroides genus indicate a trend towards enrichment by Day 42. Grey lines connect dots corresponding to the same rat. Counts used were log2 transformed and normalized by library size [73]. C) Enrichment plots for the statistically significant pathways “ABC transporters” (map02010; NES = -1.28, p.adj = 0.02), “Oxidative phosphorylation” (map00190; NES = -1.37, p.adj = 0.05) and “Methane metabolism” (map00680; NES = 1.87, p.adj = 0.05). Briefly, genes have been ranked by log2 fold change multiplied by the -log10(p.adjusted), then gsea was performed with the fgsea R/Bioconductor package [55].

**
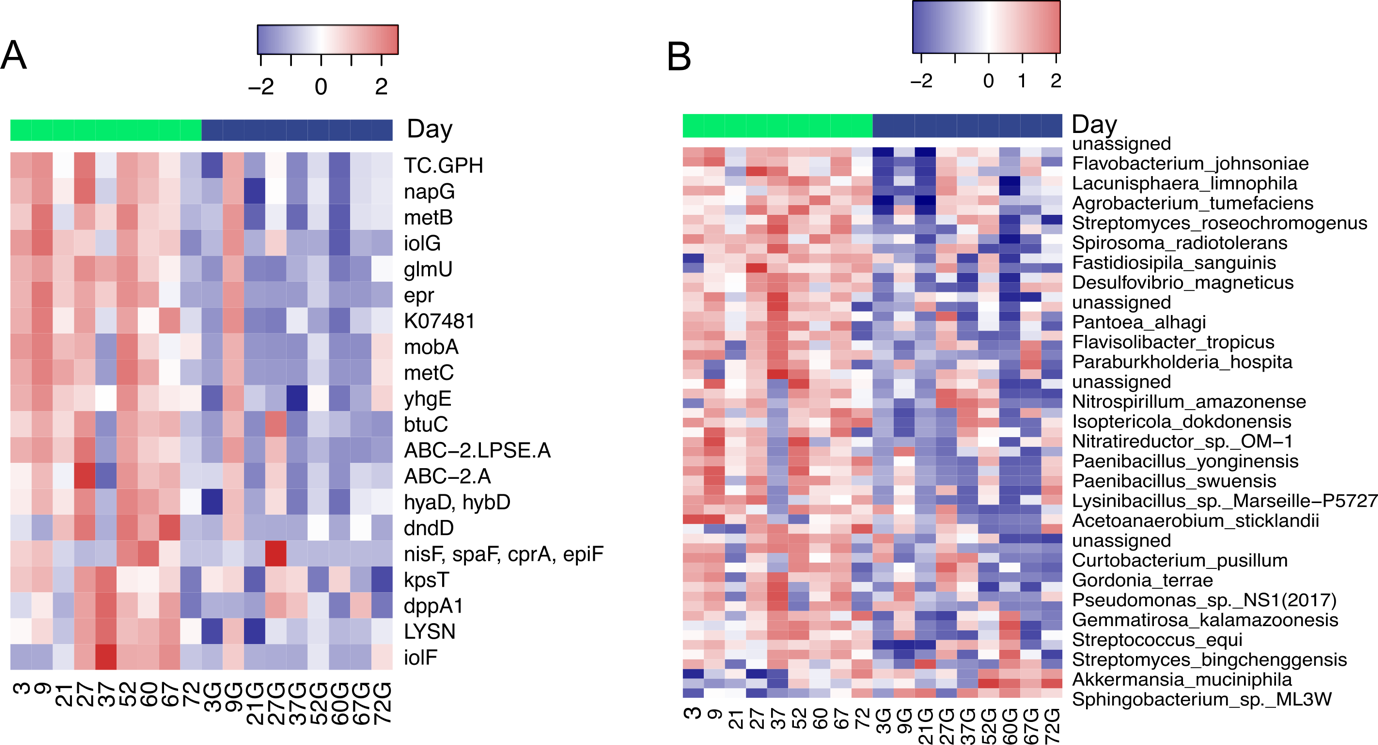
**

Additional Figure 5. Differentially abundant genes and species upon bupropion and naltrexone combination treatment. A) Differentially abundant genes between bupropion and naltrexone-treated rats at the start and end of the treatment. Heatmap represents log2-transformed and normalized read counts for each of the 20 differentially abundant genes. Significant changes in gene levels were calculated using DESeq2 with the model ~ rat number + Day [52]. P values were corrected with the Benjamini-Hochberg method, and only genes with adjusted p value <= 0. 05 were considered statistically significant. Top bar shows which columns correspond to Day -3 (green) or Day 42 (blue) samples. B) Differentially abundant species between the start and the end of the bupropion and naltrexone combined treatment. Significant changes in species abundance were calculated with DESeq2 with the model ~ rat number + Day [52]. A Heatmap was used to visualize the log2-transformed and normalized read counts for the 57 species which showed an adjusted p.value <= 0.05 and were considered statistically significant. Heatmaps were plotted using the Heatmap3 R/Bioconductor package [41].

Additional Table 1. Current pharmacotherapies for obesity. Summary of the most common pharmacological therapies for the treatment of obesity. With the exception of sibutramine, which has been withdrawn from the market due to its cardiological and, to a lesser extent, psychiatric adverse effects, all drugs are currently being used in the clinic, often together with a lifestyle change promoting diet and exercise [6,20].

| **Drug** | **Physiological mechanism** | **Chemical features** | **Administration** | **Adverse effects** |
| --- | --- | --- | --- | --- |
| Phentermine | Suppression of appetite | Sympathomimetic Amine | 15 or 37.5 mg orally once daily; 8 mg orally twice daily. Often used in combination with Topiramate. ER formulation available | Insomnia, dry mouth, constipation, dizziness |
| Orlistat | Decreased absorption of fat | Pancreatic and gastric lipase inhibitor | 120 mg three times daily | Flatulence, bloating, diarrhea |
| Lorcaserine | Modulation of food intake and appetite | Serotonin receptor agonist (5-HT2c) | 10 mg or 20 mg (ER formulation) orally once daily | Headache, dizziness, dry mouth, constipation |
| Liraglutide | Modulation of glucose homeostasis, food intake and satiety | GLP1 receptor agonist with incretin properties | Start with 0.6 mg subcutaneously once daily for a week, then weekly titration up to 1.2 mg, 2.4 mg, 3 mg | Nausea, vomiting, constipation, hypoglicemia, headache, dizziness, diarrhea, fatigue, abdominal pain |
| bupropion | Suppression of appetite | Dopamine and Norepinephrine uptake inhibitor | Upwards titration over 4 weeks up to two tablets/day | (Often used as combination treatment) Nausea, constipation, headache, vomiting, dizziness, insomnia, dry mouth, diarrhea |
| naltrexone |  | Opioid antagonist |  |  |
| sibutramine | Promotion of satiety | Monoamine reuptake inhibitor | Withdrawn | Cardiovascular events (stroke, heart attack), mood swings, seizures |

Additional Table 2. Tukey's post-hoc test significant comparisons following ANOVA on pancreatic insulin levels at Day 42

| **Tukey’s post-hoc comparison** | **Adjusted P.value (Benjamini-Hochberg)** |
| --- | --- |
| FK506 vs naltrexone | 0.02 |
| FK506 vs bupropion_naltrexone | 0.02 |
| naltrexone vs FK506_Buproprion | 0.02 |
| naltrexone vs FK506_naltrexone | 0.006 |
| FK506_buproprion vs bupropion_naltrexone | 0.03 |
| FK506_naltrexone vs bupropion_naltrexone | 0.009 |

Additional Table 3. Percentage of unassigned reads per sample for representative genes fliC, flgE and motA

|  | Unassigned reads (%) | | |
| --- | --- | --- | --- |
| Sample | fliC | motA | flgE |
| 4 | 53.6082 | 33.3333 | NA |
| 14 | 44.3439 | 100 | 0 |
| 25 | 24.9229 | 13.5647 | 3.44828 |
| 33 | 69.3111 | 41.6452 | 36.2031 |
| 40 | 45.866 | 100 | 47.5207 |
| 55 | 51.7922 | 67.4242 | 85.3261 |
| 62 | 56.2468 | 0 | 7.69231 |
| 75 | 30.7377 | 82.3529 | 52.1866 |
| 85 | 42.9543 | 33.6134 | 38.3754 |
| 4H | 11.8442 | 4.08602 | 0 |
| 14H | 100 | NA | NA |
| 25H | 32.8909 | 3.41686 | 7.20461 |
| 33H | 60.6393 | 51.0417 | 27.6786 |
| 40H | 57.453 | 38.0952 | 32.8125 |
| 55H | 80 | NA | 0 |
| 62H | 6.18557 | 0 | 0 |
| 75H | 30.4888 | 25.7062 | 10.8168 |
| 85H | 63.6459 | 26.875 | 56.5972 |
