# Supplementary material for: Pharmacologically induced weight loss is associated with distinct gut microbiome changes in obese rats
Source: BMC Microbiol. 2022 Apr 7;22:91. doi: 10.1186/s12866-022-02494-1 (PMC8988407; doi:10.1186/s12866-022-02494-1)
Supplement: Supplementary file 2 — Additional file 2: Additional report 1. This contains the full details of the study showing the effects of Sibutramine and FK506 (RDC5), bupropion and naltrexone, alone and in combination, on body weight, food and water intake, glycaemic control, pancreatic insulin levels and fat pad, liver and pancreas weights in dietary-induced obese, female Wistar rats. [file 12866_2022_2494_MOESM2_ESM.docx]

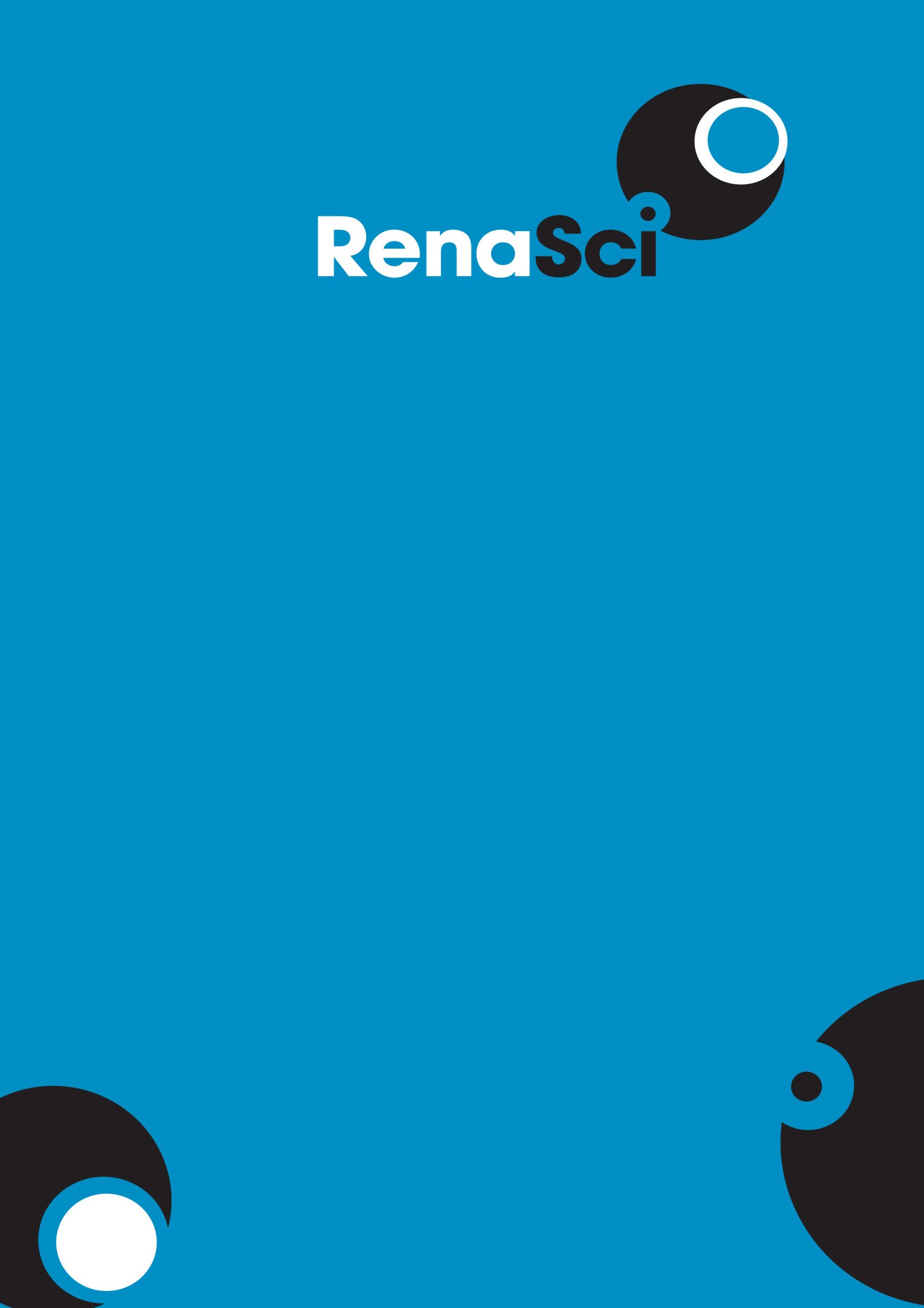


RS1183 Final Report 15-MAR-2017 Page 1 of 149

**Study Title: EFFECTS OF RDC5, BUPROPION AND NALTREXONE, ALONE AND IN COMBINATION, ON BODY WEIGHT, FOOD AND WATER INTAKE, GLYCAEMIC CONTROL, PANCREATIC INSULIN LEVELS AND FAT PAD, LIVER AND PANCREAS WEIGHTS IN DIETARY-INDUCED OBESE, FEMALE WISTAR RATS**

**Study Number: RS1183 (RENASCI)**

**Authors: Sharon C Cheetham and Helen C Jackson**

**Report Date: 15^th^ March 2017**

**STUDY TITLE: EFFECTS OF RDC5, BUPROPION AND NALTREXONE,**

**ALONE AND IN COMBINATION, ON BODY WEIGHT, FOOD AND WATER INTAKE, GLYCAEMIC CONTROL, PANCREATIC INSULIN LEVELS AND FAT PAD, LIVER AND PANCREAS WEIGHTS IN DIETARY-INDUCED OBESE, FEMALE WISTAR RATS**

**STUDY NUMER: RS1183 (RENASCI)**

**SPONSOR:** Chronoscreen

**ADDRESS:** Frieze Farm House Woodstock Road Oxford

OX2 8JX

**AUTHORS:** Sharon C Cheetham and Helen C Jackson

**TESTING FACILITY:** RenaSci Ltd

BioCity Nottingham Pennyfoot Street Nottingham

NG1 1GF UK

Facility for the animal studies:

Bio Support Unit

University of Nottingham Medical School Queens Medical Centre

Nottingham NG7 2UH UK

#### STUDY PERIOD: 10^th^ September 2015 – 15^th^ March 2016 (*in vivo* phase)

STUDY TITLE: EFFECTS OF RDCS, BUPROPION AND NALTREXONE, ALONE AND IN COMBINATION, ON BODY WEIGHT, FOOD AND WATER INTAKE, GLYCAEMIC CONTROL, PANCREATIC INSULIN LEVELS AND FAT PAD, LIVER AND PANCREAS WEIGHTS IN DIETARY-INDUCED OBESE, FEMALE WISTAR RATS

STUDY NUMBER: RS1183 (RENASCI)


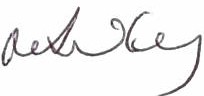
STUDY DIRECTOR:


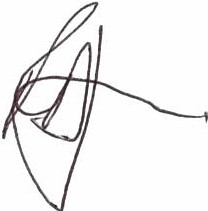
Helen L Rowley (PhD)

SCIENTIFIC REVIEW:

Rob B Jones (PhD)

STATISTICAL ANALYSIS:

Richard Brammer (MSc) *R7P*

**Kevin Thompson (DPhr** *.,,._,tf****4..f;;)--y-***

**CLIENT APPROVAL:** /

Date:
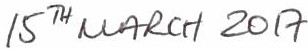


Date: *J(lc-/lJ2. 1-*

Date: *IS-* /Yl 2-o/7


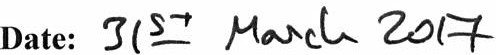


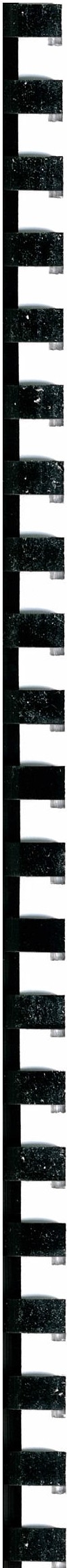


RSI183 Final Report 15-MAR-2017 Page 3 of 149

#### CONTENTS Page

[SUMMARY 7](#_bookmark0)

1. [INTRODUCTION 13](#_bookmark1)
2. [MATERIALS AND METHODS 15](#_bookmark2)
   1. [Animals 15](#_bookmark3)
   2. [Methods 16](#_bookmark4)
      1. [Feeding study 16](#_bookmark5)
      2. [Experimental procedures for the OGTT 18](#_bookmark6)
      3. [Collection of faeces 19](#_bookmark7)
   3. [Termination 19](#_bookmark8)
   4. [Pancreatic insulin 21](#_bookmark9)
   5. [Plasma glucose and insulin 22](#_bookmark10)
   6. [Drugs 22](#_bookmark11)
   7. [Data and statistical analysis 24](#_bookmark12)
3. [RESULTS 27](#_bookmark13)
   1. [General observations 27](#_bookmark14)
   2. [Effects of RDC5, bupropion and naltrexone, alone and in combination, on](#_bookmark15)

[body weight in dietary-induced obese, female Wistar rats 28](#_bookmark15)

- 1. [Effects of RDC5, bupropion and naltrexone, alone and in combination, on](#_bookmark16)

[food intake in dietary-induced obese, female Wistar rats 32](#_bookmark16)

- 1. [Effects of RDC5, bupropion and naltrexone, alone and in combination, on](#_bookmark17)

[water intake in dietary-induced obese, female Wistar rats 36](#_bookmark17)

- 1. [Effects of RDC5, bupropion and naltrexone, alone and in combination, on glucose tolerance in dietary-induced obese, female Wistar rats - Day 37](#_bookmark18)

[OGTT 39](#_bookmark18)

- 1. [Effects of RDC5, bupropion and naltrexone, alone and in combination, on terminal plasma glucose and insulin levels in dietary-induced obese, female Wistar rats 43](#_bookmark19)
  2. [Effects of RDC5, bupropion and naltrexone, alone and in combination, on pancreatic insulin levels in dietary-induced obese, female Wistar rats 44](#_bookmark20)
  3. [Effects of RDC5, bupropion and naltrexone, alone and in combination, on fat pad, liver and pancreas weights in dietary-induced obese, female Wistar rats 44](#_bookmark21)

1. [DISCUSSION 46](#_bookmark22)
2. [REFERENCES 55](#_bookmark23)

[TABLES 60](#_bookmark24)

[FIGURES 132](#_bookmark38)

[ARCHIVES STATEMENT 148](#_bookmark55)

[QUALITY CONTROL STATEMENT 149](#_bookmark56)

**LIST OF TABLES**

[**Table 1 Body weights (g) on each day and comparisons of combination treatments to**](#_bookmark25) [**their individual constituents (Baseline, Weeks 1 to 6) 60**](#_bookmark25)

[**Table 2 Changes in body weights (g) and comparisons of combination treatments to their individual constituents 74**](#_bookmark26)

[**Table 3 Food intake (kJ) on each day and comparisons of combination treatments to**](#_bookmark27) [**their individual constituents (Baseline, Weeks 1 to 6) 78**](#_bookmark27)

[**Table 4 Average daily food intake (kJ/day) and comparisons of combination**](#_bookmark28)

[**treatments to their individual constituents 92**](#_bookmark28)

[**Table 5 Cumulative food intake (kJ) on each day and comparisons of combination**](#_bookmark29) [**treatments to their individual constituents (Weeks 1 to 6) 96**](#_bookmark29)

[**Table 6 Water intake (g) on each day and comparisons of combination treatments to**](#_bookmark30) [**their individual constituents (Baseline, Weeks 1 to 6) 108**](#_bookmark30)

[**Table 7 Average daily water intake (g/day) and comparisons of combination**](#_bookmark31)

[**treatments to their individual constituents 122**](#_bookmark31)

[**Table 8 OGTT plasma glucose 126**](#_bookmark32)

[**Table 9 OGTT plasma insulin 127**](#_bookmark33)

[**Table 10 Terminal plasma glucose and insulin levels and comparisons of combination**](#_bookmark34) [**treatments to their individual constituents 128**](#_bookmark34)

[**Table 11 Pancreatic insulin levels and comparisons of combination treatments to their**](#_bookmark35) [**individual constituents 129**](#_bookmark35)

[**Table 12 Tissue weights (g) and comparisons of combination treatments to their**](#_bookmark36) [**individual constituents – adjusted for Day 1 body weight 130**](#_bookmark36)

[**Table 13 Tissue weights (g) and comparisons of combination treatments to their**](#_bookmark37) [**individual constituents – adjusted for final carcass weight 131**](#_bookmark37)

**LIST OF FIGURES**

[**Figure 1 Effects of RDC5, bupropion and naltrexone, alone and in combination, on**](#_bookmark39)

[**body weight in dietary-induced obese, female Wistar rats 132**](#_bookmark39)

[**Figure 2 Effects of RDC5, bupropion and naltrexone, alone and in combination, on**](#_bookmark40) [**body weight in dietary-induced obese, female Wistar rats expressed as**](#_bookmark40)

[**change in body weight 133**](#_bookmark40)

[**Figure 3 Effects of of RDC5, bupropion and naltrexone, alone and in combination, on**](#_bookmark41) [**daily food intake in dietary-induced obese, female Wistar rats 134**](#_bookmark41)

[**Figure 4 Effects of RDC5, bupropion and naltrexone, alone and in combination, on**](#_bookmark42) [**average daily food intake in dietary-induced obese, female Wistar rats 135**](#_bookmark42)

[**Figure 5 Effects of RDC5, bupropion and naltrexone, alone and in combination, on**](#_bookmark43) [**cumulative food intake per day in dietary-induced obese, female Wistar rats 136**](#_bookmark43)

[**Figure 6 Effects of RDC5, bupropion and naltrexone, alone and in combination, on**](#_bookmark44)

[**daily water intake in dietary-induced obese, female Wistar rats 137**](#_bookmark44)

[**Figure 7 Effects of RDC5, bupropion and naltrexone, alone and in combination, on**](#_bookmark45) [**average daily water intake in dietary-induced obese, female Wistar rats 138**](#_bookmark45)

[**Figure 8 Effects of RDC5, bupropion and naltrexone, alone and in combination, on**](#_bookmark46) [**glucose tolerance in dietary induced obese, female Wistar rats – OGTT time**](#_bookmark46) [**course 139**](#_bookmark46)

[**Figure 9 Effects of RDC5, bupropion and naltrexone, alone and in combination, on**](#_bookmark47) [**glucose tolerance in dietary-induced obese, female Wistar rats – OGTT AUC**](#_bookmark47)

[**(0-60) min 140**](#_bookmark47)

[**Figure 10 Effects of RDC5, bupropion and naltrexone, alone and in combination, on**](#_bookmark48) [**glucose tolerance in dietary-induced obese, female Wistar rats – OGTT AUC**](#_bookmark48)

#### [(0-120) min 141](#_bookmark48)

#### [Figure 11 Effects of RDC5, bupropion and naltrexone, alone and in combination, on](#_bookmark49) [glucose tolerance in dietary-induced obese, female Wistar rats – OGTT](#_bookmark49)

#### [AUCB2 (0-60) min 142](#_bookmark49)

#### [Figure 12 Effects of RDC5, bupropion and naltrexone, alone and in combination, on](#_bookmark50) [glucose tolerance in dietary-induced obese, female Wistar rats – OGTT](#_bookmark50)

#### [AUCB2 (0-120) min 143](#_bookmark50)

#### [Figure 13 Effects of RDC5, bupropion and naltrexone, alone and in combination, on](#_bookmark51) [terminal plasma glucose and insulin levels in dietary-induced obese, female](#_bookmark51) [Wistar rats 144](#_bookmark51)

#### [Figure 14 Effects of RDC5, bupropion and naltrexone, alone and in combination, on](#_bookmark52) [pancreatic insulin levels in dietary induced obese, female Wistar rats 145](#_bookmark52)

#### [Figure 15 Effects of RDC5, bupropion and naltrexone, alone and in combination, on](#_bookmark53) [tissue weights in dietary-induced obese, female Wistar rats – adjusted for](#_bookmark53)

#### [Day 1 body weight 146](#_bookmark53)

#### [Figure 16 Effects of RDC5, bupropion and naltrexone, alone and in combination, on](#_bookmark54) [tissue weights in dietary induced obese, female Wistar rats – adjusted for](#_bookmark54)

#### [final carcass weight 147](#_bookmark54)

**EFFECTS OF RDC5, BUPROPION AND NALTREXONE, ALONE AND IN COMBINATION, ON BODY WEIGHT, FOOD AND WATER INTAKE, GLYCAEMIC CONTROL, PANCREATIC INSULIN LEVELS AND FAT PAD, LIVER AND PANCREAS WEIGHTS IN DIETARY-INDUCED OBESE, FEMALE WISTAR RATS**

#### SUMMARY

This study investigated the effects of repeated administration of the prophylactic immunosuppressant agent, RDC5 (Prograf®; tacrolimus), alone and in combination with the noradrenaline and dopamine reuptake inhibitor, bupropion (20 mg/kg ip qd Day 1-25/bid Day 26-43), or the opioid antagonist, naltrexone (1 mg/kg ip), on a variety of parameters in dietary-induced obese, female Wistar rats. Bupropion and naltrexone were given alone and in combination. The 5-hydroxytryptamine and noradrenaline reuptake inhibitor, sibutramine (5 mg/kg po), was used for comparison. We have previously shown that RDC5 (1 mg/kg po) reduced body weight in rats but increased plasma glucose and reduced pancreatic insulin content. This study evaluated whether a lower dose of RDC5 (0.6 mg/kg po) could be combined with a drug with a complementary mode of action, to produce weight-loss without these adverse effects. A combination of bupropion and naltrexone is currently marketed for obesity (Contrave®). Sibutramine has also been used clinically to manage this condition.

Chronic administration of RDC5 produced a gradual decrease in body weight compared to the vehicle-treated control group. This response occurred mainly during the first 2 weeks of treatment. Body weights of rats given RDC5 were significantly lower than controls from Day 6 onwards. On Day 36 (before an overnight fast for an oral glucose tolerance test) and Day 43 (termination), body weights of the rats given RDC5 were 6.0% and 4.8% lower than those of the control group, respectively. Sibutramine produced a marked, significant decrease in body weight on Day 2. Body weights of the sibutramine group remained significantly lower than controls on every day of treatment. The effects of sibutramine on body weight occurred mainly during the first week of treatment

before body weights plateaued. On Days 36 and 43, body weights of the sibutramine group were 9.8% and 10.4% lower than controls, respectively. In comparison, bupropion and naltrexone, alone or in combination, had little effect on daily body weight, though the combination of bupropion and naltrexone produced significant weight-loss during Week 5, ie following the increase in dose of bupropion on Day 26.

The combination of RDC5 with bupropion significantly reduced body weight compared to the controls from Day 8. Body weights of animals given RDC5 with bupropion were similar to those of the RDC5 group (except during Week 6 when the combination group lost weight compared to weight gain in the RDC5 group). On Days 36 and 43, body weights of the animals given RDC5 with bupropion were 5.5% and 6.9% lower than controls, respectively. The combination of RDC5 with naltrexone did not significantly alter body weight. These animals weighed only 3.0% and 1.8% less than controls on Days 36 and 43. Thus, there was no evidence from this study that the weight-loss produced by RDC5 (roughly half that of sibutramine) could be potentiated by either bupropion or naltrexone.

RDC5 significantly reduced daily food intake on Days 6 and 9, average daily food intake during Week 1 (by 11.6%) and cumulative food intake on each day from Days 6 to 15 and on Day 18. Thus, the gradual weight-loss produced by RDC5 during the first two weeks of the study appeared to be due, at least in part, to reduced food intake. RDC5 also significantly increased average daily food intake during Week 6 (by 11.7%). Accordingly, RDC5 did not significantly reduce average daily food intake overall or cumulative food intake on Day 42. In comparison, daily food intake was sporadically increased by bupropion (Days 12, 19 and 38) and naltrexone alone (Days 11 and 16) and bupropion significantly increased average daily food intake during Week 4 (by 14.0% compared to controls), ie before the dose of bupropion was increased on Day 26. Sibutramine significantly reduced food intake by 86.4% on Day 1. Food intake of the sibutramine group remained significantly lower than controls on Days 2 to 9 though the magnitude of the hypophagic response decreased on repeated administration. Sibutramine significantly reduced average daily food intake

during Week 1 by 47.8% and over the entire drug treatment period by 11.7%. Cumulative food intake of the sibutramine group was significantly lower than controls on each day and reduced by 12.8%, compared to controls, on Day 42. Thus, the reduction in body weight produced by sibutramine would appear to be largely due to reduced food intake.

Although the combination of RDC5 with bupropion produced a similar reduction in body weight to RDC5, it had relatively little effect on daily, average daily or cumulative food intake compared to the control group suggesting that mechanisms other than reduced energy intake may have contributed to the observed weight-loss. In comparison, the combination of RDC5 with naltrexone significantly increased daily food intake on Days 22, 31, 37 and 38 and average daily food intake during Week 6 (by 15.3%) but did not alter food intake averaged over the entire study or cumulative food intake on any day.

The combination of bupropion and naltrexone significantly increased daily food intake on Days 8, 10, 12, 16 and 17, average daily food intake during Week 2,

Week 3 and Weeks 1-4 (by 17.2%, 12.0% and 10.0%, respectively) and cumulative food intake on Day 17 to 24 but did not alter food intake overall. Thus, the increases in food intake induced by the combination of these two drugs occurred before the dose of bupropion was increased on Day 26.

In general, the different drugs did not produce any overt behavioural and physiological effects which could have altered body weight or food intake in a non-specific manner or any drug-related abnormalities at post-mortem.

RDC5 significantly decreased daily water intake on Days 3, 5, 6, 9, 13, 14 and 27 and average daily water intake during Weeks 1 and 2 by 17.1% and 14.6%, respectively, compared to the control group. Rats are prandial drinkers so this could be a consequence of reduced food intake (although this was not always significant). Bupropion significantly increased daily water intake over control levels on Days 2 and 5 but had no effect on average daily water intake per week or overall. Naltrexone significantly decreased daily water intake on Days 9, 11,

14, 24, 29, 31, 35 and 36 and average daily water intake during Week 2 (by

16.7%), Week 5 (18.9%), Weeks 5-6 (15.7%), Weeks 1-5 (12.5%) and overall (12.6%). In comparison, sibutramine significantly decreased daily water intake on Days 1 and 2 (by 56.5% and 22.8%, respectively) but significantly increased daily

water intake on Days 17, 20, 21, 24, 25, 30, 32, 37 and 41, compared to the control group and also significantly increased average daily water intake during Week 3 Week 4 and overall (by 20.2%, 21.9% and 12.4%), a response that has been observed before in dietary-induced obese rats. The combination of RDC5 and bupropion significantly decreased daily water intake on Days 8, 9, 11 and 14 and average daily water intake during Week 2 (by 17.5%) and Weeks 1-4 (13.0%) whereas the combination of RDC5 with naltrexone significantly decreased daily water intake on Days 1, 2, 11, 14 and 36 and average daily water intake during Week 1 (by 13.1%) compared to the controls. These responses are consistent with the initial inhibitory effects of RDC5 on water intake. In comparison, the combination of bupropion and naltrexone had no effect on daily or average daily water intake except for a transient increase over control levels on Day 37.

An OGTT was conducted on Day 37, following on overnight fast. Baseline blood samples were taken before (B1) and one hour after drug treament (B2) and 10, 20, 30, 45, 60 and 120 min following a glucose challenge (2 g/kg po). Plasma insulin levels of the fasted rats were significantly reduced in the RDC5 group at B1 and plasma glucose levels were reduced in the sibutramine group at B2 but increased by bupropion with naltrexone. The main findings of the OGTT were that RDC5, alone and in combination with bupropion or naltrexone, markedly and significantly reduced plasma insulin levels following the glucose load while having little effect on plasma glucose levels (as shown by the area under the curve (AUC) and AUC above the B2 baseline (AUCB2) data for the 0-60 and 0-120 min time-points). The decrease in plasma insulin AUC and AUCB2 of the animals given the combination of RDC5 with bupropion or naltrexone could be attributed to RDC5 as bupropion and naltrexone, alone or in combination, had little effect on these parameters. Plasma insulin AUC and AUCB2 were reduced to a similar degree by sibutramine. Sibutramine also significantly reduced plasma

glucose AUC (0-60 min) and AUC (0-120 min) though not the area under the curve above the B2 baseline.

The improvement in insulin sensitivity in the sibutramine group could reflect the ability of the drug to reduce body weight. However, RDC5 alone, the combination of RDC5 with bupropion and the combination of RDC5 with naltrexone significantly reduced pancreatic insulin content by approximately 50%, which could have accounted for the reduction in plasma insulin levels in these treatment groups following the glucose challenge. Bupropion and naltrexone, alone and in combination, did not significantly alter pancreatic insulin content.

RDC5 significantly reduced plasma insulin levels in freely-feeding rats (terminated 3 h after dosing on Day 43), consistent with the decrease in plasma insulin in fasted rats. However, RDC5 did not increase plasma glucose in fed or fasted animals. Reductions in terminal plasma insulin were also observed in animals given RDC5 with naltrexone, naltrexone alone and bupropion with naltrexone. Sibutramine and bupropion did not significantly alter terminal plasma glucose and insulin levels compared to the control group.

RDC5, bupropion, naltrexone and the combinations of RDC5 with bupropion, RDC5 with naltrexone and bupropion with naltrexone did not significantly alter retroperitoneal fat pad or pancreas weights regardless of whether results were adjusted for body weights on Day 1 or final carcass weights. The fact that RDC5 and RDC5 with bupropion did not reduce fat pad weights reflects the relatively small decrease in body weight produced by these drug treatments compared to the control group. These drugs did not significantly alter liver weights, adjusted for body weight on Day 1. However, livers of the bupropion, RDC5 with bupropion and bupropion with naltrexone groups were significantly higher than expected (increased by 10.9%, 16.1% and 10.5%) when given the effects of these drug treatments on final carcass weight. RDC5 and naltrexone, alone or in combination, had no effect on liver weight adjusted for final carcass weight.

In summary, chronic administration of RDC5 (0.6 mg/kg po) produced a modest reduction in body weight in dietary-induced obese, female Wistar rats, an animal model with excellent predictive validity for weight-loss in the clinic. The reduction in body weight produced by RDC5 was approximately half of that produced by sibutramine (5 mg/kg po). The weight-loss produced by RDC5, could be explained at least in part, by reduced food intake. RDC5 significantly reduced plasma insulin levels during an OGTT and plasma insulin at the B1 baseline time-point of the OGTT and at termination but had little effect on plasma glucose in fed or fasted rats or following the glucose challenge. While the decrease in plasma insulin levels could indicate improved insulin sensitivity, the same dose of RDC5 significantly reduced pancreatic insulin levels by approximately 50%. This would be an unwanted adverse effect in obese patients who are often insulin resistant and may also be diabetic. Greater weight-loss may have been achieved in the model at higher doses of RDC5. However, it is clear that the drug has a narrow therapeutic index and is unlikely to produce meaningful weight-loss without detrimental effects on pancreatic function. There was no evidence that the effects of RDC5 on body weight could be potentiated by bupropion or naltrexone. In conclusion, these findings do not support the development of RDC5 for the treatment of obesity either alone or in combination with bupropion or naltrexone.

#### EFFECTS OF RDC5, BUPROPION AND NALTREXONE, ALONE AND IN COMBINATION, ON BODY WEIGHT, FOOD AND WATER INTAKE, GLYCAEMIC CONTROL, PANCREATIC INSULIN LEVELS AND FAT PAD, LIVER AND PANCREAS WEIGHTS IN DIETARY-INDUCED OBESE, FEMALE WISTAR RATS

#### INTRODUCTION

The obesity epidemic is no longer restricted to Western cultures but has a worldwide impact affecting countries such as Mexico, Brazil, China and Russia^1^. Obesity is also on the increase in Africa and is strongly linked to urbanisation.^2^ Diagnosis is based on a body mass index (BMI) of at least 30 kg/m^2^. In 2014, the World Health Organisation (WHO) estimated that over 600 million men and women were obese worldwide^3^. Furthermore, increases in childhood obesity are enormous with over 42 million children under the age of 5 being recorded as overweight or obese in 2013^3^. Obesity is a driver for several life-threatening disorders including dyslipidaemia, atherogenesis, hypertension and type 2 diabetes all of which are risk factors for cardiovascular disease and mortality^4-8^. Obesity not only pre-disposes individuals to some cancers including those of the colon, kidney, oesophagus and breast but has been implicated in resistance to chemotherapy^9,10^. In addition, obesity may lead to psychological problems such as depression and non-life threatening diseases such as arthritis and sleep apnoea^11^. Weight loss has been shown to result in clinically-relevant improvements in co-morbidities, for example, blood pressure, triglycerides, HDL cholesterol and glucose control are all improved in patients with type 2 diabetes achieving even modest weight loss of 5-10%^12^.

There are a limited number of prescription medicines available for the treatment of obesity. Recently, Contrave®, a combination of a slow release formulation of bupropion and naltrexone, has received regulatory approval in the US. Bupropion is an antidepressant which inhibits both noradrenaline and dopamine reuptake. Naltrexone is an opioid antagonist. Both compounds have been shown to produce

weight loss in man but efficacy is limited^13^. However, in combination the weight- loss is enough to meet regulatory guidelines^13,14,15^.

Male Sprague-Dawley rats fed a high fat diet become overweight and exhibit increased body fat and mild insulin and leptin resistance^16^. Accordingly, they provide a useful screen for assessing the efficacy and potential utility of putative obesity treatments. We have recently shown that repeated administration of a range of doses of the prophylactic immunosuppressant agent, RDC5 (Prograf®, tacrolimus), significantly reduced body weight in male Sprague-Dawley rats fed a high-fat diet^17^. However, the reduction in body weight achieved with the highest doses of RDC5 (1 mg/kg po) was approximately half that seen with the previously available antiobesity agent, the 5-hydroxytryptamine (5-HT) and noradrenaline reuptake inhibitor, sibutramine (7.5 mg/kg po)^17^. Furthermore, at high doses, RDC5 significantly decreased pancreatic insulin content and increased terminal plasma glucose levels in the male rats on high-fat diet. These effects are undesirable in obese patients as they are often also diabetic.

The aim of the current study was to investigate the effects of chronic administration of RDC5 on body weight, food and water intake, glycaemic control, pancreatic insulin content, terminal glucose and plasma levels and pancreas, liver and fat pad weights in dietary-induced obese, female Wistar rats ie a more rigorous animal model of obesity^18^. In this paradigm, female Wistar rats are exposed to a simplified cafeteria diet of powdered high-fat diet (containing 20% lard), ground chocolate and ground salted peanuts to mirror the typical ‘junk’ food diet of the Western world. Female rats are used in this model as they have flatter growth curves than males that continue to gain weight over their life- cycle. After several months on the simplified cafeteria diet, the ‘middle-aged’ female rats become weight-stable and can be used to assess the ability of drugs to reduce body weight, as opposed to reduce the weight gain observed in male rats on high-fat diet which are still in an active growth phase. Dietary-induced obese, female Wistar rats display hyperphagia, impaired glucose tolerance and significantly elevated basal leptin and insulin levels, compared to lean

animals^18,19^. This model has been shown to have excellent predictive validity for weight-loss in the clinic^18^.

In the current study, RDC5 was given in a moderate dose of 0.6 mg/kg po to evaluate whether its antiobesity actions could be separated from potential adverse effects and also to see if its ability to produce weight-loss could be potentiated by giving it in combination with bupropion or naltrexone, ie drugs with complementary modes of action. Sibutramine was used as a reference compound.

#### MATERIALS AND METHODS

#### Animals

One hundred and six female Wistar rats (weight range 200-250 g) were ordered from Charles River, Margate, Kent (one set of 100 for the feeding study and 6 spares) to arrive in the Bio Support Unit of the University of Nottingham Medical School on 10/09/2015. The rats were housed in pairs in polypropylene cages with solid floors and sawdust bedding at a temperature of 21±4°C and 55±20% humidity (actual range 18.7-25.0°C and 30-67% humidity). Animals were maintained on a reverse phase light-dark cycle (lights off for 8 h from 09.30-17.30 h) during which time the room was illuminated by red light. Animals had free access to powdered high fat diet (VRF1 plus 20% lard), ground chocolate, ground peanuts and tap water at all times. The three different diets were contained in separate glass feeding jars with aluminium lids (Solmedia Laboratory Suppliers, Romford, Essex). Each lid had a 3-4 cm hole cut in it to allow access to the food. Animals were housed in pairs for 17 weeks for the induction of obesity, 87 rats were then selected for the feeding study on the basis of body weight (Week 17) and condition. Animals were then individually housed in polypropylene cages with metal grid floors to enable the food intake of each rat to be recorded. Each cage contained a small amount of paper bedding for warmth, environmental enrichment and to provide an area for animals to get off the wire grid floor. Polypropylene trays with cage pads were placed beneath each cage to detect food

spillage. Animals were maintained under these conditions (individual housing and wire grid floors with paper bedding) for two further weeks before the start of experimentation. Other environmental conditions remained the same (21±4°C and 55±20% humidity; actual range during the study 18.6-22.1°C and 25-54% humidity) except that the light-dark cycle was adjusted slightly to allow for a dosing period with lights-off for 8 h from 10.00-18.00 h.

#### Methods

#### Feeding study

The feeding study included a 7-day baseline run-in period. During this time, rats were weighed (to the nearest 0.1 g using a top-pan balance) and dosed once a day with vehicle po and ip starting at 08.30 h. Towards the end of the baseline period, animals were allocated into 8 treatment groups (n = 12 for the control group and n

= 10 for 7 drug-treated groups) based on body weights on Day -2 and average food and water intake during the first 4 days of the baseline period. The five spare rats continued to be dosed with vehicle in case they were required before the start of drug treatment. One of the spare rats was used to generate blank plasma which was supplied to XenoGesis Ltd with pharmacokinetic (PK) samples collected at the end of the study (see Section 2.3). A terminal blood sample (5 ml) was taken from this animal by cardiac puncture into a 15 ml centrifuge tube (SLS, 241814) containing EDTA solution (108.7 µl of a 93 mg/ml EDTA solution) and kept on wet ice for <30 min. The EDTA solution was prepared by dissolving solid EDTA disodium salt dihydrate (Sigma Aldrich) in water. The blood sample was then centrifuged and 50 µl aliquots of plasma (60) were pipetted into a blue 96-well plate which was kept on wet ice throughout. The 96-well plate containing the samples was stored frozen at -80^o^C until the end of the study.

On Days 1-43, rats were given either the castor oil/dehydrated alcohol vehicle, RDC5 or sibutramine by the po route and either the saline vehicle, bupropion, naltrexone or bupropion and naltrexone by the ip route. Dosing began at approximately 08:30 h each day i.e. shortly before the onset of the dark period to

span the period either side of the lights going off at 10.00 h. This strategy was taken to maximise the inhibitory effects of the test drugs on food intake. From Day 26, the dose of bupropion was increased by giving it bid instead of once a day, ie rats were dosed with either the saline vehicle ip or bupropion ip approximately 8 h after dosing at 0 h. Treatment groups were therefore as indicated below.

|  | **Days 1-43** | | **Days 26-42** |  |
| --- | --- | --- | --- | --- |
| **Group** | **Treatment 1 (t = 0 h)**  **(4 ml/kg po)** | **Treatment 2 ( t= 0 h)**  **(2 ml/kg ip)** | **Treatment 3 (t = 8 h)**  **(2 ml/kg ip)** | **n** |
| A | Vehicle po | Vehicle ip | Vehicle ip | 12 |
| B | RDC5 0.6 mg/kg po | Vehicle ip | Vehicle ip | 10 |
| C | Vehicle po | Bupropion 20 mg/kg ip | Bupropion 20 mg/kg ip | 10 |
| D | Vehicle po | Naltrexone 1 mg/kg ip | Vehicle ip | 10 |
| E | RDC5 0.6 mg/kg po | Bupropion 20 mg/kg ip | Bupropion 20 mg/kg ip | 10 |
| F | RDC5 0.6 mg/kg po | Naltrexone 1 mg/kg ip | Vehicle ip | 10 |
| G | Vehicle po | Bupropion 20 mg/kg ip + Naltrexone 1 mg/kg ip | Bupropion 20 mg/kg ip | 10 |
| H | Sibutramine 5 mg/kg po | Vehicle ip | Vehicle ip | 10 |

Vehicle for po administration was castor oil/dehydrated alcohol. Vehicle for ip administration was saline.

Rats, feeding jars and water bottles were weighed (to the nearest 0.1 g) every day at 0 h. At each reading, the tray below each cage was examined for spilt food, which was returned to the feeding jar before it was weighed. In general, spillage of food from the feeding jars was negligible. The animals were weighed for dosing purposes at the afternoon dose (Day 26 onwards) but no further readings were taken at this time.

During the baseline and drug treatment periods, all animals were examined every day before and after the dosing period. Any overt behavioural/physiological effects or other relevant observations regarding the condition of the animals were recorded manually.

Due to the size of the study, animals were further divided into two equal cohorts, each containing 41 rats which were balanced for treatment as much as possible (n = 6 for the control group and n = 5 for the 7 drug treatment groups). Dosing of the two cohorts was staggered by one day and all data were pooled to give n of 10-12.

#### Experimental procedures for the OGTT

An oral glucose tolerance test (OGTT) was performed on Day 37 for each cohort of rats (n = 5-6, total of 41 animals per OGTT). Rats were deprived of food overnight before the OGTT but allowed free access to water. Thus, on Day 36, feeding jars were removed from the home cage (commencing at 16.00 h) and weighed. The following day (Day 37), the 41 fasted animals were subject to the OGTT. The animals were moved to a separate room, which was maintained under normal lighting and contained heated chambers which were used to aid the blood- sampling procedure. Animals were cannulated (lateral tail vein). When all of the rats had been cannulated and the cannulae had been checked, a blood sample was taken (Baseline 1 timepoint (B1), commencing at 08.42 h). Animals were then dosed with vehicle or drug as appropriate (60 min before the glucose challenge). Four minutes before the glucose challenge, an additional blood sample was taken (Baseline 2 timepoint (B2). Thus, baseline blood samples were taken both before vehicle/drug treatment and before the glucose challenge at 0 min. Animals were then dosed orally with D-glucose (2 g/kg) and further blood samples were taken 10, 20, 30, 45, 60 and 120 min later. Between blood-sampling animals were returned to the home cage with free access to water but not food. At all time-points, blood samples (~90 μl) were collected into lithium heparinised tubes (Sarstedt Microvette CB300) and plasma separated by centrifugation (2,400 g for 5 min at 4°C) to produce a single aliquot of plasma (~45 μl) which was frozen (-80°C) and subsequently assayed for glucose and insulin as described below. After the final tail vein bleed, the cannulae were removed and feeding jars were returned to the animals. All cages were then moved back to the holding room (reverse-phase lighting). On the day of the OGTT, the other cohort of rats was dosed as normal.

#### Collection of faeces

On the mornings of Days -3, 15 and 42, immediately prior to dosing, a fresh cage pad was placed on the tray beneath the raker cage. The following morning (i.e. 24 h later) approximately 15 faecal pellets were removed from the tray (or any pellets available if less than 15) and placed into a 50 ml tube and stored at -80°C prior to shipment to :-

Professor Jane Mellor c/o Anitha Nair

Department of Biochemistry South Parks Road

Oxford OX1 3QU

#### Termination

On the morning of Day 43, final readings took place. Animal were dosed to a timed schedule. Whole blood (250 µl) pharmacokinetic (PK) samples were taken from the tail vein by venepuncture 3 h post-drug into EDTA tubes (Sarstedt Microvette CB300) and frozen on dry-ice as whole blood. Samples were shipped to :-

Mr Gary Laban Bioanalytical Unit

Seirian Laboratories (formerly Simbec Research Ltd) Cardiff Road

Merthyr Tydfil CF48 4DR

A second PK blood sample (>230 µl) was also taken from the tail vein into plasma tubes (Alpha CP5915) containing EDTA solution (5 µl, 93 mg/ml) and the samples keep on wet ice for <30 mins. The blood samples were then centrifuged

and 2 x 50µl aliquots of plasma were pipetted into two blue 96-well plates which were kept on wet ice throughout (with the exception of rat 1C (bupropion alone) when only one plasma sample was provided). The 96-well plates containing the samples were stored frozen at -80^o^C prior to delivery to :-

Mr Jim Shelton XenoGesis Ltd BioCity Nottingham NG1 1GF

XenoGesis were also supplied with a two 1 ml ampoules containing RDC5; a sample (20 ml) of the vehicle ((200 mg/ml (20% v/v) polyoxyethylene hydrogenated castor oil (Cremaphor) and 638 mg/ml (80% v/v) used for RDC5; bupropion hydrocholoride (10 mg), naltrexone hydrochloride (10 mg) and the blank plasma mentioned in Section 2.2.1.

Following blood-sampling for PK on Day 43, animals were killed to a timed schedule (by rising CO_2_ concentration to minimise any fluid loss). A terminal blood sample was taken by cardiac puncture into 5 ml EDTA-coated tubes (Sarstedt; Cat. No. 32.332) and spun in a centrifuge (at 1,900 g for 5 min at 4ºC). Terminal plasma samples were stored (five aliquots per rat) frozen at -80ºC and kept for analysis of plasma glucose and insulin by RenaSci. Remaining terminal plasma samples were stored at -80°C.

A brief post-mortem was then performed on the animals and any unusual observations were recorded. The pancreas and liver were removed and weighed (Groups A-G only). The pancreas from half of the animals (n = 2-3 per cohort, 5- 6 in total; allocated by a Statistician) were homogenised in acid ethanol solution and processed prior to pancreatic insulin analysis. The pancreas from the remaining animals (n = 2-3 per cohort, 5-6 in total) and the left lobe of the liver (n

= 5-6 per cohort, 10-11 in total) were placed into separate vials of formalin and fixed for 24 h and 5-7 days, respectively (the remaining liver was returned to the

carcass). The samples were prepared using a Tissue Tek VIP processor (using graded alcohols to dehydrate and xylene as a clearant), impregnated with paraffin histo-wax prior to embedding in fresh histo-wax. Retroperitoneal fat pads were also removed and weighed (Groups A-G only). These fat pads are used because they are distinct and therefore any drug-induced changes in the size of the fat pad can be easily detected. Fat pads were returned to the carcasses which were weighed (final carcass weights), frozen and stored at -20°C.

Samples from this study (including terminal plasma, wax blocks (liver and pancreas samples), pancreatic insulin samples, carcasses (Groups A and B only) and plasma plates from XenoGesis) were shipped to the client in October 2016.

#### Pancreatic insulin

The pancreas from Groups A to G (n = 5-6 per group) were immediately homogenised in 10 ml of acid/ethanol solution (0.18N HCl/70% ethanol) and then incubated overnight at 4ºC. The following morning, homogenates were centrifuged at 2,600 g for 5 min. The supernatant was removed and centrifuged again after which supernatant was stored at -80ºC until required.

Total insulin concentration was determined as a single replicate using a Mercodia ultrasensitive rat insulin ELISA kit (Catalogue Number 10 1251-01). The assay was performed as per the manufacturer’s instructions except that additional insulin standards (1.50 and 1.75 ng/ml) were included to extend the range of the assay. Standards were assayed in duplicate whilst all samples were determined as a single replicate following a 30,000 dilution in saline. A five parameter logistic curve of optical density against concentration was used to create a standard curve using statistical analysis software (SAS®). Sample concentrations were estimated from the optical density readings using this curve fit. Pancreatic insulin content was then calculated as µg insulin per g (wet weight) of tissue

#### Plasma glucose and insulin

Plasma glucose was determined using a clinical glucose assay reagent (Thermo Electron Infinity stable reagent TR15421) in a 96-well format. An 11.1 mM glucose stock (Fisher D-Glucose anhydrous: G/0450/53 Lot 1165417) was prepared in 0.1% benzoic acid and stored in the fridge. The glucose stock was diluted in saline to create a series of standard concentrations from 0.25 mM to 10 mM. All determinations were performed in duplicate. Plasma samples were diluted in saline prior to analysis (10x). Standards and unknowns were pipetted into 96-well plates (Alpha Laboratories, flat bottomed FX9200) as 20 µl additions. The reaction was started by adding 250 µl of assay reagent followed by incubation at 37ºC for 10 to 15 min after which optical density at 340 nm and 400 nm (correcting wavelength) was determined using a Molecular Devices SpectraMax 340PC^384^ microplate reader. The optical density change was then calculated by subtraction.

Insulin was determined as a single replicate using a Mercodia ultrasensitive rat insulin ELISA kit (Catalogue Number 10-1251-01). The assay was performed as per the manufacturer’s instructions except that additional insulin standards (1.50 and 1.75 ng/ml) were included to extend the range of the assay. Standards were assayed in duplicate whilst all samples were determined as a single replicate. Plasma samples were diluted in saline prior to analysis (10x).

A five-parameter logistic curve of optical density against concentration was used to create standard curves using statistical analysis software (SAS®). Plasma concentrations were estimated from the curves.

#### Drugs

RDC5 (Prograf®, tacrolimus, Astellas Pharma GmbH; Batch 5A3310A) was supplied by the client as preformulated 1 ml ampoules (each containing a clear liquid of RDC5 5 mg/ml in 20% v/v castor oil and 80% v/v alcohol). Ampoules were stored in the original package in order to protect them from light, at below

25°C (placed in a fridge on arrival at RenaSci). The vehicle for RDC5 (200 mg/ml (20% v/v) polyoxyethylene hydrogenated castor oil (Cremaphor) and 638 mg/ml (80% v/v) dehydrated alcohol) was supplied as a stock solution by Quay Pharma, UK (Batch CHR/006/148) on behalf of the client and stored at room temperature.

RDC5 was prepared to achieve the required concentration of 0.15 mg/ml in a final solution comprising 1% polyoxyethylene hydrogenated castor oil and 4% dehydrated alcohol in water. Thus, RDC5 was first diluted in the stock oil/alcohol vehicle and then diluted with deionised water 1:20 to give a clear solution (pH 7.1). Dosing solutions of RDC5 were prepared fresh each day, foil-wrapped to protect from light at all times and only glass, polyethylene and polypropylene materials (e.g. syringes and gavages) were used for preparing and dispensing solutions to prevent loss of compound due to adherence to plastics. Contact with PVC was avoided.

The stock solution 20% v/v polyoxyethylene hydrogenated castor oil and 80% v/v dehydrated alcohol was diluted each day with deionised water (1:20) to create a clear dosing solution for the vehicle-treated control groups (1% v/v polyoxyethylene hydrogenated castor oil and 4% v/v dehydrated alcohol; pH 6.9).

Bupropion hydrochloride (Catalogue Number 2831; Batches 2A/179240 and 2A/180641 (Days 29 and 30 pm dose onwards); naltrexone hydrochloride (Catalogue Number 0677; Batches 6A/178810 and 6A/174974 (Days 30 and 31 onwards) and sibutramine hydrochloride (Catalogue Number 2290; Batch 5A/166755) were purchased from Tocris Bioscience. Doses were expressed as the free base (using a free base/salt correction factor of 1.15 for bupropion and naltrexone and 1.195 for sibutramine). Bupropion and naltrexone were supplied as a white powder and dissolved in 0.9% saline (pH 7.1) with the help of a vortex (2 min) and a sonic bath (10 min) to produce a clear solution (pH 6.2 for bupropion, pH 6.9 for naltrexone and pH 6.2 for the mixture of bupropion and naltrexone (second treatment of Group G)). Sibutramine was supplied as a white powder and added to the stock oil/alcohol vehicle used for RDC5 and diluted with deionisied water 1:20 to achieve the required dosing concentration and volume

(1.49 mg/ml in 1% v/v oil and 4% v/v alcohol). The sibutramine solution was agitated and then sonicated for 10 min to produce a clear liquid (pH 5.9).

RDC5, sibutramine and the castor oil/ethanol vehicle were administered orally (by gavage) using a dose volume of 4 ml/kg. Bupropion, naltrexone, the mixture of bupropion and naltrexone and the 0.9% saline vehicle were administered intraperitoneally (ip) using a dose volume of 2 ml/kg.

#### Data and statistical analysis

*Feeding study:* The body weight data (g) have been expressed as daily body weights and change in body weight from Days 1-8, 8-15, 15-22, 22-26, 26-36, 36-

43, 1-26, 26-43, 1-36 and 1-43 (due to the change in treatment of bupropion from qd to bid on Day 26 and the overnight fast on Day 36 for the OGTT on Day 37). Daily food (kJ) and water (g) intake were calculated for each animal. Food intake was calculated using the food energy values (20.79 kJ/g (high-fat chow), 23.44 kJ/g (chocolate) and 30.34 kJ/g (ground peanuts). Food and water intake have also been expressed as average daily intakes from Days 1-7, 8-14, 15-21, 22-25,

26-35, 36-42, 1-25, 26-42, 1-35 and 1-42. In addition, food intake (kJ) has been expressed as cumulative food intake on each day of treatment.

The statistical methods assumed that data were normally distributed with equal variance in the groups. There were some abnormally high and low values. For food intake, these abnormal values were not considered to compromise the analysis and so were included. Due to the large number of extreme values for water intake, this variable has been analysed by robust regression.

Body weight data on each day (except Day 1) were analysed by two-way analysis of covariance with treatment and cohort as factors and body weight on Day 1 as the covariate, because the Day 1 body weight was measured before drug treatment commenced. Weekly and overall changes in body weight were analysed in a similar manner.

Food intake (kJ) on each day and cumulative food intake (kJ) on each day were analysed by two-way analysis of covariance with treatment and cohort as factors and average food intake during the baseline phase (Days -6 to 0) as the covariate. Weekly and overall average daily food intake (kJ) were analysed similarly.

Water intake (g) on each day was analysed by a robust regression model using M estimation, Huber weighting, using the default parameter c = 1.345. The model included treatment and cohort as factors and average daily water intake over the baseline phase (Days -6 to 0) as the covariate. Weekly and overall average daily water intake (g) were analysed similarly.

*Plasma analysis:* The statistical methods assume that data are normally distributed with equal variance in the groups. Initial Shapiro-Wilk tests for normality of residuals from this study showed that both log(glucose) and log(insulin) were usually more normally distributed than glucose and insulin. However, the Shapiro-Wilk tests for log(glucose) and log(insulin) in the OGTTs were still sometimes significant, so robust regression was used to downweight any possible outliers in the analysis of these variables.

Plasma levels of glucose and insulin for each treatment group were compared at each time point of the OGTT. Results of the OGTT have also been expressed as the area under the curve (AUC) for 0-60 min and 0-120 min calculated as

*AUC*120 min = 124 (2*t*0 min + 4*t*10 min + 4*t*20 min + 5*t*30 min + 6*t*45 min + 15*t*60 min + 12*t*120 min )

*AUC*60 min = *AUC*120 min − 12 (*t*60 min + *t*120 min )

A second AUC, which included only the data above the B2 baseline (AUCB2) was also determined. The AUCB2 (0-60 min) and AUCB2 (0-120 min) values were calculated as

AUC120min - 2*t*0*h* and AUC60min - *t*0*h*.

Thus, the AUC data includes the AUC immediately under the curve and also all the data below to 0 (mM glucose or ng/ml insulin). The AUCB2 includes only the

AUC immediately under the curve ie there is no dampening down of the data by incorporation of the results below the B2 baseline. However, this does mean that there is the possibility of negative areas if the data for any time-points drops below B2.

For analysis of the OGTTs, a log transformation was used, apart from the AUCB2s, which can be negative, so these were not transformed. Analysis was by robust regression model using M estimation, Huber weighting, using the default parameter c = 1.345. The model included treatment and cohort as factors (samples from each cohort were assayed on the same day so that assay and experimental cohort were the same) and baseline (Day 1) body weight and bleeding order as covariates.

Terminal plasma glucose and insulin levels were log transformed and analysed by general linear model with treatment and cohort as factors and bleeding order and Day 1 body weight as covariates.

*Pancreatic insulin levels:* Pancreatic insulin levels were log transformed and analysed by two-way analysis of variance with treatment and cohort as factors.

*Tissue weights:* Retroperitoneal fat pads, liver and pancreas weights were analysed by two-way analysis of covariance with treatment and cohort as factors and body weight on Day 1 as the covariate. To determine effects on tissue weights, independent of changes in body weight, the fat pad, liver and pancreas weights were also analysed by two-way analysis of covariance with final carcass weight as the covariate.

Multiple comparisons to compare each treatment to the vehicle-treated control group and to compare each combination treatment to its individual constituents were by the multiple t test.

For all analysis, p<0.05 was the level accepted for statistical significance. The chance of a false positive is 5% for each compound for each time. All tests were carried out as two-sided tests.

#### RESULTS

#### General observations

All animals remained in good condition throughout the induction of obesity and, in general, remained in good condition throughout the feeding study. The different treatments were not associated with any obvious behavioural effects.

The following observations were reported during the *in vivo* phase of the feeding study. On Day -6, one of the rats (rat 35) selected for the feeding study bit through the catheter which was used for administration of castor oil/dehydrated alcohol vehicle by gavage. Part of the tubing could not be removed and therefore the rat was terminated by a Schedule 1 procedure and replaced by one of the spare animals. On Day -2, one rat (rat 47C) was observed to have pale eyes and slight piloerection prior to administration of vehicle, however, this was not observed again. On Day 12, rat 32F bled from the mouth when the dosing catheter was placed in it. No damage to the mouth was visible. The bleeding stopped and the dosing solution (RDC5 and naltrexone) was successfully administered. No ill effects were observed the following day and the food intake of the rat was not abnormal (for the rat and other animals in the same treatment group). On Day 26, red staining was noticed around 2 faecal pellets on the cagepad of rat 19A (vehicle-treated control group) and the animal had slight red staining around the anus. Food intake of this animal was low on Days 25 to 27 and Day 31 but the animal was bright and inquisitive. On Day 37, red staining was reported around the faecal pellets and anus of rat 60G. This animal had low food intake on Days 35 and 36. On Day 15, rat 82G (bupropion and naltrexone) was reported to have a small scab on the chest where the fur was thin. On Day 30, blood was noticed on the walls and feeding jars of this animal though there was no evidence of any injury.

On Day 37, mild ataxia (lasting a couple of minutes) was reported for rat 20C after the morning administration of bupropion (20 mg/kg ip). Mild salivation was reported for rat 3G (bupropion and naltrexone) prior to dosing on Day 38 and after dosing on Day 43.

On Day 4, rat 55H (sibutramine) was observed to have porphyrin staining around the nose and on Days 39 and 40, rat 51F (RDC5 and naltrexone) was reported to have porphyrin staining around the left eye.

Gross post-mortem observations were conducted on all animals at termination (with the exception of the sibutramine-treated group as tissues were not collected from these animals). The main observations were of the presence of blood in the peritoneal cavity or stomach (two of the control rats (19A and 24A) and one animal given bupropion with naltrexone (67G)); a mass and/or signs of a lesion on the liver (one of the control rats (45A), two rats given RDC5 (49B and 84B), three rats given bupropion (6C, 43C and 74C); one rat given RDC5 with bupropion (65E), two rats given RDC5 with naltrexone (51F and 52F) and two rats given bupropion with naltrexone (72G and 82G). In addition, masses were reported on the outside of the stomach (one control rat (24A) and one rat in the bupropion and naltrexone combination group (67G)) or sternum (one animal from the bupropion group (6C), one given RDC5 with bupropion (42E) and one given bupropion with naltrexone (21G)). These effects were clearly not drug-related.

#### Effects of RDC5, bupropion and naltrexone, alone and in combination, on body weight in dietary-induced obese, female Wistar rats

The effects of repeated administration of RDC5 (0.6 mg/kg po), alone and in combination with bupropion (20 mg/kg ip qd 1-25/bid 26-43) or naltrexone (1 mg/kg ip) on body weight in dietary-induced obese, female Wistar rats are shown in Tables 1 and 2 and Figures 1 and 2. These doses of bupropion and naltrexone were also given alone and in combination. Sibutramine 5 mg/kg po was used as a reference compound.

Body weights of the 8 groups of rats were similar during the 7 day baseline period. There were no significant differences between the groups of rats that subsequently received one of the 7 drug treatments and the animals allocated to the vehicle-treated control group or between rats allocated to the combination treatment groups and their individual constituents during the baseline period (Table 1 and Figure 1).

The vehicle-treated control dietary-induced obese, female Wistar rats were more or less weight-stable as expected (Figure 1), gaining on average only 16.5 g over the entire 6 week drug administration period (Overall, Day 1-43; Table 2 and Figure 2).

Chronic administration of RDC5 produced a gradual decrease in body weight in dietary-induced obese, female Wistars rats compared to the control group (Table 1 and Figure 1). This effect was mainly observed during the first 2 weeks of treatment before the body weights of the RDC5 group began to plateau. Body weights of the rats given RDC5 were significantly lower than the control group on Day 6 and then on every day of drug treatment (Table 1 and Figure 1). On Days 36 and 43 (before the overnight fast for the OGTT and at the end of the study) body weights of the rats given RDC5 were 6.0% and 4.8% lower than those of the vehicle-treated control group, respectively (Table 1 and Figure 1).

Bupropion produced small, transient decreases in body weight compared to controls on Day 5 and 6 but did not significantly alter body weight on any other day of the study. Thus, the increase in dose of bupropion to 20 mg/kg ip bid (after the body weight measurements on Day 26), had no effect on body weight compared to the vehicle-treated control group. Naltrexone produced a small, significant decrease in body weight on Day 6 only (Table 1 and Figure 1). In contrast, sibutramine produced a marked, significant decrease in body weight compared to the control group on Day 2 (ie 24 h after the first dose). Body weights of the sibutramine group remained significantly lower than controls on every day of drug treatment. The reduction in body weight produced by sibutramine occurred mainly in the first week of treatment before body weights of

the sibutramine group reached a plateau. On Days 36 and 43, body weights of the sibutramine-treated animals were 9.8% and 10.4% lower than the control group, respectively (Table 1 and Figure 1).

Body weights of rats given the combination of RDC5 and bupropion, were significantly lower than the control group on every day of the study from Day 8 onwards but were not significantly different to those of animals given RDC5 alone. Thus, the weight curves of the RDC5 with bupropion were similar to those of the animals given RDC5 alone. On Days 36 and 43, body weights of the rats given the combination of RDC5 and bupropion, were 5.5% and 6.9%, lower than the control group, respectively (compared to 6.0% and 4.8% for RDC5 alone; Table 1 and Figure 1). Body weights of the combination group were significantly lower than the bupropion group on nearly every day from Day 19 (Table 1 and Figure 1).

The combination of RDC5 and naltrexone did not significantly alter body weight compared to the vehicle-treated control group on any day of drug treatment and on Day 36 and 43, the body weights of the rats given RDC5 with naltrexone were only 3.0% and 1.8%, lower than controls, respectively (Table 1 and Figure 1). Body weights of the rats given RDC5 plus naltrexone were not significantly different to those of rats given RDC5 alone, with the exception of Day 11 when they were higher. However, these animals weighed less than the naltrexone group on Days 17, 21, 25, 27-31 and 33 of treatment (Table 1 and Figure 1).

Body weights of the obese rats given bupropion with naltrexone were not significantly different to those of vehicle-treated control group or the animals given bupropion or naltrexone alone at the doses used in this study (Table 1 and Figure 1). Increasing the dose of bupropion on Day 26 appeared to have little effect on daily body weight of the animals given the combination of bupropion and naltrexone.

The body weight results have also been expressed as change in body weight per week of treatment and over the entire 6 week drug treatment period (Table 2 and

Figure 2). Due to the change in dose of bupropion from qd to bid on Day 26, Week 4 was defined as Day 22-26 and Week 5 was defined as Day 26-36 and change in body weight was determined over Weeks 1-4 (Day 1-26) and Weeks 5-6 (Day 26-43). Finally, due to the food deprivation period for the OGTT which caused a drop in body weight in all groups of rats on Day 37 (Figure 1), change in body weight was also determined over Weeks 1-5 (Day 1-36).

The control group gained a small degree of weight during Weeks 1, 2, 3, 4 and 5, Weeks 1-4, Weeks 1-5 and overall and lost a small amount of weight following the OGTT (Week 6) and when body weight was averaged over Weeks 5-6.

RDC5 produced a small, sustained decrease in body weight during Week 1 (6.6 g) and Week 2 (6.4 g) compared to the marked weight-loss induced by sibutramine which occurred mainly during Week 1 (32.7 g). The RDC5-treated group lost

8.6 g during Weeks 1-4; 9.2 g during Weeks 1-5 and 4.4 g overall, compared to weight-loss of 28.5 g, 26.0 g and 29.2 g for the sibutramine group (Table 2). These responses were significant compared to the weight gain observed in the control group over corresponding time periods (16.6 g, 17.1 g and 16.5 g). Bupropion and naltrexone alone did not significantly alter body weight expressed as change in body weight compared to the control group, even after the dose of bupropion had been increased on Day 26 (Table 2 and Figure 2).

The combination of RDC5 plus bupropion decreased body weight during Week 1 (2.8 g) and over Weeks 1-4 (3.7 g), Weeks 1-5 (7.2 g) and overall (13.6 g). These responses were significantly different to the weight gain observed in the control group over the same periods. RDC5 plus bupropion did not significantly alter body weight expressed as change in body weight during the other intervals examined compared to the vehicle-treated controls (Table 2 and Figure 2). The changes in body weight of the RDC5 plus bupropion and RDC5 groups were not significantly different during the first 4 weeks of treatment or during Week 5 ie following the increase in dose of bupropion. However, rats given RDC5 plus bupropion lost weight after the OGTT (Week 6) compared to the small amount of weight gain in the animals given RDC5 alone (Figure 1). Accordingly, changes in

body weight between the combination group and RDC5 alone were significantly different when body weight was measured over Week 6 and Weeks 5-6 (Table 2 and Figure 2). These changes were relatively small and the weight-loss produced by RDC5 plus bupropion was comparable to that produced by RDC5 alone overall (Day 1-43; 13.6 g vs 4.4 g, respectively) as shown in Table 2 and Figure

2. The decreases in body weight observed in animals given RDC5 plus bupropion were significantly different to the small increases in body weight observed in animals given bupropion alone (Week 2, Weeks 1-4, Weeks 1-5 and overall).

The combination of RDC5 plus naltrexone did not significantly modify body weight expressed as change in body weight during any time period examined compared to the control group or the animals given naltrexone alone. During Week 2, the rats given RDC5 plus naltrexone gained weight compared to the weight-loss observed in the RDC5 alone group. This difference was significant (Table 2 and Figure 2).

Animals given the combination of bupropion and naltrexone gained a small degree of weight during Week 1, 2, 3 and 4, when bupropion was administered once a day. These changes in body weight were comparable to those seen in the control group. Increasing the dosing frequency of bupropion produced weight- loss in the animals given bupropion plus naltrexone during Week 5 and Weeks 5-6 though the combination of these two drugs did not significantly alter change in body weight after the OGTT (Week 6) or measured over Weeks 1-4, Weeks 1-5 or overall compared to the controls (Table 2 and Figure 2). There were no significant differences in changes in body weight during any time-bin between animals given the combination of bupropion plus naltrexone or rats given bupropion or naltrexone alone (Table 2 and Figure 2).

#### Effects of RDC5, bupropion and naltrexone, alone and in combination, on food intake in dietary-induced obese, female Wistar rats

The effects of repeated administration of RDC5 (0.6 mg/kg po), alone and in combination with bupropion (20 mg/kg ip qd 1-25/bid 26-43) or naltrexone

(1 mg/kg ip) on food intake in dietary-induced obese, female Wistar rats are shown in Tables 3 to 5 and Figures 3 to 5. Bupropion and naltrexone were also given alone and in combination and sibutramine 5 mg/kg po was used as a reference compound.

Daily food intakes of the 8 groups of rats were similar during the 7 day baseline period. There were no significant differences between the groups of rats that subsequently received one of the 7 drug treatments and the animals allocated to the vehicle-treated control group or between rats allocated to the combination treatment groups and their individual constituents during the baseline period with the exception of between the two groups that subsequently received RDC5 plus bupropion and bupropion alone on Day -3 (Table 3 and Figure 3). This small difference is unlikely to have been meaningful and would have been taken into account by the statistical analysis as it adjusts for differences in daily food intake averaged over the baseline period.

Repeated administration of RDC5, bupropion and naltrexone alone had relatively little effect on daily food intake during the 42 day drug treatment period. Animals given RDC5 ate significantly less than the vehicle-treated control group on Days 6 and 9 whereas bupropion significantly increased food intake on Days 12, 19 and

38 and naltrexone significantly increased food intake on Days 11 and 16 compared to the control group. Increasing the dosing frequency of bupropion on Day 26 had little effect on daily food intake compared to the controls. In comparison, sibutramine significantly reduced food intake by 86.4% in the first 24 h following administration. The food intake of the sibutramine group remained significantly lower than controls on Days 2 to 9, though the magnitude of the hypophagic response to sibutramine slowly decreased on repeated administration of the drug. From Day 10, the food intake of the sibutramine group was not significantly different from that of the control group (Table 3 and Figure 3).

Daily food intake of animals given RDC5 plus bupropion was significantly increased on Day 34 and significantly reduced on Day 41, compared to the control group. In general, however, this combination of drugs did not alter food intake.

Daily food intakes of the RDC5 plus bupropion group were significantly lower than the RDC5 group on Days 40 and 41 and the bupropion group on Days 11, 12, 15, 18, 19 and 41 (Table 3 and Figure 3).

Daily food intake of rats given RDC5 plus naltrexone was significantly higher than controls on Days 22, 31, 37 and 38 but not altered on any other day of treatment. These animals ate more than the RDC5 group on Days 14, 27, 31 and 32 and the naltrexone group on Day 39 but less than the naltrexone group on Days 11 and 12 (Table 3 and Figure 3).

Animals given the combination of bupropion and naltrexone ate significantly more than controls on Days 8, 10, 12, 16 and 17. On Day 17, these animals also ate more than rats given bupropion or naltrexone alone (Table 3 and Figure 3). The combination of bupropion and naltrexone did not significantly alter daily food intake following the increase in dose of bupropion on Day 26.

The food intake results have also been expressed in terms of average daily food intake per week (when Week 4 and Week 5 were defined as Days 22-25 and 26-35, respectively, due to the change in dose of bupropion on Day 26) and also over Weeks 1-4 (Days 1-25), Weeks 5-6 (Days 26-42); Weeks 1-5 (Days 1-35; ie up to the overnight food deprivation on Day 36 before the OGTT on Day 37) and overall (Days 1-42) (Table 4 and Figure 4).

RDC5 significantly reduced average daily food intake during Week 1 by 11.6% and significantly increased average daily food intake by 11.7% during Week 6 but did not significantly alter this parameter, compared to the control group, during any of the other time periods examined (Table 4 and Figure 4).

Bupropion significantly increased average daily food intake during Week 4, by 14.0% compared to the control group, but did not alter average daily food intake during any other week or average daily food intake during Weeks 1-4, Weeks 5-6, Weeks 1-5 or overall. Repeated administration of naltrexone alone had no effect on average daily food intake during any time period (Table 4 and Figure 4).

Sibutramine significantly reduced average daily food intake during Week 1, by 47.8% compared to the control group, but did not significantly alter average daily food intake during Weeks 2, 3, 4, 5 and 6 or Weeks 5-6. Sibutramine significantly

reduced average daily food intake by 17.6% (Weeks 1-4); 12.9% (Weeks 1-5) and 11.7% (overall) compared to the vehicle-treated controls. These decreases in average daily food intake were primarily due to the marked hypophagic effects of sibutramine in the first week of administration (Table 4 and Figure 4).

The combination of RDC5 with bupropion did not significantly modify average daily food intake during drug treatment. Average daily food intakes of rats in the combination treatment group were significantly lower than those of animals given RDC5 alone during Week 6 and bupropion alone during Week 2, 3 and Weeks 1-4 (Table 4 and Figure 4). However, daily food intakes of rats given the combination of RDC5 and bupropion were not significantly different to those of rats given either RDC5 or bupropion alone when averaged over Weeks 1-5 or overall.

The combination of RDC5 and naltrexone significantly increased average daily food intake by 15.3% compared with the vehicle-treated controls during Week 6 and by 12.9% when daily food intake was averaged over Weeks 5-6, but did not significantly alter this parameter during Weeks 1, 2, 3, 4 and 5, Weeks 1-4, Weeks 1-5 or overall (Table 4 and Figure 4). Average daily food intakes of rats given the combination of RDC5 with naltrexone were not significantly different to those of animals given RDC5 or naltrexone alone during any time period (Table 4 and Figure 4).

Repeated administration of bupropion with naltrexone, significantly increased average daily food intake during Week 2 and Week 3 by 17.2% and 12.0%, respectively and significantly increased average daily food intake measured over Weeks 1-4 by 10.0%, compared to the control group. However, the average daily food intake of rats given this combination of drugs was not significantly different to controls during any other time period. Thus, the increases in average daily food intake induced by the combination of bupropion and naltrexone occurred

before the dose of bupropion was increased on Day 26. The average daily food intake of rats given the combination of bupropion and naltrexone was not significantly different to that of animals given bupropion or naltrexone alone (Table 4 and Figure 4).

Finally, the food intake data have been expressed as cumulative food intake on each day of treatment (Table 5 and Figure 5). RDC5 significantly decreased cumulative food intake on Days 6 to 15 and on Day 18 compared to the control group. Bupropion and naltrexone alone had no effect on this parameter whereas sibutramine decreased food intake during Day 1 (as mentioned above) and cumulative food intake on every day from Day 2 to 42. Overall, sibutramine significantly reduced food intake by 12.8% during the study compared to the control group (cumulative food intake on Day 42; Table 5 and Figure 5).

The combination of RDC5 and bupropion did not significantly alter cumulative food intake per day compared to controls. Cumulative food intake of this group was significantly lower than the bupropion group on Day 3, 4, 9, 11 to 32, 41 and 42 but was not significantly different to the RDC5 group during drug treatment. Cumulative food intake of the animals given the combination of RDC5 and naltrexone was not significantly different to control levels or that of the animals given RDC5 or naltrexone alone on any day (Table 5 and Figure 5).

Cumulative food intake of rats given the combination of bupropion and naltrexone was significantly increased compared to the control group on Day 17 to 24 but was not significantly different to either bupropion or naltrexone alone on any day of treatment (Table 5 and Figure 5).

#### Effects of RDC5, bupropion and naltrexone, alone and in combination, on water intake in dietary-induced obese, female Wistar rats

The effects of repeated administration of RDC5 (0.6 mg/kg po), alone and in combination with bupropion (20 mg/kg ip qd 1-25/bid 26-43) or naltrexone (1 mg/kg ip) on water intake in dietary-induced obese, female Wistar rats are

shown in Tables 6 and 7 and Figures 6 and 7. Bupropion and naltrexone were also given alone and in combination and sibutramine 5 mg/kg po was used as a reference compound.

Daily water intakes of the 8 groups of animals used in the study were similar during the 7 day baseline period. Significant differences between the daily water intakes of the different groups of rats were observed on Days -6, -3 and -1, however, these differences were only small and would have been accounted for by the statistical analysis as it adjusts for differences in daily water intake averaged over the baseline period (Table 6 and Figure 6).

RDC5 significantly decreased daily water intake on Days 3, 5, 6, 9, 13, 14 and 27 compared to the control group. Bupropion significantly increased daily water intake over control levels on Days 2 and 5 and naltrexone significantly decreased daily water intake on Days 9, 11, 14, 24, 29, 31, 35 and 36. In comparison, sibutramine significantly decreased daily water intake on Days 1 and 2 (by 56.5% and 22.8%, respectively) and significantly increased daily water intake on Days 17, 20, 21, 24, 25, 30, 32, 37 and 41, compared to the control group.

The combination of RDC5 and bupropion significantly decreased daily water intake on Days 8, 9, 11 and 14 compared to the control group. Daily water intakes of these rats were not significantly different to those of animals given RDC5 alone on any day of treatment but were significantly lower than the bupropion group on Days 2, 5, 7 to 9, 11, 15, 16, 18 to 20 and 30 (Table 6 and Figure 6).

Repeated administration of RDC5 with naltrexone significantly decreased daily water intake on Days 1, 2, 11, 14 and 36 compared to control levels. Water intakes of the rats given the combination of RDC5 and naltrexone were not significantly different to those of animals given RDC5 alone on any day of treatment but were significantly lower than the naltrexone group on Days 1 and 2 and significantly higher on Day 30 and 35 (Table 6 and Figure 6).

The combination of bupropion and naltrexone had no effect on daily water intake except for a transient increase over control levels on Day 37. The daily water intake of the bupropion with naltrexone group was significantly lower than the bupropion group on Days 5 and 7 and significantly higher than the naltrexone group on days 14, 29, 36 to 38 (Table 6 and Figure 6).

Increasing the dose of bupropion on Day 26 appeared to have little effect on water intake regardless of whether the drug was given alone or in combination with RDC5 or naltrexone (Table 6 and Figure 6)

The water intake results have also been expressed in terms of average daily water intake per week (when Week 4 and Week 5 were defined as Days 22-25 and 26-35, respectively, due to the change in dose of bupropion on Day 26) and also over Weeks 1-4 (Days 1-25), Weeks 5-6 (Days 26-42); Weeks 1-5 (Days 1-35; ie up to the overnight food deprivation on Day 36 before the OGTT on Day 37) and overall (Days 1-42) (Table 7 and Figure 7).

RDC5 significantly decreased average daily water intake in the dietary-induce obese rats during Week 1 and Week 2, by 17.1% and 14.6%, respectively, compared to the control group, but did not alter this parameter during Weeks 3, 4, 5 and 6 or when daily water intake was averaged over Weeks 1-4, Weeks 5-6, Weeks 1-5 or overall. Bupropion did not significantly modify average daily water intake during any of the time periods examined. However, naltrexone significantly reduced average daily water intake during Week 2 (16.7%), Week 5 (18.9%).

Weeks 5-6 (15.7%), Weeks 1-5 (12.5%) and overall (12.6%), compared to the control group. In comparison, sibutramine, significantly increased average daily water intake during Week 3 and 4 and overall, by 20.2%, 21.9% and 12.4%, respectively, compared to the vehicle-treated controls (Table 7 and Figure 7).

Average daily water intake was significantly reduced by the combination of RDC5 and bupropion during Week 2 (17.5%) and Weeks 1-4 (13.0%), compared to the control group, whereas the combination of RDC5 and naltrexone significantly reduced average daily water intake during Week 1 (13.1%) but not

during any other time period (Table 7 and Figure 7). The average daily water intake of rats given RDC5 plus bupropion was similar to RDC5 alone throughout drug treatment but was significantly lower than bupropion alone during Week 1, 2 and 3 and when water intake was averaged over Weeks 1-4, Weeks 1-5 and overall as shown in Table 7 and Figure 7. The average daily water intake of rats given the combination of RDC5 and naltrexone was not significantly different to RDC5 alone during any time period but was significantly higher than naltrexone alone during Week 5 and Weeks 5-6 (Table 7 and Figure 7).

The average daily water intakes of rats given the combination of bupropion and naltrexone were not significantly different to those of the control group or animals given bupropion alone during treatment but were significantly higher than those of the naltrexone group during Week 6 and Weeks 5-6 (Table 7 and Figure 7).

Increasing the dose of bupropion on Day 26 had little effect on average daily water intake when bupropion was given alone or in combination with RDC5 or naltrexone (Table 7 and Figure 7).

#### Effects of RDC5, bupropion and naltrexone, alone and in combination, on glucose tolerance in dietary-induced obese, female Wistar rats - Day 37 OGTT

An OGTT was used to assess the effects of the various treatments on glycaemic control in dietary-induced obese, female Wistar rats. The animals were fasted overnight on Day 36 and the test was performed on Day 37. The effects of repeated administration of RDC5 (0.6 mg/kg po), alone and in combination with bupropion (20 mg/kg ip qd 1-25/bid 26-43) or naltrexone (1 mg/kg ip) on plasma glucose and insulin levels, before and following the glucose challenge (2 g/kg po), are shown in Tables 8 and 9 and Figures 8 to 12. Bupropion and naltrexone were also given alone and in combination and sibutramine 5 mg/kg po was used as a reference compound.

Fasted plasma glucose levels of the vehicle-treated control group were 6.38 mM at the B1 baseline time-point and 5.81 mM at B2 (Table 8). Fasted plasma insulin levels were 0.83 and 0.62 ng/ml, respectively, at B1 and B2 (Table 9). Plasma glucose and insulin levels of the control animals were markedly increased following the glucose challenge as expected (Tables 8 and 9 and Figure 8).

Plasma glucose levels of animals treated with RDC5, bupropion alone, naltrexone alone, RDC5 with bupropion or RDC5 with naltrexone were not significantly different to the control group at B1 or B2. Plasma glucose levels of the animals given the combination of bupropion and naltrexone, were significantly higher than controls and the naltrexone group at B2, ie 1 h after drug treatment but were not significantly different at the B1 time-point. Plasma glucose levels of the sibutramine group were significantly lower than controls 1 h after drug administration but were not significantly different at B1, ie approximately 24 h after drug treatment on Day 36 (Table 8 and Figure 8).

RDC5 did not significantly alter plasma glucose levels at any time-point after the glucose challenge except for a significant increase in plasma glucose compared to the control group at 120 min. Bupropion and naltrexone had no effect on plasma glucose levels after glucose loading (Table 8 and Figure 8). Furthermore, RDC5, bupropion and naltrexone did not significantly alter plasma glucose expressed as AUC (0-60 min), AUC (0-120 min), AUCB2 (0-60 min) or AUCB2 (0-120 min)

compared to the control group (Table 8 and Figures 9 to 12). In comparison, plasma glucose levels of the sibutramine group were significantly lower than controls 45 and 60 min post-glucose (Table 8 and Figure 8). Sibutramine significantly reduced plasma glucose AUC (0-60 min) and AUC (0-120 min) compared to controls but did not significantly alter plasma glucose AUCB2 (0-60 min) or AUCB2 (0-120 min) (Table 8 and Figures 9 to 12).

Plasma glucose levels of the rats given RDC5 with bupropion were significantly lower than controls and bupropion alone at 20 min post-glucose and RDC5 alone at 45, 60 and 120 min post-glucose (Table 8 and Figure 8). The combination of RDC5 and bupropion did not significantly alter plasma glucose AUC (0-60 min),

AUC (0-120 min), AUCB2 (0-60 min) or AUCB2 (0-120 min) compared to controls. However, plasma glucose AUC (0-60 min) was significantly reduced compared to bupropion alone and plasma glucose AUC (0-120 min) and AUCB2 (0-120 min) were significantly reduced compared to RDC5 alone (Table 8 and Figures 9 to 12).

The combination of RDC5 and naltrexone significantly reduced plasma glucose levels compared to controls at 20 min and compared to RDC5 alone at 45 and 60 min post-glucose but significantly increased plasma glucose compared to the control and naltrexone groups 120 min post-glucose (Table 8 and Figure 8). The combination of RDC5 and naltrexone did not significantly modify plasma glucose AUC (0-60 min), AUC (0-120 min), AUCB2 (0-60 min) or AUCB2 (0-120 min)

compared to the groups given vehicle, bupropion or naltrexone alone (Table 8 and Figures 9 to 12).

The combination of bupropion and naltrexone did not significantly modify plasma glucose compared to the control, bupropion or naltrexone groups at any time- point following the glucose load. The combination of these two drugs did not significantly alter plasma glucose AUC (0-60 min), AUC (0-120 min), AUCB2 (0-60 min) or AUCB2 (0-120 min) compared to the vehicle-treated controls, though plasma glucose AUCB2 (0-60 min) and AUCB2 (0-120 min) were significantly lower in the animals given bupropion with naltrexone compared to bupropion alone (Table 8 and Figures 9 to 12).

RDC5 significantly reduced plasma insulin at B1, but not at B2, compared to the control group. Plasma insulin levels of the other drug treatment groups were not significantly different from controls at B1 or at B2, ie one hour following drug administration on Day 37 (Table 9 and Figure 8).

RDC5 significantly reduced plasma insulin levels 10, 45 and 60 min following the glucose load and also significantly reduced plasma insulin AUC (0-60 min), AUC (0-120 min), AUCB2 (0-60 min) and AUCB2 (0-120 min) compared to the control group. Bupropion and naltrexone had no effect on plasma insulin levels at

any time point post-glucose and did not significantly alter plasma insulin AUC (0-60 min), AUC (0-120 min), AUCB2 (0-60 min) and AUCB2 (0-120 min)

compared to the control group. In comparision, plasma insulin levels of the sibutramine group were significantly lower than controls at 10, 30, 45 and 60 min after glucose loading. Sibutramine also significantly reduced plasma insulin AUC (0-60 min), AUC (0-120 min), AUCB2 (0-60 min) and AUCB2 (0-120 min). The

reductions in plasma insulin produced by RDC5 and sibutramine following the glucose challenge were comparable in magnitude (Table 9 and Figures 8 to 12).

Plasma insulin levels of rats in the RDC5 and bupropion combination group were significantly decreased compared to the vehicle-treated controls at 10, 20, 30, 45 and 60 min after the glucose challenge and significantly lower than bupropion alone at 10, 20 and 30 min post-glucose but were not significantly different to those of animals given RDC5 alone (Table 9 and Figure 8). The combination of RDC5 with bupropion significantly decreased plasma insulin AUC (0-60 min), AUC (0-120 min), AUCB2 (0-60 min) and AUCB2 (0-120 min) compared to the control group. The magnitude of these responses was comparable to those produced by RDC5 alone. Thus, plasma insulin AUC (0-60 min), AUC (0- 120 min), AUCB2 (0-60 min) and AUCB2 (0-120 min) values of animals given RDC5 and bupropion were not significantly different to those of the RDC5 group but were significantly reduced compared to bupropion alone (Table 9 and Figures 9 to 12).

Plasma insulin levels of the animals given RDC5 with naltrexone were significantly lower than controls at 10, 45 and 60 min post-glucose and were significantly lower than naltrexone at the 60 min time-point but were not different to the RDC5 group at any time-point following the glucose load (Table 9 and Figure 8). The combination of RDC5 with naltrexone significantly reduced plasma insulin AUC (0-60 min), AUC (0-120 min), AUCB2 (0-60 min) and AUCB2 (0-120 min) compared to the control group. These parameters were not significantly different from those of animals given RDC5 or naltrexone alone (Table 9 and Figures 9 to 12).

The combination of bupropion and naltrexone did not significantly alter plasma insulin levels at any time-point following the glucose load or plasma insulin AUC (0-60 min), AUC (0-120 min), AUCB2 (0-60 min) or AUCB2 (0-120 min)

compared to the control group or the groups of animals given bupropion or naltrexone alone (Table 9 and Figures 8 to 12).

#### Effects of RDC5, bupropion and naltrexone, alone and in combination, on terminal plasma glucose and insulin levels in dietary-induced obese, female Wistar rats

The effects of repeated administration of RDC5 (0.6 mg/kg po), alone and in combination with bupropion (20 mg/kg ip qd 1-25/bid 26-43) or naltrexone (1 mg/kg ip) on terminal plasma glucose and insulin levels in dietary-induced obese, female Wistar rats are shown in Table 10 and Figure 13. Bupropion and naltrexone were also given alone and in combination and sibutramine 5 mg/kg po was used as a reference compound. Animals had free access to food before termination, 3 h after dosing (0 h) on Day 43.

Plasma glucose and insulin levels of the vehicle–treated control group were

13.75 mM and 6.31 ng/ml, respectively (Table 10 and Figure 13). These values were much higher than the plasma glucose and insulin levels reported following an overnight fast (B1 of the OGTT) as expected. Plasma glucose levels were not significantly altered by any of the drug treatments compared to the control group. Plasma glucose levels of fed animals given bupropion with naltrexone were significantly higher than those of the bupropion group at termination. No other differences in terminal plasma glucose levels were observed between animals given combinations of two drugs and their individual constituents (Table 10 and Figure 13).

Plasma insulin levels in the fed obese rats were significantly reduced in the RDC5 (34.4%), naltrexone (44.7%), RDC5 with naltrexone (39.2%) and bupropion with naltrexone (43.0%) treatment groups but not by bupropion, RDC5 with bupropion or sibutramine. Terminal plasma insulin levels of the combination groups were

not significantly different to those of their individual constituents (Table 10 and Figure 13).

#### Effects of RDC5, bupropion and naltrexone, alone and in combination, on pancreatic insulin levels in dietary-induced obese, female Wistar rats

The effects of repeated administration of RDC5 (0.6 mg/kg po), alone and in combination with bupropion (20 mg/kg ip qd 1-25/bid 26-43) or naltrexone (1 mg/kg ip) on total pancreatic insulin levels in dietary-induced obese, female Wistar rats are shown in Table 11 and Figure 14. Bupropion and naltrexone were also given alone and in combination. Animals were terminated 3 h after dosing (0 h) on Day 43.

RDC5, RDC5 with bupropion and RDC5 with naltrexone significantly decreased total pancreatic insulin levels, by 44.8%, 46.2% and 55.4%, respectively, compared to the control group (Table 11 and Figure 14). Bupropion and naltrexone, alone or in combination, had no effect on this parameter. Pancreatic insulin levels in the RDC with bupropion and RDC5 with naltrexone combination groups were not significantly different to RDC5 alone but were significantly lower than those of animals given bupropion or naltrexone alone, respectively (Table 11 and Figure 14).

#### Effects of RDC5, bupropion and naltrexone, alone and in combination, on fat pad, liver and pancreas weights in dietary-induced obese, female Wistar rats

The effects of repeated administration of RDC5 (0.6 mg/kg po), alone and in combination with bupropion (20 mg/kg ip qd 1-25/bid 26-43) or naltrexone (1 mg/kg ip) on retroperitoneal fat pad, liver and pancreas weights in dietary- induced obese, female Wistar rats are shown in Tables 12 and 13 and Figures 15 and 16. Bupropion and naltrexone were given alone and in combination. Tissue weights have been adjusted for body weights at baseline (Day 1) and at termination (final carcass weights).

RDC5, bupropion, naltrexone, RDC5 with bupropion, RDC5 with naltrexone and bupropion with naltrexone did not significantly alter retroperitoneal fat pad, liver or pancreas weights compared to the control group when results were adjusted for differences in body weight before the start of drug treatment on Day 1 (Table 12 and Figure 15). Fat pad, liver and pancreas weights of rats given the combinations of RDC5 with bupropion, RDC5 with naltrexone or bupropion with naltrexone were not significantly different to those of their individual constituents given alone.

RDC5, bupropion, naltrexone, RDC5 with bupropion, RDC5 with naltrexone and bupropion with naltrexone did not significantly alter retroperitoneal fat pad or pancreas weights compared to controls when results were adjusted for differences in final carcass weight (Table 13 and Figure 16). Fat pad and pancreas weights of rats given the combinations of RDC5 with bupropion, RDC5 with naltrexone or bupropion with naltrexone were not significantly different to those of rats given their individual constituents alone.

RDC5 and naltrexone did not significantly alter liver weights when given alone or in combination, however, liver weights of the bupropion, RDC5 with bupropion and bupropion with naltrexone groups were significantly increased by 10.9%, 16.1% 10.5%, respectively, compared to the control group, when final carcass weight was used as the covariate in the analysis. Thus, the liver weights of the animals given bupropion, alone or in combination with RDC5 or naltrexone, were significantly higher than expected given the effect of these drug treatments on final carcass weight. Liver weights of animals given the combination of RDC5 and bupropion were not significantly different to those of rats given RDC5 or bupropion alone. The livers of rats given the combination of bupropion and naltrexone were similar in weight to those of animals given bupropion alone but weighed significantly more than the naltrexone group, when adjusted for final carcass weight (Table 13 and Figure 16).

#### DISCUSSION

One of the major finding of this study is that repeated administration of RDC5 (Prograf®, tacrolimus), a prophylactic immunosuppressant agent for organ transplantation, significantly decreased body weight in dietary-induced obese, female Wistar rats maintained on a simplified cafeteria diet. After 42 days of drug administration the body weights of the rats given RDC5 (0.6 mg/kg po) were 4.8% lower than the vehicle-treated controls. In comparison, the 5-HT and noradrenaline reuptake inhibitor, sibutramine (5 mg/kg po), which has until recently been used in the management of obesity in the clinic, decreased body weight by 10.4% compared to the control group. On Day 36, ie immediately before the overnight fast for the OGTT, these values were 6.0% and 9.8%, for RDC5 and sibutramine, respectively. Thus, RDC5 (0.6 mg/kg po) produced approximately half the decrease in body weight observed with sibutramine (5 mg/kg po). These findings are consistent with the magnitude of the decreases in body weight produced by RDC5 (1 mg/kg po) and sibutramine (7.5 mg/kg po) compared to the control group in male rats on high-fat diet (6.6% and 12.4%, respectively, following 40 days of drug administration)^17^ and also with a previous report that RDC5 reduces body weight in obese Zucker rats^20^.

It is clear from the daily body weight curve and the change in body weight data that different mechanisms underlie the marked decrease in body weight produced by sibutramine, which occurred mainly in the first week of treatment, and the more gradual reduction in body weight produced by RDC5. This emerged on Day 6 and occurred mainly during the first two weeks of the study before body weights of the RDC5-treated animals began to plateau, though remaining significantly lower than controls throughout treatment.

The body weights of animals given RDC5 with bupropion (20 mg/kg ip qd on Days 1-25; bid from Day 26) corresponded closely to those of animals given RDC5 alone. Small differences were observed, as the reduction in body weight for the RDC5 and bupropion combination did not become significant compared to controls until Day 8 (versus Day 6 for RDC5 alone) and some divergence

between the body weight curves of the two groups of animals was observed during the week after the OGTT (Week 6) as shown by the change in body weight data. Thus, the RDC5 group gained a little weight compared to the small degree of weight-loss observed in animals given the combination of RDC5 and bupropion. However, both treatments produced a similar decrease in body weight compared to the control group on Day 36 (6.0% for RDC5 and 5.5% for RDC5 with bupropion) and Day 43 (4.8% and 6.9%, respectively) and body weights of the two groups of rats were not significantly different on any day of treatment. In comparison, animals given the combination of RDC5 and bupropion weighed significantly less than the bupropion group on nearly every day from Day 19, which was not surprising, as bupropion had little effect on body weight in the obese rats when given alone. Thus, the decrease in body weight observed in the animals given RDC5 with bupropion appeared to be largely due to RDC5.

Body weights of animals given both RDC5 and naltrexone (1 mg/kg ip) were not significantly different to the control group and by the end of the study, were only 3.0% (Day 36) and 1.8% (Day 43), lower than controls. It could be argued that naltrexone, that had no effect on body weight alone, was attenuating the decrease in body weight produced by RDC5 in the obese animals. Indeed, during Week 2, the rats given RDC5 plus naltrexone gained weight compared to the weight-loss observed in the RDC5 alone group, a difference that was significant. However, in general, the body weights of rats given the combination of RDC5 and naltrexone were not significantly different to RDC5 alone.

To summarise, there was no evidence from this study, that either bupropion or naltrexone, could potentiate the modest decrease in body weight produced by a moderate dose of RDC5 (0.6 mg/kg po) in dietary-induced obese rats.

The combination of bupropion and naltrexone did not significantly alter body weight during the first 4 weeks of the study. A small degree of weight-loss was observed during Week 5 (Day 26-36) ie following the increase in dose of bupropion; however, body weights of the animals given bupropion with naltrexone were not significantly lower than controls on Day 36 or 43. This was a

little surprising as a wide range of anti-obesity agents and drug candidates have been tested in dietary-induced obese, female Wistar rats (eg sibutramine, rimonabant, orlistat, topiramate, phentermine and the combination of topiramate and phentermine (Qnexa)) and found to produce a magnitude of weight-loss that correlates closely with that observed in man^18^. However, the weight-loss produced by bupropion and naltrexone in man is only moderate and it is possible, that if bupropion had been given bid throughout the study, an overall reduction in body weight may have been achieved.

Body weight is a balance between energy input and energy output. The marked reduction in body weight produced by sibutramine in the obese rats would appear to be largely due to its inhibitory effects on food intake as both of these responses occurred mainly during the first week of treatment. Indeed, sibutramine reduced food intake by 86.4%, compared to the control group, in the first 24 h following administration on Day 1, though the magnitude of this hypophagic response rapidly decreased on repeated drug administration. In this respect, the reference compound, sibutramine, produced similar effects on both body weight and food intake to those reported in other studies in the same animal model of obesity^21.22^. Overall, sibutramine significantly decreased food intake by 12.8% (cumulative food intake on Day 42) versus a reduction in body weight of 10.4%.

In contrast, RDC5 produced only small reductions in food intake compared to the control group. These effects occurred mainly during the first two weeks of treatment (decreased daily food intake on Days 6 and 9, average daily food intake during Week 1 and cumulative food intake on Days 6 to 15 and 18) and, in time course, corresponded to the gradual reduction in body weight produced by RDC5 before the body weights of the drug-treated animals began to plateau. However, RDC5 significantly increased average daily food intake compared to the controls following the OGTT (Week 6) so that overall RDC5 did not significantly reduce food intake. Thus, although reduced food intake is likely to have contributed to the initial weight-loss produced by RDC5 compared to the control group, it cannot be precluded that other factors may have played a role in its overall effects on body weight. In support of this argument, the combination of RDC5 and

bupropion produced equivalent reductions in body weight to RDC5 but had little apparent effect on food intake. Further studies could look at this more closely by examining the effects of RDC5, alone and in combination with bupropion, on energy expenditure.

The significant increases in food intake produced by bupropion alone (average daily food intake during Week 4), and, particularly when given in combination with naltrexone (Weeks 2 and 3, 1-4 and cumulative food intake on Days 17 to 24), were unexpected. In the clinic, the combination of bupropion and naltrexone, has been reported to reduce cravings for food^23^. Bupropion is a noradrenaline and dopamine reuptake inhibitor and naltrexone is an opioid antagonist, as mentioned in the Introduction. Bupropion stimulates secretion of α-MSH from pro- opiomelanocortin (POMC) cells which would be expected to decrease food intake. However, these effects are countered by the melanocortin system’s negative feedback mechanism that increases the release of endogenous opioids to inhibit α-MSH secretion. Endogenous opioids would be expected to increase food intake. It has been postulated that blockade of the µ-opioid receptor with naltrexone, to counteract the auto-inhibitory actions of bupropion-stimulated release of endogenous opioids, underlies the antiobesity action of the bupropion and naltrexone combination^24^. It is clear that bupropion has complex effects on brain areas involved in appetite and reward^24^ and its prevailing effects on food intake are likely to be dependent on the exact doses and also the model employed. In the current study in dietary-induced obese rats with free access to a calorie- dense, high-fat palatable diet, increases in food intake were observed when bupropion (20 mg/kg ip) was given once a day (alone or with naltrexone) but were less apparent after the dose of bupropion had been increased on Day 26.

The significant increases in average food intake observed in the RDC5 and naltrexone combination group when food intake was averaged over Weeks 5-6, appear to be largely due to increased food intake during Week 6 ie following the disruption caused by the OGTT. RDC5 also increased food intake during this period though naltrexone had no effect on food intake during the study. Overall, the combination of RDC5 with naltrexone did not alter food intake compared to

the controls or its individual constituents alone and there was no indication from this study that naltrexone could potentiate the small inhibitory effects of RDC5 on food intake, consistent with the body weight data.

Rats are prandial drinkers and the significant reductions in daily water intake and average daily water intake produced by RDC5 during the first two weeks of administration may simply be because the rats were eating less than the controls during this period (though the reductions in food intake were not always significant). This presumably also explains the marked decrease in water intake in the sibutramine group during the first two days of treatment. Overall, however, RDC5 had little effect on water intake, consistent with previous findings in male rats on high-fat diet^17^. In comparison, sibutramine significantly increased water intake during Weeks 3 and 4 and overall. The mechanisms underlying this response are not known, but it has been observed before (data on file; RenaSci Ltd).

The significant decrease in water intake produced by the combination of RDC5 with bupropion during Week 2 could be attributed to RDC5 as bupropion had little effect on drinking behaviour by itself throughout the study (ie before and folllowing the increase in dose on Day 26). RDC5 could also have been largely responsible for the decrease in average water intake during Week 1 of animals given RDC5 with naltrexone as the reductions in daily water intake produced by the opioid antagonist alone did not become significant until Day 9. Naltrexone significantly reduced average daily water intake during Weeks 2 and 5 and overall when given alone, but not when combined with bupropion. The decreases in drinking behaviour produced by naltrexone presumably reflect its pharmacological activity. These effects were not associated with decreased food intake.

Anti-obesity agents would be expected to decrease body weight by specifically reducing body fat rather than by producing non-specific decreases in water (dehydration) and/or protein content (muscle wasting or cachexia). Accordingly, we have previously shown that the majority of the weight-loss produced by

sibutramine can be attributed to loss of body fat^21,22^. In the current study, RDC5 and the combination of RDC5 with bupropion did not significantly reduce retroperitoneal fat pad weight, regardless of whether the results were adjusted for body weights on Day 1 or final carcass weights to indicate if there were any changes over and above those that would be expected from the change in body weight. This was probably because the degree of weight-loss achieved by both treatments was relatively small. For this reason, even a more sensitive method of examining the effects of drugs on body composition, such as chemical analysis of the carcasses to determine fat, water, protein and ash content, would be of limited value. On the other hand, all of the drug treatments used in the study were well- tolerated and the drugs, alone or in combination, did not produce any overt, persistent behavioural or physiological effects which could have influenced food intake in a non-specific manner. Furthermore, the abnormalities seen at post- mortem (blood in the peritoneal cavity or stomach, a mass or signs of a lesion in the liver or masses on the outside of the stomach or sternum) were not drug- related.

The fact that plasma glucose and insulin levels of the control group were much lower at the baseline timepoints of the OGTT than on Day 43 were not unexpected as the animals were fasted overnight before the OGTT but had free access to food before termination. Plasma glucose levels of the seven drug treatment groups were not significantly different to controls at B1 ie before drug treatment on the day of the OGTT. However, plasma insulin levels of the RDC5 group were significantly reduced compared to controls at this time-point. Plasma glucose levels were significantly increased by the combination of bupropion and naltrexone and decreased by sibutramine one hour after drug administration. The magnitude of these changes was only small.

The main findings following the glucose challenge were that RDC5, alone or in combination with either bupropion or naltrexone, significantly decreased plasma insulin compared to the control group while having little effect on plasma glucose. This is shown most clearly by the AUC and AUCB2 data ie the area under the curve and the area under the curve above the post-treatment baseline. The

reductions in plasma insulin levels of animals given the combinations of RDC5 with bupropion or naltrexone would appear to be solely due to RDC5 as they were comparable in magnitude to RDC5 alone. Furthermore, bupropion and naltrexone, either alone or in combination, did not significantly decrease plasma insulin levels following glucose loading compared to the controls.

In comparison, sibutramine significantly decreased plasma glucose after the glucose load, though not when only the AUC above baseline was considered, and produced similar reductions in plasma insulin AUC and AUCB2 to RDC5. The mechansism underlying these responses are likely to be different as RDC5, alone and in combination with bupropion or naltrexone, also significantly reduced pancreatic insulin levels by approximately 50%. This adverse response is not desirable in obese patients who are often also diabetic. In contrast, the most plausible explantion for the reduction in plasma insulin levels in the sibutramine group is that it was due to weight-loss. Decreases in body weight have been associated with improvements in insulin sensivity in both rodents and man^25,26^. In this context, although RDC5 and RDC5 combined with bupropion reduced body weight, the degree of weight-loss was only half that of sibutramine. In addition, the combination of RDC5 with naltrexone did not significantly reduce body weight compared to the controls but produced similar reductions in plasma insulin AUC and AUCB2 and in pancreatic insulin content to RDC5 alone.

Reductions in pancreatic insulin content following administration of RDC5 (tacrolimus) have been observed before in rats^17,28^. The compound has also been reported to produce hyperglycaemia^17,20,27,28^ and been associated with the induction of pancreatic islet injury in rats^29^. In addition, RDC5 has been linked to the development of new-onset diabetes following kidney and liver transplantation in man^30,31^. The exact mechanisms underlying the diabetogenic properties of RDC5 have not been elucidated but they appear to be related to its pharmacological actions as a calcineurin inhibitor and immunosuppressant^32^.

In the current study, RDC5 given alone or in combination with bupropion or naltrexone, did not significantly alter pancreas weight. Furthermore, RDC5 did

not increase plasma glucose levels measured in fed rats at termination or in fasted rats ie at the B1 timepoint of the OGTT though under both conditions, plasma insulin levels were significantly reduced. Decreases in terminal plasma insulin levels in the freely-feeding rats were also observed in animals given naltrexone, the combination of RDC5 and naltrexone and the combination of bupropion and naltrexone but not RDC5 with bupropion or bupropion alone. These results indicate that the reductions in plasma insulin in the bupropion and naltrexone combination group were due to naltrexone. Although the results of the OGTT indicated that sibutramine improved insulin sensitivity it did not reduce plasma glucose or insulin levels at termination.

Finally livers were also weighed at the end of the study. The increase in liver weights of animals given bupropion, RDC5 with bupropion or bupropion with naltrexone, relative to the final carcass weight, would appear to be due to bupropion as RDC5 did not increase liver weights expressed in this manner, when given alone or in combination with naltrexone. Livers of the bupropion-treated groups of rats were not examined further apart from gross macroscopic examination at termination. Liver lesions and masses were observed in small numbers of animals given bupropion but also in the other treatment groups therefore these effects were not treatment-related as mentioned above. It is more likely that the observed signs of liver lesions were caused by the repeated ip administration.

In summary, chronic administration of RDC5 (0.6 mg/kg po) produced a modest reduction in body weight in dietary-induced obese, female Wistar rats, ie an animal model with excellent predictive validity for weight-loss in the clinic. The reduction in body weight produced by RDC5 was approximately half of that produced by the 5-HT and noradrenaline reuptake inhibitor and reference compound, sibutramine (5 mg/kg po). The weight-loss produced by RDC5, could be explained at least in part, by reduced food intake. RDC5 significantly reduced plasma insulin levels during an OGTT and plasma insulin levels at the B1 baseline timepoint of the OGTT and at termination but had little effect on plasma glucose levels in fed or fasted rats or following the glucose challenge. While the

decrease in plasma insulin levels could indicate improved insulin sensitivity, the same dose of RDC5 significantly reduced pancreatic insulin levels by approximately 50%. This would be an unwanted adverse effect in obese patients who are often insulin resistant and who may also have type 2 diabetes. Greater weight-loss may have been achieved in the dietary-induced obese rats at higher doses of RDC5. However, it is clear that the drug has a narrow therapeutic index and is unlikely to produce meaningful weight-loss without detrimental effects on pancreatic function. One of the aims of the study was to investigate whether the effects of a moderate dose of RDC5 on body weight could be potentiated by bupriopion or naltrexone ie drugs which reduce body weight via complementary mechanisms. There was no evidence that this occurred in the current study though bupropion and naltrexone alone or in combination, did not reduce body weight at the doses tested.

In conclusion, the results of this study do not support the development of RDC5 for the treatment of obesity either given alone or in combination with bupropion or naltrexone.

#### REFERENCES

1. Cecchini M, Sassi F, Lauer JA, Lee YY, Guajardo-Barron V, Chisholm D. Tackling of unhealthy diets, physical inactivity, and obesity: health effects and cost-effectiveness. Lancet 2010; 376: 1775-1784.
2. Adeboye B, Bermano G, Rolland C. Obesity and its health impact in Africa: a systematic review. Cardiovasc J Afr. 2012; 23: 512-521.
3. World Health Organisation Factsheet 311. 2015. <http://www.who.int/mediacentre/factsheets/fs311/en/>.
4. Pi-Sunyer FX. The obesity epidemic: pathophysiology and consequences of obesity. Obes Res. 2002; 10(2): 97-104S.
5. Lorber D. Importance of cardiovascular disease risk management in patients with type 2 diabetes mellitus. Diabetes Metab Syndr Obes. 2014; 7: 169-183.
6. DeMarco VG, Aroor AR, Sowers JR. The pathophysiology of hypertension in patients with obesity. Nat Rev Endocrinol. 2014; 10: 364-376.
7. van Rooy MJ, Pretorius E. Obesity, hypertension and hypercholesterolemia as risk factors for atherosclerosis leading to ischemic events. Curr Med Chem. 2014; 21: 2121-2129.
8. Ebbert JO, Jensen MD. Fat depots, free fatty acids, and dyslipidemia. Nutrients 2013; 5: 498-508.
9. Park J, Morley TS, Kim M, Clegg DJ, Scherer PE. Obesity and cancer - mechanisms underlying tumour progression and recurrence. Nat Rev Endocrinol. 2014; 10(8): 455-465.
10. Lashinger LM, Rossi EL, Hursting SD. Obesity and resistance to cancer chemotherapy: interacting roles of inflammation and metabolic dysregulation. Clin Pharmacol Ther. 2014; 96(4): 458-463.
11. Wolk R, Shamsuzzaman AS, Somers VK. Obesity, sleep apnoea, and hypertension. Hypertension 2003; 42: 1067-1074.
12. Wing R, Lang W, Wadden TA, Safford M, Knowler WC, Bertoni AG, Hill JO, Brancati FL, Peters A, Wagenknecht L; Look AHEAD Research Group. Benefits of modest weight loss in improving cardiovascular risk factors in overweight and obese individuals with type 2 diabetes. Diabetes Care 2011; 34: 1481-1486.
13. Greenway FL, Dunayevich E, Tollefson G, Erickson J, Guttadauria M, Fujioka K, Cowley MA. Comparison of combined bupropion and naltrexone therapy for obesity with monotherapy and placebo. J. Clin. Endocrinol. Metab. 2009; 94(12): 4898-4906.
14. Apovian CM, Aronne L, Rubino D, Still C, Wyatt H, Burns C, Kim D, Dunayevich E. A Randomized, Phase 3 Trial of Naltrexone SR/Bupropion SR on Weight and Obesity-related Risk factors (COR-II). Obesity 2013; 21: 935-943.
15. Caixas A, Albert L, Capel I, Rigla M. Naltrexone sustained-release/bupropion sustained-release for the management of obesity: review of data to date. Drug Design, Development and Therapy 2014; 8: 1419-1419.
16. Overton HA, Babbs AJ, Doel SM, Fyfe MC, Gardner LS, Griffin G, Jackson HC, Procter MJ, Rasamison CM, Tang-Christensen M, Widdowson PS, Williams GM, Reynet C. Deorphanization of a G protein-coupled receptor for oleoylethanolamide and its use in the discovery of small-molecule hypophagic agents. Cell Metab. 2006; 3(3): 167-175.
17. Vickers SP, Cheetham SC. Effects of RDC5 on body weight, food and water intake in male Sprague Dawley rats maintained on a high-fat diet. RenaSci RS1177; 2014.
18. Vickers SP, Jackson HC, Cheetham SC. The utility of animal models to evaluate novel anti-obesity agents. Br J Pharmacol 2011; 164: 1248-1262.
19. Dickinson K, North TJ, Anthony DM, Jones RB, Heal DJ. Evaluation of a simplified cafeteria model for the induction of insulin resistant obesity in rats. Int J Obesity 1998; 22 (Suppl 3): S179.
20. Rodriguez-Rodriguez AE, Triñanes J, Velazquez-Garcia S, Porrini E, Vega Prieto MJ, Diez Fuentes ML, Arevalo M, Salido Ruiz E, Torres A. The higher diabetogenic risk of tacrolimus depends on pre-existing insulin resistance. A study in obese and lean Zucker rats. Am J Transplant 2013; 13(7): 1665-1675.
21. Fisas A, Codony X, Romero G, Dordal A, Giraldo J, Mercé R, Holenz J, Vrang N, Sørensen RV, Heal D, Buschmann H, Pauwels PJ. Chronic 5-HT6 receptor modulation by E-6837 induces hypophagia and sustained weight loss in diet-induced obese rats. Br J Pharmacol. 2006; 148(7): 973-983.
22. Vickers SP, Cheetham SC, Headland KR, Dickinson K, Grempler R, Mayoux E, Mark M, Klein T. Combination of the sodium-glucose cotransporter-2 inhibitor empagliflozin with orlistat or sibutramine further improves the body-weight reduction and glucose homeostasis of obese rats fed a cafeteria diet. Diabetes Metab Syndr Obes. 2014; 7: 265-275.
23. Rebello CJ, Greenaway FL. Reward-induced eating: therapeutic approaches to addressing food cravings. Advances in Therapy 2016; 11: 1853-1866.
24. Billes SK, Sinnayah P, Cowley MA. Naltrexone/bupropion for obesity: an investigational combination pharmacology for weight-loss. Pharmacol Res. 2014; 84: 1-11.
25. Schenk S, Harber MP, Shrivastava CR, Burant CF, Horowitz JF. Improved insulin sensitivity after weight loss and exercise training is mediated by a reduction in plasma fatty acid mobilization, not enhanced oxidative capacity. J Physiol. 2009; 587(20): 4949–4961.
26. Mondon CE, Sims C, Dolkas CB, Reaven EP, Reaven GM. The effect of exercise training on insulin resistance in sedentary year old rats. J Gerontol. 1986; 41(5): 605-610.
27. Xu C, Niu YJ, Liu XJ, Teng YQ, Li CF, Wang HY, Yin JP, Wang LT, Shen ZY. Tacrolimus reversibly reduces insulin secretion, induces insulin resistance, and causes islet cell damage in rats. Int J Clin Pharmacol Ther. 2014; 52(7): 620-627.
28. Hernández-Fisac I, Pizarro-Delgado J, Calle C, Marques M, Sánchez A, Barrientos A, Tamarit-Rodriguez J. Tacrolimus-induced diabetes in rats courses with suppressed insulin gene expression in pancreatic islets. Am J Transplant 2007; 7(11): 2455-2462.
29. Jin L, Lim SW, Doh KC, Piao SG, Jin J, Heo SB, Chung BH, Yang CW. Dipeptidyl peptidase IV inhibitor MK-0626 attenuates pancreatic islet injury in tacrolimus-induced diabetic rats. PLoS One 2014; 9(6): e100798.
30. Li DW, Lu TF, Hua XW, Dai HJ, Cui XL, Zhang JJ, Xia Q. Risk factors for new onset diabetes mellitus after liver transplantation: A meta-analysis. World J Gastroenterol. 2015; 21(20): 6329-6340.
31. Chakkera HA & Mandarino LJ. Calcineurin inhibition and new-onset diabetes mellitus after transplantation. Transplantation 2013 March 15; 95(5): doi:10.1097/TP.0b013e31826e592e.
32. Chakkera HA, Kudva Y, Kaplan B. Calcineurin Inhibitors: Pharmacologic mechanisms impacting both insulin resistance and insulin secretion leading to glucose dysregulation and diabetes mellitus. Clin Pharmacol Ther 2016; Nov

2. doi: 10.1002/cpt.546. [Epub ahead of print].

#### Table 1 Body weights (g) on each day and comparisons of combination treatments to their individual constituents (Baseline, Weeks 1 to 6)

**Body weights (g) on each day (Baseline)**

Treatment n Mean SEM Difference % change p from vehicle

| Day -6 | Vehicle 4 ml/kg po +2 ml/kg ip | 12 | 410.9 | 3.5 |  | | |
| --- | --- | --- | --- | --- | --- | --- | --- |
|  | RDC5 0.6 mg/kg po | 10 | 412.5 | 2.1 | 1.6 | 0.4 | 0.725 |
|  | Bupropion 20 mg/kg ip qd | 10 | 414.5 | 2.8 | 3.6 | 0.9 | 0.419 |
|  | Naltrexone 1 mg/kg ip | 10 | 413.6 | 3.2 | 2.7 | 0.6 | 0.553 |
|  | RDC5 0.6 mg/kg po+Bupropion 20 mg/kg ip qd | 10 | 412.1 | 3.7 | 1.2 | 0.3 | 0.788 |
|  | RDC5 0.6 mg/kg po+Naltrexone 1 mg/kg ip | 10 | 411.4 | 1.9 | 0.5 | 0.1 | 0.911 |
|  | Bupropion 20 mg/kg ip qd+Naltrexone 1 mg/kg ip | 10 | 415.0 | 2.3 | 4.2 | 1.0 | 0.356 |
|  | Sibutramine 5 mg/kg po | 10 | 415.8 | 4.9 | 4.9 | 1.2 | 0.278 |
| Day -5 | Vehicle 4 ml/kg po +2 ml/kg ip | 12 | 413.4 | 2.9 |  |  |  |
|  | RDC5 0.6 mg/kg po | 10 | 413.1 | 1.8 | -0.4 | -0.1 | 0.928 |
|  | Bupropion 20 mg/kg ip qd | 10 | 416.0 | 2.8 | 2.6 | 0.6 | 0.499 |
|  | Naltrexone 1 mg/kg ip | 10 | 418.8 | 3.5 | 5.4 | 1.3 | 0.172 |
|  | RDC5 0.6 mg/kg po+Bupropion 20 mg/kg ip qd | 10 | 411.6 | 2.4 | -1.8 | -0.4 | 0.653 |
|  | RDC5 0.6 mg/kg po+Naltrexone 1 mg/kg ip | 10 | 413.5 | 1.8 | 0.1 | 0.0 | 0.986 |
|  | Bupropion 20 mg/kg ip qd+Naltrexone 1 mg/kg ip | 10 | 415.3 | 1.9 | 1.9 | 0.5 | 0.622 |
|  | Sibutramine 5 mg/kg po | 10 | 418.1 | 4.2 | 4.7 | 1.1 | 0.230 |
| Day -4 | Vehicle 4 ml/kg po +2 ml/kg ip | 12 | 416.8 | 2.2 |  |  |  |
|  | RDC5 0.6 mg/kg po | 10 | 416.9 | 2.1 | 0.1 | 0.0 | 0.978 |
|  | Bupropion 20 mg/kg ip qd | 10 | 420.1 | 2.8 | 3.3 | 0.8 | 0.363 |
|  | Naltrexone 1 mg/kg ip | 10 | 421.1 | 3.4 | 4.3 | 1.0 | 0.230 |
|  | RDC5 0.6 mg/kg po+Bupropion 20 mg/kg ip qd | 10 | 414.8 | 2.5 | -2.0 | -0.5 | 0.581 |
|  | RDC5 0.6 mg/kg po+Naltrexone 1 mg/kg ip | 10 | 416.1 | 2.1 | -0.6 | -0.2 | 0.859 |
|  | Bupropion 20 mg/kg ip qd+Naltrexone 1 mg/kg ip | 10 | 418.1 | 1.7 | 1.3 | 0.3 | 0.718 |
|  | Sibutramine 5 mg/kg po | 10 | 421.0 | 3.3 | 4.2 | 1.0 | 0.240 |
| Day -3 | Vehicle 4 ml/kg po +2 ml/kg ip | 12 | 417.4 | 2.0 |  |  |  |
|  | RDC5 0.6 mg/kg po | 10 | 417.9 | 1.4 | 0.5 | 0.1 | 0.862 |
|  | Bupropion 20 mg/kg ip qd | 10 | 420.7 | 2.6 | 3.4 | 0.8 | 0.277 |
|  | Naltrexone 1 mg/kg ip | 10 | 419.6 | 2.6 | 2.2 | 0.5 | 0.471 |
|  | RDC5 0.6 mg/kg po+Bupropion 20 mg/kg ip qd | 10 | 416.9 | 2.2 | -0.5 | -0.1 | 0.876 |
|  | RDC5 0.6 mg/kg po+Naltrexone 1 mg/kg ip | 10 | 416.0 | 0.9 | -1.4 | -0.3 | 0.653 |
|  | Bupropion 20 mg/kg ip qd+Naltrexone 1 mg/kg ip | 10 | 418.4 | 1.6 | 1.0 | 0.2 | 0.737 |
|  | Sibutramine 5 mg/kg po | 10 | 420.7 | 3.3 | 3.3 | 0.8 | 0.285 |
| Day -2 | Vehicle 4 ml/kg po +2 ml/kg ip | 12 | 420.3 | 2.3 |  |  |  |
|  | RDC5 0.6 mg/kg po | 10 | 419.8 | 1.1 | -0.5 | -0.1 | 0.870 |
|  | Bupropion 20 mg/kg ip qd | 10 | 419.6 | 2.1 | -0.7 | -0.2 | 0.798 |
|  | Naltrexone 1 mg/kg ip | 10 | 419.9 | 1.9 | -0.4 | -0.1 | 0.877 |
|  | RDC5 0.6 mg/kg po+Bupropion 20 mg/kg ip qd | 10 | 419.4 | 1.8 | -0.9 | -0.2 | 0.751 |
|  | RDC5 0.6 mg/kg po+Naltrexone 1 mg/kg ip | 10 | 418.9 | 1.2 | -1.4 | -0.3 | 0.614 |
|  | Bupropion 20 mg/kg ip qd+Naltrexone 1 mg/kg ip | 10 | 419.2 | 1.4 | -1.1 | -0.3 | 0.701 |
|  | Sibutramine 5 mg/kg po | 10 | 420.4 | 3.0 | 0.1 | 0.0 | 0.975 |
| Day -1 | Vehicle 4 ml/kg po +2 ml/kg ip | 12 | 422.1 | 2.2 |  |  |  |
|  | RDC5 0.6 mg/kg po | 10 | 421.3 | 1.5 | -0.8 | -0.2 | 0.744 |
|  | Bupropion 20 mg/kg ip qd | 10 | 419.4 | 1.5 | -2.8 | -0.7 | 0.276 |
|  | Naltrexone 1 mg/kg ip | 10 | 419.5 | 1.9 | -2.6 | -0.6 | 0.299 |
|  | RDC5 0.6 mg/kg po+Bupropion 20 mg/kg ip qd | 10 | 420.5 | 1.9 | -1.6 | -0.4 | 0.522 |
|  | RDC5 0.6 mg/kg po+Naltrexone 1 mg/kg ip | 10 | 419.8 | 1.3 | -2.3 | -0.5 | 0.363 |
|  | Bupropion 20 mg/kg ip qd+Naltrexone 1 mg/kg ip | 10 | 420.6 | 1.5 | -1.6 | -0.4 | 0.538 |
|  | Sibutramine 5 mg/kg po | 10 | 422.9 | 2.1 | 0.7 | 0.2 | 0.774 |
| Day 0 | Vehicle 4 ml/kg po +2 ml/kg ip | 12 | 422.3 | 1.6 |  |  |  |
|  | RDC5 0.6 mg/kg po | 10 | 419.9 | 1.5 | -2.3 | -0.6 | 0.226 |
|  | Bupropion 20 mg/kg ip qd | 10 | 418.9 | 0.8 | -3.4 | -0.8 | 0.083 |
|  | Naltrexone 1 mg/kg ip | 10 | 423.2 | 1.7 | 0.9 | 0.2 | 0.629 |
|  | RDC5 0.6 mg/kg po+Bupropion 20 mg/kg ip qd | 10 | 420.7 | 1.2 | -1.6 | -0.4 | 0.419 |
|  | RDC5 0.6 mg/kg po+Naltrexone 1 mg/kg ip | 10 | 420.1 | 1.0 | -2.2 | -0.5 | 0.257 |
|  | Bupropion 20 mg/kg ip qd+Naltrexone 1 mg/kg ip | 10 | 420.3 | 1.4 | -2.0 | -0.5 | 0.296 |
|  | Sibutramine 5 mg/kg po | 10 | 424.7 | 1.3 | 2.4 | 0.6 | 0.213 |
| Day 1 | Vehicle 4 ml/kg po +2 ml/kg ip | 12 | 421.9 | 0.0 |  |  |  |
|  | RDC5 0.6 mg/kg po | 10 | 421.9 | 0.0 |  |  |  |
|  | Bupropion 20 mg/kg ip qd | 10 | 421.9 | 0.0 |  |  |  |
|  | Naltrexone 1 mg/kg ip | 10 | 421.9 | 0.0 |  |  |  |
|  | RDC5 0.6 mg/kg po+Bupropion 20 mg/kg ip qd | 10 | 421.9 | 0.0 |  |  |  |
|  | RDC5 0.6 mg/kg po+Naltrexone 1 mg/kg ip | 10 | 421.9 | 0.0 |  |  |  |
|  | Bupropion 20 mg/kg ip qd+Naltrexone 1 mg/kg ip | 10 | 421.9 | 0.0 |  |  |  |
|  | Sibutramine 5 mg/kg po | 10 | 421.9 | 0.0 |  |  |  |

Means are adjusted for differences between the treatment groups at baseline (Day 1). On Day 1, all data are adjusted to become the overall mean, hence all means are the same and SEM are 0. SEM are calculated from the residuals of the statistical model.Multiple comparisons against vehicle are by the multiple t test.

Treatment n Mean SEM Difference % change p

from vehicle

| Day 2 | Vehicle 4 ml/kg po +2 ml/kg ip | 12 | 423.2 | 1.2 |  |  |  |
| --- | --- | --- | --- | --- | --- | --- | --- |
|  | RDC5 0.6 mg/kg po | 10 | 421.7 | 1.1 | -1.5 | -0.4 | 0.405 |
|  | Bupropion 20 mg/kg ip qd | 10 | 420.3 | 1.1 | -2.9 | -0.7 | 0.111 |
|  | Naltrexone 1 mg/kg ip | 10 | 420.6 | 1.2 | -2.6 | -0.6 | 0.161 |
|  | RDC5 0.6 mg/kg po+Bupropion 20 mg/kg ip qd | 10 | 421.4 | 1.3 | -1.8 | -0.4 | 0.337 |
|  | RDC5 0.6 mg/kg po+Naltrexone 1 mg/kg ip | 10 | 420.3 | 1.6 | -2.9 | -0.7 | 0.119 |
|  | Bupropion 20 mg/kg ip qd+Naltrexone 1 mg/kg ip | 10 | 419.7 | 1.0 | -3.5 | -0.8 | 0.056 |
|  | Sibutramine 5 mg/kg po | 10 | 405.2 | 1.8 | -18.0 | -4.3 | <0.001*** |
| Day 3 | Vehicle 4 ml/kg po +2 ml/kg ip | 12 | 423.9 | 1.7 |  |  |  |
|  | RDC5 0.6 mg/kg po | 10 | 422.0 | 1.4 | -1.9 | -0.4 | 0.442 |
|  | Bupropion 20 mg/kg ip qd | 10 | 421.6 | 1.7 | -2.3 | -0.5 | 0.348 |
|  | Naltrexone 1 mg/kg ip | 10 | 422.3 | 1.2 | -1.5 | -0.4 | 0.521 |
|  | RDC5 0.6 mg/kg po+Bupropion 20 mg/kg ip qd | 10 | 422.8 | 1.2 | -1.1 | -0.3 | 0.653 |
|  | RDC5 0.6 mg/kg po+Naltrexone 1 mg/kg ip | 10 | 420.4 | 2.0 | -3.5 | -0.8 | 0.146 |
|  | Bupropion 20 mg/kg ip qd+Naltrexone 1 mg/kg ip | 10 | 419.2 | 1.9 | -4.7 | -1.1 | 0.055 |
|  | Sibutramine 5 mg/kg po | 10 | 399.8 | 2.4 | -24.1 | -5.7 | <0.001*** |
| Day 4 | Vehicle 4 ml/kg po +2 ml/kg ip | 12 | 424.3 | 1.7 |  |  |  |
|  | RDC5 0.6 mg/kg po | 10 | 420.5 | 1.8 | -3.8 | -0.9 | 0.175 |
|  | Bupropion 20 mg/kg ip qd | 10 | 419.9 | 1.7 | -4.5 | -1.1 | 0.114 |
|  | Naltrexone 1 mg/kg ip | 10 | 422.7 | 2.0 | -1.7 | -0.4 | 0.547 |
|  | RDC5 0.6 mg/kg po+Bupropion 20 mg/kg ip qd | 10 | 423.2 | 1.9 | -1.2 | -0.3 | 0.671 |
|  | RDC5 0.6 mg/kg po+Naltrexone 1 mg/kg ip | 10 | 421.4 | 2.2 | -2.9 | -0.7 | 0.296 |
|  | Bupropion 20 mg/kg ip qd+Naltrexone 1 mg/kg ip | 10 | 419.5 | 2.1 | -4.8 | -1.1 | 0.090 |
|  | Sibutramine 5 mg/kg po | 10 | 396.4 | 2.7 | -27.9 | -6.6 | <0.001*** |
| Day 5 | Vehicle 4 ml/kg po +2 ml/kg ip | 12 | 425.5 | 2.0 |  |  |  |
|  | RDC5 0.6 mg/kg po | 10 | 420.8 | 1.7 | -4.6 | -1.1 | 0.117 |
|  | Bupropion 20 mg/kg ip qd | 10 | 418.3 | 2.1 | -7.2 | -1.7 | 0.017* |
|  | Naltrexone 1 mg/kg ip | 10 | 420.9 | 1.7 | -4.6 | -1.1 | 0.124 |
|  | RDC5 0.6 mg/kg po+Bupropion 20 mg/kg ip qd | 10 | 422.6 | 1.6 | -2.9 | -0.7 | 0.331 |
|  | RDC5 0.6 mg/kg po+Naltrexone 1 mg/kg ip | 10 | 420.3 | 2.8 | -5.2 | -1.2 | 0.081 |
|  | Bupropion 20 mg/kg ip qd+Naltrexone 1 mg/kg ip | 10 | 421.5 | 2.6 | -4.0 | -0.9 | 0.180 |
|  | Sibutramine 5 mg/kg po | 10 | 393.8 | 2.1 | -31.7 | -7.5 | <0.001*** |
| Day 6 | Vehicle 4 ml/kg po +2 ml/kg ip | 12 | 427.2 | 1.9 |  |  |  |
|  | RDC5 0.6 mg/kg po | 10 | 419.3 | 1.8 | -7.9 | -1.8 | 0.021* |
|  | Bupropion 20 mg/kg ip qd | 10 | 420.2 | 1.9 | -7.1 | -1.7 | 0.038* |
|  | Naltrexone 1 mg/kg ip | 10 | 420.5 | 2.4 | -6.8 | -1.6 | 0.048* |
|  | RDC5 0.6 mg/kg po+Bupropion 20 mg/kg ip qd | 10 | 421.7 | 1.7 | -5.5 | -1.3 | 0.103 |
|  | RDC5 0.6 mg/kg po+Naltrexone 1 mg/kg ip | 10 | 422.3 | 3.0 | -4.9 | -1.2 | 0.145 |
|  | Bupropion 20 mg/kg ip qd+Naltrexone 1 mg/kg ip | 10 | 423.9 | 3.3 | -3.4 | -0.8 | 0.320 |
|  | Sibutramine 5 mg/kg po | 10 | 391.6 | 2.9 | -35.6 | -8.3 | <0.001*** |
| Day 7 | Vehicle 4 ml/kg po +2 ml/kg ip | 12 | 426.5 | 2.2 |  |  |  |
|  | RDC5 0.6 mg/kg po | 10 | 417.0 | 1.7 | -9.5 | -2.2 | 0.021* |
|  | Bupropion 20 mg/kg ip qd | 10 | 420.8 | 2.8 | -5.6 | -1.3 | 0.164 |
|  | Naltrexone 1 mg/kg ip | 10 | 421.5 | 3.2 | -4.9 | -1.2 | 0.223 |
|  | RDC5 0.6 mg/kg po+Bupropion 20 mg/kg ip qd | 10 | 420.3 | 1.8 | -6.2 | -1.5 | 0.128 |
|  | RDC5 0.6 mg/kg po+Naltrexone 1 mg/kg ip | 10 | 421.5 | 3.3 | -5.0 | -1.2 | 0.219 |
|  | Bupropion 20 mg/kg ip qd+Naltrexone 1 mg/kg ip | 10 | 424.2 | 4.4 | -2.3 | -0.5 | 0.573 |
|  | Sibutramine 5 mg/kg po | 10 | 389.1 | 3.1 | -37.4 | -8.8 | <0.001*** |
| Day 8 | Vehicle 4 ml/kg po +2 ml/kg ip | 12 | 428.7 | 2.4 |  |  |  |
|  | RDC5 0.6 mg/kg po | 10 | 415.4 | 2.3 | -13.3 | -3.1 | 0.004** |
|  | Bupropion 20 mg/kg ip qd | 10 | 421.2 | 3.0 | -7.5 | -1.7 | 0.096 |
|  | Naltrexone 1 mg/kg ip | 10 | 424.0 | 3.4 | -4.6 | -1.1 | 0.299 |
|  | RDC5 0.6 mg/kg po+Bupropion 20 mg/kg ip qd | 10 | 419.1 | 1.6 | -9.5 | -2.2 | 0.035* |
|  | RDC5 0.6 mg/kg po+Naltrexone 1 mg/kg ip | 10 | 420.2 | 3.7 | -8.5 | -2.0 | 0.060 |
|  | Bupropion 20 mg/kg ip qd+Naltrexone 1 mg/kg ip | 10 | 424.9 | 4.6 | -3.7 | -0.9 | 0.404 |
|  | Sibutramine 5 mg/kg po | 10 | 389.3 | 3.8 | -39.4 | -9.2 | <0.001*** |

Means are adjusted for differences between the treatment groups at baseline (Day 1). SEM are calculated from the residuals of the statistical model. Multiple comparisons against vehicle are by the multiple t test. *p<0.05, **p<0.01, ***p<0.001.

Treatment n Mean SEM Difference % change p

from vehicle

| Day 9 | Vehicle 4 ml/kg po +2 ml/kg ip | 12 | 429.7 | 2.0 | | | |
| --- | --- | --- | --- | --- | --- | --- | --- |
|  | RDC5 0.6 mg/kg po | 10 | 414.8 | 3.0 | -14.9 | -3.5 | 0.001** |
|  | Bupropion 20 mg/kg ip qd | 10 | 421.5 | 3.9 | -8.2 | -1.9 | 0.074 |
|  | Naltrexone 1 mg/kg ip | 10 | 426.1 | 3.6 | -3.6 | -0.8 | 0.424 |
|  | RDC5 0.6 mg/kg po+Bupropion 20 mg/kg ip qd | 10 | 418.0 | 1.5 | -11.7 | -2.7 | 0.011* |
|  | RDC5 0.6 mg/kg po+Naltrexone 1 mg/kg ip | 10 | 421.1 | 3.3 | -8.6 | -2.0 | 0.059 |
|  | Bupropion 20 mg/kg ip qd+Naltrexone 1 mg/kg ip | 10 | 427.4 | 4.1 | -2.3 | -0.5 | 0.606 |
|  | Sibutramine 5 mg/kg po | 10 | 388.3 | 3.8 | -41.4 | -9.6 | <0.001*** |
| Day 10 | Vehicle 4 ml/kg po +2 ml/kg ip | 12 | 431.3 | 2.5 |  |  |  |
|  | RDC5 0.6 mg/kg po | 10 | 412.0 | 3.9 | -19.3 | -4.5 | <0.001*** |
|  | Bupropion 20 mg/kg ip qd | 10 | 423.4 | 4.1 | -8.0 | -1.8 | 0.112 |
|  | Naltrexone 1 mg/kg ip | 10 | 426.1 | 3.5 | -5.2 | -1.2 | 0.298 |
|  | RDC5 0.6 mg/kg po+Bupropion 20 mg/kg ip qd | 10 | 418.9 | 1.6 | -12.4 | -2.9 | 0.015* |
|  | RDC5 0.6 mg/kg po+Naltrexone 1 mg/kg ip | 10 | 422.0 | 3.2 | -9.3 | -2.2 | 0.063 |
|  | Bupropion 20 mg/kg ip qd+Naltrexone 1 mg/kg ip | 10 | 429.4 | 4.6 | -1.9 | -0.5 | 0.695 |
|  | Sibutramine 5 mg/kg po | 10 | 386.7 | 4.5 | -44.6 | -10.3 | <0.001*** |
| Day 11 | Vehicle (4 ml/kg po +2 ml/kg ip) | 12 | 429.4 | 3.2 |  |  |  |
|  | RDC5 0.6 mg/kg po | 10 | 411.4 | 3.7 | -18.0 | -4.2 | 0.002** |
|  | Bupropion 20 mg/kg ip qd | 10 | 422.4 | 3.5 | -7.0 | -1.6 | 0.216 |
|  | Naltrexone 1 mg/kg ip | 10 | 427.8 | 4.0 | -1.6 | -0.4 | 0.778 |
|  | RDC5 0.6 mg/kg po+Bupropion 20 mg/kg ip qd | 10 | 416.9 | 2.2 | -12.5 | -2.9 | 0.028* |
|  | RDC5 0.6 mg/kg po+Naltrexone 1 mg/kg ip | 10 | 423.0 | 4.3 | -6.4 | -1.5 | 0.257 |
|  | Bupropion 20 mg/kg ip qd+Naltrexone 1 mg/kg ip | 10 | 431.0 | 6.1 | 1.7 | 0.4 | 0.765 |
|  | Sibutramine 5 mg/kg po | 10 | 386.7 | 4.2 | -42.6 | -9.9 | <0.001*** |
| Day 12 | Vehicle (4 ml/kg po +2 ml/kg ip) | 12 | 430.2 | 3.6 |  |  |  |
|  | RDC5 0.6 mg/kg po | 10 | 410.7 | 3.5 | -19.5 | -4.5 | 0.002** |
|  | Bupropion 20 mg/kg ip qd | 10 | 424.9 | 3.8 | -5.3 | -1.2 | 0.369 |
|  | Naltrexone 1 mg/kg ip | 10 | 429.9 | 5.0 | -0.3 | -0.1 | 0.961 |
|  | RDC5 0.6 mg/kg po+Bupropion 20 mg/kg ip qd | 10 | 415.3 | 2.0 | -14.9 | -3.5 | 0.014* |
|  | RDC5 0.6 mg/kg po+Naltrexone 1 mg/kg ip | 10 | 421.2 | 4.6 | -9.0 | -2.1 | 0.133 |
|  | Bupropion 20 mg/kg ip qd+Naltrexone 1 mg/kg ip | 10 | 432.4 | 6.6 | 2.2 | 0.5 | 0.715 |
|  | Sibutramine 5 mg/kg po | 10 | 387.6 | 3.7 | -42.6 | -9.9 | <0.001*** |
| Day 13 | Vehicle (4 ml/kg po +2 ml/kg ip) | 12 | 432.4 | 3.7 |  |  |  |
|  | RDC5 0.6 mg/kg po | 10 | 411.6 | 4.3 | -20.9 | -4.8 | 0.001** |
|  | Bupropion 20 mg/kg ip qd | 10 | 426.6 | 4.0 | -5.8 | -1.3 | 0.356 |
|  | Naltrexone 1 mg/kg ip | 10 | 429.4 | 5.6 | -3.1 | -0.7 | 0.626 |
|  | RDC5 0.6 mg/kg po+Bupropion 20 mg/kg ip qd | 10 | 415.9 | 2.2 | -16.5 | -3.8 | 0.010* |
|  | RDC5 0.6 mg/kg po+Naltrexone 1 mg/kg ip | 10 | 421.0 | 3.7 | -11.4 | -2.6 | 0.071 |
|  | Bupropion 20 mg/kg ip qd+Naltrexone 1 mg/kg ip | 10 | 437.9 | 6.8 | 5.5 | 1.3 | 0.384 |
|  | Sibutramine 5 mg/kg po | 10 | 389.4 | 4.3 | -43.0 | -9.9 | <0.001*** |
| Day 14 | Vehicle (4 ml/kg po +2 ml/kg ip) | 12 | 433.5 | 3.5 |  |  |  |
|  | RDC5 0.6 mg/kg po | 10 | 411.8 | 5.1 | -21.7 | -5.0 | 0.001** |
|  | Bupropion 20 mg/kg ip qd | 10 | 427.2 | 3.9 | -6.3 | -1.5 | 0.330 |
|  | Naltrexone 1 mg/kg ip | 10 | 429.0 | 5.7 | -4.5 | -1.0 | 0.489 |
|  | RDC5 0.6 mg/kg po+Bupropion 20 mg/kg ip qd | 10 | 416.4 | 2.5 | -17.1 | -3.9 | 0.010** |
|  | RDC5 0.6 mg/kg po+Naltrexone 1 mg/kg ip | 10 | 422.6 | 3.7 | -10.9 | -2.5 | 0.095 |
|  | Bupropion 20 mg/kg ip qd+Naltrexone 1 mg/kg ip | 10 | 436.5 | 6.7 | 3.0 | 0.7 | 0.637 |
|  | Sibutramine 5 mg/kg po | 10 | 389.2 | 4.7 | -44.3 | -10.2 | <0.001*** |
| Day 15 | Vehicle (4 ml/kg po +2 ml/kg ip) | 12 | 432.0 | 4.4 |  |  |  |
|  | RDC5 0.6 mg/kg po | 10 | 408.9 | 5.4 | -23.0 | -5.3 | 0.001** |
|  | Bupropion 20 mg/kg ip qd | 10 | 427.3 | 4.7 | -4.6 | -1.1 | 0.504 |
|  | Naltrexone 1 mg/kg ip | 10 | 431.7 | 5.9 | -0.2 | -0.1 | 0.975 |
|  | RDC5 0.6 mg/kg po+Bupropion 20 mg/kg ip qd | 10 | 415.4 | 3.2 | -16.5 | -3.8 | 0.019* |
|  | RDC5 0.6 mg/kg po+Naltrexone 1 mg/kg ip | 10 | 422.9 | 3.4 | -9.0 | -2.1 | 0.195 |
|  | Bupropion 20 mg/kg ip qd+Naltrexone 1 mg/kg ip | 10 | 436.1 | 6.8 | 4.1 | 1.0 | 0.550 |
|  | Sibutramine 5 mg/kg po | 10 | 386.6 | 5.0 | -45.4 | -10.5 | <0.001*** |

Means are adjusted for differences between the treatment groups at baseline (Day 1). SEM are calculated from the residuals of the statistical model. Multiple comparisons against vehicle are by the multiple t test. *p<0.05, **p<0.01, ***p<0.001.

Treatment n Mean SEM Difference % change p

from vehicle

| Day 16 | Vehicle 4 ml/kg po +2 ml/kg ip | 12 | 430.5 | 4.3 |  |  |  |
| --- | --- | --- | --- | --- | --- | --- | --- |
|  | RDC5 0.6 mg/kg po | 10 | 409.5 | 4.7 | -21.1 | -4.9 | 0.003** |
|  | Bupropion 20 mg/kg ip qd | 10 | 428.1 | 5.0 | -2.4 | -0.6 | 0.729 |
|  | Naltrexone 1 mg/kg ip | 10 | 434.5 | 6.3 | 4.0 | 0.9 | 0.558 |
|  | RDC5 0.6 mg/kg po+Bupropion 20 mg/kg ip qd | 10 | 414.6 | 3.1 | -15.9 | -3.7 | 0.023* |
|  | RDC5 0.6 mg/kg po+Naltrexone 1 mg/kg ip | 10 | 420.8 | 3.5 | -9.7 | -2.2 | 0.160 |
|  | Bupropion 20 mg/kg ip qd+Naltrexone 1 mg/kg ip | 10 | 435.0 | 6.8 | 4.4 | 1.0 | 0.518 |
|  | Sibutramine 5 mg/kg po | 10 | 389.6 | 4.6 | -40.9 | -9.5 | <0.001*** |
| Day 17 | Vehicle 4 ml/kg po +2 ml/kg ip | 12 | 431.5 | 4.6 |  |  |  |
|  | RDC5 0.6 mg/kg po | 10 | 410.5 | 5.5 | -21.0 | -4.9 | 0.005** |
|  | Bupropion 20 mg/kg ip qd | 10 | 427.5 | 5.3 | -4.0 | -0.9 | 0.580 |
|  | Naltrexone 1 mg/kg ip | 10 | 435.3 | 6.6 | 3.8 | 0.9 | 0.603 |
|  | RDC5 0.6 mg/kg po+Bupropion 20 mg/kg ip qd | 10 | 414.7 | 3.7 | -16.8 | -3.9 | 0.023* |
|  | RDC5 0.6 mg/kg po+Naltrexone 1 mg/kg ip | 10 | 420.0 | 3.4 | -11.5 | -2.7 | 0.117 |
|  | Bupropion 20 mg/kg ip qd+Naltrexone 1 mg/kg ip | 10 | 436.4 | 7.3 | 4.9 | 1.1 | 0.498 |
|  | Sibutramine 5 mg/kg po | 10 | 389.7 | 4.3 | -41.9 | -9.7 | <0.001*** |
| Day 18 | Vehicle 4 ml/kg po +2 ml/kg ip | 12 | 434.5 | 4.3 |  |  |  |
|  | RDC5 0.6 mg/kg po | 10 | 407.3 | 6.9 | -27.2 | -6.3 | <0.001*** |
|  | Bupropion 20 mg/kg ip qd | 10 | 430.0 | 5.0 | -4.5 | -1.0 | 0.562 |
|  | Naltrexone 1 mg/kg ip | 10 | 434.1 | 7.6 | -0.4 | -0.1 | 0.958 |
|  | RDC5 0.6 mg/kg po+Bupropion 20 mg/kg ip qd | 10 | 415.6 | 3.8 | -18.9 | -4.3 | 0.018* |
|  | RDC5 0.6 mg/kg po+Naltrexone 1 mg/kg ip | 10 | 420.8 | 3.4 | -13.7 | -3.1 | 0.082 |
|  | Bupropion 20 mg/kg ip qd+Naltrexone 1 mg/kg ip | 10 | 440.2 | 7.5 | 5.6 | 1.3 | 0.470 |
|  | Sibutramine 5 mg/kg po | 10 | 390.0 | 4.8 | -44.5 | -10.2 | <0.001*** |
| Day 19 | Vehicle 4 ml/kg po +2 ml/kg ip | 12 | 436.6 | 3.9 |  |  |  |
|  | RDC5 0.6 mg/kg po | 10 | 409.2 | 7.2 | -27.5 | -6.3 | <0.001*** |
|  | Bupropion 20 mg/kg ip qd | 10 | 432.2 | 4.8 | -4.4 | -1.0 | 0.571 |
|  | Naltrexone 1 mg/kg ip | 10 | 435.3 | 7.5 | -1.4 | -0.3 | 0.859 |
|  | RDC5 0.6 mg/kg po+Bupropion 20 mg/kg ip qd | 10 | 414.9 | 4.4 | -21.7 | -5.0 | 0.006** |
|  | RDC5 0.6 mg/kg po+Naltrexone 1 mg/kg ip | 10 | 424.9 | 3.3 | -11.7 | -2.7 | 0.134 |
|  | Bupropion 20 mg/kg ip qd+Naltrexone 1 mg/kg ip | 10 | 440.1 | 6.7 | 3.5 | 0.8 | 0.652 |
|  | Sibutramine 5 mg/kg po | 10 | 391.9 | 5.3 | -44.7 | -10.2 | <0.001*** |
| Day 20 | Vehicle 4 ml/kg po +2 ml/kg ip | 12 | 437.0 | 3.8 |  |  |  |
|  | RDC5 0.6 mg/kg po | 10 | 411.3 | 7.2 | -25.7 | -5.9 | 0.002** |
|  | Bupropion 20 mg/kg ip qd | 10 | 435.4 | 5.2 | -1.5 | -0.3 | 0.847 |
|  | Naltrexone 1 mg/kg ip | 10 | 439.0 | 7.6 | 2.0 | 0.5 | 0.798 |
|  | RDC5 0.6 mg/kg po+Bupropion 20 mg/kg ip qd | 10 | 415.2 | 5.0 | -21.7 | -5.0 | 0.007** |
|  | RDC5 0.6 mg/kg po+Naltrexone 1 mg/kg ip | 10 | 423.4 | 3.4 | -13.5 | -3.1 | 0.090 |
|  | Bupropion 20 mg/kg ip qd+Naltrexone 1 mg/kg ip | 10 | 441.2 | 6.9 | 4.3 | 1.0 | 0.588 |
|  | Sibutramine 5 mg/kg po | 10 | 393.4 | 5.3 | -43.6 | -10.0 | <0.001*** |
| Day 21 | Vehicle 4 ml/kg po +2 ml/kg ip | 12 | 437.1 | 3.6 |  |  |  |
|  | RDC5 0.6 mg/kg po | 10 | 411.4 | 6.6 | -25.7 | -5.9 | 0.002** |
|  | Bupropion 20 mg/kg ip qd | 10 | 434.2 | 5.6 | -2.9 | -0.7 | 0.713 |
|  | Naltrexone 1 mg/kg ip | 10 | 439.0 | 8.0 | 1.9 | 0.4 | 0.812 |
|  | RDC5 0.6 mg/kg po+Bupropion 20 mg/kg ip qd | 10 | 415.1 | 4.5 | -22.1 | -5.1 | 0.006** |
|  | RDC5 0.6 mg/kg po+Naltrexone 1 mg/kg ip | 10 | 422.2 | 3.6 | -15.0 | -3.4 | 0.061 |
|  | Bupropion 20 mg/kg ip qd+Naltrexone 1 mg/kg ip | 10 | 440.8 | 6.7 | 3.6 | 0.8 | 0.646 |
|  | Sibutramine 5 mg/kg po | 10 | 393.5 | 5.9 | -43.7 | -10.0 | <0.001*** |
| Day 22 | Vehicle 4 ml/kg po +2 ml/kg ip | 12 | 437.8 | 4.0 |  |  |  |
|  | RDC5 0.6 mg/kg po | 10 | 412.2 | 6.7 | -25.6 | -5.8 | 0.003** |
|  | Bupropion 20 mg/kg ip qd | 10 | 434.0 | 4.9 | -3.8 | -0.9 | 0.640 |
|  | Naltrexone 1 mg/kg ip | 10 | 440.8 | 8.0 | 2.9 | 0.7 | 0.720 |
|  | RDC5 0.6 mg/kg po+Bupropion 20 mg/kg ip qd | 10 | 416.3 | 4.8 | -21.5 | -4.9 | 0.011* |
|  | RDC5 0.6 mg/kg po+Naltrexone 1 mg/kg ip | 10 | 424.6 | 3.7 | -13.2 | -3.0 | 0.111 |
|  | Bupropion 20 mg/kg ip qd+Naltrexone 1 mg/kg ip | 10 | 441.1 | 7.6 | 3.3 | 0.8 | 0.688 |
|  | Sibutramine 5 mg/kg po | 10 | 391.5 | 6.5 | -46.4 | -10.6 | <0.001*** |

Means are adjusted for differences between the treatment groups at baseline (Day 1). SEM are calculated from the residuals of the statistical model. Multiple comparisons against vehicle are by the multiple t test. *p<0.05, **p<0.01, ***p<0.001.

Treatment n Mean SEM Difference % change p

from vehicle

| Day 23 | Vehicle 4 ml/kg po +2 ml/kg ip | 12 | 439.6 | 3.9 |  |  |  |
| --- | --- | --- | --- | --- | --- | --- | --- |
|  | RDC5 0.6 mg/kg po | 10 | 414.7 | 6.9 | -24.9 | -5.7 | 0.004** |
|  | Bupropion 20 mg/kg ip qd | 10 | 436.3 | 4.8 | -3.3 | -0.7 | 0.692 |
|  | Naltrexone 1 mg/kg ip | 10 | 443.0 | 8.3 | 3.4 | 0.8 | 0.684 |
|  | RDC5 0.6 mg/kg po+Bupropion 20 mg/kg ip qd | 10 | 419.3 | 4.8 | -20.3 | -4.6 | 0.016* |
|  | RDC5 0.6 mg/kg po+Naltrexone 1 mg/kg ip | 10 | 427.5 | 3.4 | -12.2 | -2.8 | 0.145 |
|  | Bupropion 20 mg/kg ip qd+Naltrexone 1 mg/kg ip | 10 | 442.5 | 7.9 | 2.9 | 0.7 | 0.728 |
|  | Sibutramine 5 mg/kg po | 10 | 393.0 | 6.2 | -46.7 | -10.6 | <0.001*** |
| Day 24 | Vehicle 4 ml/kg po +2 ml/kg ip | 12 | 441.8 | 3.4 |  |  |  |
|  | RDC5 0.6 mg/kg po | 10 | 414.0 | 7.3 | -27.7 | -6.3 | 0.001** |
|  | Bupropion 20 mg/kg ip qd | 10 | 437.9 | 4.9 | -3.8 | -0.9 | 0.647 |
|  | Naltrexone 1 mg/kg ip | 10 | 444.7 | 8.6 | 2.9 | 0.7 | 0.730 |
|  | RDC5 0.6 mg/kg po+Bupropion 20 mg/kg ip qd | 10 | 419.3 | 5.9 | -22.5 | -5.1 | 0.009** |
|  | RDC5 0.6 mg/kg po+Naltrexone 1 mg/kg ip | 10 | 427.4 | 3.5 | -14.4 | -3.3 | 0.089 |
|  | Bupropion 20 mg/kg ip qd+Naltrexone 1 mg/kg ip | 10 | 442.8 | 6.9 | 1.0 | 0.2 | 0.901 |
|  | Sibutramine 5 mg/kg po | 10 | 393.4 | 6.2 | -48.4 | -11.0 | <0.001*** |
| Day 25 | Vehicle 4 ml/kg po +2 ml/kg ip | 12 | 438.7 | 3.1 |  |  |  |
|  | RDC5 0.6 mg/kg po | 10 | 413.0 | 7.6 | -25.7 | -5.9 | 0.003** |
|  | Bupropion 20 mg/kg ip qd | 10 | 438.0 | 5.2 | -0.6 | -0.1 | 0.940 |
|  | Naltrexone 1 mg/kg ip | 10 | 442.4 | 8.5 | 3.7 | 0.8 | 0.664 |
|  | RDC5 0.6 mg/kg po+Bupropion 20 mg/kg ip qd | 10 | 416.9 | 6.8 | -21.8 | -5.0 | 0.012* |
|  | RDC5 0.6 mg/kg po+Naltrexone 1 mg/kg ip | 10 | 424.4 | 3.4 | -14.2 | -3.2 | 0.097 |
|  | Bupropion 20 mg/kg ip qd+Naltrexone 1 mg/kg ip | 10 | 444.2 | 6.6 | 5.6 | 1.3 | 0.512 |
|  | Sibutramine 5 mg/kg po | 10 | 392.7 | 6.3 | -45.9 | -10.5 | <0.001*** |
| Day 26 | Vehicle 4 ml/kg po +2 ml/kg ip | 11 | 439.3 | 3.8 |  |  |  |
|  | RDC5 0.6 mg/kg po | 10 | 414.1 | 7.6 | -25.2 | -5.7 | 0.007** |
|  | Bupropion 20 mg/kg ip bid | 10 | 438.6 | 5.6 | -0.6 | -0.1 | 0.944 |
|  | Naltrexone 1 mg/kg ip | 10 | 443.8 | 8.5 | 4.5 | 1.0 | 0.623 |
|  | RDC5 0.6 mg/kg po+Bupropion 20 mg/kg ip bid | 10 | 419.0 | 7.0 | -20.3 | -4.6 | 0.030* |
|  | RDC5 0.6 mg/kg po+Naltrexone 1 mg/kg ip | 10 | 425.2 | 3.1 | -14.0 | -3.2 | 0.129 |
|  | Bupropion 20 mg/kg ip bid+Naltrexone 1 mg/kg ip | 10 | 445.5 | 7.4 | 6.2 | 1.4 | 0.499 |
|  | Sibutramine 5 mg/kg po | 10 | 394.2 | 7.3 | -45.1 | -10.3 | <0.001*** |
| Day 27 | Vehicle 4 ml/kg po +2 ml/kg ip | 11 | 441.0 | 4.0 |  |  |  |
|  | RDC5 0.6 mg/kg po | 10 | 414.7 | 6.8 | -26.3 | -6.0 | 0.006** |
|  | Bupropion 20 mg/kg ip bid | 10 | 440.9 | 5.8 | -0.1 | -0.0 | 0.994 |
|  | Naltrexone 1 mg/kg ip | 10 | 446.7 | 8.3 | 5.7 | 1.3 | 0.538 |
|  | RDC5 0.6 mg/kg po+Bupropion 20 mg/kg ip bid | 10 | 419.7 | 7.4 | -21.3 | -4.8 | 0.024* |
|  | RDC5 0.6 mg/kg po+Naltrexone 1 mg/kg ip | 10 | 427.1 | 3.4 | -13.9 | -3.2 | 0.136 |
|  | Bupropion 20 mg/kg ip bid+Naltrexone 1 mg/kg ip | 10 | 443.6 | 7.6 | 2.6 | 0.6 | 0.782 |
|  | Sibutramine 5 mg/kg po | 10 | 395.1 | 7.7 | -45.9 | -10.4 | <0.001*** |
| Day 28 | Vehicle 4 ml/kg po +2 ml/kg ip | 11 | 443.1 | 3.3 |  |  |  |
|  | RDC5 0.6 mg/kg po | 10 | 413.8 | 7.5 | -29.3 | -6.6 | 0.002** |
|  | Bupropion 20 mg/kg ip bid | 10 | 440.5 | 5.8 | -2.5 | -0.6 | 0.776 |
|  | Naltrexone 1 mg/kg ip | 10 | 447.8 | 8.1 | 4.8 | 1.1 | 0.593 |
|  | RDC5 0.6 mg/kg po+Bupropion 20 mg/kg ip bid | 10 | 419.3 | 6.8 | -23.8 | -5.4 | 0.009** |
|  | RDC5 0.6 mg/kg po+Naltrexone 1 mg/kg ip | 10 | 427.1 | 3.6 | -16.0 | -3.6 | 0.076 |
|  | Bupropion 20 mg/kg ip bid+Naltrexone 1 mg/kg ip | 10 | 441.3 | 6.8 | -1.8 | -0.4 | 0.840 |
|  | Sibutramine 5 mg/kg po | 10 | 396.0 | 7.3 | -47.1 | -10.6 | <0.001*** |
| Day 29 | Vehicle 4 ml/kg po +2 ml/kg ip | 12 | 439.1 | 3.9 |  |  |  |
|  | RDC5 0.6 mg/kg po | 10 | 414.6 | 7.9 | -24.5 | -5.6 | 0.006** |
|  | Bupropion 20 mg/kg ip bid | 10 | 439.1 | 5.8 | 0.0 | 0.0 | >0.999 |
|  | Naltrexone 1 mg/kg ip | 10 | 446.1 | 7.3 | 7.0 | 1.6 | 0.417 |
|  | RDC5 0.6 mg/kg po+Bupropion 20 mg/kg ip bid | 10 | 418.7 | 7.1 | -20.4 | -4.7 | 0.021* |
|  | RDC5 0.6 mg/kg po+Naltrexone 1 mg/kg ip | 10 | 423.7 | 4.0 | -15.4 | -3.5 | 0.079 |
|  | Bupropion 20 mg/kg ip bid+Naltrexone 1 mg/kg ip | 10 | 442.5 | 6.0 | 3.5 | 0.8 | 0.689 |
|  | Sibutramine 5 mg/kg po | 10 | 394.4 | 7.1 | -44.7 | -10.2 | <0.001*** |

Means are adjusted for differences between the treatment groups at baseline (Day 1). SEM are calculated from the residuals of the statistical model. Multiple comparisons against vehicle are by the multiple t test. *p<0.05, **p<0.01, ***p<0.001.

Treatment n Mean SEM Difference % change p

from vehicle

| Day 30 | Vehicle 4 ml/kg po +2 ml/kg ip | 12 | 440.2 | 4.1 |  |  |  |
| --- | --- | --- | --- | --- | --- | --- | --- |
|  | RDC5 0.6 mg/kg po | 10 | 414.8 | 8.1 | -25.4 | -5.8 | 0.005** |
|  | Bupropion 20 mg/kg ip bid | 10 | 439.0 | 6.1 | -1.2 | -0.3 | 0.891 |
|  | Naltrexone 1 mg/kg ip | 10 | 443.3 | 7.0 | 3.1 | 0.7 | 0.725 |
|  | RDC5 0.6 mg/kg po+Bupropion 20 mg/kg ip bid | 10 | 419.8 | 7.1 | -20.4 | -4.6 | 0.022* |
|  | RDC5 0.6 mg/kg po+Naltrexone 1 mg/kg ip | 10 | 423.6 | 4.1 | -16.6 | -3.8 | 0.061 |
|  | Bupropion 20 mg/kg ip bid+Naltrexone 1 mg/kg ip | 10 | 443.5 | 6.2 | 3.3 | 0.7 | 0.707 |
|  | Sibutramine 5 mg/kg po | 10 | 394.1 | 7.1 | -46.1 | -10.5 | <0.001*** |
| Day 31 | Vehicle 4 ml/kg po +2 ml/kg ip | 12 | 439.6 | 4.6 |  |  |  |
|  | RDC5 0.6 mg/kg po | 10 | 415.3 | 8.1 | -24.3 | -5.5 | 0.009** |
|  | Bupropion 20 mg/kg ip bid | 10 | 439.5 | 6.3 | -0.0 | -0.0 | 0.997 |
|  | Naltrexone 1 mg/kg ip | 10 | 444.4 | 6.9 | 4.8 | 1.1 | 0.595 |
|  | RDC5 0.6 mg/kg po+Bupropion 20 mg/kg ip bid | 10 | 419.2 | 6.8 | -20.3 | -4.6 | 0.027* |
|  | RDC5 0.6 mg/kg po+Naltrexone 1 mg/kg ip | 10 | 424.4 | 4.2 | -15.2 | -3.5 | 0.097 |
|  | Bupropion 20 mg/kg ip bid+Naltrexone 1 mg/kg ip | 10 | 442.6 | 7.4 | 3.1 | 0.7 | 0.734 |
|  | Sibutramine 5 mg/kg po | 10 | 394.2 | 7.4 | -45.3 | -10.3 | <0.001*** |
| Day 32 | Vehicle 4 ml/kg po +2 ml/kg ip | 12 | 438.0 | 5.1 |  |  |  |
|  | RDC5 0.6 mg/kg po | 10 | 413.1 | 8.5 | -24.9 | -5.7 | 0.010* |
|  | Bupropion 20 mg/kg ip bid | 10 | 437.6 | 6.1 | -0.4 | -0.1 | 0.963 |
|  | Naltrexone 1 mg/kg ip | 10 | 444.7 | 7.6 | 6.7 | 1.5 | 0.480 |
|  | RDC5 0.6 mg/kg po+Bupropion 20 mg/kg ip bid | 10 | 416.5 | 7.2 | -21.5 | -4.9 | 0.025* |
|  | RDC5 0.6 mg/kg po+Naltrexone 1 mg/kg ip | 10 | 425.3 | 4.8 | -12.7 | -2.9 | 0.181 |
|  | Bupropion 20 mg/kg ip bid+Naltrexone 1 mg/kg ip | 10 | 443.0 | 7.7 | 5.0 | 1.1 | 0.597 |
|  | Sibutramine 5 mg/kg po | 10 | 394.9 | 6.9 | -43.2 | -9.9 | <0.001*** |
| Day 33 | Vehicle 4 ml/kg po +2 ml/kg ip | 12 | 438.3 | 4.4 |  |  |  |
|  | RDC5 0.6 mg/kg po | 10 | 411.5 | 9.1 | -26.8 | -6.1 | 0.007** |
|  | Bupropion 20 mg/kg ip bid | 10 | 435.9 | 6.4 | -2.4 | -0.5 | 0.805 |
|  | Naltrexone 1 mg/kg ip | 10 | 443.6 | 7.2 | 5.3 | 1.2 | 0.580 |
|  | RDC5 0.6 mg/kg po+Bupropion 20 mg/kg ip bid | 10 | 414.6 | 7.1 | -23.7 | -5.4 | 0.016* |
|  | RDC5 0.6 mg/kg po+Naltrexone 1 mg/kg ip | 10 | 423.2 | 5.3 | -15.1 | -3.5 | 0.119 |
|  | Bupropion 20 mg/kg ip bid+Naltrexone 1 mg/kg ip | 10 | 439.0 | 8.5 | 0.8 | 0.2 | 0.937 |
|  | Sibutramine 5 mg/kg po | 10 | 395.0 | 6.9 | -43.3 | -9.9 | <0.001*** |
| Day 34 | Vehicle 4 ml/kg po +2 ml/kg ip | 12 | 438.2 | 4.1 |  |  |  |
|  | RDC5 0.6 mg/kg po | 10 | 413.4 | 9.0 | -24.8 | -5.7 | 0.010* |
|  | Bupropion 20 mg/kg ip bid | 10 | 437.8 | 6.6 | -0.4 | -0.1 | 0.966 |
|  | Naltrexone 1 mg/kg ip | 10 | 441.9 | 6.3 | 3.7 | 0.8 | 0.697 |
|  | RDC5 0.6 mg/kg po+Bupropion 20 mg/kg ip bid | 10 | 415.1 | 7.6 | -23.1 | -5.3 | 0.016* |
|  | RDC5 0.6 mg/kg po+Naltrexone 1 mg/kg ip | 10 | 425.0 | 4.4 | -13.2 | -3.0 | 0.163 |
|  | Bupropion 20 mg/kg ip bid+Naltrexone 1 mg/kg ip | 10 | 438.7 | 8.2 | 0.5 | 0.1 | 0.959 |
|  | Sibutramine 5 mg/kg po | 10 | 394.6 | 7.1 | -43.6 | -10.0 | <0.001*** |
| Day 35 | Vehicle 4 ml/kg po +2 ml/kg ip | 12 | 436.1 | 4.1 |  |  |  |
|  | RDC5 0.6 mg/kg po | 10 | 412.0 | 9.0 | -24.1 | -5.5 | 0.012* |
|  | Bupropion 20 mg/kg ip bid | 10 | 436.3 | 5.7 | 0.2 | 0.0 | 0.984 |
|  | Naltrexone 1 mg/kg ip | 10 | 440.6 | 6.6 | 4.5 | 1.0 | 0.629 |
|  | RDC5 0.6 mg/kg po+Bupropion 20 mg/kg ip bid | 10 | 415.7 | 7.7 | -20.4 | -4.7 | 0.033* |
|  | RDC5 0.6 mg/kg po+Naltrexone 1 mg/kg ip | 10 | 424.7 | 4.7 | -11.4 | -2.6 | 0.227 |
|  | Bupropion 20 mg/kg ip bid+Naltrexone 1 mg/kg ip | 10 | 438.9 | 8.7 | 2.8 | 0.6 | 0.764 |
|  | Sibutramine 5 mg/kg po | 10 | 395.1 | 6.6 | -41.0 | -9.4 | <0.001*** |
| Day 36 | Vehicle 4 ml/kg po +2 ml/kg ip | 12 | 439.0 | 4.5 |  |  |  |
|  | RDC5 0.6 mg/kg po | 10 | 412.8 | 8.7 | -26.3 | -6.0 | 0.008** |
|  | Bupropion 20 mg/kg ip bid | 10 | 436.2 | 5.3 | -2.8 | -0.6 | 0.773 |
|  | Naltrexone 1 mg/kg ip | 10 | 442.3 | 7.4 | 3.3 | 0.7 | 0.736 |
|  | RDC5 0.6 mg/kg po+Bupropion 20 mg/kg ip bid | 10 | 414.8 | 8.1 | -24.2 | -5.5 | 0.015* |
|  | RDC5 0.6 mg/kg po+Naltrexone 1 mg/kg ip | 10 | 425.7 | 4.7 | -13.3 | -3.0 | 0.175 |
|  | Bupropion 20 mg/kg ip bid+Naltrexone 1 mg/kg ip | 10 | 437.6 | 9.6 | -1.4 | -0.3 | 0.888 |
|  | Sibutramine 5 mg/kg po | 10 | 395.9 | 6.5 | -43.1 | -9.8 | <0.001*** |

Means are adjusted for differences between the treatment groups at baseline (Day 1). SEM are calculated from the residuals of the statistical model. Multiple comparisons against vehicle are by the multiple t test. *p<0.05, **p<0.01, ***p<0.001.

Treatment n Mean SEM Difference % change p

from vehicle

| Day 37 | Vehicle 4 ml/kg po +2 ml/kg ip | 12 | 430.0 | 3.9 | | | |
| --- | --- | --- | --- | --- | --- | --- | --- |
|  | RDC5 0.6 mg/kg po | 10 | 404.4 | 9.1 | -25.7 | -6.0 | 0.009** |
|  | Bupropion 20 mg/kg ip bid | 10 | 429.2 | 5.0 | -0.9 | -0.2 | 0.926 |
|  | Naltrexone 1 mg/kg ip | 10 | 433.0 | 7.8 | 2.9 | 0.7 | 0.760 |
|  | RDC5 0.6 mg/kg po+Bupropion 20 mg/kg ip bid | 10 | 404.6 | 6.8 | -25.5 | -5.9 | 0.009** |
|  | RDC5 0.6 mg/kg po+Naltrexone 1 mg/kg ip | 10 | 415.3 | 4.3 | -14.8 | -3.4 | 0.125 |
|  | Bupropion 20 mg/kg ip bid+Naltrexone 1 mg/kg ip | 10 | 428.3 | 9.8 | -1.8 | -0.4 | 0.852 |
|  | Sibutramine 5 mg/kg po | 10 | 387.8 | 6.3 | -42.2 | -9.8 | <0.001*** |
| Day 38 | Vehicle 4 ml/kg po +2 ml/kg ip | 12 | 433.0 | 3.5 |  |  |  |
|  | RDC5 0.6 mg/kg po | 10 | 409.6 | 7.6 | -23.4 | -5.4 | 0.012* |
|  | Bupropion 20 mg/kg ip bid | 10 | 434.8 | 4.8 | 1.8 | 0.4 | 0.839 |
|  | Naltrexone 1 mg/kg ip | 10 | 436.3 | 7.3 | 3.3 | 0.8 | 0.719 |
|  | RDC5 0.6 mg/kg po+Bupropion 20 mg/kg ip bid | 10 | 410.2 | 7.3 | -22.8 | -5.3 | 0.014* |
|  | RDC5 0.6 mg/kg po+Naltrexone 1 mg/kg ip | 10 | 422.3 | 4.8 | -10.7 | -2.5 | 0.241 |
|  | Bupropion 20 mg/kg ip bid+Naltrexone 1 mg/kg ip | 10 | 432.7 | 8.8 | -0.3 | -0.1 | 0.970 |
|  | Sibutramine 5 mg/kg po | 10 | 392.4 | 7.1 | -40.6 | -9.4 | <0.001*** |
| Day 39 | Vehicle 4 ml/kg po +2 ml/kg ip | 12 | 434.1 | 3.2 |  |  |  |
|  | RDC5 0.6 mg/kg po | 10 | 410.5 | 8.1 | -23.6 | -5.4 | 0.012* |
|  | Bupropion 20 mg/kg ip bid | 10 | 435.4 | 5.0 | 1.3 | 0.3 | 0.892 |
|  | Naltrexone 1 mg/kg ip | 10 | 436.6 | 6.8 | 2.5 | 0.6 | 0.789 |
|  | RDC5 0.6 mg/kg po+Bupropion 20 mg/kg ip bid | 10 | 410.2 | 7.5 | -24.0 | -5.5 | 0.011* |
|  | RDC5 0.6 mg/kg po+Naltrexone 1 mg/kg ip | 10 | 423.2 | 5.5 | -10.9 | -2.5 | 0.241 |
|  | Bupropion 20 mg/kg ip bid+Naltrexone 1 mg/kg ip | 10 | 432.0 | 8.8 | -2.1 | -0.5 | 0.818 |
|  | Sibutramine 5 mg/kg po | 10 | 389.3 | 7.4 | -44.8 | -10.3 | <0.001*** |
| Day 40 | Vehicle 4 ml/kg po +2 ml/kg ip | 12 | 433.6 | 3.3 |  |  |  |
|  | RDC5 0.6 mg/kg po | 10 | 411.5 | 7.3 | -22.2 | -5.1 | 0.020* |
|  | Bupropion 20 mg/kg ip bid | 10 | 432.4 | 5.6 | -1.2 | -0.3 | 0.901 |
|  | Naltrexone 1 mg/kg ip | 10 | 436.6 | 6.6 | 3.0 | 0.7 | 0.748 |
|  | RDC5 0.6 mg/kg po+Bupropion 20 mg/kg ip bid | 10 | 408.2 | 8.1 | -25.4 | -5.8 | 0.008** |
|  | RDC5 0.6 mg/kg po+Naltrexone 1 mg/kg ip | 10 | 424.3 | 5.8 | -9.3 | -2.2 | 0.321 |
|  | Bupropion 20 mg/kg ip bid+Naltrexone 1 mg/kg ip | 10 | 427.9 | 8.7 | -5.7 | -1.3 | 0.546 |
|  | Sibutramine 5 mg/kg po | 10 | 388.2 | 7.8 | -45.4 | -10.5 | <0.001*** |
| Day 41 | Vehicle 4 ml/kg po +2 ml/kg ip | 12 | 433.9 | 3.0 |  |  |  |
|  | RDC5 0.6 mg/kg po | 10 | 414.5 | 7.0 | -19.5 | -4.5 | 0.035* |
|  | Bupropion 20 mg/kg ip bid | 10 | 434.5 | 5.9 | 0.5 | 0.1 | 0.954 |
|  | Naltrexone 1 mg/kg ip | 10 | 440.0 | 6.7 | 6.1 | 1.4 | 0.503 |
|  | RDC5 0.6 mg/kg po+Bupropion 20 mg/kg ip bid | 10 | 407.0 | 7.6 | -26.9 | -6.2 | 0.004** |
|  | RDC5 0.6 mg/kg po+Naltrexone 1 mg/kg ip | 10 | 425.9 | 5.6 | -8.0 | -1.9 | 0.379 |
|  | Bupropion 20 mg/kg ip bid+Naltrexone 1 mg/kg ip | 10 | 429.8 | 8.4 | -4.1 | -1.0 | 0.650 |
|  | Sibutramine 5 mg/kg po | 10 | 391.5 | 7.5 | -42.5 | -9.8 | <0.001*** |
| Day 42 | Vehicle 4 ml/kg po +2 ml/kg ip | 12 | 435.7 | 2.6 |  |  |  |
|  | RDC5 0.6 mg/kg po | 10 | 415.5 | 6.7 | -20.2 | -4.6 | 0.027* |
|  | Bupropion 20 mg/kg ip bid | 10 | 433.7 | 6.2 | -1.9 | -0.4 | 0.829 |
|  | Naltrexone 1 mg/kg ip | 10 | 440.3 | 6.2 | 4.6 | 1.1 | 0.606 |
|  | RDC5 0.6 mg/kg po+Bupropion 20 mg/kg ip bid | 10 | 406.3 | 7.4 | -29.4 | -6.7 | 0.002** |
|  | RDC5 0.6 mg/kg po+Naltrexone 1 mg/kg ip | 10 | 427.6 | 5.3 | -8.1 | -1.8 | 0.371 |
|  | Bupropion 20 mg/kg ip bid+Naltrexone 1 mg/kg ip | 10 | 431.6 | 8.7 | -4.1 | -0.9 | 0.647 |
|  | Sibutramine 5 mg/kg po | 10 | 392.1 | 7.8 | -43.6 | -10.0 | <0.001*** |
| Day 43 | Vehicle 4 ml/kg po +2 ml/kg ip | 12 | 438.4 | 3.0 |  |  |  |
|  | RDC5 0.6 mg/kg po | 10 | 417.6 | 7.0 | -20.8 | -4.8 | 0.023* |
|  | Bupropion 20 mg/kg ip bid | 10 | 433.7 | 6.8 | -4.7 | -1.1 | 0.604 |
|  | Naltrexone 1 mg/kg ip | 10 | 441.9 | 5.7 | 3.5 | 0.8 | 0.698 |
|  | RDC5 0.6 mg/kg po+Bupropion 20 mg/kg ip bid | 10 | 408.4 | 7.1 | -30.1 | -6.9 | 0.001** |
|  | RDC5 0.6 mg/kg po+Naltrexone 1 mg/kg ip | 10 | 430.4 | 6.1 | -8.0 | -1.8 | 0.374 |
|  | Bupropion 20 mg/kg ip bid+Naltrexone 1 mg/kg ip | 10 | 432.0 | 8.2 | -6.4 | -1.5 | 0.479 |
|  | Sibutramine 5 mg/kg po | 10 | 392.7 | 7.3 | -45.7 | -10.4 | <0.001*** |

Means are adjusted for differences between the treatment groups at baseline (Day 1). SEM are calculated from the residuals of the statistical model. Multiple comparisons against vehicle are by the multiple t test. *p<0.05, **p<0.01, ***p<0.001.

| Time | Comparison | Difference | p |
| --- | --- | --- | --- |
| Day -6 | RDC5 (0.6 mg/kg po)+Bupropion (20 mg/kg ip qd) vs RDC5 | -0.4 | 0.937 |
|  | RDC5 (0.6 mg/kg po)+Naltrexone (1 mg/kg ip) vs RDC5 | -1.1 | 0.818 |
|  | RDC5 (0.6 mg/kg po)+Bupropion (20 mg/kg ip qd) vs Bupropion | -2.4 | 0.605 |
|  | Bupropion (20 mg/kg ip qd)+Naltrexone (1 mg/kg ip) vs Bupropion | 0.5 | 0.912 |
|  | RDC5 (0.6 mg/kg po)+Naltrexone (1 mg/kg ip) vs Naltrexone | -2.2 | 0.645 |
|  | Bupropion (20 mg/kg ip qd)+Naltrexone (1 mg/kg ip) vs Naltrexone | 1.5 | 0.751 |
| Day -5 | RDC5 (0.6 mg/kg po)+Bupropion (20 mg/kg ip qd) vs RDC5 | -1.4 | 0.731 |
|  | RDC5 (0.6 mg/kg po)+Naltrexone (1 mg/kg ip) vs RDC5 | 0.4 | 0.918 |
|  | RDC5 (0.6 mg/kg po)+Bupropion (20 mg/kg ip qd) vs Bupropion | -4.4 | 0.283 |
|  | Bupropion (20 mg/kg ip qd)+Naltrexone (1 mg/kg ip) vs Bupropion | -0.7 | 0.861 |
|  | RDC5 (0.6 mg/kg po)+Naltrexone (1 mg/kg ip) vs Naltrexone | -5.3 | 0.197 |
|  | Bupropion (20 mg/kg ip qd)+Naltrexone (1 mg/kg ip) vs Naltrexone | -3.4 | 0.401 |
| Day -4 | RDC5 (0.6 mg/kg po)+Bupropion (20 mg/kg ip qd) vs RDC5 | -2.1 | 0.580 |
|  | RDC5 (0.6 mg/kg po)+Naltrexone (1 mg/kg ip) vs RDC5 | -0.7 | 0.844 |
|  | RDC5 (0.6 mg/kg po)+Bupropion (20 mg/kg ip qd) vs Bupropion | -5.3 | 0.164 |
|  | Bupropion (20 mg/kg ip qd)+Naltrexone (1 mg/kg ip) vs Bupropion | -2.0 | 0.598 |
|  | RDC5 (0.6 mg/kg po)+Naltrexone (1 mg/kg ip) vs Naltrexone | -5.0 | 0.188 |
|  | Bupropion (20 mg/kg ip qd)+Naltrexone (1 mg/kg ip) vs Naltrexone | -3.0 | 0.420 |
| Day -3 | RDC5 (0.6 mg/kg po)+Bupropion (20 mg/kg ip qd) vs RDC5 | -1.0 | 0.752 |
|  | RDC5 (0.6 mg/kg po)+Naltrexone (1 mg/kg ip) vs RDC5 | -1.9 | 0.551 |
|  | RDC5 (0.6 mg/kg po)+Bupropion (20 mg/kg ip qd) vs Bupropion | -3.8 | 0.235 |
|  | Bupropion (20 mg/kg ip qd)+Naltrexone (1 mg/kg ip) vs Bupropion | -2.3 | 0.471 |
|  | RDC5 (0.6 mg/kg po)+Naltrexone (1 mg/kg ip) vs Naltrexone | -3.6 | 0.264 |
|  | Bupropion (20 mg/kg ip qd)+Naltrexone (1 mg/kg ip) vs Naltrexone | -1.2 | 0.712 |
| Day -2 | RDC5 (0.6 mg/kg po)+Bupropion (20 mg/kg ip qd) vs RDC5 | -0.4 | 0.883 |
|  | RDC5 (0.6 mg/kg po)+Naltrexone (1 mg/kg ip) vs RDC5 | -0.9 | 0.744 |
|  | RDC5 (0.6 mg/kg po)+Bupropion (20 mg/kg ip qd) vs Bupropion | -0.2 | 0.953 |
|  | Bupropion (20 mg/kg ip qd)+Naltrexone (1 mg/kg ip) vs Bupropion | -0.3 | 0.903 |
|  | RDC5 (0.6 mg/kg po)+Naltrexone (1 mg/kg ip) vs Naltrexone | -1.0 | 0.738 |
|  | Bupropion (20 mg/kg ip qd)+Naltrexone (1 mg/kg ip) vs Naltrexone | -0.6 | 0.827 |
| Day -1 | RDC5 (0.6 mg/kg po)+Bupropion (20 mg/kg ip qd) vs RDC5 | -0.8 | 0.764 |
|  | RDC5 (0.6 mg/kg po)+Naltrexone (1 mg/kg ip) vs RDC5 | -1.5 | 0.575 |
|  | RDC5 (0.6 mg/kg po)+Bupropion (20 mg/kg ip qd) vs Bupropion | 1.1 | 0.665 |
|  | Bupropion (20 mg/kg ip qd)+Naltrexone (1 mg/kg ip) vs Bupropion | 1.2 | 0.648 |
|  | RDC5 (0.6 mg/kg po)+Naltrexone (1 mg/kg ip) vs Naltrexone | 0.3 | 0.901 |
|  | Bupropion (20 mg/kg ip qd)+Naltrexone (1 mg/kg ip) vs Naltrexone | 1.1 | 0.683 |
| Day 0 | RDC5 (0.6 mg/kg po)+Bupropion (20 mg/kg ip qd) vs RDC5 | 0.8 | 0.697 |
|  | RDC5 (0.6 mg/kg po)+Naltrexone (1 mg/kg ip) vs RDC5 | 0.1 | 0.941 |
|  | RDC5 (0.6 mg/kg po)+Bupropion (20 mg/kg ip qd) vs Bupropion | 1.8 | 0.368 |
|  | Bupropion (20 mg/kg ip qd)+Naltrexone (1 mg/kg ip) vs Bupropion | 1.3 | 0.501 |
|  | RDC5 (0.6 mg/kg po)+Naltrexone (1 mg/kg ip) vs Naltrexone | -3.1 | 0.124 |
|  | Bupropion (20 mg/kg ip qd)+Naltrexone (1 mg/kg ip) vs Naltrexone | -2.9 | 0.146 |

| Time | Comparison | Difference | p |
| --- | --- | --- | --- |
| Day 2 | RDC5 (0.6 mg/kg po)+Bupropion (20 mg/kg ip qd) vs RDC5 | -0.2 | 0.901 |
|  | RDC5 (0.6 mg/kg po)+Naltrexone (1 mg/kg ip) vs RDC5 | -1.3 | 0.481 |
|  | RDC5 (0.6 mg/kg po)+Bupropion (20 mg/kg ip qd) vs Bupropion | 1.2 | 0.538 |
|  | Bupropion (20 mg/kg ip qd)+Naltrexone (1 mg/kg ip) vs Bupropion | -0.6 | 0.756 |
|  | RDC5 (0.6 mg/kg po)+Naltrexone (1 mg/kg ip) vs Naltrexone | -0.3 | 0.878 |
|  | Bupropion (20 mg/kg ip qd)+Naltrexone (1 mg/kg ip) vs Naltrexone | -1.0 | 0.618 |
| Day 3 | RDC5 (0.6 mg/kg po)+Bupropion (20 mg/kg ip qd) vs RDC5 | 0.8 | 0.759 |
|  | RDC5 (0.6 mg/kg po)+Naltrexone (1 mg/kg ip) vs RDC5 | -1.7 | 0.507 |
|  | RDC5 (0.6 mg/kg po)+Bupropion (20 mg/kg ip qd) vs Bupropion | 1.2 | 0.639 |
|  | Bupropion (20 mg/kg ip qd)+Naltrexone (1 mg/kg ip) vs Bupropion | -2.4 | 0.340 |
|  | RDC5 (0.6 mg/kg po)+Naltrexone (1 mg/kg ip) vs Naltrexone | -2.0 | 0.432 |
|  | Bupropion (20 mg/kg ip qd)+Naltrexone (1 mg/kg ip) vs Naltrexone | -3.1 | 0.217 |
| Day 4 | RDC5 (0.6 mg/kg po)+Bupropion (20 mg/kg ip qd) vs RDC5 | 2.6 | 0.369 |
|  | RDC5 (0.6 mg/kg po)+Naltrexone (1 mg/kg ip) vs RDC5 | 0.9 | 0.762 |
|  | RDC5 (0.6 mg/kg po)+Bupropion (20 mg/kg ip qd) vs Bupropion | 3.3 | 0.265 |
|  | Bupropion (20 mg/kg ip qd)+Naltrexone (1 mg/kg ip) vs Bupropion | -0.3 | 0.909 |
|  | RDC5 (0.6 mg/kg po)+Naltrexone (1 mg/kg ip) vs Naltrexone | -1.3 | 0.670 |
|  | Bupropion (20 mg/kg ip qd)+Naltrexone (1 mg/kg ip) vs Naltrexone | -3.1 | 0.290 |
| Day 5 | RDC5 (0.6 mg/kg po)+Bupropion (20 mg/kg ip qd) vs RDC5 | 1.8 | 0.564 |
|  | RDC5 (0.6 mg/kg po)+Naltrexone (1 mg/kg ip) vs RDC5 | -0.5 | 0.858 |
|  | RDC5 (0.6 mg/kg po)+Bupropion (20 mg/kg ip qd) vs Bupropion | 4.3 | 0.164 |
|  | Bupropion (20 mg/kg ip qd)+Naltrexone (1 mg/kg ip) vs Bupropion | 3.2 | 0.298 |
|  | RDC5 (0.6 mg/kg po)+Naltrexone (1 mg/kg ip) vs Naltrexone | -0.6 | 0.838 |
|  | Bupropion (20 mg/kg ip qd)+Naltrexone (1 mg/kg ip) vs Naltrexone | 0.6 | 0.846 |
| Day 6 | RDC5 (0.6 mg/kg po)+Bupropion (20 mg/kg ip qd) vs RDC5 | 2.4 | 0.503 |
|  | RDC5 (0.6 mg/kg po)+Naltrexone (1 mg/kg ip) vs RDC5 | 2.9 | 0.402 |
|  | RDC5 (0.6 mg/kg po)+Bupropion (20 mg/kg ip qd) vs Bupropion | 1.5 | 0.660 |
|  | Bupropion (20 mg/kg ip qd)+Naltrexone (1 mg/kg ip) vs Bupropion | 3.7 | 0.292 |
|  | RDC5 (0.6 mg/kg po)+Naltrexone (1 mg/kg ip) vs Naltrexone | 1.8 | 0.605 |
|  | Bupropion (20 mg/kg ip qd)+Naltrexone (1 mg/kg ip) vs Naltrexone | 3.4 | 0.335 |
| Day 7 | RDC5 (0.6 mg/kg po)+Bupropion (20 mg/kg ip qd) vs RDC5 | 3.3 | 0.438 |
|  | RDC5 (0.6 mg/kg po)+Naltrexone (1 mg/kg ip) vs RDC5 | 4.5 | 0.289 |
|  | RDC5 (0.6 mg/kg po)+Bupropion (20 mg/kg ip qd) vs Bupropion | -0.5 | 0.897 |
|  | Bupropion (20 mg/kg ip qd)+Naltrexone (1 mg/kg ip) vs Bupropion | 3.4 | 0.425 |
|  | RDC5 (0.6 mg/kg po)+Naltrexone (1 mg/kg ip) vs Naltrexone | -0.0 | 0.992 |
|  | Bupropion (20 mg/kg ip qd)+Naltrexone (1 mg/kg ip) vs Naltrexone | 2.7 | 0.527 |
| Day 8 | RDC5 (0.6 mg/kg po)+Bupropion (20 mg/kg ip qd) vs RDC5 | 3.8 | 0.419 |
|  | RDC5 (0.6 mg/kg po)+Naltrexone (1 mg/kg ip) vs RDC5 | 4.8 | 0.301 |
|  | RDC5 (0.6 mg/kg po)+Bupropion (20 mg/kg ip qd) vs Bupropion | -2.0 | 0.660 |
|  | Bupropion (20 mg/kg ip qd)+Naltrexone (1 mg/kg ip) vs Bupropion | 3.8 | 0.419 |
|  | RDC5 (0.6 mg/kg po)+Naltrexone (1 mg/kg ip) vs Naltrexone | -3.8 | 0.412 |
|  | Bupropion (20 mg/kg ip qd)+Naltrexone (1 mg/kg ip) vs Naltrexone | 0.9 | 0.843 |

| Time | Comparison | Difference | p |
| --- | --- | --- | --- |
| Day 9 | RDC5 (0.6 mg/kg po)+Bupropion (20 mg/kg ip qd) vs RDC5 | 3.1 | 0.509 |
|  | RDC5 (0.6 mg/kg po)+Naltrexone (1 mg/kg ip) vs RDC5 | 6.2 | 0.190 |
|  | RDC5 (0.6 mg/kg po)+Bupropion (20 mg/kg ip qd) vs Bupropion | -3.6 | 0.450 |
|  | Bupropion (20 mg/kg ip qd)+Naltrexone (1 mg/kg ip) vs Bupropion | 5.8 | 0.218 |
|  | RDC5 (0.6 mg/kg po)+Naltrexone (1 mg/kg ip) vs Naltrexone | -5.0 | 0.289 |
|  | Bupropion (20 mg/kg ip qd)+Naltrexone (1 mg/kg ip) vs Naltrexone | 1.3 | 0.784 |
| Day 10 | RDC5 (0.6 mg/kg po)+Bupropion (20 mg/kg ip qd) vs RDC5 | 7.0 | 0.183 |
|  | RDC5 (0.6 mg/kg po)+Naltrexone (1 mg/kg ip) vs RDC5 | 10.0 | 0.057 |
|  | RDC5 (0.6 mg/kg po)+Bupropion (20 mg/kg ip qd) vs Bupropion | -4.4 | 0.395 |
|  | Bupropion (20 mg/kg ip qd)+Naltrexone (1 mg/kg ip) vs Bupropion | 6.0 | 0.249 |
|  | RDC5 (0.6 mg/kg po)+Naltrexone (1 mg/kg ip) vs Naltrexone | -4.1 | 0.426 |
|  | Bupropion (20 mg/kg ip qd)+Naltrexone (1 mg/kg ip) vs Naltrexone | 3.2 | 0.533 |
| Day 11 | RDC5 (0.6 mg/kg po)+Bupropion (20 mg/kg ip qd) vs RDC5 | 5.5 | 0.344 |
|  | RDC5 (0.6 mg/kg po)+Naltrexone (1 mg/kg ip) vs RDC5 | 11.7 | 0.049* |
|  | RDC5 (0.6 mg/kg po)+Bupropion (20 mg/kg ip qd) vs Bupropion | -5.5 | 0.346 |
|  | Bupropion (20 mg/kg ip qd)+Naltrexone (1 mg/kg ip) vs Bupropion | 8.6 | 0.142 |
|  | RDC5 (0.6 mg/kg po)+Naltrexone (1 mg/kg ip) vs Naltrexone | -4.8 | 0.414 |
|  | Bupropion (20 mg/kg ip qd)+Naltrexone (1 mg/kg ip) vs Naltrexone | 3.2 | 0.579 |
| Day 12 | RDC5 (0.6 mg/kg po)+Bupropion (20 mg/kg ip qd) vs RDC5 | 4.5 | 0.465 |
|  | RDC5 (0.6 mg/kg po)+Naltrexone (1 mg/kg ip) vs RDC5 | 10.5 | 0.094 |
|  | RDC5 (0.6 mg/kg po)+Bupropion (20 mg/kg ip qd) vs Bupropion | -9.6 | 0.125 |
|  | Bupropion (20 mg/kg ip qd)+Naltrexone (1 mg/kg ip) vs Bupropion | 7.5 | 0.228 |
|  | RDC5 (0.6 mg/kg po)+Naltrexone (1 mg/kg ip) vs Naltrexone | -8.7 | 0.164 |
|  | Bupropion (20 mg/kg ip qd)+Naltrexone (1 mg/kg ip) vs Naltrexone | 2.5 | 0.692 |
| Day 13 | RDC5 (0.6 mg/kg po)+Bupropion (20 mg/kg ip qd) vs RDC5 | 4.4 | 0.505 |
|  | RDC5 (0.6 mg/kg po)+Naltrexone (1 mg/kg ip) vs RDC5 | 9.4 | 0.152 |
|  | RDC5 (0.6 mg/kg po)+Bupropion (20 mg/kg ip qd) vs Bupropion | -10.7 | 0.106 |
|  | Bupropion (20 mg/kg ip qd)+Naltrexone (1 mg/kg ip) vs Bupropion | 11.3 | 0.088 |
|  | RDC5 (0.6 mg/kg po)+Naltrexone (1 mg/kg ip) vs Naltrexone | -8.4 | 0.203 |
|  | Bupropion (20 mg/kg ip qd)+Naltrexone (1 mg/kg ip) vs Naltrexone | 8.5 | 0.195 |
| Day 14 | RDC5 (0.6 mg/kg po)+Bupropion (20 mg/kg ip qd) vs RDC5 | 4.6 | 0.496 |
|  | RDC5 (0.6 mg/kg po)+Naltrexone (1 mg/kg ip) vs RDC5 | 10.8 | 0.113 |
|  | RDC5 (0.6 mg/kg po)+Bupropion (20 mg/kg ip qd) vs Bupropion | -10.8 | 0.113 |
|  | Bupropion (20 mg/kg ip qd)+Naltrexone (1 mg/kg ip) vs Bupropion | 9.4 | 0.168 |
|  | RDC5 (0.6 mg/kg po)+Naltrexone (1 mg/kg ip) vs Naltrexone | -6.4 | 0.342 |
|  | Bupropion (20 mg/kg ip qd)+Naltrexone (1 mg/kg ip) vs Naltrexone | 7.5 | 0.267 |
| Day 15 | RDC5 (0.6 mg/kg po)+Bupropion (20 mg/kg ip qd) vs RDC5 | 6.5 | 0.369 |
|  | RDC5 (0.6 mg/kg po)+Naltrexone (1 mg/kg ip) vs RDC5 | 14.0 | 0.055 |
|  | RDC5 (0.6 mg/kg po)+Bupropion (20 mg/kg ip qd) vs Bupropion | -11.9 | 0.103 |
|  | Bupropion (20 mg/kg ip qd)+Naltrexone (1 mg/kg ip) vs Bupropion | 8.8 | 0.227 |
|  | RDC5 (0.6 mg/kg po)+Naltrexone (1 mg/kg ip) vs Naltrexone | -8.8 | 0.226 |
|  | Bupropion (20 mg/kg ip qd)+Naltrexone (1 mg/kg ip) vs Naltrexone | 4.4 | 0.547 |

| Time | Comparison | Difference | p |
| --- | --- | --- | --- |
| Day 16 | RDC5 (0.6 mg/kg po)+Bupropion (20 mg/kg ip qd) vs RDC5 | 5.2 | 0.470 |
|  | RDC5 (0.6 mg/kg po)+Naltrexone (1 mg/kg ip) vs RDC5 | 11.4 | 0.115 |
|  | RDC5 (0.6 mg/kg po)+Bupropion (20 mg/kg ip qd) vs Bupropion | -13.5 | 0.062 |
|  | Bupropion (20 mg/kg ip qd)+Naltrexone (1 mg/kg ip) vs Bupropion | 6.8 | 0.343 |
|  | RDC5 (0.6 mg/kg po)+Naltrexone (1 mg/kg ip) vs Naltrexone | -13.7 | 0.059 |
|  | Bupropion (20 mg/kg ip qd)+Naltrexone (1 mg/kg ip) vs Naltrexone | 0.4 | 0.953 |
| Day 17 | RDC5 (0.6 mg/kg po)+Bupropion (20 mg/kg ip qd) vs RDC5 | 4.2 | 0.584 |
|  | RDC5 (0.6 mg/kg po)+Naltrexone (1 mg/kg ip) vs RDC5 | 9.5 | 0.213 |
|  | RDC5 (0.6 mg/kg po)+Bupropion (20 mg/kg ip qd) vs Bupropion | -12.8 | 0.095 |
|  | Bupropion (20 mg/kg ip qd)+Naltrexone (1 mg/kg ip) vs Bupropion | 9.0 | 0.241 |
|  | RDC5 (0.6 mg/kg po)+Naltrexone (1 mg/kg ip) vs Naltrexone | -15.3 | 0.047* |
|  | Bupropion (20 mg/kg ip qd)+Naltrexone (1 mg/kg ip) vs Naltrexone | 1.1 | 0.880 |
| Day 18 | RDC5 (0.6 mg/kg po)+Bupropion (20 mg/kg ip qd) vs RDC5 | 8.3 | 0.309 |
|  | RDC5 (0.6 mg/kg po)+Naltrexone (1 mg/kg ip) vs RDC5 | 13.5 | 0.100 |
|  | RDC5 (0.6 mg/kg po)+Bupropion (20 mg/kg ip qd) vs Bupropion | -14.4 | 0.081 |
|  | Bupropion (20 mg/kg ip qd)+Naltrexone (1 mg/kg ip) vs Bupropion | 10.2 | 0.214 |
|  | RDC5 (0.6 mg/kg po)+Naltrexone (1 mg/kg ip) vs Naltrexone | -13.3 | 0.106 |
|  | Bupropion (20 mg/kg ip qd)+Naltrexone (1 mg/kg ip) vs Naltrexone | 6.0 | 0.459 |
| Day 19 | RDC5 (0.6 mg/kg po)+Bupropion (20 mg/kg ip qd) vs RDC5 | 5.7 | 0.478 |
|  | RDC5 (0.6 mg/kg po)+Naltrexone (1 mg/kg ip) vs RDC5 | 15.8 | 0.054 |
|  | RDC5 (0.6 mg/kg po)+Bupropion (20 mg/kg ip qd) vs Bupropion | -17.3 | 0.035* |
|  | Bupropion (20 mg/kg ip qd)+Naltrexone (1 mg/kg ip) vs Bupropion | 7.9 | 0.331 |
|  | RDC5 (0.6 mg/kg po)+Naltrexone (1 mg/kg ip) vs Naltrexone | -10.3 | 0.204 |
|  | Bupropion (20 mg/kg ip qd)+Naltrexone (1 mg/kg ip) vs Naltrexone | 4.9 | 0.548 |
| Day 20 | RDC5 (0.6 mg/kg po)+Bupropion (20 mg/kg ip qd) vs RDC5 | 3.9 | 0.635 |
|  | RDC5 (0.6 mg/kg po)+Naltrexone (1 mg/kg ip) vs RDC5 | 12.1 | 0.145 |
|  | RDC5 (0.6 mg/kg po)+Bupropion (20 mg/kg ip qd) vs Bupropion | -20.2 | 0.016* |
|  | Bupropion (20 mg/kg ip qd)+Naltrexone (1 mg/kg ip) vs Bupropion | 5.8 | 0.482 |
|  | RDC5 (0.6 mg/kg po)+Naltrexone (1 mg/kg ip) vs Naltrexone | -15.6 | 0.062 |
|  | Bupropion (20 mg/kg ip qd)+Naltrexone (1 mg/kg ip) vs Naltrexone | 2.3 | 0.784 |
| Day 21 | RDC5 (0.6 mg/kg po)+Bupropion (20 mg/kg ip qd) vs RDC5 | 3.7 | 0.657 |
|  | RDC5 (0.6 mg/kg po)+Naltrexone (1 mg/kg ip) vs RDC5 | 10.8 | 0.194 |
|  | RDC5 (0.6 mg/kg po)+Bupropion (20 mg/kg ip qd) vs Bupropion | -19.2 | 0.022* |
|  | Bupropion (20 mg/kg ip qd)+Naltrexone (1 mg/kg ip) vs Bupropion | 6.5 | 0.429 |
|  | RDC5 (0.6 mg/kg po)+Naltrexone (1 mg/kg ip) vs Naltrexone | -16.9 | 0.044* |
|  | Bupropion (20 mg/kg ip qd)+Naltrexone (1 mg/kg ip) vs Naltrexone | 1.8 | 0.831 |
| Day 22 | RDC5 (0.6 mg/kg po)+Bupropion (20 mg/kg ip qd) vs RDC5 | 4.1 | 0.632 |
|  | RDC5 (0.6 mg/kg po)+Naltrexone (1 mg/kg ip) vs RDC5 | 12.4 | 0.152 |
|  | RDC5 (0.6 mg/kg po)+Bupropion (20 mg/kg ip qd) vs Bupropion | -17.6 | 0.043* |
|  | Bupropion (20 mg/kg ip qd)+Naltrexone (1 mg/kg ip) vs Bupropion | 7.1 | 0.407 |
|  | RDC5 (0.6 mg/kg po)+Naltrexone (1 mg/kg ip) vs Naltrexone | -16.2 | 0.063 |
|  | Bupropion (20 mg/kg ip qd)+Naltrexone (1 mg/kg ip) vs Naltrexone | 0.3 | 0.968 |

| Time | Comparison | Difference | p |
| --- | --- | --- | --- |
| Day 23 | RDC5 (0.6 mg/kg po)+Bupropion (20 mg/kg ip qd) vs RDC5 | 4.6 | 0.598 |
|  | RDC5 (0.6 mg/kg po)+Naltrexone (1 mg/kg ip) vs RDC5 | 12.7 | 0.145 |
|  | RDC5 (0.6 mg/kg po)+Bupropion (20 mg/kg ip qd) vs Bupropion | -17.0 | 0.052 |
|  | Bupropion (20 mg/kg ip qd)+Naltrexone (1 mg/kg ip) vs Bupropion | 6.2 | 0.477 |
|  | RDC5 (0.6 mg/kg po)+Naltrexone (1 mg/kg ip) vs Naltrexone | -15.5 | 0.076 |
|  | Bupropion (20 mg/kg ip qd)+Naltrexone (1 mg/kg ip) vs Naltrexone | -0.5 | 0.955 |
| Day 24 | RDC5 (0.6 mg/kg po)+Bupropion (20 mg/kg ip qd) vs RDC5 | 5.3 | 0.549 |
|  | RDC5 (0.6 mg/kg po)+Naltrexone (1 mg/kg ip) vs RDC5 | 13.4 | 0.130 |
|  | RDC5 (0.6 mg/kg po)+Bupropion (20 mg/kg ip qd) vs Bupropion | -18.6 | 0.036* |
|  | Bupropion (20 mg/kg ip qd)+Naltrexone (1 mg/kg ip) vs Bupropion | 4.9 | 0.577 |
|  | RDC5 (0.6 mg/kg po)+Naltrexone (1 mg/kg ip) vs Naltrexone | -17.3 | 0.051 |
|  | Bupropion (20 mg/kg ip qd)+Naltrexone (1 mg/kg ip) vs Naltrexone | -1.9 | 0.832 |
| Day 25 | RDC5 (0.6 mg/kg po)+Bupropion (20 mg/kg ip qd) vs RDC5 | 3.9 | 0.657 |
|  | RDC5 (0.6 mg/kg po)+Naltrexone (1 mg/kg ip) vs RDC5 | 11.5 | 0.199 |
|  | RDC5 (0.6 mg/kg po)+Bupropion (20 mg/kg ip qd) vs Bupropion | -21.1 | 0.020* |
|  | Bupropion (20 mg/kg ip qd)+Naltrexone (1 mg/kg ip) vs Bupropion | 6.2 | 0.484 |
|  | RDC5 (0.6 mg/kg po)+Naltrexone (1 mg/kg ip) vs Naltrexone | -17.9 | 0.046* |
|  | Bupropion (20 mg/kg ip qd)+Naltrexone (1 mg/kg ip) vs Naltrexone | 1.9 | 0.832 |
| Day 26 | RDC5 (0.6 mg/kg po)+Bupropion (20 mg/kg ip bid) vs RDC5 | 4.9 | 0.602 |
|  | RDC5 (0.6 mg/kg po)+Naltrexone (1 mg/kg ip) vs RDC5 | 11.1 | 0.238 |
|  | RDC5 (0.6 mg/kg po)+Bupropion (20 mg/kg ip bid) vs Bupropion | -19.6 | 0.039* |
|  | Bupropion (20 mg/kg ip bid)+Naltrexone (1 mg/kg ip) vs Bupropion | 6.8 | 0.467 |
|  | RDC5 (0.6 mg/kg po)+Naltrexone (1 mg/kg ip) vs Naltrexone | -18.6 | 0.051 |
|  | Bupropion (20 mg/kg ip bid)+Naltrexone (1 mg/kg ip) vs Naltrexone | 1.7 | 0.857 |
| Day 27 | RDC5 (0.6 mg/kg po)+Bupropion (20 mg/kg ip bid) vs RDC5 | 5.0 | 0.600 |
|  | RDC5 (0.6 mg/kg po)+Naltrexone (1 mg/kg ip) vs RDC5 | 12.4 | 0.193 |
|  | RDC5 (0.6 mg/kg po)+Bupropion (20 mg/kg ip bid) vs Bupropion | -21.3 | 0.027* |
|  | Bupropion (20 mg/kg ip bid)+Naltrexone (1 mg/kg ip) vs Bupropion | 2.6 | 0.781 |
|  | RDC5 (0.6 mg/kg po)+Naltrexone (1 mg/kg ip) vs Naltrexone | -19.6 | 0.041* |
|  | Bupropion (20 mg/kg ip bid)+Naltrexone (1 mg/kg ip) vs Naltrexone | -3.2 | 0.739 |
| Day 28 | RDC5 (0.6 mg/kg po)+Bupropion (20 mg/kg ip bid) vs RDC5 | 5.5 | 0.546 |
|  | RDC5 (0.6 mg/kg po)+Naltrexone (1 mg/kg ip) vs RDC5 | 13.3 | 0.146 |
|  | RDC5 (0.6 mg/kg po)+Bupropion (20 mg/kg ip bid) vs Bupropion | -21.3 | 0.022* |
|  | Bupropion (20 mg/kg ip bid)+Naltrexone (1 mg/kg ip) vs Bupropion | 0.7 | 0.935 |
|  | RDC5 (0.6 mg/kg po)+Naltrexone (1 mg/kg ip) vs Naltrexone | -20.7 | 0.026* |
|  | Bupropion (20 mg/kg ip bid)+Naltrexone (1 mg/kg ip) vs Naltrexone | -6.6 | 0.472 |
| Day 29 | RDC5 (0.6 mg/kg po)+Bupropion (20 mg/kg ip bid) vs RDC5 | 4.1 | 0.653 |
|  | RDC5 (0.6 mg/kg po)+Naltrexone (1 mg/kg ip) vs RDC5 | 9.1 | 0.318 |
|  | RDC5 (0.6 mg/kg po)+Bupropion (20 mg/kg ip bid) vs Bupropion | -20.4 | 0.027* |
|  | Bupropion (20 mg/kg ip bid)+Naltrexone (1 mg/kg ip) vs Bupropion | 3.5 | 0.703 |
|  | RDC5 (0.6 mg/kg po)+Naltrexone (1 mg/kg ip) vs Naltrexone | -22.5 | 0.015* |
|  | Bupropion (20 mg/kg ip bid)+Naltrexone (1 mg/kg ip) vs Naltrexone | -3.6 | 0.693 |

Multiple comparisons are by the multiple t test. *p<0.05.

| Time | Comparison | Difference | p |
| --- | --- | --- | --- |
| Day 30 | RDC5 (0.6 mg/kg po)+Bupropion (20 mg/kg ip bid) vs RDC5 | 5.0 | 0.587 |
|  | RDC5 (0.6 mg/kg po)+Naltrexone (1 mg/kg ip) vs RDC5 | 8.8 | 0.340 |
|  | RDC5 (0.6 mg/kg po)+Bupropion (20 mg/kg ip bid) vs Bupropion | -19.2 | 0.039* |
|  | Bupropion (20 mg/kg ip bid)+Naltrexone (1 mg/kg ip) vs Bupropion | 4.5 | 0.624 |
|  | RDC5 (0.6 mg/kg po)+Naltrexone (1 mg/kg ip) vs Naltrexone | -19.7 | 0.034* |
|  | Bupropion (20 mg/kg ip bid)+Naltrexone (1 mg/kg ip) vs Naltrexone | 0.2 | 0.982 |
| Day 31 | RDC5 (0.6 mg/kg po)+Bupropion (20 mg/kg ip bid) vs RDC5 | 3.9 | 0.677 |
|  | RDC5 (0.6 mg/kg po)+Naltrexone (1 mg/kg ip) vs RDC5 | 9.1 | 0.338 |
|  | RDC5 (0.6 mg/kg po)+Bupropion (20 mg/kg ip bid) vs Bupropion | -20.3 | 0.035* |
|  | Bupropion (20 mg/kg ip bid)+Naltrexone (1 mg/kg ip) vs Bupropion | 3.1 | 0.742 |
|  | RDC5 (0.6 mg/kg po)+Naltrexone (1 mg/kg ip) vs Naltrexone | -20.0 | 0.038* |
|  | Bupropion (20 mg/kg ip bid)+Naltrexone (1 mg/kg ip) vs Naltrexone | -1.7 | 0.854 |
| Day 32 | RDC5 (0.6 mg/kg po)+Bupropion (20 mg/kg ip bid) vs RDC5 | 3.3 | 0.735 |
|  | RDC5 (0.6 mg/kg po)+Naltrexone (1 mg/kg ip) vs RDC5 | 12.2 | 0.220 |
|  | RDC5 (0.6 mg/kg po)+Bupropion (20 mg/kg ip bid) vs Bupropion | -21.1 | 0.035* |
|  | Bupropion (20 mg/kg ip bid)+Naltrexone (1 mg/kg ip) vs Bupropion | 5.5 | 0.581 |
|  | RDC5 (0.6 mg/kg po)+Naltrexone (1 mg/kg ip) vs Naltrexone | -19.4 | 0.053 |
|  | Bupropion (20 mg/kg ip bid)+Naltrexone (1 mg/kg ip) vs Naltrexone | -1.7 | 0.865 |
| Day 33 | RDC5 (0.6 mg/kg po)+Bupropion (20 mg/kg ip bid) vs RDC5 | 3.1 | 0.759 |
|  | RDC5 (0.6 mg/kg po)+Naltrexone (1 mg/kg ip) vs RDC5 | 11.7 | 0.248 |
|  | RDC5 (0.6 mg/kg po)+Bupropion (20 mg/kg ip bid) vs Bupropion | -21.3 | 0.037* |
|  | Bupropion (20 mg/kg ip bid)+Naltrexone (1 mg/kg ip) vs Bupropion | 3.1 | 0.755 |
|  | RDC5 (0.6 mg/kg po)+Naltrexone (1 mg/kg ip) vs Naltrexone | -20.5 | 0.045* |
|  | Bupropion (20 mg/kg ip bid)+Naltrexone (1 mg/kg ip) vs Naltrexone | -4.6 | 0.649 |
| Day 34 | RDC5 (0.6 mg/kg po)+Bupropion (20 mg/kg ip bid) vs RDC5 | 1.7 | 0.861 |
|  | RDC5 (0.6 mg/kg po)+Naltrexone (1 mg/kg ip) vs RDC5 | 11.6 | 0.241 |
|  | RDC5 (0.6 mg/kg po)+Bupropion (20 mg/kg ip bid) vs Bupropion | -22.7 | 0.024* |
|  | Bupropion (20 mg/kg ip bid)+Naltrexone (1 mg/kg ip) vs Bupropion | 0.9 | 0.928 |
|  | RDC5 (0.6 mg/kg po)+Naltrexone (1 mg/kg ip) vs Naltrexone | -16.9 | 0.089 |
|  | Bupropion (20 mg/kg ip bid)+Naltrexone (1 mg/kg ip) vs Naltrexone | -3.2 | 0.746 |
| Day 35 | RDC5 (0.6 mg/kg po)+Bupropion (20 mg/kg ip bid) vs RDC5 | 3.7 | 0.710 |
|  | RDC5 (0.6 mg/kg po)+Naltrexone (1 mg/kg ip) vs RDC5 | 12.7 | 0.200 |
|  | RDC5 (0.6 mg/kg po)+Bupropion (20 mg/kg ip bid) vs Bupropion | -20.6 | 0.039* |
|  | Bupropion (20 mg/kg ip bid)+Naltrexone (1 mg/kg ip) vs Bupropion | 2.6 | 0.788 |
|  | RDC5 (0.6 mg/kg po)+Naltrexone (1 mg/kg ip) vs Naltrexone | -16.0 | 0.107 |
|  | Bupropion (20 mg/kg ip bid)+Naltrexone (1 mg/kg ip) vs Naltrexone | -1.7 | 0.861 |
| Day 36 | RDC5 (0.6 mg/kg po)+Bupropion (20 mg/kg ip bid) vs RDC5 | 2.0 | 0.842 |
|  | RDC5 (0.6 mg/kg po)+Naltrexone (1 mg/kg ip) vs RDC5 | 13.0 | 0.204 |
|  | RDC5 (0.6 mg/kg po)+Bupropion (20 mg/kg ip bid) vs Bupropion | -21.4 | 0.038* |
|  | Bupropion (20 mg/kg ip bid)+Naltrexone (1 mg/kg ip) vs Bupropion | 1.4 | 0.887 |
|  | RDC5 (0.6 mg/kg po)+Naltrexone (1 mg/kg ip) vs Naltrexone | -16.6 | 0.107 |
|  | Bupropion (20 mg/kg ip bid)+Naltrexone (1 mg/kg ip) vs Naltrexone | -4.7 | 0.648 |

Multiple comparisons are by the multiple t test. *p<0.05.

| Time | Comparison | Difference | p |
| --- | --- | --- | --- |
| Day 37 | RDC5 (0.6 mg/kg po)+Bupropion (20 mg/kg ip bid) vs RDC5 | 0.2 | 0.986 |
|  | RDC5 (0.6 mg/kg po)+Naltrexone (1 mg/kg ip) vs RDC5 | 10.9 | 0.275 |
|  | RDC5 (0.6 mg/kg po)+Bupropion (20 mg/kg ip bid) vs Bupropion | -24.6 | 0.016* |
|  | Bupropion (20 mg/kg ip bid)+Naltrexone (1 mg/kg ip) vs Bupropion | -0.9 | 0.928 |
|  | RDC5 (0.6 mg/kg po)+Naltrexone (1 mg/kg ip) vs Naltrexone | -17.7 | 0.079 |
|  | Bupropion (20 mg/kg ip bid)+Naltrexone (1 mg/kg ip) vs Naltrexone | -4.7 | 0.637 |
| Day 38 | RDC5 (0.6 mg/kg po)+Bupropion (20 mg/kg ip bid) vs RDC5 | 0.6 | 0.946 |
|  | RDC5 (0.6 mg/kg po)+Naltrexone (1 mg/kg ip) vs RDC5 | 12.7 | 0.182 |
|  | RDC5 (0.6 mg/kg po)+Bupropion (20 mg/kg ip bid) vs Bupropion | -24.6 | 0.011* |
|  | Bupropion (20 mg/kg ip bid)+Naltrexone (1 mg/kg ip) vs Bupropion | -2.2 | 0.818 |
|  | RDC5 (0.6 mg/kg po)+Naltrexone (1 mg/kg ip) vs Naltrexone | -14.0 | 0.144 |
|  | Bupropion (20 mg/kg ip bid)+Naltrexone (1 mg/kg ip) vs Naltrexone | -3.6 | 0.704 |
| Day 39 | RDC5 (0.6 mg/kg po)+Bupropion (20 mg/kg ip bid) vs RDC5 | -0.3 | 0.974 |
|  | RDC5 (0.6 mg/kg po)+Naltrexone (1 mg/kg ip) vs RDC5 | 12.7 | 0.190 |
|  | RDC5 (0.6 mg/kg po)+Bupropion (20 mg/kg ip bid) vs Bupropion | -25.2 | 0.011* |
|  | Bupropion (20 mg/kg ip bid)+Naltrexone (1 mg/kg ip) vs Bupropion | -3.4 | 0.726 |
|  | RDC5 (0.6 mg/kg po)+Naltrexone (1 mg/kg ip) vs Naltrexone | -13.4 | 0.169 |
|  | Bupropion (20 mg/kg ip bid)+Naltrexone (1 mg/kg ip) vs Naltrexone | -4.6 | 0.634 |
| Day 40 | RDC5 (0.6 mg/kg po)+Bupropion (20 mg/kg ip bid) vs RDC5 | -3.2 | 0.743 |
|  | RDC5 (0.6 mg/kg po)+Naltrexone (1 mg/kg ip) vs RDC5 | 12.8 | 0.193 |
|  | RDC5 (0.6 mg/kg po)+Bupropion (20 mg/kg ip bid) vs Bupropion | -24.2 | 0.016* |
|  | Bupropion (20 mg/kg ip bid)+Naltrexone (1 mg/kg ip) vs Bupropion | -4.5 | 0.646 |
|  | RDC5 (0.6 mg/kg po)+Naltrexone (1 mg/kg ip) vs Naltrexone | -12.4 | 0.210 |
|  | Bupropion (20 mg/kg ip bid)+Naltrexone (1 mg/kg ip) vs Naltrexone | -8.7 | 0.377 |
| Day 41 | RDC5 (0.6 mg/kg po)+Bupropion (20 mg/kg ip bid) vs RDC5 | -7.5 | 0.433 |
|  | RDC5 (0.6 mg/kg po)+Naltrexone (1 mg/kg ip) vs RDC5 | 11.4 | 0.231 |
|  | RDC5 (0.6 mg/kg po)+Bupropion (20 mg/kg ip bid) vs Bupropion | -27.5 | 0.005** |
|  | Bupropion (20 mg/kg ip bid)+Naltrexone (1 mg/kg ip) vs Bupropion | -4.7 | 0.624 |
|  | RDC5 (0.6 mg/kg po)+Naltrexone (1 mg/kg ip) vs Naltrexone | -14.1 | 0.140 |
|  | Bupropion (20 mg/kg ip bid)+Naltrexone (1 mg/kg ip) vs Naltrexone | -10.2 | 0.284 |
| Day 42 | RDC5 (0.6 mg/kg po)+Bupropion (20 mg/kg ip bid) vs RDC5 | -9.2 | 0.329 |
|  | RDC5 (0.6 mg/kg po)+Naltrexone (1 mg/kg ip) vs RDC5 | 12.1 | 0.199 |
|  | RDC5 (0.6 mg/kg po)+Bupropion (20 mg/kg ip bid) vs Bupropion | -27.4 | 0.005** |
|  | Bupropion (20 mg/kg ip bid)+Naltrexone (1 mg/kg ip) vs Bupropion | -2.2 | 0.817 |
|  | RDC5 (0.6 mg/kg po)+Naltrexone (1 mg/kg ip) vs Naltrexone | -12.7 | 0.179 |
|  | Bupropion (20 mg/kg ip bid)+Naltrexone (1 mg/kg ip) vs Naltrexone | -8.8 | 0.353 |
| Day 43 | RDC5 (0.6 mg/kg po)+Bupropion (20 mg/kg ip bid) vs RDC5 | -9.2 | 0.328 |
|  | RDC5 (0.6 mg/kg po)+Naltrexone (1 mg/kg ip) vs RDC5 | 12.8 | 0.175 |
|  | RDC5 (0.6 mg/kg po)+Bupropion (20 mg/kg ip bid) vs Bupropion | -25.4 | 0.008** |
|  | Bupropion (20 mg/kg ip bid)+Naltrexone (1 mg/kg ip) vs Bupropion | -1.7 | 0.856 |
|  | RDC5 (0.6 mg/kg po)+Naltrexone (1 mg/kg ip) vs Naltrexone | -11.5 | 0.223 |
|  | Bupropion (20 mg/kg ip bid)+Naltrexone (1 mg/kg ip) vs Naltrexone | -9.9 | 0.295 |

Multiple comparisons are by the multiple t test. *p<0.05. **p<0.01.

#### Changes in body weights (g)

Treatment n Mean SEM Difference p

|  | | | | | from vehicle |  |
| --- | --- | --- | --- | --- | --- | --- |
| Week 1 | Vehicle 4 ml/kg po +2 ml/kg ip | 12 | 6.7 | 2.4 |  |  |
| (Day 1-8) | RDC5 0.6 mg/kg po | 10 | -6.6 | 2.3 | -13.3 | 0.004** |
|  | Bupropion 20 mg/kg ip qd | 10 | -0.8 | 3.0 | -7.5 | 0.096 |
|  | Naltrexone 1 mg/kg ip | 10 | 2.1 | 3.4 | -4.6 | 0.299 |
|  | RDC5 0.6 mg/kg po+Bupropion 20 mg/kg ip qd | 10 | -2.8 | 1.6 | -9.5 | 0.035* |
|  | RDC5 0.6 mg/kg po+Naltrexone 1 mg/kg ip | 10 | -1.8 | 3.7 | -8.5 | 0.060 |
|  | Bupropion 20 mg/kg ip qd+Naltrexone 1 mg/kg ip | 10 | 3.0 | 4.6 | -3.7 | 0.404 |
| Sibutramine 5 mg/kg po 10 | | | -32.7 | 3.8 | -39.4 <0.001*** | |
| Week 2 | Vehicle 4 ml/kg po +2 ml/kg ip | 12 | 3.3 | 2.6 |  |  |
| (Day 8-15) | RDC5 0.6 mg/kg po | 10 | -6.4 | 4.2 | -9.7 | 0.019* |
|  | Bupropion 20 mg/kg ip qd | 10 | 6.2 | 2.4 | 2.9 | 0.484 |
|  | Naltrexone 1 mg/kg ip | 10 | 7.7 | 3.6 | 4.4 | 0.280 |
|  | RDC5 0.6 mg/kg po+Bupropion 20 mg/kg ip qd | 10 | -3.7 | 3.4 | -7.0 | 0.089 |
|  | RDC5 0.6 mg/kg po+Naltrexone 1 mg/kg ip | 10 | 2.7 | 1.9 | -0.6 | 0.892 |
|  | Bupropion 20 mg/kg ip qd+Naltrexone 1 mg/kg ip | 10 | 11.2 | 3.0 | 7.9 | 0.057 |
|  | Sibutramine 5 mg/kg po | 10 | -2.7 | 1.5 | -6.0 | 0.143 |
| Week 3 | Vehicle 4 ml/kg po +2 ml/kg ip | 12 | 5.9 | 3.1 |  |  |
| (Day 15-22) | RDC5 0.6 mg/kg po | 10 | 3.3 | 2.4 | -2.6 | 0.582 |
|  | Bupropion 20 mg/kg ip qd | 10 | 6.7 | 3.9 | 0.8 | 0.866 |
|  | Naltrexone 1 mg/kg ip | 10 | 9.0 | 5.5 | 3.2 | 0.497 |
|  | RDC5 0.6 mg/kg po+Bupropion 20 mg/kg ip qd | 10 | 0.9 | 3.3 | -5.0 | 0.289 |
|  | RDC5 0.6 mg/kg po+Naltrexone 1 mg/kg ip | 10 | 1.7 | 2.4 | -4.2 | 0.367 |
|  | Bupropion 20 mg/kg ip qd+Naltrexone 1 mg/kg ip | 10 | 5.0 | 2.2 | -0.8 | 0.856 |
|  | Sibutramine 5 mg/kg po | 10 | 4.9 | 2.6 | -1.0 | 0.838 |
| Week 4 | Vehicle 4 ml/kg po +2 ml/kg ip | 11 | 0.3 | 1.8 |  |  |
| (Day 22-26) | RDC5 0.6 mg/kg po | 10 | 1.2 | 1.8 | 0.9 | 0.725 |
|  | Bupropion 20 mg/kg ip qd | 10 | 4.0 | 1.5 | 3.7 | 0.167 |
|  | Naltrexone 1 mg/kg ip | 10 | 2.4 | 2.3 | 2.1 | 0.435 |
|  | RDC5 0.6 mg/kg po+Bupropion 20 mg/kg ip qd | 10 | 2.0 | 2.8 | 1.7 | 0.520 |
|  | RDC5 0.6 mg/kg po+Naltrexone 1 mg/kg ip | 10 | -0.0 | 1.5 | -0.3 | 0.910 |
|  | Bupropion 20 mg/kg ip qd +Naltrexone 1 mg/kg ip | 10 | 3.7 | 1.7 | 3.4 | 0.204 |
|  | Sibutramine 5 mg/kg po | 10 | 2.1 | 1.2 | 1.8 | 0.506 |
| Week 5 | Vehicle 4 ml/kg po +2 ml/kg ip | 11 | 2.7 | 1.8 |  |  |
| (Day 26-36) | RDC5 0.6 mg/kg po | 10 | -0.7 | 2.4 | -3.3 | 0.440 |
|  | Bupropion 20 mg/kg ip bid | 10 | -1.8 | 2.7 | -4.4 | 0.305 |
|  | Naltrexone 1 mg/kg ip | 10 | -0.9 | 3.1 | -3.5 | 0.415 |
|  | RDC5 0.6 mg/kg po+Bupropion 20 mg/kg ip bid | 10 | -3.5 | 4.5 | -6.2 | 0.153 |
|  | RDC5 0.6 mg/kg po+Naltrexone 1 mg/kg ip | 10 | 1.2 | 2.8 | -1.5 | 0.733 |
|  | Bupropion 20 mg/kg ip bid+Naltrexone 1 mg/kg ip | 10 | -7.1 | 3.8 | -9.8 | 0.025* |
|  | Sibutramine 5 mg/kg po | 10 | 2.4 | 2.4 | -0.2 | 0.954 |
| Week 6 | Vehicle 4 ml/kg po +2 ml/kg ip | 12 | -0.6 | 3.4 |  |  |
| (Day 36-43) | RDC5 0.6 mg/kg po | 10 | 4.8 | 4.7 | 5.4 | 0.316 |
|  | Bupropion 20 mg/kg ip bid | 10 | -2.5 | 3.7 | -1.9 | 0.728 |
|  | Naltrexone 1 mg/kg ip | 10 | -0.4 | 4.7 | 0.2 | 0.969 |
|  | RDC5 0.6 mg/kg po+Bupropion 20 mg/kg ip bid | 10 | -6.4 | 2.9 | -5.8 | 0.280 |
|  | RDC5 0.6 mg/kg po+Naltrexone 1 mg/kg ip | 10 | 4.7 | 3.2 | 5.2 | 0.331 |
|  | Bupropion 20 mg/kg ip bid+Naltrexone 1 mg/kg ip | 10 | -5.6 | 5.0 | -5.0 | 0.352 |
|  | Sibutramine 5 mg/kg po | 10 | -3.2 | 2.4 | -2.6 | 0.625 |

Means are adjusted for differences between the treatment groups at baseline (Day 1). SEM are calculated from the residuals of the statistical model. Multiple comparisons against vehicle are by the multiple t test. *p<0.05, **p<0.01, ***p<0.001.

| Treatment n | | | Mean | SEM | Difference  from vehicle | p |
| --- | --- | --- | --- | --- | --- | --- |
| Weeks 1-4 Vehicle 4 ml/kg po +2 ml/kg ip 11 | | | 16.6 | 3.8 |  |  |
| (Day 1-26) RDC5 0.6 mg/kg po 10 | | | -8.6 | 7.6 | -25.2 | 0.007** |
| Bupropion 20 mg/kg ip qd | | 10 | 16.0 | 5.6 | -0.6 | 0.944 |
| Naltrexone 1 mg/kg ip | | 10 | 21.1 | 8.5 | 4.5 | 0.623 |
| RDC5 0.6 mg/kg po+Bupropion 20 mg/kg ip qd | | 10 | -3.7 | 7.0 | -20.3 | 0.030* |
| RDC5 0.6 mg/kg po+Naltrexone 1 mg/kg ip | | 10 | 2.6 | 3.1 | -14.0 | 0.129 |
| Bupropion 20 mg/kg ip qd+Naltrexone 1 mg/kg ip | | 10 | 22.8 | 7.4 | 6.2 | 0.499 |
| Sibutramine 5 mg/kg po | | 10 | -28.5 | 7.3 | -45.1 | <0.001*** |
| Weeks 5-6 Vehicle 4 ml/kg po +2 ml/kg ip | | 11 | -0.3 | 3.1 |  |  |
| (Day 26-43) RDC5 0.6 mg/kg po | | 10 | 4.1 | 4.4 | 4.5 | 0.438 |
| Bupropion 20 mg/kg ip bid | | 10 | -4.2 | 3.8 | -3.9 | 0.502 |
| Naltrexone 1 mg/kg ip | | 10 | -1.2 | 5.6 | -0.9 | 0.881 |
| RDC5 0.6 mg/kg po+Bupropion 20 mg/kg ip bid | | 10 | -10.0 | 3.6 | -9.6 | 0.097 |
| RDC5 0.6 mg/kg po+Naltrexone 1 mg/kg ip | | 10 | 5.8 | 4.1 | 6.2 | 0.286 |
| Bupropion 20 mg/kg ip bid+Naltrexone 1 mg/kg ip | | 10 | -12.8 | 4.7 | -12.4 | 0.033* |
| Sibutramine 5 mg/kg po | | 10 | -0.8 | 2.9 | -0.5 | 0.935 |
| Weeks 1-5 Vehicle 4 ml/kg po +2 ml/kg ip | | 12 | 17.1 | 4.5 |  |  |
| (Day 1-36) RDC5 0.6 mg/kg po | | 10 | -9.2 | 8.7 | -26.3 | 0.008** |
| Bupropion 20 mg/kg ip qd/bid | | 10 | 14.3 | 5.3 | -2.8 | 0.773 |
| Naltrexone 1 mg/kg ip | | 10 | 20.4 | 7.4 | 3.3 | 0.736 |
| RDC5 0.6 mg/kg po+Bupropion 20 mg/kg ip qd/bid | | 10 | -7.2 | 8.1 | -24.2 | 0.015* |
| RDC5 0.6 mg/kg po+Naltrexone 1 mg/kg ip | | 10 | 3.8 | 4.7 | -13.3 | 0.175 |
| Bupropion 20 mg/kg ip qd/bid+Naltrexone 1 mg/kg ip | | 10 | 15.7 | 9.6 | -1.4 | 0.888 |
| Sibutramine 5 mg/kg po 10 | | | -26.0 | 6.5 | -43.1 <0.001*** | |
| Overall | Vehicle 4 ml/kg po +2 ml/kg ip | 12 | 16.5 | 3.0 |  |  |
| (Day 1-43) | RDC5 0.6 mg/kg po | 10 | -4.4 | 7.0 | -20.8 | 0.023* |
|  | Bupropion 20 mg/kg ip qd/bid | 10 | 11.8 | 6.8 | -4.7 | 0.604 |
|  | Naltrexone 1 mg/kg ip | 10 | 20.0 | 5.7 | 3.5 | 0.698 |
|  | RDC5 0.6 mg/kg po+Bupropion 20 mg/kg ip qd/bid | 10 | -13.6 | 7.1 | -30.1 | 0.001** |
|  | RDC5 0.6 mg/kg po+Naltrexone 1 mg/kg ip | 10 | 8.4 | 6.1 | -8.0 | 0.374 |
|  | Bupropion 20 mg/kg ip qd/bid+Naltrexone 1 mg/kg ip | 10 | 10.1 | 8.2 | -6.4 | 0.479 |
|  | Sibutramine 5 mg/kg po | 10 | -29.2 | 7.3 | -45.7 | <0.001*** |

Means are adjusted for differences between the treatment groups at baseline (Day 1). SEM are calculated from the residuals of the statistical model. Multiple comparisons against vehicle are by the multiple t test. *p<0.05, **p<0.01, ***p<0.001.

| Time | Comparison | Difference | p |
| --- | --- | --- | --- |
| Week 1 | RDC5 (0.6 mg/kg po)+Bupropion (20 mg/kg ip qd) vs RDC5 | 3.8 | 0.419 |
| (Day 1-8) | RDC5 (0.6 mg/kg po)+Naltrexone (1 mg/kg ip) vs RDC5 | 4.8 | 0.301 |
|  | RDC5 (0.6 mg/kg po)+Bupropion (20 mg/kg ip qd) vs Bupropion | -2.0 | 0.660 |
|  | Bupropion (20 mg/kg ip qd)+Naltrexone (1 mg/kg ip) vs Bupropion | 3.8 | 0.419 |
|  | RDC5 (0.6 mg/kg po)+Naltrexone (1 mg/kg ip) vs Naltrexone | -3.8 | 0.412 |
|  | Bupropion (20 mg/kg ip qd)+Naltrexone (1 mg/kg ip) vs Naltrexone | 0.9 | 0.843 |
| Week 2 | RDC5 (0.6 mg/kg po)+Bupropion (20 mg/kg ip qd) vs RDC5 | 2.7 | 0.519 |
| (Day 8-15) | RDC5 (0.6 mg/kg po)+Naltrexone (1 mg/kg ip) vs RDC5 | 9.2 | 0.033* |
|  | RDC5 (0.6 mg/kg po)+Bupropion (20 mg/kg ip qd) vs Bupropion | -9.8 | 0.023* |
|  | Bupropion (20 mg/kg ip qd)+Naltrexone (1 mg/kg ip) vs Bupropion | 5.0 | 0.241 |
|  | RDC5 (0.6 mg/kg po)+Naltrexone (1 mg/kg ip) vs Naltrexone | -5.0 | 0.245 |
|  | Bupropion (20 mg/kg ip qd)+Naltrexone (1 mg/kg ip) vs Naltrexone | 3.4 | 0.420 |
| Week 3 | RDC5 (0.6 mg/kg po)+Bupropion (20 mg/kg ip qd) vs RDC5 | -2.4 | 0.623 |
| (Day 15-22) | RDC5 (0.6 mg/kg po)+Naltrexone (1 mg/kg ip) vs RDC5 | -1.6 | 0.735 |
|  | RDC5 (0.6 mg/kg po)+Bupropion (20 mg/kg ip qd) vs Bupropion | -5.7 | 0.240 |
|  | Bupropion (20 mg/kg ip qd)+Naltrexone (1 mg/kg ip) vs Bupropion | -1.6 | 0.738 |
|  | RDC5 (0.6 mg/kg po)+Naltrexone (1 mg/kg ip) vs Naltrexone | -7.4 | 0.133 |
|  | Bupropion (20 mg/kg ip qd)+Naltrexone (1 mg/kg ip) vs Naltrexone | -4.0 | 0.411 |
| Week 4 | RDC5 (0.6 mg/kg po)+Bupropion (20 mg/kg ip qd) vs RDC5 | 0.8 | 0.774 |
| (Day 22-26) | RDC5 (0.6 mg/kg po)+Naltrexone (1 mg/kg ip) vs RDC5 | -1.2 | 0.650 |
|  | RDC5 (0.6 mg/kg po)+Bupropion (20 mg/kg ip qd) vs Bupropion | -2.0 | 0.467 |
|  | Bupropion (20 mg/kg ip qd)+Naltrexone (1 mg/kg ip) vs Bupropion | -0.3 | 0.912 |
|  | RDC5 (0.6 mg/kg po)+Naltrexone (1 mg/kg ip) vs Naltrexone | -2.4 | 0.383 |
|  | Bupropion (20 mg/kg ip qd)+Naltrexone (1 mg/kg ip) vs Naltrexone | 1.3 | 0.629 |
| Week 5 | RDC5 (0.6 mg/kg po)+Bupropion (20 mg/kg ip bid) vs RDC5 | -2.9 | 0.516 |
| (Day 26-36) | RDC5 (0.6 mg/kg po)+Naltrexone (1 mg/kg ip) vs RDC5 | 1.9 | 0.672 |
|  | RDC5 (0.6 mg/kg po)+Bupropion (20 mg/kg ip bid) vs Bupropion | -1.8 | 0.690 |
|  | Bupropion (20 mg/kg ip bid)+Naltrexone (1 mg/kg ip) vs Bupropion | -5.4 | 0.226 |
|  | RDC5 (0.6 mg/kg po)+Naltrexone (1 mg/kg ip) vs Naltrexone | 2.0 | 0.642 |
|  | Bupropion (20 mg/kg ip bid)+Naltrexone (1 mg/kg ip) vs Naltrexone | -6.3 | 0.157 |
| Week 6 | RDC5 (0.6 mg/kg po)+Bupropion (20 mg/kg ip bid) vs RDC5 | -11.2 | 0.048* |
| (Day 36-43) | RDC5 (0.6 mg/kg po)+Naltrexone (1 mg/kg ip) vs RDC5 | -0.2 | 0.977 |
|  | RDC5 (0.6 mg/kg po)+Bupropion (20 mg/kg ip bid) vs Bupropion | -4.0 | 0.481 |
|  | Bupropion (20 mg/kg ip bid)+Naltrexone (1 mg/kg ip) vs Bupropion | -3.1 | 0.575 |
|  | RDC5 (0.6 mg/kg po)+Naltrexone (1 mg/kg ip) vs Naltrexone | 5.0 | 0.371 |
|  | Bupropion (20 mg/kg ip bid)+Naltrexone (1 mg/kg ip) vs Naltrexone | -5.2 | 0.354 |

Multiple comparisons are by the multiple t test. *p<0.05.

| Time | Comparison | Difference | p |
| --- | --- | --- | --- |
| Weeks 1-4 | RDC5 (0.6 mg/kg po)+Bupropion (20 mg/kg ip qd) vs RDC5 | 4.9 | 0.602 |
| (Day 1-26) | RDC5 (0.6 mg/kg po)+Naltrexone (1 mg/kg ip) vs RDC5 | 11.1 | 0.238 |
|  | RDC5 (0.6 mg/kg po)+Bupropion (20 mg/kg ip qd) vs Bupropion | -19.6 | 0.039* |
|  | Bupropion (20 mg/kg ip qd)+Naltrexone (1 mg/kg ip) vs Bupropion | 6.8 | 0.467 |
|  | RDC5 (0.6 mg/kg po)+Naltrexone (1 mg/kg ip) vs Naltrexone | -18.6 | 0.051 |
|  | Bupropion (20 mg/kg ip qd)+Naltrexone (1 mg/kg ip) vs Naltrexone | 1.7 | 0.857 |
| Weeks 5-6 | RDC5 (0.6 mg/kg po)+Bupropion (20 mg/kg ip bid) vs RDC5 | -14.1 | 0.019* |
| (Day 26-43) | RDC5 (0.6 mg/kg po)+Naltrexone (1 mg/kg ip) vs RDC5 | 1.7 | 0.773 |
|  | RDC5 (0.6 mg/kg po)+Bupropion (20 mg/kg ip bid) vs Bupropion | -5.8 | 0.329 |
|  | Bupropion (20 mg/kg ip bid)+Naltrexone (1 mg/kg ip) vs Bupropion | -8.6 | 0.149 |
|  | RDC5 (0.6 mg/kg po)+Naltrexone (1 mg/kg ip) vs Naltrexone | 7.0 | 0.235 |
|  | Bupropion (20 mg/kg ip bid)+Naltrexone (1 mg/kg ip) vs Naltrexone | -11.6 | 0.053 |
| Weeks 1-5 | RDC5 (0.6 mg/kg po)+Bupropion (20 mg/kg ip qd/bid) vs RDC5 | 2.0 | 0.842 |
| (Day 1-36) | RDC5 (0.6 mg/kg po)+Naltrexone (1 mg/kg ip) vs RDC5 | 13.0 | 0.204 |
|  | RDC5 (0.6 mg/kg po)+Bupropion (20 mg/kg ip qd/bid) vs Bupropion | -21.4 | 0.038* |
|  | Bupropion (20 mg/kg ip qd/bid)+Naltrexone (1 mg/kg ip) vs Bupropion | 1.4 | 0.887 |
|  | RDC5 (0.6 mg/kg po)+Naltrexone (1 mg/kg ip) vs Naltrexone | -16.6 | 0.107 |
|  | Bupropion (20 mg/kg ip qd/bid)+Naltrexone (1 mg/kg ip) vs Naltrexone | -4.7 | 0.648 |
| Overall | RDC5 (0.6 mg/kg po)+Bupropion (20 mg/kg ip qd/bid) vs RDC5 | -9.2 | 0.328 |
| (Day 1-43) | RDC5 (0.6 mg/kg po)+Naltrexone (1 mg/kg ip) vs RDC5 | 12.8 | 0.175 |
|  | RDC5 (0.6 mg/kg po)+Bupropion (20 mg/kg ip qd/bid) vs Bupropion | -25.4 | 0.008** |
|  | Bupropion (20 mg/kg ip qd/bid)+Naltrexone (1 mg/kg ip) vs Bupropion | -1.7 | 0.856 |
|  | RDC5 (0.6 mg/kg po)+Naltrexone (1 mg/kg ip) vs Naltrexone | -11.5 | 0.223 |
|  | Bupropion (20 mg/kg ip qd/bid)+Naltrexone (1 mg/kg ip) vs Naltrexone | -9.9 | 0.295 |

Multiple comparisons are by the multiple t test. *p<0.05, **p<0.01.

#### their individual constituents (Baseline, Weeks 1 to 6) Food intakes (kJ) on each day (Baseline)

Treatment n Mean SEM Difference % change p from vehicle

| Day -6 | Vehicle 4 ml/kg po +2 ml/kg ip | 11 | 392.4 | 11.8 |  | | |
| --- | --- | --- | --- | --- | --- | --- | --- |
|  | RDC5 0.6 mg/kg po | 10 | 383.1 | 15.6 | -9.3 | -2.4 | 0.718 |
|  | Bupropion 20 mg/kg ip qd | 10 | 391.8 | 21.0 | -0.6 | -0.1 | 0.982 |
|  | Naltrexone 1 mg/kg ip | 10 | 405.7 | 23.1 | 13.4 | 3.4 | 0.603 |
|  | RDC5 0.6 mg/kg po+Bupropion 20 mg/kg ip qd | 10 | 359.2 | 11.9 | -33.1 | -8.4 | 0.200 |
|  | RDC5 0.6 mg/kg po+Naltrexone 1 mg/kg ip | 10 | 399.8 | 15.4 | 7.5 | 1.9 | 0.771 |
|  | Bupropion 20 mg/kg ip qd+Naltrexone 1 mg/kg ip | 10 | 398.7 | 19.7 | 6.3 | 1.6 | 0.805 |
|  | Sibutramine 5 mg/kg po | 10 | 393.0 | 23.4 | 0.6 | 0.2 | 0.982 |
| Day -5 | Vehicle 4 ml/kg po +2 ml/kg ip | 12 | 424.2 | 12.2 |  |  |  |
|  | RDC5 0.6 mg/kg po | 10 | 413.4 | 16.4 | -10.9 | -2.6 | 0.659 |
|  | Bupropion 20 mg/kg ip qd | 10 | 437.5 | 19.2 | 13.2 | 3.1 | 0.592 |
|  | Naltrexone 1 mg/kg ip | 9 | 428.2 | 16.5 | 3.9 | 0.9 | 0.878 |
|  | RDC5 0.6 mg/kg po+Bupropion 20 mg/kg ip qd | 10 | 419.8 | 24.5 | -4.4 | -1.0 | 0.858 |
|  | RDC5 0.6 mg/kg po+Naltrexone 1 mg/kg ip | 10 | 411.5 | 12.3 | -12.7 | -3.0 | 0.605 |
|  | Bupropion 20 mg/kg ip qd+Naltrexone 1 mg/kg ip | 10 | 421.5 | 21.9 | -2.8 | -0.7 | 0.911 |
|  | Sibutramine 5 mg/kg po | 10 | 416.0 | 16.8 | -8.3 | -1.9 | 0.737 |
| Day -4 | Vehicle 4 ml/kg po +2 ml/kg ip | 12 | 400.6 | 13.1 |  |  |  |
|  | RDC5 0.6 mg/kg po | 10 | 391.2 | 17.8 | -9.4 | -2.3 | 0.731 |
|  | Bupropion 20 mg/kg ip qd | 10 | 440.5 | 12.9 | 39.8 | 9.9 | 0.149 |
|  | Naltrexone 1 mg/kg ip | 10 | 416.7 | 31.3 | 16.1 | 4.0 | 0.556 |
|  | RDC5 0.6 mg/kg po+Bupropion 20 mg/kg ip qd | 10 | 388.5 | 20.7 | -12.2 | -3.0 | 0.656 |
|  | RDC5 0.6 mg/kg po+Naltrexone 1 mg/kg ip | 10 | 382.2 | 14.6 | -18.4 | -4.6 | 0.500 |
|  | Bupropion 20 mg/kg ip qd+Naltrexone 1 mg/kg ip | 10 | 408.2 | 23.8 | 7.5 | 1.9 | 0.782 |
|  | Sibutramine 5 mg/kg po | 10 | 397.4 | 17.3 | -3.2 | -0.8 | 0.907 |
| Day -3 | Vehicle 4 ml/kg po +2 ml/kg ip | 12 | 395.7 | 11.3 |  |  |  |
|  | RDC5 0.6 mg/kg po | 10 | 386.5 | 12.0 | -9.2 | -2.3 | 0.723 |
|  | Bupropion 20 mg/kg ip qd | 10 | 345.6 | 23.2 | -50.1 | -12.7 | 0.057 |
|  | Naltrexone 1 mg/kg ip | 10 | 356.3 | 29.3 | -39.4 | -10.0 | 0.131 |
|  | RDC5 0.6 mg/kg po+Bupropion 20 mg/kg ip qd | 10 | 402.5 | 14.4 | 6.8 | 1.7 | 0.793 |
|  | RDC5 0.6 mg/kg po+Naltrexone 1 mg/kg ip | 10 | 395.2 | 14.3 | -0.5 | -0.1 | 0.985 |
|  | Bupropion 20 mg/kg ip qd+Naltrexone 1 mg/kg ip | 10 | 370.2 | 13.2 | -25.5 | -6.4 | 0.326 |
|  | Sibutramine 5 mg/kg po | 10 | 362.9 | 24.1 | -32.8 | -8.3 | 0.207 |
| Day -2 | Vehicle 4 ml/kg po +2 ml/kg ip | 12 | 392.3 | 17.9 |  |  |  |
|  | RDC5 0.6 mg/kg po | 10 | 400.8 | 10.4 | 8.5 | 2.2 | 0.721 |
|  | Bupropion 20 mg/kg ip qd | 10 | 373.3 | 15.4 | -19.0 | -4.8 | 0.426 |
|  | Naltrexone 1 mg/kg ip | 10 | 381.0 | 15.9 | -11.3 | -2.9 | 0.634 |
|  | RDC5 0.6 mg/kg po+Bupropion 20 mg/kg ip qd | 10 | 393.3 | 16.9 | 1.0 | 0.3 | 0.966 |
|  | RDC5 0.6 mg/kg po+Naltrexone 1 mg/kg ip | 10 | 406.1 | 16.7 | 13.8 | 3.5 | 0.562 |
|  | Bupropion 20 mg/kg ip qd+Naltrexone 1 mg/kg ip | 10 | 405.2 | 16.5 | 12.9 | 3.3 | 0.589 |
|  | Sibutramine 5 mg/kg po | 10 | 392.4 | 23.3 | 0.1 | 0.0 | 0.997 |
| Day -1 | Vehicle 4 ml/kg po +2 ml/kg ip | 12 | 382.7 | 9.8 |  |  |  |
|  | RDC5 0.6 mg/kg po | 10 | 362.4 | 8.7 | -20.4 | -5.3 | 0.425 |
|  | Bupropion 20 mg/kg ip qd | 10 | 380.1 | 21.9 | -2.6 | -0.7 | 0.918 |
|  | Naltrexone 1 mg/kg ip | 10 | 388.1 | 10.1 | 5.3 | 1.4 | 0.834 |
|  | RDC5 0.6 mg/kg po+Bupropion 20 mg/kg ip qd | 10 | 373.7 | 21.0 | -9.1 | -2.4 | 0.722 |
|  | RDC5 0.6 mg/kg po+Naltrexone 1 mg/kg ip | 10 | 368.1 | 16.8 | -14.7 | -3.8 | 0.566 |
|  | Bupropion 20 mg/kg ip qd+Naltrexone 1 mg/kg ip | 10 | 375.1 | 30.6 | -7.7 | -2.0 | 0.764 |
|  | Sibutramine 5 mg/kg po | 10 | 392.1 | 18.8 | 9.3 | 2.4 | 0.715 |
| Day 0 | Vehicle 4 ml/kg po +2 ml/kg ip | 12 | 334.8 | 23.5 |  |  |  |
|  | RDC5 0.6 mg/kg po | 10 | 385.6 | 15.9 | 50.9 | 15.2 | 0.082 |
|  | Bupropion 20 mg/kg ip qd | 10 | 354.5 | 16.7 | 19.7 | 5.9 | 0.497 |
|  | Naltrexone 1 mg/kg ip | 10 | 353.8 | 20.5 | 19.0 | 5.7 | 0.511 |
|  | RDC5 0.6 mg/kg po+Bupropion 20 mg/kg ip qd | 10 | 385.9 | 19.1 | 51.1 | 15.3 | 0.081 |
|  | RDC5 0.6 mg/kg po+Naltrexone 1 mg/kg ip | 10 | 360.1 | 20.7 | 25.3 | 7.6 | 0.383 |
|  | Bupropion 20 mg/kg ip qd+Naltrexone 1 mg/kg ip | 10 | 344.3 | 27.5 | 9.5 | 2.9 | 0.742 |
|  | Sibutramine 5 mg/kg po | 10 | 369.2 | 17.7 | 34.5 | 10.3 | 0.236 |

Means are adjusted for differences between the treatment groups at baseline (average of Days -6 to 0). SEM are calculated from the residuals of the statistical model. Multiple comparisons against vehicle are by the multiple t test.

Treatment n Mean SEM Difference % change p

from vehicle

| Day 1 | Vehicle 4 ml/kg po +2 ml/kg ip | 12 | 345.3 | 25.0 |  |  |  |
| --- | --- | --- | --- | --- | --- | --- | --- |
|  | RDC5 0.6 mg/kg po | 10 | 320.5 | 26.0 | -24.8 | -7.2 | 0.333 |
|  | Bupropion 20 mg/kg ip qd | 10 | 336.5 | 14.7 | -8.9 | -2.6 | 0.730 |
|  | Naltrexone 1 mg/kg ip | 10 | 298.2 | 16.8 | -47.2 | -13.7 | 0.069 |
|  | RDC5 0.6 mg/kg po+Bupropion 20 mg/kg ip qd | 10 | 313.7 | 19.3 | -31.6 | -9.1 | 0.220 |
|  | RDC5 0.6 mg/kg po+Naltrexone 1 mg/kg ip | 10 | 298.6 | 11.7 | -46.7 | -13.5 | 0.071 |
|  | Bupropion 20 mg/kg ip qd+Naltrexone 1 mg/kg ip | 10 | 325.5 | 13.7 | -19.8 | -5.7 | 0.440 |
|  | Sibutramine 5 mg/kg po | 10 | 46.9 | 7.3 | -298.5 | -86.4 | <0.001*** |
| Day 2 | Vehicle 4 ml/kg po +2 ml/kg ip | 12 | 361.4 | 20.8 |  |  |  |
|  | RDC5 0.6 mg/kg po | 10 | 365.2 | 15.7 | 3.7 | 1.0 | 0.882 |
|  | Bupropion 20 mg/kg ip qd | 10 | 384.0 | 15.5 | 22.6 | 6.3 | 0.372 |
|  | Naltrexone 1 mg/kg ip | 10 | 345.2 | 18.5 | -16.3 | -4.5 | 0.519 |
|  | RDC5 0.6 mg/kg po+Bupropion 20 mg/kg ip qd | 10 | 341.8 | 10.9 | -19.7 | -5.4 | 0.436 |
|  | RDC5 0.6 mg/kg po+Naltrexone 1 mg/kg ip | 10 | 352.3 | 16.4 | -9.2 | -2.5 | 0.716 |
|  | Bupropion 20 mg/kg ip qd+Naltrexone 1 mg/kg ip | 10 | 346.7 | 21.0 | -14.7 | -4.1 | 0.560 |
|  | Sibutramine 5 mg/kg po | 10 | 88.3 | 21.5 | -273.1 | -75.6 | <0.001*** |
| Day 3 | Vehicle 4 ml/kg po +2 ml/kg ip | 12 | 351.7 | 15.3 |  |  |  |
|  | RDC5 0.6 mg/kg po | 10 | 304.0 | 21.8 | -47.7 | -13.6 | 0.096 |
|  | Bupropion 20 mg/kg ip qd | 10 | 368.0 | 18.3 | 16.3 | 4.6 | 0.567 |
|  | Naltrexone 1 mg/kg ip | 10 | 357.3 | 19.4 | 5.7 | 1.6 | 0.842 |
|  | RDC5 0.6 mg/kg po+Bupropion 20 mg/kg ip qd | 10 | 314.3 | 15.4 | -37.4 | -10.6 | 0.191 |
|  | RDC5 0.6 mg/kg po+Naltrexone 1 mg/kg ip | 10 | 324.7 | 20.9 | -27.0 | -7.7 | 0.343 |
|  | Bupropion 20 mg/kg ip qd+Naltrexone 1 mg/kg ip | 10 | 341.0 | 22.2 | -10.6 | -3.0 | 0.708 |
|  | Sibutramine 5 mg/kg po | 10 | 179.0 | 28.2 | -172.6 | -49.1 | <0.001*** |
| Day 4 | Vehicle 4 ml/kg po +2 ml/kg ip | 12 | 352.4 | 15.2 |  |  |  |
|  | RDC5 0.6 mg/kg po | 10 | 315.4 | 22.4 | -37.0 | -10.5 | 0.192 |
|  | Bupropion 20 mg/kg ip qd | 10 | 366.4 | 20.4 | 14.0 | 4.0 | 0.621 |
|  | Naltrexone 1 mg/kg ip | 10 | 335.4 | 28.6 | -17.0 | -4.8 | 0.547 |
|  | RDC5 0.6 mg/kg po+Bupropion 20 mg/kg ip qd | 10 | 325.5 | 20.0 | -26.9 | -7.6 | 0.342 |
|  | RDC5 0.6 mg/kg po+Naltrexone 1 mg/kg ip | 10 | 321.9 | 21.7 | -30.5 | -8.7 | 0.281 |
|  | Bupropion 20 mg/kg ip qd+Naltrexone 1 mg/kg ip | 10 | 384.3 | 15.5 | 31.9 | 9.1 | 0.259 |
|  | Sibutramine 5 mg/kg po | 10 | 227.0 | 16.2 | -125.4 | -35.6 | <0.001*** |
| Day 5 | Vehicle 4 ml/kg po +2 ml/kg ip | 12 | 373.3 | 17.0 |  |  |  |
|  | RDC5 0.6 mg/kg po | 10 | 328.7 | 28.8 | -44.6 | -11.9 | 0.181 |
|  | Bupropion 20 mg/kg ip qd | 10 | 353.0 | 10.2 | -20.3 | -5.4 | 0.542 |
|  | Naltrexone 1 mg/kg ip | 10 | 325.8 | 30.4 | -47.5 | -12.7 | 0.155 |
|  | RDC5 0.6 mg/kg po+Bupropion 20 mg/kg ip qd | 10 | 338.8 | 17.9 | -34.5 | -9.2 | 0.301 |
|  | RDC5 0.6 mg/kg po+Naltrexone 1 mg/kg ip | 10 | 330.8 | 19.0 | -42.5 | -11.4 | 0.202 |
|  | Bupropion 20 mg/kg ip qd+Naltrexone 1 mg/kg ip | 10 | 390.0 | 32.2 | 16.7 | 4.5 | 0.615 |
|  | Sibutramine 5 mg/kg po | 10 | 241.1 | 27.8 | -132.2 | -35.4 | <0.001*** |
| Day 6 | Vehicle 4 ml/kg po +2 ml/kg ip | 12 | 354.6 | 22.4 |  |  |  |
|  | RDC5 0.6 mg/kg po | 10 | 283.0 | 18.3 | -71.6 | -20.2 | 0.027* |
|  | Bupropion 20 mg/kg ip qd | 10 | 374.4 | 22.1 | 19.8 | 5.6 | 0.537 |
|  | Naltrexone 1 mg/kg ip | 10 | 355.5 | 26.5 | 0.9 | 0.3 | 0.977 |
|  | RDC5 0.6 mg/kg po+Bupropion 20 mg/kg ip qd | 10 | 333.1 | 18.0 | -21.4 | -6.0 | 0.502 |
|  | RDC5 0.6 mg/kg po+Naltrexone 1 mg/kg ip | 10 | 335.9 | 21.2 | -18.7 | -5.3 | 0.557 |
|  | Bupropion 20 mg/kg ip qd+Naltrexone 1 mg/kg ip | 10 | 413.2 | 25.4 | 58.6 | 16.5 | 0.069 |
|  | Sibutramine 5 mg/kg po | 10 | 234.4 | 26.9 | -120.2 | -33.9 | <0.001*** |
| Day 7 | Vehicle 4 ml/kg po +2 ml/kg ip | 12 | 359.3 | 25.4 |  |  |  |
|  | RDC5 0.6 mg/kg po | 10 | 292.3 | 18.5 | -67.1 | -18.7 | 0.061 |
|  | Bupropion 20 mg/kg ip qd | 10 | 344.9 | 22.9 | -14.4 | -4.0 | 0.684 |
|  | Naltrexone 1 mg/kg ip | 10 | 383.0 | 30.9 | 23.6 | 6.6 | 0.505 |
|  | RDC5 0.6 mg/kg po+Bupropion 20 mg/kg ip qd | 10 | 296.8 | 28.0 | -62.5 | -17.4 | 0.081 |
|  | RDC5 0.6 mg/kg po+Naltrexone 1 mg/kg ip | 10 | 316.4 | 12.5 | -42.9 | -11.9 | 0.228 |
|  | Bupropion 20 mg/kg ip qd+Naltrexone 1 mg/kg ip | 10 | 340.3 | 32.8 | -19.0 | -5.3 | 0.592 |
|  | Sibutramine 5 mg/kg po | 10 | 288.2 | 25.6 | -71.1 | -19.8 | 0.048* |

Means are adjusted for differences between the treatment groups at baseline (average of Days -6 to 0). SEM are calculated from the residuals of the statistical model. Multiple comparisons against vehicle are by the multiple t test. *p<0.05,

***p<0.001.

Treatment n Mean SEM Difference % change p

from vehicle

| Day 8 | Vehicle 4 ml/kg po +2 ml/kg ip | 12 | 334.6 | 22.1 |  |  |  |
| --- | --- | --- | --- | --- | --- | --- | --- |
|  | RDC5 0.6 mg/kg po | 10 | 300.8 | 24.2 | -33.8 | -10.1 | 0.291 |
|  | Bupropion 20 mg/kg ip qd | 10 | 372.5 | 24.9 | 37.9 | 11.3 | 0.239 |
|  | Naltrexone 1 mg/kg ip | 9 | 374.9 | 23.8 | 40.3 | 12.1 | 0.223 |
|  | RDC5 0.6 mg/kg po+Bupropion 20 mg/kg ip qd | 10 | 330.8 | 19.7 | -3.8 | -1.1 | 0.905 |
|  | RDC5 0.6 mg/kg po+Naltrexone 1 mg/kg ip | 10 | 324.0 | 16.5 | -10.6 | -3.2 | 0.739 |
|  | Bupropion 20 mg/kg ip qd+Naltrexone 1 mg/kg ip | 10 | 413.2 | 30.4 | 78.5 | 23.5 | 0.016* |
|  | Sibutramine 5 mg/kg po | 10 | 270.4 | 19.9 | -64.2 | -19.2 | 0.048* |
| Day 9 | Vehicle 4 ml/kg po +2 ml/kg ip | 12 | 381.0 | 17.1 |  |  |  |
|  | RDC5 0.6 mg/kg po | 9 | 300.5 | 26.8 | -80.5 | -21.1 | 0.023* |
|  | Bupropion 20 mg/kg ip qd | 10 | 374.1 | 20.3 | -6.9 | -1.8 | 0.839 |
|  | Naltrexone 1 mg/kg ip | 9 | 366.1 | 25.6 | -14.9 | -3.9 | 0.668 |
|  | RDC5 0.6 mg/kg po+Bupropion 20 mg/kg ip qd | 10 | 331.4 | 23.3 | -49.6 | -13.0 | 0.146 |
|  | RDC5 0.6 mg/kg po+Naltrexone 1 mg/kg ip | 10 | 367.3 | 32.8 | -13.7 | -3.6 | 0.684 |
|  | Bupropion 20 mg/kg ip qd+Naltrexone 1 mg/kg ip | 10 | 425.8 | 24.9 | 44.8 | 11.8 | 0.188 |
|  | Sibutramine 5 mg/kg po | 10 | 281.9 | 25.2 | -99.1 | -26.0 | 0.004** |
| Day 10 | Vehicle 4 ml/kg po +2 ml/kg ip | 12 | 335.0 | 27.5 |  |  |  |
|  | RDC5 0.6 mg/kg po | 10 | 305.6 | 27.5 | -29.4 | -8.8 | 0.380 |
|  | Bupropion 20 mg/kg ip qd | 10 | 362.9 | 15.2 | 27.9 | 8.3 | 0.406 |
|  | Naltrexone 1 mg/kg ip | 10 | 366.6 | 17.6 | 31.6 | 9.4 | 0.346 |
|  | RDC5 0.6 mg/kg po+Bupropion 20 mg/kg ip qd | 10 | 347.0 | 28.1 | 12.0 | 3.6 | 0.720 |
|  | RDC5 0.6 mg/kg po+Naltrexone 1 mg/kg ip | 10 | 366.8 | 27.9 | 31.7 | 9.5 | 0.344 |
|  | Bupropion 20 mg/kg ip qd+Naltrexone 1 mg/kg ip | 10 | 409.2 | 16.6 | 74.2 | 22.1 | 0.029* |
|  | Sibutramine 5 mg/kg po | 10 | 292.8 | 24.2 | -42.3 | -12.6 | 0.209 |
| Day 11 | Vehicle 4 ml/kg po +2 ml/kg ip | 12 | 336.3 | 28.1 |  |  |  |
|  | RDC5 0.6 mg/kg po | 10 | 311.9 | 19.4 | -24.3 | -7.2 | 0.492 |
|  | Bupropion 20 mg/kg ip qd | 10 | 397.1 | 26.2 | 60.9 | 18.1 | 0.090 |
|  | Naltrexone 1 mg/kg ip | 10 | 409.5 | 24.2 | 73.2 | 21.8 | 0.041* |
|  | RDC5 0.6 mg/kg po+Bupropion 20 mg/kg ip qd | 10 | 290.1 | 25.6 | -46.2 | -13.7 | 0.195 |
|  | RDC5 0.6 mg/kg po+Naltrexone 1 mg/kg ip | 10 | 336.1 | 19.1 | -0.2 | -0.1 | 0.996 |
|  | Bupropion 20 mg/kg ip qd+Naltrexone 1 mg/kg ip | 10 | 392.4 | 36.0 | 56.1 | 16.7 | 0.116 |
|  | Sibutramine 5 mg/kg po | 10 | 354.9 | 17.4 | 18.6 | 5.5 | 0.599 |
| Day 12 | Vehicle 4 ml/kg po +2 ml/kg ip | 12 | 335.5 | 25.9 |  |  |  |
|  | RDC5 0.6 mg/kg po | 10 | 346.0 | 16.3 | 10.5 | 3.1 | 0.725 |
|  | Bupropion 20 mg/kg ip qd | 10 | 407.4 | 24.6 | 71.9 | 21.4 | 0.018* |
|  | Naltrexone 1 mg/kg ip | 10 | 393.7 | 25.1 | 58.2 | 17.3 | 0.054 |
|  | RDC5 0.6 mg/kg po+Bupropion 20 mg/kg ip qd | 10 | 323.6 | 22.7 | -11.9 | -3.5 | 0.690 |
|  | RDC5 0.6 mg/kg po+Naltrexone 1 mg/kg ip | 10 | 326.2 | 12.7 | -9.3 | -2.8 | 0.754 |
|  | Bupropion 20 mg/kg ip qd+Naltrexone 1 mg/kg ip | 10 | 419.8 | 19.0 | 84.3 | 25.1 | 0.006** |
|  | Sibutramine 5 mg/kg po | 10 | 340.5 | 17.9 | 5.0 | 1.5 | 0.866 |
| Day 13 | Vehicle 4 ml/kg po +2 ml/kg ip | 12 | 373.0 | 19.4 |  |  |  |
|  | RDC5 0.6 mg/kg po | 10 | 316.4 | 31.5 | -56.7 | -15.2 | 0.074 |
|  | Bupropion 20 mg/kg ip qd | 10 | 400.1 | 18.6 | 27.0 | 7.3 | 0.391 |
|  | Naltrexone 1 mg/kg ip | 10 | 372.7 | 27.8 | -0.3 | -0.1 | 0.993 |
|  | RDC5 0.6 mg/kg po+Bupropion 20 mg/kg ip qd | 10 | 340.7 | 23.3 | -32.3 | -8.7 | 0.306 |
|  | RDC5 0.6 mg/kg po+Naltrexone 1 mg/kg ip | 10 | 361.9 | 20.4 | -11.1 | -3.0 | 0.723 |
|  | Bupropion 20 mg/kg ip qd+Naltrexone 1 mg/kg ip | 10 | 404.1 | 15.2 | 31.1 | 8.3 | 0.323 |
|  | Sibutramine 5 mg/kg po | 10 | 330.0 | 20.3 | -43.0 | -11.5 | 0.173 |
| Day 14 | Vehicle 4 ml/kg po +2 ml/kg ip | 12 | 341.1 | 23.1 |  |  |  |
|  | RDC5 0.6 mg/kg po | 10 | 287.3 | 26.8 | -53.8 | -15.8 | 0.119 |
|  | Bupropion 20 mg/kg ip qd | 10 | 362.4 | 18.3 | 21.3 | 6.2 | 0.537 |
|  | Naltrexone 1 mg/kg ip | 10 | 355.6 | 21.2 | 14.5 | 4.2 | 0.673 |
|  | RDC5 0.6 mg/kg po+Bupropion 20 mg/kg ip qd | 10 | 340.9 | 26.6 | -0.2 | -0.1 | 0.995 |
|  | RDC5 0.6 mg/kg po+Naltrexone 1 mg/kg ip | 10 | 395.5 | 33.0 | 54.3 | 15.9 | 0.116 |
|  | Bupropion 20 mg/kg ip qd+Naltrexone 1 mg/kg ip | 10 | 390.8 | 21.1 | 49.7 | 14.6 | 0.150 |
|  | Sibutramine 5 mg/kg po | 10 | 289.8 | 23.6 | -51.3 | -15.0 | 0.137 |

Means are adjusted for differences between the treatment groups at baseline (average of Days -6 to 0). SEM are calculated from the residuals of the statistical model. Multiple comparisons against vehicle are by the multiple t test. *p<0.05, **p<0.01.

Treatment n Mean SEM Difference % change p

from vehicle

| Day 15 | Vehicle 4 ml/kg po +2 ml/kg ip | 12 | 330.0 | 22.0 |  |  |  |
| --- | --- | --- | --- | --- | --- | --- | --- |
|  | RDC5 0.6 mg/kg po | 10 | 320.8 | 15.3 | -9.2 | -2.8 | 0.757 |
|  | Bupropion 20 mg/kg ip qd | 10 | 384.1 | 26.4 | 54.2 | 16.4 | 0.073 |
|  | Naltrexone 1 mg/kg ip | 10 | 382.1 | 16.6 | 52.1 | 15.8 | 0.084 |
|  | RDC5 0.6 mg/kg po+Bupropion 20 mg/kg ip qd | 10 | 312.2 | 24.5 | -17.8 | -5.4 | 0.551 |
|  | RDC5 0.6 mg/kg po+Naltrexone 1 mg/kg ip | 10 | 359.2 | 19.6 | 29.2 | 8.8 | 0.329 |
|  | Bupropion 20 mg/kg ip qd+Naltrexone 1 mg/kg ip | 10 | 337.0 | 26.7 | 7.1 | 2.1 | 0.813 |
|  | Sibutramine 5 mg/kg po | 10 | 351.9 | 15.2 | 21.9 | 6.6 | 0.464 |
| Day 16 | Vehicle 4 ml/kg po +2 ml/kg ip | 12 | 338.4 | 20.5 |  |  |  |
|  | RDC5 0.6 mg/kg po | 10 | 371.6 | 17.3 | 33.2 | 9.8 | 0.346 |
|  | Bupropion 20 mg/kg ip qd | 10 | 378.5 | 27.0 | 40.1 | 11.9 | 0.257 |
|  | Naltrexone 1 mg/kg ip | 10 | 431.2 | 35.4 | 92.8 | 27.4 | 0.010** |
|  | RDC5 0.6 mg/kg po+Bupropion 20 mg/kg ip qd | 10 | 324.1 | 19.6 | -14.3 | -4.2 | 0.685 |
|  | RDC5 0.6 mg/kg po+Naltrexone 1 mg/kg ip | 10 | 372.6 | 14.9 | 34.2 | 10.1 | 0.332 |
|  | Bupropion 20 mg/kg ip qd+Naltrexone 1 mg/kg ip | 10 | 416.4 | 38.6 | 78.0 | 23.1 | 0.029* |
|  | Sibutramine 5 mg/kg po | 10 | 379.8 | 18.9 | 41.4 | 12.2 | 0.242 |
| Day 17 | Vehicle 4 ml/kg po +2 ml/kg ip | 12 | 345.9 | 17.1 |  |  |  |
|  | RDC5 0.6 mg/kg po | 10 | 301.9 | 42.2 | -44.0 | -12.7 | 0.280 |
|  | Bupropion 20 mg/kg ip qd | 10 | 349.0 | 34.1 | 3.1 | 0.9 | 0.939 |
|  | Naltrexone 1 mg/kg ip | 10 | 327.8 | 35.8 | -18.1 | -5.2 | 0.655 |
|  | RDC5 0.6 mg/kg po+Bupropion 20 mg/kg ip qd | 10 | 333.0 | 26.0 | -12.9 | -3.7 | 0.751 |
|  | RDC5 0.6 mg/kg po+Naltrexone 1 mg/kg ip | 10 | 373.0 | 31.5 | 27.1 | 7.8 | 0.503 |
|  | Bupropion 20 mg/kg ip qd+Naltrexone 1 mg/kg ip | 10 | 440.0 | 24.4 | 94.1 | 27.2 | 0.023* |
|  | Sibutramine 5 mg/kg po | 10 | 328.3 | 13.4 | -17.5 | -5.1 | 0.666 |
| Day 18 | Vehicle 4 ml/kg po +2 ml/kg ip | 12 | 405.4 | 21.6 |  |  |  |
|  | RDC5 0.6 mg/kg po | 10 | 349.8 | 26.7 | -55.5 | -13.7 | 0.093 |
|  | Bupropion 20 mg/kg ip qd | 10 | 443.9 | 23.8 | 38.6 | 9.5 | 0.243 |
|  | Naltrexone 1 mg/kg ip | 10 | 422.2 | 18.8 | 16.8 | 4.1 | 0.608 |
|  | RDC5 0.6 mg/kg po+Bupropion 20 mg/kg ip qd | 10 | 364.7 | 28.1 | -40.7 | -10.0 | 0.218 |
|  | RDC5 0.6 mg/kg po+Naltrexone 1 mg/kg ip | 10 | 409.8 | 25.3 | 4.4 | 1.1 | 0.893 |
|  | Bupropion 20 mg/kg ip qd+Naltrexone 1 mg/kg ip | 10 | 434.8 | 15.3 | 29.4 | 7.3 | 0.371 |
|  | Sibutramine 5 mg/kg po | 10 | 355.4 | 26.0 | -49.9 | -12.3 | 0.131 |
| Day 19 | Vehicle 4 ml/kg po +2 ml/kg ip | 12 | 373.7 | 17.2 |  |  |  |
|  | RDC5 0.6 mg/kg po | 10 | 371.2 | 19.1 | -2.5 | -0.7 | 0.934 |
|  | Bupropion 20 mg/kg ip qd | 10 | 444.2 | 19.4 | 70.5 | 18.9 | 0.022* |
|  | Naltrexone 1 mg/kg ip | 10 | 417.0 | 24.2 | 43.3 | 11.6 | 0.153 |
|  | RDC5 0.6 mg/kg po+Bupropion 20 mg/kg ip qd | 10 | 351.2 | 27.0 | -22.5 | -6.0 | 0.457 |
|  | RDC5 0.6 mg/kg po+Naltrexone 1 mg/kg ip | 10 | 408.0 | 27.9 | 34.4 | 9.2 | 0.255 |
|  | Bupropion 20 mg/kg ip qd+Naltrexone 1 mg/kg ip | 10 | 397.8 | 19.3 | 24.1 | 6.5 | 0.424 |
|  | Sibutramine 5 mg/kg po | 10 | 354.3 | 16.8 | -19.3 | -5.2 | 0.521 |
| Day 20 | Vehicle 4 ml/kg po +2 ml/kg ip | 12 | 344.4 | 19.4 |  |  |  |
|  | RDC5 0.6 mg/kg po | 10 | 364.4 | 14.6 | 20.0 | 5.8 | 0.462 |
|  | Bupropion 20 mg/kg ip qd | 10 | 378.3 | 15.3 | 33.9 | 9.8 | 0.216 |
|  | Naltrexone 1 mg/kg ip | 10 | 375.8 | 27.0 | 31.4 | 9.1 | 0.249 |
|  | RDC5 0.6 mg/kg po+Bupropion 20 mg/kg ip qd | 10 | 345.5 | 23.1 | 1.1 | 0.3 | 0.967 |
|  | RDC5 0.6 mg/kg po+Naltrexone 1 mg/kg ip | 10 | 357.1 | 21.4 | 12.7 | 3.7 | 0.640 |
|  | Bupropion 20 mg/kg ip qd+Naltrexone 1 mg/kg ip | 10 | 367.0 | 16.1 | 22.6 | 6.6 | 0.406 |
|  | Sibutramine 5 mg/kg po | 10 | 347.8 | 14.8 | 3.4 | 1.0 | 0.900 |
| Day 21 | Vehicle 4 ml/kg po +2 ml/kg ip | 12 | 342.6 | 19.3 |  |  |  |
|  | RDC5 0.6 mg/kg po | 10 | 372.0 | 21.8 | 29.4 | 8.6 | 0.408 |
|  | Bupropion 20 mg/kg ip qd | 10 | 351.3 | 29.4 | 8.6 | 2.5 | 0.808 |
|  | Naltrexone 1 mg/kg ip | 10 | 375.1 | 30.1 | 32.5 | 9.5 | 0.359 |
|  | RDC5 0.6 mg/kg po+Bupropion 20 mg/kg ip qd | 10 | 355.7 | 30.5 | 13.0 | 3.8 | 0.713 |
|  | RDC5 0.6 mg/kg po+Naltrexone 1 mg/kg ip | 10 | 375.3 | 26.7 | 32.7 | 9.5 | 0.357 |
|  | Bupropion 20 mg/kg ip qd+Naltrexone 1 mg/kg ip | 10 | 384.2 | 23.5 | 41.6 | 12.1 | 0.242 |
|  | Sibutramine 5 mg/kg po | 10 | 323.1 | 20.8 | -19.5 | -5.7 | 0.582 |

Means are adjusted for differences between the treatment groups at baseline (average of Days -6 to 0). SEM are calculated from the residuals of the statistical model. Multiple comparisons against vehicle are by the multiple t test. *p<0.05, **p<0.01.

Treatment n Mean SEM Difference % change p

from vehicle

| Day 22 | Vehicle 4 ml/kg po +2 ml/kg ip | 12 | 346.9 | 20.8 |  |  |  |
| --- | --- | --- | --- | --- | --- | --- | --- |
|  | RDC5 0.6 mg/kg po | 10 | 371.5 | 31.6 | 24.6 | 7.1 | 0.448 |
|  | Bupropion 20 mg/kg ip qd | 10 | 398.2 | 10.6 | 51.2 | 14.8 | 0.118 |
|  | Naltrexone 1 mg/kg ip | 10 | 402.3 | 30.2 | 55.4 | 16.0 | 0.091 |
|  | RDC5 0.6 mg/kg po+Bupropion 20 mg/kg ip qd | 10 | 399.1 | 24.6 | 52.1 | 15.0 | 0.111 |
|  | RDC5 0.6 mg/kg po+Naltrexone 1 mg/kg ip | 10 | 413.2 | 21.5 | 66.3 | 19.1 | 0.044* |
|  | Bupropion 20 mg/kg ip qd+Naltrexone 1 mg/kg ip | 10 | 379.7 | 15.9 | 32.8 | 9.5 | 0.313 |
|  | Sibutramine 5 mg/kg po | 10 | 319.0 | 23.7 | -27.9 | -8.0 | 0.390 |
| Day 23 | Vehicle 4 ml/kg po +2 ml/kg ip | 12 | 372.7 | 20.9 |  |  |  |
|  | RDC5 0.6 mg/kg po | 10 | 378.8 | 19.5 | 6.1 | 1.6 | 0.845 |
|  | Bupropion 20 mg/kg ip qd | 10 | 417.6 | 19.2 | 44.9 | 12.0 | 0.151 |
|  | Naltrexone 1 mg/kg ip | 10 | 433.5 | 20.8 | 60.8 | 16.3 | 0.053 |
|  | RDC5 0.6 mg/kg po+Bupropion 20 mg/kg ip qd | 10 | 382.3 | 32.9 | 9.6 | 2.6 | 0.757 |
|  | RDC5 0.6 mg/kg po+Naltrexone 1 mg/kg ip | 10 | 391.6 | 24.1 | 18.8 | 5.1 | 0.543 |
|  | Bupropion 20 mg/kg ip qd+Naltrexone 1 mg/kg ip | 10 | 386.7 | 21.2 | 13.9 | 3.7 | 0.653 |
|  | Sibutramine 5 mg/kg po | 10 | 353.0 | 14.7 | -19.8 | -5.3 | 0.524 |
| Day 24 | Vehicle 4 ml/kg po +2 ml/kg ip | 12 | 351.8 | 19.5 |  |  |  |
|  | RDC5 0.6 mg/kg po | 10 | 351.5 | 24.6 | -0.3 | -0.1 | 0.992 |
|  | Bupropion 20 mg/kg ip qd | 10 | 407.6 | 21.3 | 55.9 | 15.9 | 0.083 |
|  | Naltrexone 1 mg/kg ip | 10 | 355.5 | 18.9 | 3.7 | 1.0 | 0.908 |
|  | RDC5 0.6 mg/kg po+Bupropion 20 mg/kg ip qd | 10 | 380.6 | 30.8 | 28.9 | 8.2 | 0.367 |
|  | RDC5 0.6 mg/kg po+Naltrexone 1 mg/kg ip | 10 | 351.1 | 20.3 | -0.7 | -0.2 | 0.983 |
|  | Bupropion 20 mg/kg ip qd+Naltrexone 1 mg/kg ip | 10 | 396.0 | 27.8 | 44.2 | 12.6 | 0.168 |
|  | Sibutramine 5 mg/kg po | 10 | 348.6 | 16.9 | -3.2 | -0.9 | 0.920 |
| Day 25 | Vehicle 4 ml/kg po +2 ml/kg ip | 11 | 338.7 | 21.4 |  |  |  |
|  | RDC5 0.6 mg/kg po | 10 | 360.5 | 25.1 | 21.8 | 6.4 | 0.526 |
|  | Bupropion 20 mg/kg ip qd | 10 | 381.9 | 25.2 | 43.2 | 12.8 | 0.211 |
|  | Naltrexone 1 mg/kg ip | 10 | 374.5 | 29.8 | 35.8 | 10.6 | 0.299 |
|  | RDC5 0.6 mg/kg po+Bupropion 20 mg/kg ip qd | 10 | 375.0 | 30.2 | 36.2 | 10.7 | 0.295 |
|  | RDC5 0.6 mg/kg po+Naltrexone 1 mg/kg ip | 10 | 324.6 | 20.5 | -14.1 | -4.2 | 0.681 |
|  | Bupropion 20 mg/kg ip qd+Naltrexone 1 mg/kg ip | 10 | 372.0 | 23.2 | 33.3 | 9.8 | 0.333 |
|  | Sibutramine 5 mg/kg po | 10 | 342.3 | 15.8 | 3.6 | 1.1 | 0.917 |
| Day 26 | Vehicle 4 ml/kg po +2 ml/kg ip | 11 | 355.4 | 21.5 |  |  |  |
|  | RDC5 0.6 mg/kg po | 10 | 369.9 | 23.8 | 14.5 | 4.1 | 0.682 |
|  | Bupropion 20 mg/kg ip bid | 10 | 388.8 | 25.2 | 33.3 | 9.4 | 0.348 |
|  | Naltrexone 1 mg/kg ip | 10 | 407.5 | 30.2 | 52.1 | 14.7 | 0.144 |
|  | RDC5 0.6 mg/kg po+Bupropion 20 mg/kg ip bid | 10 | 389.1 | 25.9 | 33.7 | 9.5 | 0.343 |
|  | RDC5 0.6 mg/kg po+Naltrexone 1 mg/kg ip | 10 | 378.6 | 17.4 | 23.2 | 6.5 | 0.512 |
|  | Bupropion 20 mg/kg ip bid+Naltrexone 1 mg/kg ip | 10 | 371.6 | 27.6 | 16.2 | 4.6 | 0.647 |
|  | Sibutramine 5 mg/kg po | 10 | 335.6 | 26.8 | -19.8 | -5.6 | 0.577 |
| Day 27 | Vehicle 4 ml/kg po +2 ml/kg ip | 11 | 353.6 | 18.6 |  |  |  |
|  | RDC5 0.6 mg/kg po | 10 | 304.1 | 9.4 | -49.5 | -14.0 | 0.101 |
|  | Bupropion 20 mg/kg ip bid | 10 | 368.9 | 23.5 | 15.3 | 4.3 | 0.609 |
|  | Naltrexone 1 mg/kg ip | 10 | 364.2 | 19.7 | 10.6 | 3.0 | 0.722 |
|  | RDC5 0.6 mg/kg po+Bupropion 20 mg/kg ip bid | 10 | 343.7 | 24.8 | -9.9 | -2.8 | 0.741 |
|  | RDC5 0.6 mg/kg po+Naltrexone 1 mg/kg ip | 10 | 371.2 | 19.8 | 17.6 | 5.0 | 0.555 |
|  | Bupropion 20 mg/kg ip bid+Naltrexone 1 mg/kg ip | 9 | 316.9 | 31.8 | -36.6 | -10.4 | 0.235 |
|  | Sibutramine 5 mg/kg po | 10 | 338.3 | 18.1 | -15.3 | -4.3 | 0.610 |
| Day 28 | Vehicle 4 ml/kg po +2 ml/kg ip | 12 | 318.8 | 14.8 |  |  |  |
|  | RDC5 0.6 mg/kg po | 10 | 348.9 | 20.5 | 30.1 | 9.4 | 0.278 |
|  | Bupropion 20 mg/kg ip bid | 10 | 348.4 | 29.1 | 29.6 | 9.3 | 0.287 |
|  | Naltrexone 1 mg/kg ip | 10 | 372.1 | 13.5 | 53.3 | 16.7 | 0.057 |
|  | RDC5 0.6 mg/kg po+Bupropion 20 mg/kg ip bid | 10 | 348.1 | 20.8 | 29.3 | 9.2 | 0.291 |
|  | RDC5 0.6 mg/kg po+Naltrexone 1 mg/kg ip | 10 | 365.7 | 23.3 | 46.9 | 14.7 | 0.093 |
|  | Bupropion 20 mg/kg ip bid+Naltrexone 1 mg/kg ip | 9 | 350.5 | 18.2 | 31.7 | 9.9 | 0.267 |
|  | Sibutramine 5 mg/kg po | 10 | 320.3 | 15.9 | 1.5 | 0.5 | 0.956 |

Means are adjusted for differences between the treatment groups at baseline (average of Days -6 to 0). SEM are calculated from the residuals of the statistical model. Multiple comparisons against vehicle are by the multiple t test. *p<0.05.

Treatment n Mean SEM Difference % change p

from vehicle

| Day 29 | Vehicle 4 ml/kg po +2 ml/kg ip | 12 | 343.2 | 29.4 |  |  |  |
| --- | --- | --- | --- | --- | --- | --- | --- |
|  | RDC5 0.6 mg/kg po | 10 | 354.7 | 26.5 | 11.5 | 3.4 | 0.732 |
|  | Bupropion 20 mg/kg ip bid | 10 | 358.8 | 23.0 | 15.6 | 4.6 | 0.643 |
|  | Naltrexone 1 mg/kg ip | 10 | 320.0 | 26.2 | -23.3 | -6.8 | 0.489 |
|  | RDC5 0.6 mg/kg po+Bupropion 20 mg/kg ip bid | 10 | 379.1 | 22.0 | 35.9 | 10.5 | 0.287 |
|  | RDC5 0.6 mg/kg po+Naltrexone 1 mg/kg ip | 10 | 309.3 | 23.1 | -33.9 | -9.9 | 0.314 |
|  | Bupropion 20 mg/kg ip bid+Naltrexone 1 mg/kg ip | 10 | 371.4 | 16.9 | 28.2 | 8.2 | 0.403 |
|  | Sibutramine 5 mg/kg po | 10 | 288.3 | 19.8 | -54.9 | -16.0 | 0.105 |
| Day 30 | Vehicle 4 ml/kg po +2 ml/kg ip | 12 | 329.8 | 26.2 |  |  |  |
|  | RDC5 0.6 mg/kg po | 10 | 375.5 | 44.4 | 45.7 | 13.8 | 0.284 |
|  | Bupropion 20 mg/kg ip bid | 10 | 369.6 | 24.7 | 39.8 | 12.1 | 0.352 |
|  | Naltrexone 1 mg/kg ip | 10 | 367.0 | 19.2 | 37.2 | 11.3 | 0.383 |
|  | RDC5 0.6 mg/kg po+Bupropion 20 mg/kg ip bid | 10 | 384.0 | 15.7 | 54.1 | 16.4 | 0.206 |
|  | RDC5 0.6 mg/kg po+Naltrexone 1 mg/kg ip | 10 | 408.2 | 24.5 | 78.4 | 23.8 | 0.068 |
|  | Bupropion 20 mg/kg ip bid+Naltrexone 1 mg/kg ip | 10 | 374.5 | 52.9 | 44.7 | 13.5 | 0.295 |
|  | Sibutramine 5 mg/kg po | 10 | 337.2 | 15.0 | 7.4 | 2.2 | 0.862 |
| Day 31 | Vehicle 4 ml/kg po +2 ml/kg ip | 12 | 308.2 | 36.1 |  |  |  |
|  | RDC5 0.6 mg/kg po | 10 | 307.4 | 30.5 | -0.8 | -0.3 | 0.984 |
|  | Bupropion 20 mg/kg ip bid | 10 | 360.2 | 29.5 | 52.0 | 16.9 | 0.209 |
|  | Naltrexone 1 mg/kg ip | 10 | 360.7 | 25.7 | 52.5 | 17.0 | 0.203 |
|  | RDC5 0.6 mg/kg po+Bupropion 20 mg/kg ip bid | 10 | 361.1 | 25.8 | 52.9 | 17.1 | 0.201 |
|  | RDC5 0.6 mg/kg po+Naltrexone 1 mg/kg ip | 10 | 392.7 | 25.3 | 84.4 | 27.4 | 0.042* |
|  | Bupropion 20 mg/kg ip bid+Naltrexone 1 mg/kg ip | 10 | 374.3 | 38.2 | 66.1 | 21.4 | 0.110 |
|  | Sibutramine 5 mg/kg po | 10 | 339.0 | 12.0 | 30.7 | 10.0 | 0.455 |
| Day 32 | Vehicle 4 ml/kg po +2 ml/kg ip | 12 | 341.9 | 26.0 |  |  |  |
|  | RDC5 0.6 mg/kg po | 10 | 269.5 | 39.2 | -72.4 | -21.2 | 0.056 |
|  | Bupropion 20 mg/kg ip bid | 10 | 330.3 | 30.8 | -11.6 | -3.4 | 0.757 |
|  | Naltrexone 1 mg/kg ip | 10 | 356.9 | 21.4 | 15.0 | 4.4 | 0.688 |
|  | RDC5 0.6 mg/kg po+Bupropion 20 mg/kg ip bid | 10 | 316.8 | 27.8 | -25.1 | -7.3 | 0.504 |
|  | RDC5 0.6 mg/kg po+Naltrexone 1 mg/kg ip | 10 | 350.9 | 19.9 | 9.0 | 2.6 | 0.811 |
|  | Bupropion 20 mg/kg ip bid+Naltrexone 1 mg/kg ip | 10 | 307.9 | 26.8 | -34.1 | -10.0 | 0.364 |
|  | Sibutramine 5 mg/kg po | 10 | 332.5 | 15.5 | -9.4 | -2.8 | 0.801 |
| Day 33 | Vehicle 4 ml/kg po +2 ml/kg ip | 12 | 325.0 | 26.1 |  |  |  |
|  | RDC5 0.6 mg/kg po | 10 | 332.8 | 33.3 | 7.8 | 2.4 | 0.836 |
|  | Bupropion 20 mg/kg ip bid | 10 | 346.6 | 25.8 | 21.7 | 6.7 | 0.567 |
|  | Naltrexone 1 mg/kg ip | 9 | 337.2 | 27.3 | 12.2 | 3.8 | 0.754 |
|  | RDC5 0.6 mg/kg po+Bupropion 20 mg/kg ip bid | 10 | 333.2 | 26.4 | 8.3 | 2.5 | 0.827 |
|  | RDC5 0.6 mg/kg po+Naltrexone 1 mg/kg ip | 9 | 361.6 | 35.1 | 36.7 | 11.3 | 0.346 |
|  | Bupropion 20 mg/kg ip bid+Naltrexone 1 mg/kg ip | 10 | 324.8 | 26.7 | -0.2 | -0.1 | 0.996 |
|  | Sibutramine 5 mg/kg po | 10 | 343.2 | 14.9 | 18.2 | 5.6 | 0.629 |
| Day 34 | Vehicle 4 ml/kg po +2 ml/kg ip | 12 | 316.4 | 28.1 |  |  |  |
|  | RDC5 0.6 mg/kg po | 10 | 358.0 | 9.4 | 41.6 | 13.2 | 0.210 |
|  | Bupropion 20 mg/kg ip bid | 10 | 352.6 | 12.6 | 36.2 | 11.4 | 0.276 |
|  | Naltrexone 1 mg/kg ip | 10 | 286.6 | 17.1 | -29.7 | -9.4 | 0.369 |
|  | RDC5 0.6 mg/kg po+Bupropion 20 mg/kg ip bid | 10 | 384.8 | 20.3 | 68.4 | 21.6 | 0.042* |
|  | RDC5 0.6 mg/kg po+Naltrexone 1 mg/kg ip | 10 | 353.4 | 28.5 | 37.0 | 11.7 | 0.265 |
|  | Bupropion 20 mg/kg ip bid+Naltrexone 1 mg/kg ip | 10 | 346.8 | 33.9 | 30.4 | 9.6 | 0.359 |
|  | Sibutramine 5 mg/kg po | 9 | 328.1 | 27.0 | 11.7 | 3.7 | 0.731 |
| Day 35 | Vehicle 4 ml/kg po +2 ml/kg ip | 12 | 331.7 | 23.1 |  |  |  |
|  | RDC5 0.6 mg/kg po | 10 | 388.3 | 12.7 | 56.6 | 17.1 | 0.102 |
|  | Bupropion 20 mg/kg ip bid | 10 | 343.5 | 24.4 | 11.8 | 3.6 | 0.731 |
|  | Naltrexone 1 mg/kg ip | 10 | 347.7 | 24.6 | 16.0 | 4.8 | 0.641 |
|  | RDC5 0.6 mg/kg po+Bupropion 20 mg/kg ip bid | 10 | 363.6 | 25.6 | 32.0 | 9.6 | 0.353 |
|  | RDC5 0.6 mg/kg po+Naltrexone 1 mg/kg ip | 10 | 386.5 | 17.7 | 54.8 | 16.5 | 0.113 |
|  | Bupropion 20 mg/kg ip bid+Naltrexone 1 mg/kg ip | 10 | 303.0 | 39.1 | -28.7 | -8.7 | 0.404 |
|  | Sibutramine 5 mg/kg po | 9 | 339.5 | 21.7 | 7.8 | 2.4 | 0.825 |

Means are adjusted for differences between the treatment groups at baseline (average of Days -6 to 0). SEM are calculated from the residuals of the statistical model. Multiple comparisons against vehicle are by the multiple t test. *p<0.05.

Treatment n Mean SEM Difference % change p

from vehicle

| Day 36 | Vehicle 4 ml/kg po +2 ml/kg ip | 12 | 171.3 | 15.4 |  |  |  |
| --- | --- | --- | --- | --- | --- | --- | --- |
|  | RDC5 0.6 mg/kg po | 10 | 172.4 | 19.4 | 1.1 | 0.6 | 0.970 |
|  | Bupropion 20 mg/kg ip bid | 10 | 171.7 | 12.3 | 0.4 | 0.2 | 0.990 |
|  | Naltrexone 1 mg/kg ip | 10 | 152.2 | 12.9 | -19.1 | -11.1 | 0.518 |
|  | RDC5 0.6 mg/kg po+Bupropion 20 mg/kg ip bid | 10 | 168.3 | 13.1 | -3.0 | -1.8 | 0.918 |
|  | RDC5 0.6 mg/kg po+Naltrexone 1 mg/kg ip | 10 | 193.0 | 41.9 | 21.7 | 12.6 | 0.464 |
|  | Bupropion 20 mg/kg ip bid+Naltrexone 1 mg/kg ip | 10 | 144.6 | 18.8 | -26.7 | -15.6 | 0.368 |
|  | Sibutramine 5 mg/kg po | 10 | 149.4 | 21.2 | -21.9 | -12.8 | 0.460 |
| Day 37 | Vehicle 4 ml/kg po +2 ml/kg ip | 12 | 291.8 | 21.0 |  |  |  |
|  | RDC5 0.6 mg/kg po | 10 | 339.7 | 23.5 | 47.9 | 16.4 | 0.096 |
|  | Bupropion 20 mg/kg ip bid | 10 | 341.4 | 20.5 | 49.6 | 17.0 | 0.087 |
|  | Naltrexone 1 mg/kg ip | 10 | 322.0 | 19.3 | 30.2 | 10.3 | 0.292 |
|  | RDC5 0.6 mg/kg po+Bupropion 20 mg/kg ip bid | 10 | 315.1 | 15.1 | 23.3 | 8.0 | 0.416 |
|  | RDC5 0.6 mg/kg po+Naltrexone 1 mg/kg ip | 10 | 362.8 | 24.4 | 71.0 | 24.3 | 0.015* |
|  | Bupropion 20 mg/kg ip bid+Naltrexone 1 mg/kg ip | 10 | 338.5 | 23.5 | 46.7 | 16.0 | 0.105 |
|  | Sibutramine 5 mg/kg po | 10 | 297.7 | 12.8 | 5.9 | 2.0 | 0.837 |
| Day 38 | Vehicle 4 ml/kg po +2 ml/kg ip | 12 | 320.1 | 23.7 |  |  |  |
|  | RDC5 0.6 mg/kg po | 10 | 383.7 | 22.8 | 63.6 | 19.9 | 0.054 |
|  | Bupropion 20 mg/kg ip bid | 10 | 392.7 | 23.0 | 72.6 | 22.7 | 0.029* |
|  | Naltrexone 1 mg/kg ip | 10 | 355.9 | 26.4 | 35.9 | 11.2 | 0.273 |
|  | RDC5 0.6 mg/kg po+Bupropion 20 mg/kg ip bid | 10 | 367.4 | 27.8 | 47.3 | 14.8 | 0.149 |
|  | RDC5 0.6 mg/kg po+Naltrexone 1 mg/kg ip | 10 | 389.6 | 21.7 | 69.5 | 21.7 | 0.035* |
|  | Bupropion 20 mg/kg ip bid+Naltrexone 1 mg/kg ip | 10 | 378.1 | 17.5 | 58.0 | 18.1 | 0.078 |
|  | Sibutramine 5 mg/kg po | 10 | 328.4 | 21.6 | 8.3 | 2.6 | 0.798 |
| Day 39 | Vehicle 4 ml/kg po +2 ml/kg ip | 12 | 355.7 | 26.3 |  |  |  |
|  | RDC5 0.6 mg/kg po | 10 | 374.9 | 14.8 | 19.2 | 5.4 | 0.580 |
|  | Bupropion 20 mg/kg ip bid | 10 | 368.0 | 19.0 | 12.3 | 3.5 | 0.724 |
|  | Naltrexone 1 mg/kg ip | 10 | 340.5 | 24.7 | -15.2 | -4.3 | 0.662 |
|  | RDC5 0.6 mg/kg po+Bupropion 20 mg/kg ip bid | 9 | 335.2 | 38.7 | -20.5 | -5.8 | 0.569 |
|  | RDC5 0.6 mg/kg po+Naltrexone 1 mg/kg ip | 10 | 415.0 | 20.4 | 59.3 | 16.7 | 0.091 |
|  | Bupropion 20 mg/kg ip bid+Naltrexone 1 mg/kg ip | 10 | 334.3 | 22.8 | -21.4 | -6.0 | 0.539 |
|  | Sibutramine 5 mg/kg po | 10 | 322.4 | 28.5 | -33.3 | -9.4 | 0.340 |
| Day 40 | Vehicle 4 ml/kg po +2 ml/kg ip | 12 | 358.3 | 21.3 |  |  |  |
|  | RDC5 0.6 mg/kg po | 10 | 416.6 | 22.8 | 58.3 | 16.3 | 0.100 |
|  | Bupropion 20 mg/kg ip bid | 10 | 384.6 | 25.4 | 26.3 | 7.4 | 0.456 |
|  | Naltrexone 1 mg/kg ip | 10 | 397.9 | 25.0 | 39.6 | 11.0 | 0.262 |
|  | RDC5 0.6 mg/kg po+Bupropion 20 mg/kg ip bid | 10 | 320.1 | 37.8 | -38.2 | -10.7 | 0.280 |
|  | RDC5 0.6 mg/kg po+Naltrexone 1 mg/kg ip | 10 | 411.7 | 20.3 | 53.4 | 14.9 | 0.132 |
|  | Bupropion 20 mg/kg ip bid+Naltrexone 1 mg/kg ip | 10 | 346.4 | 28.8 | -11.9 | -3.3 | 0.734 |
|  | Sibutramine 5 mg/kg po | 10 | 373.5 | 15.0 | 15.2 | 4.2 | 0.665 |
| Day 41 | Vehicle 4 ml/kg po +2 ml/kg ip | 12 | 359.9 | 16.5 |  |  |  |
|  | RDC5 0.6 mg/kg po | 10 | 404.4 | 21.3 | 44.5 | 12.4 | 0.123 |
|  | Bupropion 20 mg/kg ip bid | 10 | 365.3 | 20.7 | 5.3 | 1.5 | 0.852 |
|  | Naltrexone 1 mg/kg ip | 10 | 374.3 | 29.4 | 14.3 | 4.0 | 0.617 |
|  | RDC5 0.6 mg/kg po+Bupropion 20 mg/kg ip bid | 10 | 301.3 | 22.4 | -58.6 | -16.3 | 0.044* |
|  | RDC5 0.6 mg/kg po+Naltrexone 1 mg/kg ip | 10 | 380.4 | 21.5 | 20.5 | 5.7 | 0.475 |
|  | Bupropion 20 mg/kg ip bid+Naltrexone 1 mg/kg ip | 10 | 348.3 | 12.2 | -11.6 | -3.2 | 0.685 |
|  | Sibutramine 5 mg/kg po | 10 | 321.4 | 17.1 | -38.5 | -10.7 | 0.182 |
| Day 42 | Vehicle 4 ml/kg po +2 ml/kg ip | 12 | 361.5 | 19.0 |  |  |  |
|  | RDC5 0.6 mg/kg po | 10 | 387.2 | 25.9 | 25.6 | 7.1 | 0.375 |
|  | Bupropion 20 mg/kg ip bid | 10 | 371.0 | 24.7 | 9.5 | 2.6 | 0.743 |
|  | Naltrexone 1 mg/kg ip | 10 | 379.8 | 24.1 | 18.2 | 5.0 | 0.528 |
|  | RDC5 0.6 mg/kg po+Bupropion 20 mg/kg ip bid | 10 | 359.5 | 14.2 | -2.0 | -0.6 | 0.944 |
|  | RDC5 0.6 mg/kg po+Naltrexone 1 mg/kg ip | 10 | 406.5 | 18.8 | 45.0 | 12.4 | 0.122 |
|  | Bupropion 20 mg/kg ip bid+Naltrexone 1 mg/kg ip | 10 | 381.3 | 19.0 | 19.7 | 5.5 | 0.495 |
|  | Sibutramine 5 mg/kg po | 10 | 318.1 | 17.3 | -43.5 | -12.0 | 0.135 |

Means are adjusted for differences between the treatment groups at baseline (average of Days -6 to 0). SEM are calculated from the residuals of the statistical model. Multiple comparisons against vehicle are by the multiple t test. *p<0.05.

| Time | Comparison | Difference | p |
| --- | --- | --- | --- |
| Day -6 | RDC5 (0.6 mg/kg po)+Bupropion (20 mg/kg ip qd) vs RDC5 | -23.9 | 0.366 |
|  | RDC5 (0.6 mg/kg po)+Naltrexone (1 mg/kg ip) vs RDC5 | 16.7 | 0.524 |
|  | RDC5 (0.6 mg/kg po)+Bupropion (20 mg/kg ip qd) vs Bupropion | -32.6 | 0.222 |
|  | Bupropion (20 mg/kg ip qd)+Naltrexone (1 mg/kg ip) vs Bupropion | 6.9 | 0.793 |
|  | RDC5 (0.6 mg/kg po)+Naltrexone (1 mg/kg ip) vs Naltrexone | -5.9 | 0.823 |
|  | Bupropion (20 mg/kg ip qd)+Naltrexone (1 mg/kg ip) vs Naltrexone | -7.0 | 0.789 |
| Day -5 | RDC5 (0.6 mg/kg po)+Bupropion (20 mg/kg ip qd) vs RDC5 | 6.5 | 0.802 |
|  | RDC5 (0.6 mg/kg po)+Naltrexone (1 mg/kg ip) vs RDC5 | -1.9 | 0.942 |
|  | RDC5 (0.6 mg/kg po)+Bupropion (20 mg/kg ip qd) vs Bupropion | -17.7 | 0.497 |
|  | Bupropion (20 mg/kg ip qd)+Naltrexone (1 mg/kg ip) vs Bupropion | -16.0 | 0.535 |
|  | RDC5 (0.6 mg/kg po)+Naltrexone (1 mg/kg ip) vs Naltrexone | -16.6 | 0.530 |
|  | Bupropion (20 mg/kg ip qd)+Naltrexone (1 mg/kg ip) vs Naltrexone | -6.7 | 0.801 |
| Day -4 | RDC5 (0.6 mg/kg po)+Bupropion (20 mg/kg ip qd) vs RDC5 | -2.8 | 0.922 |
|  | RDC5 (0.6 mg/kg po)+Naltrexone (1 mg/kg ip) vs RDC5 | -9.0 | 0.751 |
|  | RDC5 (0.6 mg/kg po)+Bupropion (20 mg/kg ip qd) vs Bupropion | -52.0 | 0.074 |
|  | Bupropion (20 mg/kg ip qd)+Naltrexone (1 mg/kg ip) vs Bupropion | -32.3 | 0.260 |
|  | RDC5 (0.6 mg/kg po)+Naltrexone (1 mg/kg ip) vs Naltrexone | -34.5 | 0.229 |
|  | Bupropion (20 mg/kg ip qd)+Naltrexone (1 mg/kg ip) vs Naltrexone | -8.5 | 0.765 |
| Day -3 | RDC5 (0.6 mg/kg po)+Bupropion (20 mg/kg ip qd) vs RDC5 | 16.0 | 0.555 |
|  | RDC5 (0.6 mg/kg po)+Naltrexone (1 mg/kg ip) vs RDC5 | 8.7 | 0.748 |
|  | RDC5 (0.6 mg/kg po)+Bupropion (20 mg/kg ip qd) vs Bupropion | 56.9 | 0.040* |
|  | Bupropion (20 mg/kg ip qd)+Naltrexone (1 mg/kg ip) vs Bupropion | 24.6 | 0.365 |
|  | RDC5 (0.6 mg/kg po)+Naltrexone (1 mg/kg ip) vs Naltrexone | 38.9 | 0.153 |
|  | Bupropion (20 mg/kg ip qd)+Naltrexone (1 mg/kg ip) vs Naltrexone | 13.9 | 0.606 |
| Day -2 | RDC5 (0.6 mg/kg po)+Bupropion (20 mg/kg ip qd) vs RDC5 | -7.5 | 0.763 |
|  | RDC5 (0.6 mg/kg po)+Naltrexone (1 mg/kg ip) vs RDC5 | 5.3 | 0.832 |
|  | RDC5 (0.6 mg/kg po)+Bupropion (20 mg/kg ip qd) vs Bupropion | 20.0 | 0.424 |
|  | Bupropion (20 mg/kg ip qd)+Naltrexone (1 mg/kg ip) vs Bupropion | 31.9 | 0.202 |
|  | RDC5 (0.6 mg/kg po)+Naltrexone (1 mg/kg ip) vs Naltrexone | 25.1 | 0.313 |
|  | Bupropion (20 mg/kg ip qd)+Naltrexone (1 mg/kg ip) vs Naltrexone | 24.2 | 0.331 |
| Day -1 | RDC5 (0.6 mg/kg po)+Bupropion (20 mg/kg ip qd) vs RDC5 | 11.3 | 0.672 |
|  | RDC5 (0.6 mg/kg po)+Naltrexone (1 mg/kg ip) vs RDC5 | 5.7 | 0.830 |
|  | RDC5 (0.6 mg/kg po)+Bupropion (20 mg/kg ip qd) vs Bupropion | -6.5 | 0.810 |
|  | Bupropion (20 mg/kg ip qd)+Naltrexone (1 mg/kg ip) vs Bupropion | -5.0 | 0.850 |
|  | RDC5 (0.6 mg/kg po)+Naltrexone (1 mg/kg ip) vs Naltrexone | -20.0 | 0.454 |
|  | Bupropion (20 mg/kg ip qd)+Naltrexone (1 mg/kg ip) vs Naltrexone | -13.0 | 0.626 |
| Day 0 | RDC5 (0.6 mg/kg po)+Bupropion (20 mg/kg ip qd) vs RDC5 | 0.2 | 0.994 |
|  | RDC5 (0.6 mg/kg po)+Naltrexone (1 mg/kg ip) vs RDC5 | -25.5 | 0.399 |
|  | RDC5 (0.6 mg/kg po)+Bupropion (20 mg/kg ip qd) vs Bupropion | 31.3 | 0.305 |
|  | Bupropion (20 mg/kg ip qd)+Naltrexone (1 mg/kg ip) vs Bupropion | -10.2 | 0.736 |
|  | RDC5 (0.6 mg/kg po)+Naltrexone (1 mg/kg ip) vs Naltrexone | 6.3 | 0.835 |
|  | Bupropion (20 mg/kg ip qd)+Naltrexone (1 mg/kg ip) vs Naltrexone | -9.5 | 0.754 |

Multiple comparisons are by the multiple t test. *p<0.05.

| Time | Comparison | Difference | p |
| --- | --- | --- | --- |
| Day 1 | RDC5 (0.6 mg/kg po)+Bupropion (20 mg/kg ip qd) vs RDC5 | -6.8 | 0.800 |
|  | RDC5 (0.6 mg/kg po)+Naltrexone (1 mg/kg ip) vs RDC5 | -21.9 | 0.414 |
|  | RDC5 (0.6 mg/kg po)+Bupropion (20 mg/kg ip qd) vs Bupropion | -22.7 | 0.400 |
|  | Bupropion (20 mg/kg ip qd)+Naltrexone (1 mg/kg ip) vs Bupropion | -10.9 | 0.683 |
|  | RDC5 (0.6 mg/kg po)+Naltrexone (1 mg/kg ip) vs Naltrexone | 0.5 | 0.986 |
|  | Bupropion (20 mg/kg ip qd)+Naltrexone (1 mg/kg ip) vs Naltrexone | 27.4 | 0.307 |
| Day 2 | RDC5 (0.6 mg/kg po)+Bupropion (20 mg/kg ip qd) vs RDC5 | -23.4 | 0.375 |
|  | RDC5 (0.6 mg/kg po)+Naltrexone (1 mg/kg ip) vs RDC5 | -12.9 | 0.624 |
|  | RDC5 (0.6 mg/kg po)+Bupropion (20 mg/kg ip qd) vs Bupropion | -42.3 | 0.114 |
|  | Bupropion (20 mg/kg ip qd)+Naltrexone (1 mg/kg ip) vs Bupropion | -37.3 | 0.160 |
|  | RDC5 (0.6 mg/kg po)+Naltrexone (1 mg/kg ip) vs Naltrexone | 7.1 | 0.787 |
|  | Bupropion (20 mg/kg ip qd)+Naltrexone (1 mg/kg ip) vs Naltrexone | 1.6 | 0.953 |
| Day 3 | RDC5 (0.6 mg/kg po)+Bupropion (20 mg/kg ip qd) vs RDC5 | 10.3 | 0.728 |
|  | RDC5 (0.6 mg/kg po)+Naltrexone (1 mg/kg ip) vs RDC5 | 20.7 | 0.486 |
|  | RDC5 (0.6 mg/kg po)+Bupropion (20 mg/kg ip qd) vs Bupropion | -53.7 | 0.076 |
|  | Bupropion (20 mg/kg ip qd)+Naltrexone (1 mg/kg ip) vs Bupropion | -26.9 | 0.365 |
|  | RDC5 (0.6 mg/kg po)+Naltrexone (1 mg/kg ip) vs Naltrexone | -32.7 | 0.273 |
|  | Bupropion (20 mg/kg ip qd)+Naltrexone (1 mg/kg ip) vs Naltrexone | -16.3 | 0.583 |
| Day 4 | RDC5 (0.6 mg/kg po)+Bupropion (20 mg/kg ip qd) vs RDC5 | 10.0 | 0.733 |
|  | RDC5 (0.6 mg/kg po)+Naltrexone (1 mg/kg ip) vs RDC5 | 6.4 | 0.827 |
|  | RDC5 (0.6 mg/kg po)+Bupropion (20 mg/kg ip qd) vs Bupropion | -40.9 | 0.171 |
|  | Bupropion (20 mg/kg ip qd)+Naltrexone (1 mg/kg ip) vs Bupropion | 17.9 | 0.543 |
|  | RDC5 (0.6 mg/kg po)+Naltrexone (1 mg/kg ip) vs Naltrexone | -13.5 | 0.647 |
|  | Bupropion (20 mg/kg ip qd)+Naltrexone (1 mg/kg ip) vs Naltrexone | 48.9 | 0.100 |
| Day 5 | RDC5 (0.6 mg/kg po)+Bupropion (20 mg/kg ip qd) vs RDC5 | 10.1 | 0.771 |
|  | RDC5 (0.6 mg/kg po)+Naltrexone (1 mg/kg ip) vs RDC5 | 2.1 | 0.952 |
|  | RDC5 (0.6 mg/kg po)+Bupropion (20 mg/kg ip qd) vs Bupropion | -14.2 | 0.685 |
|  | Bupropion (20 mg/kg ip qd)+Naltrexone (1 mg/kg ip) vs Bupropion | 37.0 | 0.288 |
|  | RDC5 (0.6 mg/kg po)+Naltrexone (1 mg/kg ip) vs Naltrexone | 5.0 | 0.886 |
|  | Bupropion (20 mg/kg ip qd)+Naltrexone (1 mg/kg ip) vs Naltrexone | 64.2 | 0.067 |
| Day 6 | RDC5 (0.6 mg/kg po)+Bupropion (20 mg/kg ip qd) vs RDC5 | 50.1 | 0.135 |
|  | RDC5 (0.6 mg/kg po)+Naltrexone (1 mg/kg ip) vs RDC5 | 52.9 | 0.115 |
|  | RDC5 (0.6 mg/kg po)+Bupropion (20 mg/kg ip qd) vs Bupropion | -41.2 | 0.222 |
|  | Bupropion (20 mg/kg ip qd)+Naltrexone (1 mg/kg ip) vs Bupropion | 38.9 | 0.246 |
|  | RDC5 (0.6 mg/kg po)+Naltrexone (1 mg/kg ip) vs Naltrexone | -19.6 | 0.556 |
|  | Bupropion (20 mg/kg ip qd)+Naltrexone (1 mg/kg ip) vs Naltrexone | 57.7 | 0.086 |
| Day 7 | RDC5 (0.6 mg/kg po)+Bupropion (20 mg/kg ip qd) vs RDC5 | 4.5 | 0.903 |
|  | RDC5 (0.6 mg/kg po)+Naltrexone (1 mg/kg ip) vs RDC5 | 24.1 | 0.515 |
|  | RDC5 (0.6 mg/kg po)+Bupropion (20 mg/kg ip qd) vs Bupropion | -48.1 | 0.200 |
|  | Bupropion (20 mg/kg ip qd)+Naltrexone (1 mg/kg ip) vs Bupropion | -4.5 | 0.902 |
|  | RDC5 (0.6 mg/kg po)+Naltrexone (1 mg/kg ip) vs Naltrexone | -66.6 | 0.075 |
|  | Bupropion (20 mg/kg ip qd)+Naltrexone (1 mg/kg ip) vs Naltrexone | -42.6 | 0.251 |

Multiple comparisons are by the multiple t test.

| Time | Comparison | Difference | p |
| --- | --- | --- | --- |
| Day 8 | RDC5 (0.6 mg/kg po)+Bupropion (20 mg/kg ip qd) vs RDC5 | 30.0 | 0.370 |
|  | RDC5 (0.6 mg/kg po)+Naltrexone (1 mg/kg ip) vs RDC5 | 23.2 | 0.488 |
|  | RDC5 (0.6 mg/kg po)+Bupropion (20 mg/kg ip qd) vs Bupropion | -41.7 | 0.218 |
|  | Bupropion (20 mg/kg ip qd)+Naltrexone (1 mg/kg ip) vs Bupropion | 40.6 | 0.226 |
|  | RDC5 (0.6 mg/kg po)+Naltrexone (1 mg/kg ip) vs Naltrexone | -51.0 | 0.140 |
|  | Bupropion (20 mg/kg ip qd)+Naltrexone (1 mg/kg ip) vs Naltrexone | 38.2 | 0.267 |
| Day 9 | RDC5 (0.6 mg/kg po)+Bupropion (20 mg/kg ip qd) vs RDC5 | 30.9 | 0.396 |
|  | RDC5 (0.6 mg/kg po)+Naltrexone (1 mg/kg ip) vs RDC5 | 66.7 | 0.069 |
|  | RDC5 (0.6 mg/kg po)+Bupropion (20 mg/kg ip qd) vs Bupropion | -42.7 | 0.233 |
|  | Bupropion (20 mg/kg ip qd)+Naltrexone (1 mg/kg ip) vs Bupropion | 51.7 | 0.146 |
|  | RDC5 (0.6 mg/kg po)+Naltrexone (1 mg/kg ip) vs Naltrexone | 1.2 | 0.974 |
|  | Bupropion (20 mg/kg ip qd)+Naltrexone (1 mg/kg ip) vs Naltrexone | 59.7 | 0.103 |
| Day 10 | RDC5 (0.6 mg/kg po)+Bupropion (20 mg/kg ip qd) vs RDC5 | 41.4 | 0.238 |
|  | RDC5 (0.6 mg/kg po)+Naltrexone (1 mg/kg ip) vs RDC5 | 61.2 | 0.083 |
|  | RDC5 (0.6 mg/kg po)+Bupropion (20 mg/kg ip qd) vs Bupropion | -15.9 | 0.651 |
|  | Bupropion (20 mg/kg ip qd)+Naltrexone (1 mg/kg ip) vs Bupropion | 46.3 | 0.188 |
|  | RDC5 (0.6 mg/kg po)+Naltrexone (1 mg/kg ip) vs Naltrexone | 0.2 | 0.997 |
|  | Bupropion (20 mg/kg ip qd)+Naltrexone (1 mg/kg ip) vs Naltrexone | 42.6 | 0.225 |
| Day 11 | RDC5 (0.6 mg/kg po)+Bupropion (20 mg/kg ip qd) vs RDC5 | -21.9 | 0.555 |
|  | RDC5 (0.6 mg/kg po)+Naltrexone (1 mg/kg ip) vs RDC5 | 24.2 | 0.514 |
|  | RDC5 (0.6 mg/kg po)+Bupropion (20 mg/kg ip qd) vs Bupropion | -107.0 | 0.005** |
|  | Bupropion (20 mg/kg ip qd)+Naltrexone (1 mg/kg ip) vs Bupropion | -4.8 | 0.897 |
|  | RDC5 (0.6 mg/kg po)+Naltrexone (1 mg/kg ip) vs Naltrexone | -73.4 | 0.050* |
|  | Bupropion (20 mg/kg ip qd)+Naltrexone (1 mg/kg ip) vs Naltrexone | -17.2 | 0.642 |
| Day 12 | RDC5 (0.6 mg/kg po)+Bupropion (20 mg/kg ip qd) vs RDC5 | -22.4 | 0.473 |
|  | RDC5 (0.6 mg/kg po)+Naltrexone (1 mg/kg ip) vs RDC5 | -19.8 | 0.525 |
|  | RDC5 (0.6 mg/kg po)+Bupropion (20 mg/kg ip qd) vs Bupropion | -83.8 | 0.009** |
|  | Bupropion (20 mg/kg ip qd)+Naltrexone (1 mg/kg ip) vs Bupropion | 12.4 | 0.691 |
|  | RDC5 (0.6 mg/kg po)+Naltrexone (1 mg/kg ip) vs Naltrexone | -67.5 | 0.033* |
|  | Bupropion (20 mg/kg ip qd)+Naltrexone (1 mg/kg ip) vs Naltrexone | 26.1 | 0.401 |
| Day 13 | RDC5 (0.6 mg/kg po)+Bupropion (20 mg/kg ip qd) vs RDC5 | 24.3 | 0.459 |
|  | RDC5 (0.6 mg/kg po)+Naltrexone (1 mg/kg ip) vs RDC5 | 45.5 | 0.168 |
|  | RDC5 (0.6 mg/kg po)+Bupropion (20 mg/kg ip qd) vs Bupropion | -59.4 | 0.076 |
|  | Bupropion (20 mg/kg ip qd)+Naltrexone (1 mg/kg ip) vs Bupropion | 4.0 | 0.902 |
|  | RDC5 (0.6 mg/kg po)+Naltrexone (1 mg/kg ip) vs Naltrexone | -10.9 | 0.741 |
|  | Bupropion (20 mg/kg ip qd)+Naltrexone (1 mg/kg ip) vs Naltrexone | 31.4 | 0.340 |
| Day 14 | RDC5 (0.6 mg/kg po)+Bupropion (20 mg/kg ip qd) vs RDC5 | 53.6 | 0.138 |
|  | RDC5 (0.6 mg/kg po)+Naltrexone (1 mg/kg ip) vs RDC5 | 108.1 | 0.003** |
|  | RDC5 (0.6 mg/kg po)+Bupropion (20 mg/kg ip qd) vs Bupropion | -21.5 | 0.552 |
|  | Bupropion (20 mg/kg ip qd)+Naltrexone (1 mg/kg ip) vs Bupropion | 28.4 | 0.428 |
|  | RDC5 (0.6 mg/kg po)+Naltrexone (1 mg/kg ip) vs Naltrexone | 39.9 | 0.268 |
|  | Bupropion (20 mg/kg ip qd)+Naltrexone (1 mg/kg ip) vs Naltrexone | 35.2 | 0.326 |

| Time | Comparison | Difference | p |
| --- | --- | --- | --- |
| Day 15 | RDC5 (0.6 mg/kg po)+Bupropion (20 mg/kg ip qd) vs RDC5 | -8.6 | 0.783 |
|  | RDC5 (0.6 mg/kg po)+Naltrexone (1 mg/kg ip) vs RDC5 | 38.4 | 0.220 |
|  | RDC5 (0.6 mg/kg po)+Bupropion (20 mg/kg ip qd) vs Bupropion | -72.0 | 0.024* |
|  | Bupropion (20 mg/kg ip qd)+Naltrexone (1 mg/kg ip) vs Bupropion | -47.1 | 0.134 |
|  | RDC5 (0.6 mg/kg po)+Naltrexone (1 mg/kg ip) vs Naltrexone | -23.0 | 0.462 |
|  | Bupropion (20 mg/kg ip qd)+Naltrexone (1 mg/kg ip) vs Naltrexone | -45.1 | 0.150 |
| Day 16 | RDC5 (0.6 mg/kg po)+Bupropion (20 mg/kg ip qd) vs RDC5 | -47.5 | 0.198 |
|  | RDC5 (0.6 mg/kg po)+Naltrexone (1 mg/kg ip) vs RDC5 | 0.9 | 0.980 |
|  | RDC5 (0.6 mg/kg po)+Bupropion (20 mg/kg ip qd) vs Bupropion | -54.4 | 0.145 |
|  | Bupropion (20 mg/kg ip qd)+Naltrexone (1 mg/kg ip) vs Bupropion | 37.9 | 0.304 |
|  | RDC5 (0.6 mg/kg po)+Naltrexone (1 mg/kg ip) vs Naltrexone | -58.7 | 0.113 |
|  | Bupropion (20 mg/kg ip qd)+Naltrexone (1 mg/kg ip) vs Naltrexone | -14.8 | 0.687 |
| Day 17 | RDC5 (0.6 mg/kg po)+Bupropion (20 mg/kg ip qd) vs RDC5 | 31.1 | 0.464 |
|  | RDC5 (0.6 mg/kg po)+Naltrexone (1 mg/kg ip) vs RDC5 | 71.1 | 0.096 |
|  | RDC5 (0.6 mg/kg po)+Bupropion (20 mg/kg ip qd) vs Bupropion | -16.0 | 0.708 |
|  | Bupropion (20 mg/kg ip qd)+Naltrexone (1 mg/kg ip) vs Bupropion | 91.0 | 0.034* |
|  | RDC5 (0.6 mg/kg po)+Naltrexone (1 mg/kg ip) vs Naltrexone | 45.2 | 0.287 |
|  | Bupropion (20 mg/kg ip qd)+Naltrexone (1 mg/kg ip) vs Naltrexone | 112.2 | 0.010** |
| Day 18 | RDC5 (0.6 mg/kg po)+Bupropion (20 mg/kg ip qd) vs RDC5 | 14.8 | 0.665 |
|  | RDC5 (0.6 mg/kg po)+Naltrexone (1 mg/kg ip) vs RDC5 | 59.9 | 0.083 |
|  | RDC5 (0.6 mg/kg po)+Bupropion (20 mg/kg ip qd) vs Bupropion | -79.3 | 0.024* |
|  | Bupropion (20 mg/kg ip qd)+Naltrexone (1 mg/kg ip) vs Bupropion | -9.1 | 0.790 |
|  | RDC5 (0.6 mg/kg po)+Naltrexone (1 mg/kg ip) vs Naltrexone | -12.4 | 0.717 |
|  | Bupropion (20 mg/kg ip qd)+Naltrexone (1 mg/kg ip) vs Naltrexone | 12.6 | 0.712 |
| Day 19 | RDC5 (0.6 mg/kg po)+Bupropion (20 mg/kg ip qd) vs RDC5 | -20.0 | 0.526 |
|  | RDC5 (0.6 mg/kg po)+Naltrexone (1 mg/kg ip) vs RDC5 | 36.8 | 0.243 |
|  | RDC5 (0.6 mg/kg po)+Bupropion (20 mg/kg ip qd) vs Bupropion | -92.9 | 0.004** |
|  | Bupropion (20 mg/kg ip qd)+Naltrexone (1 mg/kg ip) vs Bupropion | -46.4 | 0.143 |
|  | RDC5 (0.6 mg/kg po)+Naltrexone (1 mg/kg ip) vs Naltrexone | -8.9 | 0.776 |
|  | Bupropion (20 mg/kg ip qd)+Naltrexone (1 mg/kg ip) vs Naltrexone | -19.2 | 0.542 |
| Day 20 | RDC5 (0.6 mg/kg po)+Bupropion (20 mg/kg ip qd) vs RDC5 | -18.9 | 0.506 |
|  | RDC5 (0.6 mg/kg po)+Naltrexone (1 mg/kg ip) vs RDC5 | -7.3 | 0.796 |
|  | RDC5 (0.6 mg/kg po)+Bupropion (20 mg/kg ip qd) vs Bupropion | -32.8 | 0.254 |
|  | Bupropion (20 mg/kg ip qd)+Naltrexone (1 mg/kg ip) vs Bupropion | -11.2 | 0.692 |
|  | RDC5 (0.6 mg/kg po)+Naltrexone (1 mg/kg ip) vs Naltrexone | -18.7 | 0.510 |
|  | Bupropion (20 mg/kg ip qd)+Naltrexone (1 mg/kg ip) vs Naltrexone | -8.8 | 0.757 |
| Day 21 | RDC5 (0.6 mg/kg po)+Bupropion (20 mg/kg ip qd) vs RDC5 | -16.3 | 0.659 |
|  | RDC5 (0.6 mg/kg po)+Naltrexone (1 mg/kg ip) vs RDC5 | 3.3 | 0.928 |
|  | RDC5 (0.6 mg/kg po)+Bupropion (20 mg/kg ip qd) vs Bupropion | 4.4 | 0.906 |
|  | Bupropion (20 mg/kg ip qd)+Naltrexone (1 mg/kg ip) vs Bupropion | 32.9 | 0.375 |
|  | RDC5 (0.6 mg/kg po)+Naltrexone (1 mg/kg ip) vs Naltrexone | 0.2 | 0.996 |
|  | Bupropion (20 mg/kg ip qd)+Naltrexone (1 mg/kg ip) vs Naltrexone | 9.1 | 0.806 |

| Time | Comparison | Difference | p |
| --- | --- | --- | --- |
| Day 22 | RDC5 (0.6 mg/kg po)+Bupropion (20 mg/kg ip qd) vs RDC5 | 27.5 | 0.417 |
|  | RDC5 (0.6 mg/kg po)+Naltrexone (1 mg/kg ip) vs RDC5 | 41.7 | 0.220 |
|  | RDC5 (0.6 mg/kg po)+Bupropion (20 mg/kg ip qd) vs Bupropion | 0.9 | 0.979 |
|  | Bupropion (20 mg/kg ip qd)+Naltrexone (1 mg/kg ip) vs Bupropion | -18.4 | 0.587 |
|  | RDC5 (0.6 mg/kg po)+Naltrexone (1 mg/kg ip) vs Naltrexone | 10.9 | 0.747 |
|  | Bupropion (20 mg/kg ip qd)+Naltrexone (1 mg/kg ip) vs Naltrexone | -22.6 | 0.505 |
| Day 23 | RDC5 (0.6 mg/kg po)+Bupropion (20 mg/kg ip qd) vs RDC5 | 3.5 | 0.913 |
|  | RDC5 (0.6 mg/kg po)+Naltrexone (1 mg/kg ip) vs RDC5 | 12.8 | 0.693 |
|  | RDC5 (0.6 mg/kg po)+Bupropion (20 mg/kg ip qd) vs Bupropion | -35.3 | 0.281 |
|  | Bupropion (20 mg/kg ip qd)+Naltrexone (1 mg/kg ip) vs Bupropion | -30.9 | 0.341 |
|  | RDC5 (0.6 mg/kg po)+Naltrexone (1 mg/kg ip) vs Naltrexone | -41.9 | 0.197 |
|  | Bupropion (20 mg/kg ip qd)+Naltrexone (1 mg/kg ip) vs Naltrexone | -46.8 | 0.150 |
| Day 24 | RDC5 (0.6 mg/kg po)+Bupropion (20 mg/kg ip qd) vs RDC5 | 29.2 | 0.382 |
|  | RDC5 (0.6 mg/kg po)+Naltrexone (1 mg/kg ip) vs RDC5 | -0.3 | 0.992 |
|  | RDC5 (0.6 mg/kg po)+Bupropion (20 mg/kg ip qd) vs Bupropion | -27.0 | 0.422 |
|  | Bupropion (20 mg/kg ip qd)+Naltrexone (1 mg/kg ip) vs Bupropion | -11.6 | 0.727 |
|  | RDC5 (0.6 mg/kg po)+Naltrexone (1 mg/kg ip) vs Naltrexone | -4.4 | 0.896 |
|  | Bupropion (20 mg/kg ip qd)+Naltrexone (1 mg/kg ip) vs Naltrexone | 40.5 | 0.225 |
| Day 25 | RDC5 (0.6 mg/kg po)+Bupropion (20 mg/kg ip qd) vs RDC5 | 14.5 | 0.681 |
|  | RDC5 (0.6 mg/kg po)+Naltrexone (1 mg/kg ip) vs RDC5 | -35.9 | 0.309 |
|  | RDC5 (0.6 mg/kg po)+Bupropion (20 mg/kg ip qd) vs Bupropion | -7.0 | 0.844 |
|  | Bupropion (20 mg/kg ip qd)+Naltrexone (1 mg/kg ip) vs Bupropion | -9.9 | 0.779 |
|  | RDC5 (0.6 mg/kg po)+Naltrexone (1 mg/kg ip) vs Naltrexone | -49.9 | 0.159 |
|  | Bupropion (20 mg/kg ip qd)+Naltrexone (1 mg/kg ip) vs Naltrexone | -2.4 | 0.945 |
| Day 26 | RDC5 (0.6 mg/kg po)+Bupropion (20 mg/kg ip bid) vs RDC5 | 19.2 | 0.596 |
|  | RDC5 (0.6 mg/kg po)+Naltrexone (1 mg/kg ip) vs RDC5 | 8.7 | 0.809 |
|  | RDC5 (0.6 mg/kg po)+Bupropion (20 mg/kg ip bid) vs Bupropion | 0.4 | 0.992 |
|  | Bupropion (20 mg/kg ip bid)+Naltrexone (1 mg/kg ip) vs Bupropion | -17.1 | 0.636 |
|  | RDC5 (0.6 mg/kg po)+Naltrexone (1 mg/kg ip) vs Naltrexone | -28.9 | 0.426 |
|  | Bupropion (20 mg/kg ip bid)+Naltrexone (1 mg/kg ip) vs Naltrexone | -35.9 | 0.323 |
| Day 27 | RDC5 (0.6 mg/kg po)+Bupropion (20 mg/kg ip bid) vs RDC5 | 39.6 | 0.198 |
|  | RDC5 (0.6 mg/kg po)+Naltrexone (1 mg/kg ip) vs RDC5 | 67.1 | 0.031* |
|  | RDC5 (0.6 mg/kg po)+Bupropion (20 mg/kg ip bid) vs Bupropion | -25.2 | 0.415 |
|  | Bupropion (20 mg/kg ip bid)+Naltrexone (1 mg/kg ip) vs Bupropion | -51.9 | 0.102 |
|  | RDC5 (0.6 mg/kg po)+Naltrexone (1 mg/kg ip) vs Naltrexone | 7.0 | 0.818 |
|  | Bupropion (20 mg/kg ip bid)+Naltrexone (1 mg/kg ip) vs Naltrexone | -47.3 | 0.135 |
| Day 28 | RDC5 (0.6 mg/kg po)+Bupropion (20 mg/kg ip bid) vs RDC5 | -0.8 | 0.979 |
|  | RDC5 (0.6 mg/kg po)+Naltrexone (1 mg/kg ip) vs RDC5 | 16.8 | 0.561 |
|  | RDC5 (0.6 mg/kg po)+Bupropion (20 mg/kg ip bid) vs Bupropion | -0.3 | 0.993 |
|  | Bupropion (20 mg/kg ip bid)+Naltrexone (1 mg/kg ip) vs Bupropion | 2.1 | 0.943 |
|  | RDC5 (0.6 mg/kg po)+Naltrexone (1 mg/kg ip) vs Naltrexone | -6.5 | 0.823 |
|  | Bupropion (20 mg/kg ip bid)+Naltrexone (1 mg/kg ip) vs Naltrexone | -21.6 | 0.467 |

| Time | Comparison | Difference | p |
| --- | --- | --- | --- |
| Day 29 | RDC5 (0.6 mg/kg po)+Bupropion (20 mg/kg ip bid) vs RDC5 | 24.4 | 0.488 |
|  | RDC5 (0.6 mg/kg po)+Naltrexone (1 mg/kg ip) vs RDC5 | -45.4 | 0.198 |
|  | RDC5 (0.6 mg/kg po)+Bupropion (20 mg/kg ip bid) vs Bupropion | 20.3 | 0.567 |
|  | Bupropion (20 mg/kg ip bid)+Naltrexone (1 mg/kg ip) vs Bupropion | 12.6 | 0.721 |
|  | RDC5 (0.6 mg/kg po)+Naltrexone (1 mg/kg ip) vs Naltrexone | -10.6 | 0.762 |
|  | Bupropion (20 mg/kg ip bid)+Naltrexone (1 mg/kg ip) vs Naltrexone | 51.4 | 0.145 |
| Day 30 | RDC5 (0.6 mg/kg po)+Bupropion (20 mg/kg ip bid) vs RDC5 | 8.5 | 0.849 |
|  | RDC5 (0.6 mg/kg po)+Naltrexone (1 mg/kg ip) vs RDC5 | 32.7 | 0.462 |
|  | RDC5 (0.6 mg/kg po)+Bupropion (20 mg/kg ip bid) vs Bupropion | 14.4 | 0.749 |
|  | Bupropion (20 mg/kg ip bid)+Naltrexone (1 mg/kg ip) vs Bupropion | 4.9 | 0.912 |
|  | RDC5 (0.6 mg/kg po)+Naltrexone (1 mg/kg ip) vs Naltrexone | 41.2 | 0.355 |
|  | Bupropion (20 mg/kg ip bid)+Naltrexone (1 mg/kg ip) vs Naltrexone | 7.5 | 0.865 |
| Day 31 | RDC5 (0.6 mg/kg po)+Bupropion (20 mg/kg ip bid) vs RDC5 | 53.7 | 0.213 |
|  | RDC5 (0.6 mg/kg po)+Naltrexone (1 mg/kg ip) vs RDC5 | 85.2 | 0.050* |
|  | RDC5 (0.6 mg/kg po)+Bupropion (20 mg/kg ip bid) vs Bupropion | 0.9 | 0.984 |
|  | Bupropion (20 mg/kg ip bid)+Naltrexone (1 mg/kg ip) vs Bupropion | 14.1 | 0.742 |
|  | RDC5 (0.6 mg/kg po)+Naltrexone (1 mg/kg ip) vs Naltrexone | 31.9 | 0.457 |
|  | Bupropion (20 mg/kg ip bid)+Naltrexone (1 mg/kg ip) vs Naltrexone | 13.6 | 0.751 |
| Day 32 | RDC5 (0.6 mg/kg po)+Bupropion (20 mg/kg ip bid) vs RDC5 | 47.3 | 0.229 |
|  | RDC5 (0.6 mg/kg po)+Naltrexone (1 mg/kg ip) vs RDC5 | 81.4 | 0.040* |
|  | RDC5 (0.6 mg/kg po)+Bupropion (20 mg/kg ip bid) vs Bupropion | -13.5 | 0.733 |
|  | Bupropion (20 mg/kg ip bid)+Naltrexone (1 mg/kg ip) vs Bupropion | -22.4 | 0.567 |
|  | RDC5 (0.6 mg/kg po)+Naltrexone (1 mg/kg ip) vs Naltrexone | -6.0 | 0.877 |
|  | Bupropion (20 mg/kg ip bid)+Naltrexone (1 mg/kg ip) vs Naltrexone | -49.1 | 0.212 |
| Day 33 | RDC5 (0.6 mg/kg po)+Bupropion (20 mg/kg ip bid) vs RDC5 | 0.5 | 0.991 |
|  | RDC5 (0.6 mg/kg po)+Naltrexone (1 mg/kg ip) vs RDC5 | 28.9 | 0.476 |
|  | RDC5 (0.6 mg/kg po)+Bupropion (20 mg/kg ip bid) vs Bupropion | -13.4 | 0.736 |
|  | Bupropion (20 mg/kg ip bid)+Naltrexone (1 mg/kg ip) vs Bupropion | -21.9 | 0.580 |
|  | RDC5 (0.6 mg/kg po)+Naltrexone (1 mg/kg ip) vs Naltrexone | 24.5 | 0.556 |
|  | Bupropion (20 mg/kg ip bid)+Naltrexone (1 mg/kg ip) vs Naltrexone | -12.4 | 0.759 |
| Day 34 | RDC5 (0.6 mg/kg po)+Bupropion (20 mg/kg ip bid) vs RDC5 | 26.8 | 0.439 |
|  | RDC5 (0.6 mg/kg po)+Naltrexone (1 mg/kg ip) vs RDC5 | -4.6 | 0.893 |
|  | RDC5 (0.6 mg/kg po)+Bupropion (20 mg/kg ip bid) vs Bupropion | 32.2 | 0.356 |
|  | Bupropion (20 mg/kg ip bid)+Naltrexone (1 mg/kg ip) vs Bupropion | -5.9 | 0.865 |
|  | RDC5 (0.6 mg/kg po)+Naltrexone (1 mg/kg ip) vs Naltrexone | 66.7 | 0.056 |
|  | Bupropion (20 mg/kg ip bid)+Naltrexone (1 mg/kg ip) vs Naltrexone | 60.1 | 0.085 |
| Day 35 | RDC5 (0.6 mg/kg po)+Bupropion (20 mg/kg ip bid) vs RDC5 | -24.7 | 0.492 |
|  | RDC5 (0.6 mg/kg po)+Naltrexone (1 mg/kg ip) vs RDC5 | -1.8 | 0.960 |
|  | RDC5 (0.6 mg/kg po)+Bupropion (20 mg/kg ip bid) vs Bupropion | 20.2 | 0.577 |
|  | Bupropion (20 mg/kg ip bid)+Naltrexone (1 mg/kg ip) vs Bupropion | -40.5 | 0.261 |
|  | RDC5 (0.6 mg/kg po)+Naltrexone (1 mg/kg ip) vs Naltrexone | 38.8 | 0.280 |
|  | Bupropion (20 mg/kg ip bid)+Naltrexone (1 mg/kg ip) vs Naltrexone | -44.7 | 0.214 |

| Time | Comparison | Difference | p |
| --- | --- | --- | --- |
| Day 36 | RDC5 (0.6 mg/kg po)+Bupropion (20 mg/kg ip bid) vs RDC5 | -4.1 | 0.893 |
|  | RDC5 (0.6 mg/kg po)+Naltrexone (1 mg/kg ip) vs RDC5 | 20.6 | 0.506 |
|  | RDC5 (0.6 mg/kg po)+Bupropion (20 mg/kg ip bid) vs Bupropion | -3.4 | 0.912 |
|  | Bupropion (20 mg/kg ip bid)+Naltrexone (1 mg/kg ip) vs Bupropion | -27.1 | 0.382 |
|  | RDC5 (0.6 mg/kg po)+Naltrexone (1 mg/kg ip) vs Naltrexone | 40.7 | 0.189 |
|  | Bupropion (20 mg/kg ip bid)+Naltrexone (1 mg/kg ip) vs Naltrexone | -7.6 | 0.806 |
| Day 37 | RDC5 (0.6 mg/kg po)+Bupropion (20 mg/kg ip bid) vs RDC5 | -24.7 | 0.410 |
|  | RDC5 (0.6 mg/kg po)+Naltrexone (1 mg/kg ip) vs RDC5 | 23.0 | 0.440 |
|  | RDC5 (0.6 mg/kg po)+Bupropion (20 mg/kg ip bid) vs Bupropion | -26.3 | 0.383 |
|  | Bupropion (20 mg/kg ip bid)+Naltrexone (1 mg/kg ip) vs Bupropion | -2.8 | 0.924 |
|  | RDC5 (0.6 mg/kg po)+Naltrexone (1 mg/kg ip) vs Naltrexone | 40.8 | 0.174 |
|  | Bupropion (20 mg/kg ip bid)+Naltrexone (1 mg/kg ip) vs Naltrexone | 16.6 | 0.579 |
| Day 38 | RDC5 (0.6 mg/kg po)+Bupropion (20 mg/kg ip bid) vs RDC5 | -16.3 | 0.633 |
|  | RDC5 (0.6 mg/kg po)+Naltrexone (1 mg/kg ip) vs RDC5 | 5.9 | 0.863 |
|  | RDC5 (0.6 mg/kg po)+Bupropion (20 mg/kg ip bid) vs Bupropion | -25.3 | 0.461 |
|  | Bupropion (20 mg/kg ip bid)+Naltrexone (1 mg/kg ip) vs Bupropion | -14.6 | 0.667 |
|  | RDC5 (0.6 mg/kg po)+Naltrexone (1 mg/kg ip) vs Naltrexone | 33.6 | 0.324 |
|  | Bupropion (20 mg/kg ip bid)+Naltrexone (1 mg/kg ip) vs Naltrexone | 22.2 | 0.515 |
| Day 39 | RDC5 (0.6 mg/kg po)+Bupropion (20 mg/kg ip bid) vs RDC5 | -39.7 | 0.290 |
|  | RDC5 (0.6 mg/kg po)+Naltrexone (1 mg/kg ip) vs RDC5 | 40.1 | 0.271 |
|  | RDC5 (0.6 mg/kg po)+Bupropion (20 mg/kg ip bid) vs Bupropion | -32.8 | 0.387 |
|  | Bupropion (20 mg/kg ip bid)+Naltrexone (1 mg/kg ip) vs Bupropion | -33.7 | 0.355 |
|  | RDC5 (0.6 mg/kg po)+Naltrexone (1 mg/kg ip) vs Naltrexone | 74.6 | 0.043* |
|  | Bupropion (20 mg/kg ip bid)+Naltrexone (1 mg/kg ip) vs Naltrexone | -6.2 | 0.865 |
| Day 40 | RDC5 (0.6 mg/kg po)+Bupropion (20 mg/kg ip bid) vs RDC5 | -96.5 | 0.010* |
|  | RDC5 (0.6 mg/kg po)+Naltrexone (1 mg/kg ip) vs RDC5 | -4.9 | 0.894 |
|  | RDC5 (0.6 mg/kg po)+Bupropion (20 mg/kg ip bid) vs Bupropion | -64.5 | 0.085 |
|  | Bupropion (20 mg/kg ip bid)+Naltrexone (1 mg/kg ip) vs Bupropion | -38.3 | 0.299 |
|  | RDC5 (0.6 mg/kg po)+Naltrexone (1 mg/kg ip) vs Naltrexone | 13.8 | 0.706 |
|  | Bupropion (20 mg/kg ip bid)+Naltrexone (1 mg/kg ip) vs Naltrexone | -51.5 | 0.163 |
| Day 41 | RDC5 (0.6 mg/kg po)+Bupropion (20 mg/kg ip bid) vs RDC5 | -103.1 | <0.001*** |
|  | RDC5 (0.6 mg/kg po)+Naltrexone (1 mg/kg ip) vs RDC5 | -24.0 | 0.423 |
|  | RDC5 (0.6 mg/kg po)+Bupropion (20 mg/kg ip bid) vs Bupropion | -64.0 | 0.037* |
|  | Bupropion (20 mg/kg ip bid)+Naltrexone (1 mg/kg ip) vs Bupropion | -17.0 | 0.572 |
|  | RDC5 (0.6 mg/kg po)+Naltrexone (1 mg/kg ip) vs Naltrexone | 6.1 | 0.837 |
|  | Bupropion (20 mg/kg ip bid)+Naltrexone (1 mg/kg ip) vs Naltrexone | -25.9 | 0.387 |
| Day 42 | RDC5 (0.6 mg/kg po)+Bupropion (20 mg/kg ip bid) vs RDC5 | -27.7 | 0.360 |
|  | RDC5 (0.6 mg/kg po)+Naltrexone (1 mg/kg ip) vs RDC5 | 19.3 | 0.522 |
|  | RDC5 (0.6 mg/kg po)+Bupropion (20 mg/kg ip bid) vs Bupropion | -11.5 | 0.705 |
|  | Bupropion (20 mg/kg ip bid)+Naltrexone (1 mg/kg ip) vs Bupropion | 10.2 | 0.735 |
|  | RDC5 (0.6 mg/kg po)+Naltrexone (1 mg/kg ip) vs Naltrexone | 26.7 | 0.376 |
|  | Bupropion (20 mg/kg ip bid)+Naltrexone (1 mg/kg ip) vs Naltrexone | 1.5 | 0.960 |

Multiple comparisons are by the multiple t test. *p<0.05, ***p<0.001.

#### Average daily food intake (kJ/day)

Treatment n Mean SEM Difference % change p from vehicle

| Week 1 | Vehicle 4 ml/kg po +2 ml/kg ip | 12 | 356.9 | 13.7 |  | | |
| --- | --- | --- | --- | --- | --- | --- | --- |
| (Day 1-7) | RDC5 0.6 mg/kg po | 10 | 315.6 | 10.4 | -41.3 | -11.6 | 0.028* |
|  | Bupropion 20 mg/kg ip qd | 10 | 361.0 | 10.2 | 4.1 | 1.2 | 0.823 |
|  | Naltrexone 1 mg/kg ip | 10 | 342.9 | 16.3 | -14.0 | -3.9 | 0.450 |
|  | RDC5 0.6 mg/kg po+Bupropion 20 mg/kg ip qd | 10 | 323.4 | 11.0 | -33.4 | -9.4 | 0.074 |
|  | RDC5 0.6 mg/kg po+Naltrexone 1 mg/kg ip | 10 | 325.8 | 10.9 | -31.1 | -8.7 | 0.095 |
|  | Bupropion 20 mg/kg ip qd+Naltrexone 1 mg/kg ip | 10 | 363.0 | 15.5 | 6.2 | 1.7 | 0.738 |
| Sibutramine 5 mg/kg po 10 | | | 186.4 | 15.4 | -170.4 | -47.8 <0.001*** | |
| Week 2 | Vehicle 4 ml/kg po +2 ml/kg ip | 12 | 348.0 | 18.5 |  |  |  |
| (Day 8-14) | RDC5 0.6 mg/kg po | 10 | 309.3 | 17.8 | -38.6 | -11.1 | 0.080 |
|  | Bupropion 20 mg/kg ip qd | 10 | 382.2 | 11.3 | 34.3 | 9.9 | 0.121 |
|  | Naltrexone 1 mg/kg ip | 10 | 377.2 | 17.0 | 29.2 | 8.4 | 0.184 |
|  | RDC5 0.6 mg/kg po+Bupropion 20 mg/kg ip qd | 10 | 329.1 | 16.2 | -18.9 | -5.4 | 0.390 |
|  | RDC5 0.6 mg/kg po+Naltrexone 1 mg/kg ip | 10 | 353.8 | 13.2 | 5.9 | 1.7 | 0.788 |
|  | Bupropion 20 mg/kg ip qd+Naltrexone 1 mg/kg ip | 10 | 407.8 | 15.3 | 59.8 | 17.2 | 0.008** |
|  | Sibutramine 5 mg/kg po | 10 | 308.5 | 12.6 | -39.5 | -11.3 | 0.074 |
| Week 3 | Vehicle 4 ml/kg po +2 ml/kg ip | 12 | 354.3 | 11.8 |  |  |  |
| (Day 15-21) | RDC5 0.6 mg/kg po | 10 | 350.3 | 13.7 | -4.1 | -1.2 | 0.846 |
|  | Bupropion 20 mg/kg ip qd | 10 | 389.9 | 11.0 | 35.6 | 10.0 | 0.095 |
|  | Naltrexone 1 mg/kg ip | 10 | 390.2 | 22.0 | 35.8 | 10.1 | 0.091 |
|  | RDC5 0.6 mg/kg po+Bupropion 20 mg/kg ip qd | 10 | 340.9 | 19.4 | -13.4 | -3.8 | 0.524 |
|  | RDC5 0.6 mg/kg po+Naltrexone 1 mg/kg ip | 10 | 379.3 | 14.6 | 25.0 | 7.0 | 0.237 |
|  | Bupropion 20 mg/kg ip qd+Naltrexone 1 mg/kg ip | 10 | 396.8 | 15.1 | 42.4 | 12.0 | 0.047* |
|  | Sibutramine 5 mg/kg po | 10 | 348.7 | 9.9 | -5.7 | -1.6 | 0.788 |
| Week 4 | Vehicle 4 ml/kg po +2 ml/kg ip | 12 | 352.0 | 13.2 |  |  |  |
| (Day 22-25) | RDC5 0.6 mg/kg po | 10 | 365.7 | 17.4 | 13.7 | 3.9 | 0.536 |
|  | Bupropion 20 mg/kg ip bid qd | 10 | 401.4 | 12.1 | 49.4 | 14.0 | 0.028* |
|  | Naltrexone 1 mg/kg ip | 10 | 391.5 | 20.1 | 39.5 | 11.2 | 0.077 |
|  | RDC5 0.6 mg/kg po+Bupropion 20 mg/kg ip qd | 10 | 384.4 | 25.0 | 32.4 | 9.2 | 0.147 |
|  | RDC5 0.6 mg/kg po+Naltrexone 1 mg/kg ip | 10 | 370.2 | 11.4 | 18.2 | 5.2 | 0.411 |
|  | Bupropion 20 mg/kg ip qd+Naltrexone 1 mg/kg ip | 10 | 383.7 | 10.8 | 31.7 | 9.0 | 0.154 |
|  | Sibutramine 5 mg/kg po | 10 | 340.9 | 11.7 | -11.2 | -3.2 | 0.615 |
| Week 5 | Vehicle 4 ml/kg po +2 ml/kg ip | 12 | 329.8 | 18.8 |  |  |  |
| (Day 26-35) | RDC5 0.6 mg/kg po | 10 | 341.0 | 15.1 | 11.2 | 3.4 | 0.592 |
|  | Bupropion 20 mg/kg ip bid | 10 | 356.7 | 14.2 | 27.0 | 8.2 | 0.202 |
|  | Naltrexone 1 mg/kg ip | 10 | 351.9 | 10.4 | 22.1 | 6.7 | 0.293 |
|  | RDC5 0.6 mg/kg po+Bupropion 20 mg/kg ip bid | 10 | 360.5 | 15.3 | 30.8 | 9.3 | 0.146 |
|  | RDC5 0.6 mg/kg po+Naltrexone 1 mg/kg ip | 10 | 368.6 | 12.0 | 38.8 | 11.8 | 0.067 |
|  | Bupropion 20 mg/kg ip bid+Naltrexone 1 mg/kg ip | 10 | 345.9 | 20.2 | 16.1 | 4.9 | 0.442 |
|  | Sibutramine 5 mg/kg po | 10 | 330.3 | 7.8 | 0.5 | 0.1 | 0.982 |
| Week 6 | Vehicle 4 ml/kg po +2 ml/kg ip | 12 | 316.9 | 11.7 |  |  |  |
| (Day 36-42) | RDC5 0.6 mg/kg po | 10 | 354.1 | 13.0 | 37.2 | 11.7 | 0.037* |
|  | Bupropion 20 mg/kg ip bid | 10 | 342.1 | 13.5 | 25.2 | 7.9 | 0.156 |
|  | Naltrexone 1 mg/kg ip | 10 | 331.7 | 11.9 | 14.8 | 4.7 | 0.399 |
|  | RDC5 0.6 mg/kg po+Bupropion 20 mg/kg ip bid | 10 | 309.2 | 12.6 | -7.7 | -2.4 | 0.663 |
|  | RDC5 0.6 mg/kg po+Naltrexone 1 mg/kg ip | 10 | 365.5 | 13.0 | 48.6 | 15.3 | 0.007** |
|  | Bupropion 20 mg/kg ip bid+Naltrexone 1 mg/kg ip | 10 | 324.4 | 13.1 | 7.6 | 2.4 | 0.667 |
|  | Sibutramine 5 mg/kg po | 10 | 301.5 | 11.9 | -15.4 | -4.9 | 0.382 |

Means are adjusted for differences between the treatment groups at baseline (average of Days -6 to 0). SEM are calculated from the residuals of the statistical model. Multiple comparisons against vehicle are by the multiple t test. *p<0.05, **p<0.01,

***p<0.001.

Treatment n Mean SEM Difference % change p from vehicle

| Weeks 1-4 Vehicle 4 ml/kg po +2 ml/kg ip | 12 | 352.9 | 11.9 |  | | |
| --- | --- | --- | --- | --- | --- | --- |
| (Day 1-25) RDC5 0.6 mg/kg po | 10 | 331.7 | 11.3 | -21.2 | -6.0 | 0.189 |
| Bupropion 20 mg/kg ip qd | 10 | 381.5 | 7.8 | 28.6 | 8.1 | 0.078 |
| Naltrexone 1 mg/kg ip | 10 | 373.6 | 13.8 | 20.7 | 5.9 | 0.199 |
| RDC5 0.6 mg/kg po+Bupropion 20 mg/kg ip qd | 10 | 339.7 | 13.2 | -13.2 | -3.7 | 0.411 |
| RDC5 0.6 mg/kg po+Naltrexone 1 mg/kg ip | 10 | 355.7 | 9.0 | 2.9 | 0.8 | 0.858 |
| Bupropion 20 mg/kg ip qd+Naltrexone 1 mg/kg ip | 10 | 388.3 | 12.1 | 35.4 | 10.0 | 0.029* |
| Sibutramine 5 mg/kg po | 10 | 290.7 | 10.9 | -62.1 | -17.6 | <0.001*** |
| Weeks 5-6 Vehicle 4 ml/kg po +2 ml/kg ip | 12 | 325.2 | 12.6 |  |  |  |
| (Day 26-42) RDC5 0.6 mg/kg po | 10 | 346.4 | 8.7 | 21.1 | 6.5 | 0.176 |
| Bupropion 20 mg/kg ip bid | 10 | 350.7 | 11.4 | 25.5 | 7.8 | 0.105 |
| Naltrexone 1 mg/kg ip | 10 | 343.6 | 9.0 | 18.4 | 5.6 | 0.239 |
| RDC5 0.6 mg/kg po+Bupropion 20 mg/kg ip bid | 10 | 340.0 | 12.0 | 14.8 | 4.5 | 0.344 |
| RDC5 0.6 mg/kg po+Naltrexone 1 mg/kg ip | 10 | 367.3 | 10.4 | 42.0 | 12.9 | 0.008** |
| Bupropion 20 mg/kg ip bid+Naltrexone 1 mg/kg ip | 10 | 336.1 | 13.8 | 10.9 | 3.3 | 0.483 |
| Sibutramine 5 mg/kg po | 10 | 318.4 | 8.9 | -6.8 | -2.1 | 0.659 |
| Weeks 1-5 Vehicle 4 ml/kg po +2 ml/kg ip | 12 | 346.6 | 12.5 |  |  |  |
| (Day 1-35) RDC5 0.6 mg/kg po | 10 | 334.4 | 11.6 | -12.2 | -3.5 | 0.445 |
| Bupropion 20 mg/kg ip qd/bid | 10 | 374.5 | 8.9 | 27.8 | 8.0 | 0.085 |
| Naltrexone 1 mg/kg ip | 10 | 367.5 | 11.3 | 20.8 | 6.0 | 0.194 |
| RDC5 0.6 mg/kg po+Bupropion 20 mg/kg ip qd/bid | 10 | 345.6 | 12.6 | -1.0 | -0.3 | 0.950 |
| RDC5 0.6 mg/kg po+Naltrexone 1 mg/kg ip | 10 | 359.3 | 9.2 | 12.7 | 3.7 | 0.427 |
| Bupropion 20 mg/kg ip qd/bid +Naltrexone 1 mg/kg ip | 10 | 376.3 | 14.0 | 29.7 | 8.6 | 0.066 |
| Sibutramine 5 mg/kg po | 10 | 301.8 | 9.5 | -44.8 | -12.9 | 0.006** |
| Overall Vehicle 4 ml/kg po +2 ml/kg ip | 12 | 341.7 | 11.3 |  |  |  |
| (Day 1-42) RDC5 0.6 mg/kg po | 10 | 337.7 | 9.6 | -4.1 | -1.2 | 0.774 |
| Bupropion 20 mg/kg ip qd/bid | 10 | 369.1 | 8.7 | 27.3 | 8.0 | 0.059 |
| Naltrexone 1 mg/kg ip | 10 | 361.4 | 9.5 | 19.7 | 5.8 | 0.169 |
| RDC5 0.6 mg/kg po+Bupropion 20 mg/kg ip qd/bid | 10 | 339.7 | 11.6 | -2.0 | -0.6 | 0.887 |
| RDC5 0.6 mg/kg po+Naltrexone 1 mg/kg ip | 10 | 360.4 | 8.3 | 18.6 | 5.4 | 0.193 |
| Bupropion 20 mg/kg ip qd/bid +Naltrexone 1 mg/kg ip | 10 | 367.5 | 11.8 | 25.7 | 7.5 | 0.074 |
| Sibutramine 5 mg/kg po | 10 | 301.8 | 9.4 | -39.9 | -11.7 | 0.006** |

Means are adjusted for differences between the treatment groups at baseline (average of Days -6 to 0). SEM are calculated from the residuals of the statistical model. Multiple comparisons against vehicle are by the multiple t test. *p<0.05, **p<0.01,

***p<0.001.

| Time | Comparison | Difference | p |
| --- | --- | --- | --- |
| Week 1 | RDC5 (0.6 mg/kg po)+Bupropion (20 mg/kg ip qd) vs RDC5 | 7.8 | 0.684 |
| (Day 1-7) | RDC5 (0.6 mg/kg po)+Naltrexone (1 mg/kg ip) vs RDC5 | 10.2 | 0.597 |
|  | RDC5 (0.6 mg/kg po)+Bupropion (20 mg/kg ip qd) vs Bupropion | -37.6 | 0.056 |
|  | Bupropion (20 mg/kg ip qd)+Naltrexone (1 mg/kg ip) vs Bupropion | 2.0 | 0.917 |
|  | RDC5 (0.6 mg/kg po)+Naltrexone (1 mg/kg ip) vs Naltrexone | -17.1 | 0.376 |
|  | Bupropion (20 mg/kg ip qd)+Naltrexone (1 mg/kg ip) vs Naltrexone | 20.1 | 0.298 |
| Week 2 | RDC5 (0.6 mg/kg po)+Bupropion (20 mg/kg ip qd) vs RDC5 | 19.8 | 0.388 |
| (Day 8-14) | RDC5 (0.6 mg/kg po)+Naltrexone (1 mg/kg ip) vs RDC5 | 44.5 | 0.054 |
|  | RDC5 (0.6 mg/kg po)+Bupropion (20 mg/kg ip qd) vs Bupropion | -53.1 | 0.023* |
|  | Bupropion (20 mg/kg ip qd)+Naltrexone (1 mg/kg ip) vs Bupropion | 25.5 | 0.266 |
|  | RDC5 (0.6 mg/kg po)+Naltrexone (1 mg/kg ip) vs Naltrexone | -23.4 | 0.308 |
|  | Bupropion (20 mg/kg ip qd)+Naltrexone (1 mg/kg ip) vs Naltrexone | 30.6 | 0.183 |
| Week 3 | RDC5 (0.6 mg/kg po)+Bupropion (20 mg/kg ip qd) vs RDC5 | -9.3 | 0.671 |
| (Day 15-21) | RDC5 (0.6 mg/kg po)+Naltrexone (1 mg/kg ip) vs RDC5 | 29.0 | 0.189 |
|  | RDC5 (0.6 mg/kg po)+Bupropion (20 mg/kg ip qd) vs Bupropion | -49.0 | 0.030* |
|  | Bupropion (20 mg/kg ip qd)+Naltrexone (1 mg/kg ip) vs Bupropion | 6.9 | 0.755 |
|  | RDC5 (0.6 mg/kg po)+Naltrexone (1 mg/kg ip) vs Naltrexone | -10.9 | 0.620 |
|  | Bupropion (20 mg/kg ip qd)+Naltrexone (1 mg/kg ip) vs Naltrexone | 6.6 | 0.765 |
| Week 4 | RDC5 (0.6 mg/kg po)+Bupropion (20 mg/kg ip qd) vs RDC5 | 18.7 | 0.419 |
| (Day 22-25) | RDC5 (0.6 mg/kg po)+Naltrexone (1 mg/kg ip) vs RDC5 | 4.6 | 0.844 |
|  | RDC5 (0.6 mg/kg po)+Bupropion (20 mg/kg ip qd) vs Bupropion | -17.0 | 0.466 |
|  | Bupropion (20 mg/kg ip qd)+Naltrexone (1 mg/kg ip) vs Bupropion | -17.7 | 0.445 |
|  | RDC5 (0.6 mg/kg po)+Naltrexone (1 mg/kg ip) vs Naltrexone | -21.3 | 0.358 |
|  | Bupropion (20 mg/kg ip qd)+Naltrexone (1 mg/kg ip) vs Naltrexone | -7.8 | 0.735 |
| Week 5 | RDC5 (0.6 mg/kg po)+Bupropion (20 mg/kg ip bid) vs RDC5 | 19.5 | 0.374 |
| (Day 26-35) | RDC5 (0.6 mg/kg po)+Naltrexone (1 mg/kg ip) vs RDC5 | 27.5 | 0.211 |
|  | RDC5 (0.6 mg/kg po)+Bupropion (20 mg/kg ip bid) vs Bupropion | 3.8 | 0.864 |
|  | Bupropion (20 mg/kg ip bid)+Naltrexone (1 mg/kg ip) vs Bupropion | -10.8 | 0.621 |
|  | RDC5 (0.6 mg/kg po)+Naltrexone (1 mg/kg ip) vs Naltrexone | 16.7 | 0.448 |
|  | Bupropion (20 mg/kg ip bid)+Naltrexone (1 mg/kg ip) vs Naltrexone | -6.0 | 0.785 |
| Week 6 | RDC5 (0.6 mg/kg po)+Bupropion (20 mg/kg ip bid) vs RDC5 | -44.9 | 0.017* |
| (Day 36-42) | RDC5 (0.6 mg/kg po)+Naltrexone (1 mg/kg ip) vs RDC5 | 11.4 | 0.533 |
|  | RDC5 (0.6 mg/kg po)+Bupropion (20 mg/kg ip bid) vs Bupropion | -32.8 | 0.079 |
|  | Bupropion (20 mg/kg ip bid)+Naltrexone (1 mg/kg ip) vs Bupropion | -17.6 | 0.339 |
|  | RDC5 (0.6 mg/kg po)+Naltrexone (1 mg/kg ip) vs Naltrexone | 33.8 | 0.069 |
|  | Bupropion (20 mg/kg ip bid)+Naltrexone (1 mg/kg ip) vs Naltrexone | -7.3 | 0.691 |

Multiple comparisons are by the multiple t test. *p<0.05.

| Time | Comparison | Difference | p |
| --- | --- | --- | --- |
| Weeks 1-4 | RDC5 (0.6 mg/kg po)+Bupropion (20 mg/kg ip qd) vs RDC5 | 8.0 | 0.634 |
| (Day 1-25) | RDC5 (0.6 mg/kg po)+Naltrexone (1 mg/kg ip) vs RDC5 | 24.0 | 0.153 |
|  | RDC5 (0.6 mg/kg po)+Bupropion (20 mg/kg ip qd) vs Bupropion | -41.8 | 0.015* |
|  | Bupropion (20 mg/kg ip qd)+Naltrexone (1 mg/kg ip) vs Bupropion | 6.8 | 0.684 |
|  | RDC5 (0.6 mg/kg po)+Naltrexone (1 mg/kg ip) vs Naltrexone | -17.8 | 0.288 |
|  | Bupropion (20 mg/kg ip qd)+Naltrexone (1 mg/kg ip) vs Naltrexone | 14.8 | 0.379 |
| Weeks 5-6 | RDC5 (0.6 mg/kg po)+Bupropion (20 mg/kg ip bid) vs RDC5 | -6.4 | 0.694 |
| (Day 26-42) | RDC5 (0.6 mg/kg po)+Naltrexone (1 mg/kg ip) vs RDC5 | 20.9 | 0.200 |
|  | RDC5 (0.6 mg/kg po)+Bupropion (20 mg/kg ip bid) vs Bupropion | -10.7 | 0.513 |
|  | Bupropion (20 mg/kg ip bid)+Naltrexone (1 mg/kg ip) vs Bupropion | -14.6 | 0.371 |
|  | RDC5 (0.6 mg/kg po)+Naltrexone (1 mg/kg ip) vs Naltrexone | 23.6 | 0.148 |
|  | Bupropion (20 mg/kg ip bid)+Naltrexone (1 mg/kg ip) vs Naltrexone | -7.5 | 0.645 |
| Weeks 1-5 | RDC5 (0.6 mg/kg po)+Bupropion (20 mg/kg ip qd/bid) vs RDC5 | 11.2 | 0.502 |
| (Day 1-35) | RDC5 (0.6 mg/kg po)+Naltrexone (1 mg/kg ip) vs RDC5 | 24.9 | 0.138 |
|  | RDC5 (0.6 mg/kg po)+Bupropion (20 mg/kg ip qd/bid) vs Bupropion | -28.9 | 0.089 |
|  | Bupropion (20 mg/kg ip qd/bid)+Naltrexone (1 mg/kg ip) vs Bupropion | 1.8 | 0.913 |
|  | RDC5 (0.6 mg/kg po)+Naltrexone (1 mg/kg ip) vs Naltrexone | -8.1 | 0.626 |
|  | Bupropion (20 mg/kg ip qd/bid)+Naltrexone (1 mg/kg ip) vs Naltrexone | 8.8 | 0.597 |
| Overall | RDC5 (0.6 mg/kg po)+Bupropion (20 mg/kg ip qd/bid) vs RDC5 | 2.0 | 0.890 |
| (Day 1-42) | RDC5 (0.6 mg/kg po)+Naltrexone (1 mg/kg ip) vs RDC5 | 22.7 | 0.130 |
|  | RDC5 (0.6 mg/kg po)+Bupropion (20 mg/kg ip qd/bid) vs Bupropion | -29.3 | 0.053 |
|  | Bupropion (20 mg/kg ip qd/bid)+Naltrexone (1 mg/kg ip) vs Bupropion | -1.6 | 0.916 |
|  | RDC5 (0.6 mg/kg po)+Naltrexone (1 mg/kg ip) vs Naltrexone | -1.1 | 0.943 |
|  | Bupropion (20 mg/kg ip qd/bid)+Naltrexone (1 mg/kg ip) vs Naltrexone | 6.1 | 0.683 |

Multiple comparisons are by the multiple t test. *p<0.05.

#### treatments to their individual constituents (Weeks 1 to 6) Cumulative food intake (kJ) on each day (Week 1)

Treatment n Mean SEM Difference % change p from vehicle

| Day 1 | Vehicle 4 ml/kg po +2 ml/kg ip | 12 | 345 | 25 |  | | |
| --- | --- | --- | --- | --- | --- | --- | --- |
|  | RDC5 0.6 mg/kg po | 10 | 321 | 26 | -25 | -7.2 | 0.333 |
|  | Bupropion 20 mg/kg ip qd | 10 | 336 | 15 | -9 | -2.6 | 0.730 |
|  | Naltrexone 1 mg/kg ip | 10 | 298 | 17 | -47 | -13.7 | 0.069 |
|  | RDC5 0.6 mg/kg po+Bupropion 20 mg/kg ip qd | 10 | 314 | 19 | -32 | -9.1 | 0.220 |
|  | RDC5 0.6 mg/kg po+Naltrexone 1 mg/kg ip | 10 | 299 | 12 | -47 | -13.5 | 0.071 |
|  | Bupropion 20 mg/kg ip qd+Naltrexone 1 mg/kg ip | 10 | 326 | 14 | -20 | -5.7 | 0.440 |
|  | Sibutramine 5 mg/kg po | 10 | 47 | 7 | -298 | -86.4 | <0.001*** |
| Day 2 | Vehicle 4 ml/kg po +2 ml/kg ip | 12 | 707 | 39 |  |  |  |
|  | RDC5 0.6 mg/kg po | 10 | 686 | 29 | -21 | -3.0 | 0.597 |
|  | Bupropion 20 mg/kg ip qd | 10 | 720 | 24 | 14 | 1.9 | 0.731 |
|  | Naltrexone 1 mg/kg ip | 10 | 643 | 24 | -63 | -9.0 | 0.115 |
|  | RDC5 0.6 mg/kg po+Bupropion 20 mg/kg ip qd | 10 | 655 | 28 | -51 | -7.3 | 0.202 |
|  | RDC5 0.6 mg/kg po+Naltrexone 1 mg/kg ip | 10 | 651 | 25 | -56 | -7.9 | 0.164 |
|  | Bupropion 20 mg/kg ip qd+Naltrexone 1 mg/kg ip | 10 | 672 | 27 | -34 | -4.9 | 0.388 |
|  | Sibutramine 5 mg/kg po | 10 | 135 | 23 | -572 | -80.9 | <0.001*** |
| Day 3 | Vehicle 4 ml/kg po +2 ml/kg ip | 12 | 1058 | 49 |  |  |  |
|  | RDC5 0.6 mg/kg po | 10 | 990 | 24 | -69 | -6.5 | 0.194 |
|  | Bupropion 20 mg/kg ip qd | 10 | 1088 | 26 | 30 | 2.8 | 0.570 |
|  | Naltrexone 1 mg/kg ip | 10 | 1001 | 28 | -58 | -5.5 | 0.275 |
|  | RDC5 0.6 mg/kg po+Bupropion 20 mg/kg ip qd | 10 | 970 | 28 | -89 | -8.4 | 0.096 |
|  | RDC5 0.6 mg/kg po+Naltrexone 1 mg/kg ip | 10 | 976 | 41 | -83 | -7.8 | 0.119 |
|  | Bupropion 20 mg/kg ip qd+Naltrexone 1 mg/kg ip | 10 | 1013 | 39 | -45 | -4.3 | 0.393 |
|  | Sibutramine 5 mg/kg po | 10 | 314 | 49 | -744 | -70.3 | <0.001*** |
| Day 4 | Vehicle 4 ml/kg po +2 ml/kg ip | 12 | 1411 | 52 |  |  |  |
|  | RDC5 0.6 mg/kg po | 10 | 1305 | 35 | -106 | -7.5 | 0.116 |
|  | Bupropion 20 mg/kg ip qd | 10 | 1455 | 42 | 44 | 3.1 | 0.510 |
|  | Naltrexone 1 mg/kg ip | 10 | 1336 | 49 | -75 | -5.3 | 0.264 |
|  | RDC5 0.6 mg/kg po+Bupropion 20 mg/kg ip qd | 10 | 1295 | 41 | -116 | -8.2 | 0.086 |
|  | RDC5 0.6 mg/kg po+Naltrexone 1 mg/kg ip | 10 | 1297 | 51 | -113 | -8.0 | 0.092 |
|  | Bupropion 20 mg/kg ip qd+Naltrexone 1 mg/kg ip | 10 | 1398 | 46 | -13 | -0.9 | 0.843 |
|  | Sibutramine 5 mg/kg po | 10 | 541 | 59 | -870 | -61.6 | <0.001*** |
| Day 5 | Vehicle 4 ml/kg po +2 ml/kg ip | 12 | 1784 | 65 |  |  |  |
|  | RDC5 0.6 mg/kg po | 10 | 1634 | 55 | -150 | -8.4 | 0.086 |
|  | Bupropion 20 mg/kg ip qd | 10 | 1808 | 45 | 24 | 1.3 | 0.785 |
|  | Naltrexone 1 mg/kg ip | 10 | 1662 | 72 | -122 | -6.9 | 0.161 |
|  | RDC5 0.6 mg/kg po+Bupropion 20 mg/kg ip qd | 10 | 1634 | 56 | -150 | -8.4 | 0.087 |
|  | RDC5 0.6 mg/kg po+Naltrexone 1 mg/kg ip | 10 | 1628 | 57 | -156 | -8.7 | 0.075 |
|  | Bupropion 20 mg/kg ip qd+Naltrexone 1 mg/kg ip | 10 | 1788 | 65 | 4 | 0.2 | 0.968 |
|  | Sibutramine 5 mg/kg po | 10 | 782 | 73 | -1002 | -56.2 | <0.001*** |
| Day 6 | Vehicle 4 ml/kg po +2 ml/kg ip | 12 | 2139 | 81 |  |  |  |
|  | RDC5 0.6 mg/kg po | 10 | 1917 | 60 | -222 | -10.4 | 0.043* |
|  | Bupropion 20 mg/kg ip qd | 10 | 2182 | 61 | 43 | 2.0 | 0.688 |
|  | Naltrexone 1 mg/kg ip | 10 | 2017 | 93 | -121 | -5.7 | 0.263 |
|  | RDC5 0.6 mg/kg po+Bupropion 20 mg/kg ip qd | 10 | 1967 | 65 | -172 | -8.0 | 0.116 |
|  | RDC5 0.6 mg/kg po+Naltrexone 1 mg/kg ip | 10 | 1964 | 67 | -175 | -8.2 | 0.109 |
|  | Bupropion 20 mg/kg ip qd+Naltrexone 1 mg/kg ip | 10 | 2201 | 89 | 62 | 2.9 | 0.565 |
|  | Sibutramine 5 mg/kg po | 10 | 1017 | 90 | -1122 | -52.5 | <0.001*** |
| Day 7 | Vehicle 4 ml/kg po +2 ml/kg ip | 12 | 2498 | 96 |  |  |  |
|  | RDC5 0.6 mg/kg po | 10 | 2209 | 73 | -289 | -11.6 | 0.028* |
|  | Bupropion 20 mg/kg ip qd | 10 | 2527 | 71 | 29 | 1.2 | 0.823 |
|  | Naltrexone 1 mg/kg ip | 10 | 2400 | 114 | -98 | -3.9 | 0.450 |
|  | RDC5 0.6 mg/kg po+Bupropion 20 mg/kg ip qd | 10 | 2264 | 77 | -234 | -9.4 | 0.074 |
|  | RDC5 0.6 mg/kg po+Naltrexone 1 mg/kg ip | 10 | 2281 | 77 | -218 | -8.7 | 0.095 |
|  | Bupropion 20 mg/kg ip qd+Naltrexone 1 mg/kg ip | 10 | 2541 | 109 | 43 | 1.7 | 0.738 |
|  | Sibutramine 5 mg/kg po | 10 | 1305 | 108 | -1193 | -47.8 | <0.001*** |

Means are adjusted for differences between the treatment groups at baseline (average of Days -6 to 0). SEM are calculated from the residuals of the statistical model. Multiple comparisons against vehicle are by the multiple t test. *p<0.05,

***p<0.001.

Treatment n Mean SEM Difference % change p

from vehicle

| Day 8 | Vehicle 4 ml/kg po +2 ml/kg ip | 12 | 2832 | 110 |  |  |  |
| --- | --- | --- | --- | --- | --- | --- | --- |
|  | RDC5 0.6 mg/kg po | 10 | 2510 | 91 | -323 | -11.4 | 0.031* |
|  | Bupropion 20 mg/kg ip qd | 10 | 2899 | 92 | 67 | 2.4 | 0.650 |
|  | Naltrexone 1 mg/kg ip | 10 | 2773 | 128 | -59 | -2.1 | 0.686 |
|  | RDC5 0.6 mg/kg po+Bupropion 20 mg/kg ip qd | 10 | 2594 | 89 | -238 | -8.4 | 0.110 |
|  | RDC5 0.6 mg/kg po+Naltrexone 1 mg/kg ip | 10 | 2604 | 83 | -228 | -8.1 | 0.124 |
|  | Bupropion 20 mg/kg ip qd+Naltrexone 1 mg/kg ip | 10 | 2954 | 119 | 122 | 4.3 | 0.409 |
|  | Sibutramine 5 mg/kg po | 10 | 1575 | 118 | -1257 | -44.4 | <0.001*** |
| Day 9 | Vehicle 4 ml/kg po +2 ml/kg ip | 12 | 3213 | 123 |  |  |  |
|  | RDC5 0.6 mg/kg po | 10 | 2806 | 107 | -407 | -12.7 | 0.015* |
|  | Bupropion 20 mg/kg ip qd | 10 | 3273 | 104 | 60 | 1.9 | 0.715 |
|  | Naltrexone 1 mg/kg ip | 10 | 3144 | 137 | -69 | -2.1 | 0.675 |
|  | RDC5 0.6 mg/kg po+Bupropion 20 mg/kg ip qd | 10 | 2925 | 92 | -287 | -8.9 | 0.083 |
|  | RDC5 0.6 mg/kg po+Naltrexone 1 mg/kg ip | 10 | 2971 | 93 | -242 | -7.5 | 0.142 |
|  | Bupropion 20 mg/kg ip qd+Naltrexone 1 mg/kg ip | 10 | 3379 | 131 | 166 | 5.2 | 0.311 |
|  | Sibutramine 5 mg/kg po | 10 | 1856 | 135 | -1356 | -42.2 | <0.001*** |
| Day 10 | Vehicle 4 ml/kg po +2 ml/kg ip | 12 | 3548 | 147 |  |  |  |
|  | RDC5 0.6 mg/kg po | 10 | 3112 | 128 | -436 | -12.3 | 0.019* |
|  | Bupropion 20 mg/kg ip qd | 10 | 3636 | 106 | 88 | 2.5 | 0.633 |
|  | Naltrexone 1 mg/kg ip | 10 | 3511 | 137 | -37 | -1.0 | 0.840 |
|  | RDC5 0.6 mg/kg po+Bupropion 20 mg/kg ip qd | 10 | 3272 | 110 | -275 | -7.8 | 0.136 |
|  | RDC5 0.6 mg/kg po+Naltrexone 1 mg/kg ip | 10 | 3338 | 113 | -210 | -5.9 | 0.253 |
|  | Bupropion 20 mg/kg ip qd+Naltrexone 1 mg/kg ip | 10 | 3789 | 142 | 241 | 6.8 | 0.192 |
|  | Sibutramine 5 mg/kg po | 10 | 2149 | 150 | -1399 | -39.4 | <0.001*** |
| Day 11 | Vehicle 4 ml/kg po +2 ml/kg ip | 12 | 3884 | 168 |  |  |  |
|  | RDC5 0.6 mg/kg po | 10 | 3424 | 138 | -461 | -11.9 | 0.026* |
|  | Bupropion 20 mg/kg ip qd | 10 | 4033 | 114 | 149 | 3.8 | 0.466 |
|  | Naltrexone 1 mg/kg ip | 10 | 3920 | 156 | 36 | 0.9 | 0.858 |
|  | RDC5 0.6 mg/kg po+Bupropion 20 mg/kg ip qd | 10 | 3563 | 126 | -322 | -8.3 | 0.117 |
|  | RDC5 0.6 mg/kg po+Naltrexone 1 mg/kg ip | 10 | 3674 | 130 | -210 | -5.4 | 0.302 |
|  | Bupropion 20 mg/kg ip qd+Naltrexone 1 mg/kg ip | 10 | 4181 | 164 | 297 | 7.6 | 0.147 |
|  | Sibutramine 5 mg/kg po | 10 | 2504 | 146 | -1380 | -35.5 | <0.001*** |
| Day 12 | Vehicle 4 ml/kg po +2 ml/kg ip | 12 | 4220 | 187 |  |  |  |
|  | RDC5 0.6 mg/kg po | 10 | 3769 | 150 | -450 | -10.7 | 0.042* |
|  | Bupropion 20 mg/kg ip qd | 10 | 4440 | 109 | 221 | 5.2 | 0.315 |
|  | Naltrexone 1 mg/kg ip | 10 | 4314 | 172 | 94 | 2.2 | 0.665 |
|  | RDC5 0.6 mg/kg po+Bupropion 20 mg/kg ip qd | 10 | 3886 | 131 | -334 | -7.9 | 0.130 |
|  | RDC5 0.6 mg/kg po+Naltrexone 1 mg/kg ip | 10 | 4000 | 130 | -220 | -5.2 | 0.316 |
|  | Bupropion 20 mg/kg ip qd+Naltrexone 1 mg/kg ip | 10 | 4601 | 180 | 381 | 9.0 | 0.084 |
|  | Sibutramine 5 mg/kg po | 10 | 2845 | 157 | -1375 | -32.6 | <0.001*** |
| Day 13 | Vehicle 4 ml/kg po +2 ml/kg ip | 12 | 4593 | 200 |  |  |  |
|  | RDC5 0.6 mg/kg po | 10 | 4086 | 173 | -507 | -11.0 | 0.034* |
|  | Bupropion 20 mg/kg ip qd | 10 | 4840 | 123 | 248 | 5.4 | 0.295 |
|  | Naltrexone 1 mg/kg ip | 10 | 4687 | 186 | 94 | 2.1 | 0.689 |
|  | RDC5 0.6 mg/kg po+Bupropion 20 mg/kg ip qd | 10 | 4227 | 138 | -366 | -8.0 | 0.123 |
|  | RDC5 0.6 mg/kg po+Naltrexone 1 mg/kg ip | 10 | 4362 | 134 | -231 | -5.0 | 0.327 |
|  | Bupropion 20 mg/kg ip qd+Naltrexone 1 mg/kg ip | 10 | 5005 | 190 | 412 | 9.0 | 0.083 |
|  | Sibutramine 5 mg/kg po | 10 | 3175 | 167 | -1418 | -30.9 | <0.001*** |
| Day 14 | Vehicle 4 ml/kg po +2 ml/kg ip | 12 | 4934 | 218 |  |  |  |
|  | RDC5 0.6 mg/kg po | 10 | 4373 | 184 | -561 | -11.4 | 0.029* |
|  | Bupropion 20 mg/kg ip qd | 10 | 5203 | 134 | 269 | 5.5 | 0.291 |
|  | Naltrexone 1 mg/kg ip | 10 | 5042 | 197 | 109 | 2.2 | 0.668 |
|  | RDC5 0.6 mg/kg po+Bupropion 20 mg/kg ip qd | 10 | 4568 | 151 | -366 | -7.4 | 0.152 |
|  | RDC5 0.6 mg/kg po+Naltrexone 1 mg/kg ip | 10 | 4757 | 142 | -176 | -3.6 | 0.486 |
|  | Bupropion 20 mg/kg ip qd+Naltrexone 1 mg/kg ip | 10 | 5396 | 203 | 462 | 9.4 | 0.071 |
|  | Sibutramine 5 mg/kg po | 10 | 3464 | 183 | -1469 | -29.8 | <0.001*** |

Means are adjusted for differences between the treatment groups at baseline (average of Days -6 to 0). SEM are calculated from the residuals of the statistical model. Multiple comparisons against vehicle are by the multiple t test. *p<0.05,

***p<0.001.

Treatment n Mean SEM Difference % change p

from vehicle

| Day 15 | Vehicle 4 ml/kg po +2 ml/kg ip | 12 | 5264 | 232 |  |  |  |
| --- | --- | --- | --- | --- | --- | --- | --- |
|  | RDC5 0.6 mg/kg po | 10 | 4694 | 194 | -570 | -10.8 | 0.036* |
|  | Bupropion 20 mg/kg ip qd | 10 | 5587 | 149 | 323 | 6.1 | 0.230 |
|  | Naltrexone 1 mg/kg ip | 10 | 5425 | 208 | 161 | 3.1 | 0.547 |
|  | RDC5 0.6 mg/kg po+Bupropion 20 mg/kg ip qd | 10 | 4880 | 163 | -384 | -7.3 | 0.154 |
|  | RDC5 0.6 mg/kg po+Naltrexone 1 mg/kg ip | 10 | 5116 | 139 | -147 | -2.8 | 0.581 |
|  | Bupropion 20 mg/kg ip qd+Naltrexone 1 mg/kg ip | 10 | 5733 | 212 | 469 | 8.9 | 0.082 |
|  | Sibutramine 5 mg/kg po | 10 | 3816 | 189 | -1447 | -27.5 | <0.001*** |
| Day 16 | Vehicle 4 ml/kg po +2 ml/kg ip | 12 | 5602 | 244 |  |  |  |
|  | RDC5 0.6 mg/kg po | 10 | 5066 | 204 | -537 | -9.6 | 0.060 |
|  | Bupropion 20 mg/kg ip qd | 10 | 5965 | 167 | 363 | 6.5 | 0.202 |
|  | Naltrexone 1 mg/kg ip | 10 | 5856 | 226 | 254 | 4.5 | 0.370 |
|  | RDC5 0.6 mg/kg po+Bupropion 20 mg/kg ip qd | 10 | 5204 | 166 | -398 | -7.1 | 0.162 |
|  | RDC5 0.6 mg/kg po+Naltrexone 1 mg/kg ip | 10 | 5489 | 137 | -113 | -2.0 | 0.689 |
|  | Bupropion 20 mg/kg ip qd+Naltrexone 1 mg/kg ip | 10 | 6149 | 237 | 547 | 9.8 | 0.056 |
|  | Sibutramine 5 mg/kg po | 10 | 4196 | 186 | -1406 | -25.1 | <0.001*** |
| Day 17 | Vehicle 4 ml/kg po +2 ml/kg ip | 12 | 5948 | 255 |  |  |  |
|  | RDC5 0.6 mg/kg po | 10 | 5367 | 241 | -581 | -9.8 | 0.059 |
|  | Bupropion 20 mg/kg ip qd | 10 | 6314 | 165 | 366 | 6.2 | 0.232 |
|  | Naltrexone 1 mg/kg ip | 10 | 6184 | 250 | 236 | 4.0 | 0.440 |
|  | RDC5 0.6 mg/kg po+Bupropion 20 mg/kg ip qd | 10 | 5537 | 180 | -411 | -6.9 | 0.180 |
|  | RDC5 0.6 mg/kg po+Naltrexone 1 mg/kg ip | 10 | 5862 | 156 | -86 | -1.4 | 0.777 |
|  | Bupropion 20 mg/kg ip qd+Naltrexone 1 mg/kg ip | 10 | 6589 | 257 | 641 | 10.8 | 0.038* |
|  | Sibutramine 5 mg/kg po | 10 | 4524 | 188 | -1424 | -23.9 | <0.001*** |
| Day 18 | Vehicle 4 ml/kg po +2 ml/kg ip | 12 | 6353 | 266 |  |  |  |
|  | RDC5 0.6 mg/kg po | 10 | 5717 | 255 | -636 | -10.0 | 0.048* |
|  | Bupropion 20 mg/kg ip qd | 10 | 6758 | 165 | 405 | 6.4 | 0.207 |
|  | Naltrexone 1 mg/kg ip | 10 | 6606 | 256 | 252 | 4.0 | 0.428 |
|  | RDC5 0.6 mg/kg po+Bupropion 20 mg/kg ip qd | 10 | 5902 | 197 | -452 | -7.1 | 0.159 |
|  | RDC5 0.6 mg/kg po+Naltrexone 1 mg/kg ip | 10 | 6272 | 164 | -82 | -1.3 | 0.797 |
|  | Bupropion 20 mg/kg ip qd+Naltrexone 1 mg/kg ip | 10 | 7024 | 261 | 671 | 10.6 | 0.038* |
|  | Sibutramine 5 mg/kg po | 10 | 4880 | 205 | -1474 | -23.2 | <0.001*** |
| Day 19 | Vehicle 4 ml/kg po +2 ml/kg ip | 12 | 6727 | 269 |  |  |  |
|  | RDC5 0.6 mg/kg po | 10 | 6089 | 253 | -639 | -9.5 | 0.055 |
|  | Bupropion 20 mg/kg ip qd | 10 | 7203 | 171 | 475 | 7.1 | 0.152 |
|  | Naltrexone 1 mg/kg ip | 10 | 7023 | 273 | 296 | 4.4 | 0.369 |
|  | RDC5 0.6 mg/kg po+Bupropion 20 mg/kg ip qd | 10 | 6253 | 215 | -474 | -7.0 | 0.152 |
|  | RDC5 0.6 mg/kg po+Naltrexone 1 mg/kg ip | 10 | 6680 | 172 | -47 | -0.7 | 0.886 |
|  | Bupropion 20 mg/kg ip qd+Naltrexone 1 mg/kg ip | 10 | 7422 | 266 | 695 | 10.3 | 0.037* |
|  | Sibutramine 5 mg/kg po | 10 | 5234 | 216 | -1493 | -22.2 | <0.001*** |
| Day 20 | Vehicle 4 ml/kg po +2 ml/kg ip | 12 | 7072 | 271 |  |  |  |
|  | RDC5 0.6 mg/kg po | 10 | 6453 | 258 | -619 | -8.7 | 0.070 |
|  | Bupropion 20 mg/kg ip qd | 10 | 7581 | 175 | 509 | 7.2 | 0.136 |
|  | Naltrexone 1 mg/kg ip | 10 | 7399 | 284 | 327 | 4.6 | 0.335 |
|  | RDC5 0.6 mg/kg po+Bupropion 20 mg/kg ip qd | 10 | 6598 | 223 | -473 | -6.7 | 0.165 |
|  | RDC5 0.6 mg/kg po+Naltrexone 1 mg/kg ip | 10 | 7037 | 187 | -35 | -0.5 | 0.919 |
|  | Bupropion 20 mg/kg ip qd+Naltrexone 1 mg/kg ip | 10 | 7789 | 273 | 717 | 10.1 | 0.037* |
|  | Sibutramine 5 mg/kg po | 10 | 5582 | 225 | -1489 | -21.1 | <0.001*** |
| Day 21 | Vehicle 4 ml/kg po +2 ml/kg ip | 12 | 7414 | 276 |  |  |  |
|  | RDC5 0.6 mg/kg po | 10 | 6825 | 259 | -589 | -7.9 | 0.097 |
|  | Bupropion 20 mg/kg ip qd | 10 | 7932 | 173 | 518 | 7.0 | 0.145 |
|  | Naltrexone 1 mg/kg ip | 10 | 7774 | 298 | 360 | 4.9 | 0.308 |
|  | RDC5 0.6 mg/kg po+Bupropion 20 mg/kg ip qd | 10 | 6954 | 242 | -460 | -6.2 | 0.194 |
|  | RDC5 0.6 mg/kg po+Naltrexone 1 mg/kg ip | 10 | 7412 | 200 | -2 | -0.0 | 0.996 |
|  | Bupropion 20 mg/kg ip qd+Naltrexone 1 mg/kg ip | 10 | 8173 | 289 | 759 | 10.2 | 0.034* |
|  | Sibutramine 5 mg/kg po | 10 | 5905 | 236 | -1509 | -20.4 | <0.001*** |

Means are adjusted for differences between the treatment groups at baseline (average of Days -6 to 0). SEM are calculated from the residuals of the statistical model. Multiple comparisons against vehicle are by the multiple t test. *p<0.05,

***p<0.001.

Treatment n Mean SEM Difference % change p

from vehicle

| Day 22 | Vehicle 4 ml/kg po +2 ml/kg ip | 12 | 7761 | 287 |  |  |  |
| --- | --- | --- | --- | --- | --- | --- | --- |
|  | RDC5 0.6 mg/kg po | 10 | 7196 | 258 | -565 | -7.3 | 0.125 |
|  | Bupropion 20 mg/kg ip qd | 10 | 8330 | 176 | 569 | 7.3 | 0.123 |
|  | Naltrexone 1 mg/kg ip | 10 | 8176 | 314 | 415 | 5.3 | 0.257 |
|  | RDC5 0.6 mg/kg po+Bupropion 20 mg/kg ip qd | 10 | 7353 | 254 | -408 | -5.3 | 0.266 |
|  | RDC5 0.6 mg/kg po+Naltrexone 1 mg/kg ip | 10 | 7826 | 211 | 64 | 0.8 | 0.860 |
|  | Bupropion 20 mg/kg ip qd+Naltrexone 1 mg/kg ip | 10 | 8553 | 300 | 792 | 10.2 | 0.033* |
|  | Sibutramine 5 mg/kg po | 10 | 6224 | 244 | -1537 | -19.8 | <0.001*** |
| Day 23 | Vehicle 4 ml/kg po +2 ml/kg ip | 12 | 8134 | 292 |  |  |  |
|  | RDC5 0.6 mg/kg po | 10 | 7575 | 263 | -559 | -6.9 | 0.140 |
|  | Bupropion 20 mg/kg ip qd | 10 | 8748 | 170 | 614 | 7.5 | 0.106 |
|  | Naltrexone 1 mg/kg ip | 10 | 8610 | 327 | 476 | 5.8 | 0.208 |
|  | RDC5 0.6 mg/kg po+Bupropion 20 mg/kg ip qd | 10 | 7735 | 282 | -398 | -4.9 | 0.292 |
|  | RDC5 0.6 mg/kg po+Naltrexone 1 mg/kg ip | 10 | 8217 | 211 | 83 | 1.0 | 0.825 |
|  | Bupropion 20 mg/kg ip qd+Naltrexone 1 mg/kg ip | 10 | 8939 | 304 | 806 | 9.9 | 0.035* |
|  | Sibutramine 5 mg/kg po | 10 | 6577 | 254 | -1557 | -19.1 | <0.001*** |
| Day 24 | Vehicle 4 ml/kg po +2 ml/kg ip | 12 | 8486 | 296 |  |  |  |
|  | RDC5 0.6 mg/kg po | 10 | 7927 | 275 | -559 | -6.6 | 0.151 |
|  | Bupropion 20 mg/kg ip qd | 10 | 9155 | 184 | 670 | 7.9 | 0.087 |
|  | Naltrexone 1 mg/kg ip | 10 | 8965 | 333 | 479 | 5.6 | 0.217 |
|  | RDC5 0.6 mg/kg po+Bupropion 20 mg/kg ip qd | 10 | 8116 | 306 | -369 | -4.4 | 0.341 |
|  | RDC5 0.6 mg/kg po+Naltrexone 1 mg/kg ip | 10 | 8568 | 222 | 83 | 1.0 | 0.831 |
|  | Bupropion 20 mg/kg ip qd+Naltrexone 1 mg/kg ip | 10 | 9335 | 295 | 850 | 10.0 | 0.030* |
|  | Sibutramine 5 mg/kg po | 10 | 6926 | 261 | -1560 | -18.4 | <0.001*** |
| Day 25 | Vehicle 4 ml/kg po +2 ml/kg ip | 11 | 8911 | 303 |  |  |  |
|  | RDC5 0.6 mg/kg po | 10 | 8275 | 284 | -636 | -7.1 | 0.121 |
|  | Bupropion 20 mg/kg ip qd | 10 | 9531 | 197 | 620 | 7.0 | 0.131 |
|  | Naltrexone 1 mg/kg ip | 10 | 9329 | 344 | 418 | 4.7 | 0.306 |
|  | RDC5 0.6 mg/kg po+Bupropion 20 mg/kg ip qd | 10 | 8475 | 330 | -436 | -4.9 | 0.288 |
|  | RDC5 0.6 mg/kg po+Naltrexone 1 mg/kg ip | 10 | 8881 | 228 | -31 | -0.3 | 0.940 |
|  | Bupropion 20 mg/kg ip qd+Naltrexone 1 mg/kg ip | 10 | 9697 | 303 | 786 | 8.8 | 0.057 |
|  | Sibutramine 5 mg/kg po | 10 | 7253 | 277 | -1658 | -18.6 | <0.001*** |
| Day 26 | Vehicle 4 ml/kg po +2 ml/kg ip | 11 | 9266 | 317 |  |  |  |
|  | RDC5 0.6 mg/kg po | 10 | 8645 | 287 | -622 | -6.7 | 0.146 |
|  | Bupropion 20 mg/kg ip bid | 10 | 9920 | 214 | 654 | 7.1 | 0.127 |
|  | Naltrexone 1 mg/kg ip | 10 | 9737 | 346 | 470 | 5.1 | 0.269 |
|  | RDC5 0.6 mg/kg po+Bupropion 20 mg/kg ip bid | 10 | 8864 | 353 | -402 | -4.3 | 0.346 |
|  | RDC5 0.6 mg/kg po+Naltrexone 1 mg/kg ip | 10 | 9259 | 229 | -7 | -0.1 | 0.986 |
|  | Bupropion 20 mg/kg ip bid+Naltrexone 1 mg/kg ip | 10 | 10069 | 321 | 803 | 8.7 | 0.062 |
|  | Sibutramine 5 mg/kg po | 10 | 7589 | 293 | -1678 | -18.1 | <0.001*** |
| Day 27 | Vehicle 4 ml/kg po +2 ml/kg ip | 11 | 9620 | 328 |  |  |  |
|  | RDC5 0.6 mg/kg po | 10 | 8949 | 291 | -671 | -7.0 | 0.128 |
|  | Bupropion 20 mg/kg ip bid | 10 | 10289 | 233 | 669 | 7.0 | 0.130 |
|  | Naltrexone 1 mg/kg ip | 10 | 10101 | 354 | 481 | 5.0 | 0.273 |
|  | RDC5 0.6 mg/kg po+Bupropion 20 mg/kg ip bid | 10 | 9208 | 364 | -412 | -4.3 | 0.349 |
|  | RDC5 0.6 mg/kg po+Naltrexone 1 mg/kg ip | 10 | 9631 | 235 | 10 | 0.1 | 0.981 |
|  | Bupropion 20 mg/kg ip bid+Naltrexone 1 mg/kg ip | 10 | 10384 | 329 | 764 | 7.9 | 0.084 |
|  | Sibutramine 5 mg/kg po | 10 | 7927 | 304 | -1693 | -17.6 | <0.001*** |
| Day 28 | Vehicle 4 ml/kg po +2 ml/kg ip | 11 | 9940 | 334 |  |  |  |
|  | RDC5 0.6 mg/kg po | 10 | 9297 | 304 | -643 | -6.5 | 0.153 |
|  | Bupropion 20 mg/kg ip bid | 10 | 10637 | 236 | 697 | 7.0 | 0.122 |
|  | Naltrexone 1 mg/kg ip | 10 | 10473 | 354 | 533 | 5.4 | 0.235 |
|  | RDC5 0.6 mg/kg po+Bupropion 20 mg/kg ip bid | 10 | 9556 | 379 | -384 | -3.9 | 0.392 |
|  | RDC5 0.6 mg/kg po+Naltrexone 1 mg/kg ip | 10 | 9996 | 248 | 56 | 0.6 | 0.901 |
|  | Bupropion 20 mg/kg ip bid+Naltrexone 1 mg/kg ip | 10 | 10740 | 324 | 800 | 8.0 | 0.076 |
|  | Sibutramine 5 mg/kg po | 10 | 8247 | 310 | -1693 | -17.0 | <0.001*** |

Means are adjusted for differences between the treatment groups at baseline (average of Days -6 to 0). SEM are calculated from the residuals of the statistical model. Multiple comparisons against vehicle are by the multiple t test. *p<0.05,

***p<0.001.

Treatment n Mean SEM Difference % change p

from vehicle

| Day 29 | Vehicle 4 ml/kg po +2 ml/kg ip | 11 | 10288 | 340 |  |  |  |
| --- | --- | --- | --- | --- | --- | --- | --- |
|  | RDC5 0.6 mg/kg po | 10 | 9652 | 319 | -636 | -6.2 | 0.165 |
|  | Bupropion 20 mg/kg ip bid | 10 | 10996 | 250 | 708 | 6.9 | 0.122 |
|  | Naltrexone 1 mg/kg ip | 10 | 10793 | 348 | 505 | 4.9 | 0.268 |
|  | RDC5 0.6 mg/kg po+Bupropion 20 mg/kg ip bid | 10 | 9934 | 381 | -353 | -3.4 | 0.439 |
|  | RDC5 0.6 mg/kg po+Naltrexone 1 mg/kg ip | 10 | 10305 | 263 | 17 | 0.2 | 0.970 |
|  | Bupropion 20 mg/kg ip bid+Naltrexone 1 mg/kg ip | 10 | 11111 | 322 | 823 | 8.0 | 0.073 |
|  | Sibutramine 5 mg/kg po | 10 | 8535 | 319 | -1753 | -17.0 | <0.001*** |
| Day 30 | Vehicle 4 ml/kg po +2 ml/kg ip | 11 | 10632 | 354 |  |  |  |
|  | RDC5 0.6 mg/kg po | 10 | 10027 | 335 | -605 | -5.7 | 0.205 |
|  | Bupropion 20 mg/kg ip bid | 10 | 11366 | 259 | 734 | 6.9 | 0.125 |
|  | Naltrexone 1 mg/kg ip | 10 | 11160 | 349 | 528 | 5.0 | 0.268 |
|  | RDC5 0.6 mg/kg po+Bupropion 20 mg/kg ip bid | 10 | 10317 | 381 | -315 | -3.0 | 0.509 |
|  | RDC5 0.6 mg/kg po+Naltrexone 1 mg/kg ip | 10 | 10712 | 279 | 81 | 0.8 | 0.865 |
|  | Bupropion 20 mg/kg ip bid+Naltrexone 1 mg/kg ip | 10 | 11485 | 370 | 853 | 8.0 | 0.075 |
|  | Sibutramine 5 mg/kg po | 10 | 8871 | 328 | -1761 | -16.6 | <0.001*** |
| Day 31 | Vehicle 4 ml/kg po +2 ml/kg ip | 11 | 10971 | 368 |  |  |  |
|  | RDC5 0.6 mg/kg po | 10 | 10333 | 351 | -638 | -5.8 | 0.200 |
|  | Bupropion 20 mg/kg ip bid | 10 | 11728 | 277 | 756 | 6.9 | 0.130 |
|  | Naltrexone 1 mg/kg ip | 10 | 11520 | 368 | 549 | 5.0 | 0.269 |
|  | RDC5 0.6 mg/kg po+Bupropion 20 mg/kg ip bid | 10 | 10676 | 398 | -295 | -2.7 | 0.553 |
|  | RDC5 0.6 mg/kg po+Naltrexone 1 mg/kg ip | 10 | 11104 | 284 | 133 | 1.2 | 0.788 |
|  | Bupropion 20 mg/kg ip bid+Naltrexone 1 mg/kg ip | 10 | 11859 | 398 | 888 | 8.1 | 0.076 |
|  | Sibutramine 5 mg/kg po | 10 | 9208 | 324 | -1763 | -16.1 | <0.001*** |
| Day 32 | Vehicle 4 ml/kg po +2 ml/kg ip | 11 | 11323 | 382 |  |  |  |
|  | RDC5 0.6 mg/kg po | 10 | 10602 | 379 | -721 | -6.4 | 0.163 |
|  | Bupropion 20 mg/kg ip bid | 10 | 12058 | 297 | 735 | 6.5 | 0.155 |
|  | Naltrexone 1 mg/kg ip | 10 | 11877 | 377 | 554 | 4.9 | 0.282 |
|  | RDC5 0.6 mg/kg po+Bupropion 20 mg/kg ip bid | 10 | 10992 | 404 | -331 | -2.9 | 0.521 |
|  | RDC5 0.6 mg/kg po+Naltrexone 1 mg/kg ip | 10 | 11455 | 286 | 132 | 1.2 | 0.797 |
|  | Bupropion 20 mg/kg ip bid+Naltrexone 1 mg/kg ip | 10 | 12167 | 418 | 844 | 7.5 | 0.103 |
|  | Sibutramine 5 mg/kg po | 10 | 9540 | 326 | -1783 | -15.7 | <0.001*** |
| Day 33 | Vehicle 4 ml/kg po +2 ml/kg ip | 11 | 11656 | 395 |  |  |  |
|  | RDC5 0.6 mg/kg po | 10 | 10935 | 398 | -721 | -6.2 | 0.177 |
|  | Bupropion 20 mg/kg ip bid | 10 | 12405 | 309 | 748 | 6.4 | 0.161 |
|  | Naltrexone 1 mg/kg ip | 10 | 12207 | 380 | 551 | 4.7 | 0.300 |
|  | RDC5 0.6 mg/kg po+Bupropion 20 mg/kg ip bid | 10 | 11325 | 415 | -331 | -2.8 | 0.534 |
|  | RDC5 0.6 mg/kg po+Naltrexone 1 mg/kg ip | 10 | 11812 | 294 | 156 | 1.3 | 0.769 |
|  | Bupropion 20 mg/kg ip bid+Naltrexone 1 mg/kg ip | 10 | 12492 | 437 | 835 | 7.2 | 0.118 |
|  | Sibutramine 5 mg/kg po | 10 | 9883 | 334 | -1773 | -15.2 | 0.001** |
| Day 34 | Vehicle 4 ml/kg po +2 ml/kg ip | 11 | 11983 | 404 |  |  |  |
|  | RDC5 0.6 mg/kg po | 10 | 11292 | 404 | -691 | -5.8 | 0.208 |
|  | Bupropion 20 mg/kg ip bid | 10 | 12757 | 315 | 774 | 6.5 | 0.159 |
|  | Naltrexone 1 mg/kg ip | 10 | 12493 | 379 | 510 | 4.3 | 0.351 |
|  | RDC5 0.6 mg/kg po+Bupropion 20 mg/kg ip bid | 10 | 11709 | 427 | -275 | -2.3 | 0.616 |
|  | RDC5 0.6 mg/kg po+Naltrexone 1 mg/kg ip | 10 | 12165 | 319 | 181 | 1.5 | 0.740 |
|  | Bupropion 20 mg/kg ip bid+Naltrexone 1 mg/kg ip | 10 | 12838 | 464 | 855 | 7.1 | 0.120 |
|  | Sibutramine 5 mg/kg po | 10 | 10208 | 339 | -1776 | -14.8 | 0.002** |
| Day 35 | Vehicle 4 ml/kg po +2 ml/kg ip | 11 | 12326 | 411 |  |  |  |
|  | RDC5 0.6 mg/kg po | 10 | 11680 | 406 | -647 | -5.2 | 0.250 |
|  | Bupropion 20 mg/kg ip bid | 10 | 13101 | 316 | 775 | 6.3 | 0.169 |
|  | Naltrexone 1 mg/kg ip | 10 | 12841 | 395 | 514 | 4.2 | 0.359 |
|  | RDC5 0.6 mg/kg po+Bupropion 20 mg/kg ip bid | 10 | 12071 | 442 | -255 | -2.1 | 0.649 |
|  | RDC5 0.6 mg/kg po+Naltrexone 1 mg/kg ip | 10 | 12550 | 320 | 224 | 1.8 | 0.689 |
|  | Bupropion 20 mg/kg ip bid+Naltrexone 1 mg/kg ip | 10 | 13140 | 489 | 814 | 6.6 | 0.148 |
|  | Sibutramine 5 mg/kg po | 10 | 10544 | 340 | -1782 | -14.5 | 0.002** |

Means are adjusted for differences between the treatment groups at baseline (average of Days -6 to 0). SEM are calculated from the residuals of the statistical model. Multiple comparisons against vehicle are by the multiple t test. **p<0.01,

***p<0.001.

Treatment n Mean SEM Difference % change p

from vehicle

| Day 36 | Vehicle 4 ml/kg po +2 ml/kg ip | 11 | 12502 | 415 |  |  |  |
| --- | --- | --- | --- | --- | --- | --- | --- |
|  | RDC5 0.6 mg/kg po | 10 | 11852 | 416 | -650 | -5.2 | 0.250 |
|  | Bupropion 20 mg/kg ip bid | 10 | 13273 | 311 | 771 | 6.2 | 0.173 |
|  | Naltrexone 1 mg/kg ip | 10 | 12992 | 403 | 491 | 3.9 | 0.384 |
|  | RDC5 0.6 mg/kg po+Bupropion 20 mg/kg ip bid | 10 | 12239 | 437 | -263 | -2.1 | 0.641 |
|  | RDC5 0.6 mg/kg po+Naltrexone 1 mg/kg ip | 10 | 12743 | 309 | 241 | 1.9 | 0.668 |
|  | Bupropion 20 mg/kg ip bid+Naltrexone 1 mg/kg ip | 10 | 13285 | 498 | 783 | 6.3 | 0.166 |
|  | Sibutramine 5 mg/kg po | 10 | 10693 | 343 | -1808 | -14.5 | 0.002** |
| Day 37 | Vehicle 4 ml/kg po +2 ml/kg ip | 11 | 12801 | 429 |  |  |  |
|  | RDC5 0.6 mg/kg po | 10 | 12191 | 421 | -610 | -4.8 | 0.288 |
|  | Bupropion 20 mg/kg ip bid | 10 | 13614 | 323 | 813 | 6.3 | 0.159 |
|  | Naltrexone 1 mg/kg ip | 10 | 13314 | 414 | 513 | 4.0 | 0.371 |
|  | RDC5 0.6 mg/kg po+Bupropion 20 mg/kg ip bid | 10 | 12553 | 437 | -248 | -1.9 | 0.666 |
|  | RDC5 0.6 mg/kg po+Naltrexone 1 mg/kg ip | 10 | 13105 | 317 | 304 | 2.4 | 0.596 |
|  | Bupropion 20 mg/kg ip bid+Naltrexone 1 mg/kg ip | 10 | 13623 | 499 | 822 | 6.4 | 0.154 |
|  | Sibutramine 5 mg/kg po | 10 | 10990 | 349 | -1811 | -14.1 | 0.002** |
| Day 38 | Vehicle 4 ml/kg po +2 ml/kg ip | 11 | 13122 | 443 |  |  |  |
|  | RDC5 0.6 mg/kg po | 10 | 12574 | 433 | -548 | -4.2 | 0.346 |
|  | Bupropion 20 mg/kg ip bid | 10 | 14006 | 322 | 885 | 6.7 | 0.130 |
|  | Naltrexone 1 mg/kg ip | 10 | 13670 | 402 | 548 | 4.2 | 0.345 |
|  | RDC5 0.6 mg/kg po+Bupropion 20 mg/kg ip bid | 10 | 12920 | 439 | -202 | -1.5 | 0.729 |
|  | RDC5 0.6 mg/kg po+Naltrexone 1 mg/kg ip | 10 | 13494 | 329 | 373 | 2.8 | 0.520 |
|  | Bupropion 20 mg/kg ip bid+Naltrexone 1 mg/kg ip | 10 | 14001 | 501 | 879 | 6.7 | 0.132 |
|  | Sibutramine 5 mg/kg po | 10 | 11318 | 359 | -1803 | -13.7 | 0.003** |
| Day 39 | Vehicle 4 ml/kg po +2 ml/kg ip | 11 | 13457 | 447 |  |  |  |
|  | RDC5 0.6 mg/kg po | 10 | 12948 | 428 | -509 | -3.8 | 0.388 |
|  | Bupropion 20 mg/kg ip bid | 10 | 14372 | 332 | 915 | 6.8 | 0.123 |
|  | Naltrexone 1 mg/kg ip | 10 | 14009 | 400 | 552 | 4.1 | 0.349 |
|  | RDC5 0.6 mg/kg po+Bupropion 20 mg/kg ip bid | 10 | 13260 | 465 | -198 | -1.5 | 0.738 |
|  | RDC5 0.6 mg/kg po+Naltrexone 1 mg/kg ip | 10 | 13909 | 343 | 451 | 3.4 | 0.443 |
|  | Bupropion 20 mg/kg ip bid+Naltrexone 1 mg/kg ip | 10 | 14334 | 494 | 876 | 6.5 | 0.139 |
|  | Sibutramine 5 mg/kg po | 10 | 11641 | 375 | -1817 | -13.5 | 0.003** |
| Day 40 | Vehicle 4 ml/kg po +2 ml/kg ip | 11 | 13810 | 451 |  |  |  |
|  | RDC5 0.6 mg/kg po | 10 | 13364 | 417 | -446 | -3.2 | 0.455 |
|  | Bupropion 20 mg/kg ip bid | 10 | 14756 | 344 | 946 | 6.8 | 0.115 |
|  | Naltrexone 1 mg/kg ip | 10 | 14406 | 404 | 596 | 4.3 | 0.318 |
|  | RDC5 0.6 mg/kg po+Bupropion 20 mg/kg ip bid | 10 | 13580 | 486 | -230 | -1.7 | 0.700 |
|  | RDC5 0.6 mg/kg po+Naltrexone 1 mg/kg ip | 10 | 14320 | 344 | 510 | 3.7 | 0.393 |
|  | Bupropion 20 mg/kg ip bid+Naltrexone 1 mg/kg ip | 10 | 14679 | 495 | 869 | 6.3 | 0.147 |
|  | Sibutramine 5 mg/kg po | 10 | 12014 | 383 | -1796 | -13.0 | 0.003** |
| Day 41 | Vehicle 4 ml/kg po +2 ml/kg ip | 11 | 14166 | 462 |  |  |  |
|  | RDC5 0.6 mg/kg po | 10 | 13768 | 410 | -397 | -2.8 | 0.509 |
|  | Bupropion 20 mg/kg ip bid | 10 | 15120 | 356 | 955 | 6.7 | 0.116 |
|  | Naltrexone 1 mg/kg ip | 10 | 14780 | 398 | 614 | 4.3 | 0.308 |
|  | RDC5 0.6 mg/kg po+Bupropion 20 mg/kg ip bid | 10 | 13880 | 492 | -285 | -2.0 | 0.636 |
|  | RDC5 0.6 mg/kg po+Naltrexone 1 mg/kg ip | 10 | 14700 | 349 | 534 | 3.8 | 0.376 |
|  | Bupropion 20 mg/kg ip bid+Naltrexone 1 mg/kg ip | 10 | 15027 | 499 | 861 | 6.1 | 0.154 |
|  | Sibutramine 5 mg/kg po | 10 | 12335 | 390 | -1831 | -12.9 | 0.003** |
| Day 42 | Vehicle 4 ml/kg po +2 ml/kg ip | 11 | 14517 | 469 |  |  |  |
|  | RDC5 0.6 mg/kg po | 10 | 14155 | 402 | -362 | -2.5 | 0.549 |
|  | Bupropion 20 mg/kg ip bid | 10 | 15490 | 367 | 973 | 6.7 | 0.110 |
|  | Naltrexone 1 mg/kg ip | 10 | 15159 | 400 | 642 | 4.4 | 0.290 |
|  | RDC5 0.6 mg/kg po+Bupropion 20 mg/kg ip bid | 10 | 14240 | 489 | -277 | -1.9 | 0.647 |
|  | RDC5 0.6 mg/kg po+Naltrexone 1 mg/kg ip | 10 | 15106 | 348 | 589 | 4.1 | 0.331 |
|  | Bupropion 20 mg/kg ip bid+Naltrexone 1 mg/kg ip | 10 | 15408 | 498 | 891 | 6.1 | 0.143 |
|  | Sibutramine 5 mg/kg po | 10 | 12652 | 399 | -1865 | -12.8 | 0.003** |

Means are adjusted for differences between the treatment groups at baseline (average of Days -6 to 0). SEM are calculated from the residuals of the statistical model. Multiple comparisons against vehicle are by the multiple t test. **p<0.01.

| Time | Comparison | Difference | p |
| --- | --- | --- | --- |
| Day 1 | RDC5 (0.6 mg/kg po)+Bupropion (20 mg/kg ip qd) vs RDC5 | -7 | 0.800 |
|  | RDC5 (0.6 mg/kg po)+Naltrexone (1 mg/kg ip) vs RDC5 | -22 | 0.414 |
|  | RDC5 (0.6 mg/kg po)+Bupropion (20 mg/kg ip qd) vs Bupropion | -23 | 0.400 |
|  | Bupropion (20 mg/kg ip qd)+Naltrexone (1 mg/kg ip) vs Bupropion | -11 | 0.683 |
|  | RDC5 (0.6 mg/kg po)+Naltrexone (1 mg/kg ip) vs Naltrexone | 0 | 0.986 |
|  | Bupropion (20 mg/kg ip qd)+Naltrexone (1 mg/kg ip) vs Naltrexone | 27 | 0.307 |
| Day 2 | RDC5 (0.6 mg/kg po)+Bupropion (20 mg/kg ip qd) vs RDC5 | -30 | 0.470 |
|  | RDC5 (0.6 mg/kg po)+Naltrexone (1 mg/kg ip) vs RDC5 | -35 | 0.405 |
|  | RDC5 (0.6 mg/kg po)+Bupropion (20 mg/kg ip qd) vs Bupropion | -65 | 0.125 |
|  | Bupropion (20 mg/kg ip qd)+Naltrexone (1 mg/kg ip) vs Bupropion | -48 | 0.250 |
|  | RDC5 (0.6 mg/kg po)+Naltrexone (1 mg/kg ip) vs Naltrexone | 8 | 0.856 |
|  | Bupropion (20 mg/kg ip qd)+Naltrexone (1 mg/kg ip) vs Naltrexone | 29 | 0.488 |
| Day 3 | RDC5 (0.6 mg/kg po)+Bupropion (20 mg/kg ip qd) vs RDC5 | -20 | 0.718 |
|  | RDC5 (0.6 mg/kg po)+Naltrexone (1 mg/kg ip) vs RDC5 | -14 | 0.798 |
|  | RDC5 (0.6 mg/kg po)+Bupropion (20 mg/kg ip qd) vs Bupropion | -119 | 0.035* |
|  | Bupropion (20 mg/kg ip qd)+Naltrexone (1 mg/kg ip) vs Bupropion | -75 | 0.175 |
|  | RDC5 (0.6 mg/kg po)+Naltrexone (1 mg/kg ip) vs Naltrexone | -25 | 0.648 |
|  | Bupropion (20 mg/kg ip qd)+Naltrexone (1 mg/kg ip) vs Naltrexone | 13 | 0.818 |
| Day 4 | RDC5 (0.6 mg/kg po)+Bupropion (20 mg/kg ip qd) vs RDC5 | -10 | 0.888 |
|  | RDC5 (0.6 mg/kg po)+Naltrexone (1 mg/kg ip) vs RDC5 | -8 | 0.912 |
|  | RDC5 (0.6 mg/kg po)+Bupropion (20 mg/kg ip qd) vs Bupropion | -160 | 0.025* |
|  | Bupropion (20 mg/kg ip qd)+Naltrexone (1 mg/kg ip) vs Bupropion | -57 | 0.412 |
|  | RDC5 (0.6 mg/kg po)+Naltrexone (1 mg/kg ip) vs Naltrexone | -39 | 0.579 |
|  | Bupropion (20 mg/kg ip qd)+Naltrexone (1 mg/kg ip) vs Naltrexone | 62 | 0.377 |
| Day 5 | RDC5 (0.6 mg/kg po)+Bupropion (20 mg/kg ip qd) vs RDC5 | 0 | 0.998 |
|  | RDC5 (0.6 mg/kg po)+Naltrexone (1 mg/kg ip) vs RDC5 | -6 | 0.951 |
|  | RDC5 (0.6 mg/kg po)+Bupropion (20 mg/kg ip qd) vs Bupropion | -174 | 0.060 |
|  | Bupropion (20 mg/kg ip qd)+Naltrexone (1 mg/kg ip) vs Bupropion | -20 | 0.824 |
|  | RDC5 (0.6 mg/kg po)+Naltrexone (1 mg/kg ip) vs Naltrexone | -34 | 0.711 |
|  | Bupropion (20 mg/kg ip qd)+Naltrexone (1 mg/kg ip) vs Naltrexone | 126 | 0.167 |
| Day 6 | RDC5 (0.6 mg/kg po)+Bupropion (20 mg/kg ip qd) vs RDC5 | 50 | 0.656 |
|  | RDC5 (0.6 mg/kg po)+Naltrexone (1 mg/kg ip) vs RDC5 | 47 | 0.675 |
|  | RDC5 (0.6 mg/kg po)+Bupropion (20 mg/kg ip qd) vs Bupropion | -215 | 0.062 |
|  | Bupropion (20 mg/kg ip qd)+Naltrexone (1 mg/kg ip) vs Bupropion | 19 | 0.869 |
|  | RDC5 (0.6 mg/kg po)+Naltrexone (1 mg/kg ip) vs Naltrexone | -53 | 0.637 |
|  | Bupropion (20 mg/kg ip qd)+Naltrexone (1 mg/kg ip) vs Naltrexone | 183 | 0.107 |
| Day 7 | RDC5 (0.6 mg/kg po)+Bupropion (20 mg/kg ip qd) vs RDC5 | 55 | 0.684 |
|  | RDC5 (0.6 mg/kg po)+Naltrexone (1 mg/kg ip) vs RDC5 | 71 | 0.597 |
|  | RDC5 (0.6 mg/kg po)+Bupropion (20 mg/kg ip qd) vs Bupropion | -263 | 0.056 |
|  | Bupropion (20 mg/kg ip qd)+Naltrexone (1 mg/kg ip) vs Bupropion | 14 | 0.917 |
|  | RDC5 (0.6 mg/kg po)+Naltrexone (1 mg/kg ip) vs Naltrexone | -120 | 0.376 |
|  | Bupropion (20 mg/kg ip qd)+Naltrexone (1 mg/kg ip) vs Naltrexone | 141 | 0.298 |

Multiple comparisons are by the multiple t test. *p<0.05.

| Time | Comparison | Difference | p |
| --- | --- | --- | --- |
| Day 8 | RDC5 (0.6 mg/kg po)+Bupropion (20 mg/kg ip qd) vs RDC5 | 85 | 0.581 |
|  | RDC5 (0.6 mg/kg po)+Naltrexone (1 mg/kg ip) vs RDC5 | 95 | 0.539 |
|  | RDC5 (0.6 mg/kg po)+Bupropion (20 mg/kg ip qd) vs Bupropion | -305 | 0.052 |
|  | Bupropion (20 mg/kg ip qd)+Naltrexone (1 mg/kg ip) vs Bupropion | 55 | 0.722 |
|  | RDC5 (0.6 mg/kg po)+Naltrexone (1 mg/kg ip) vs Naltrexone | -169 | 0.274 |
|  | Bupropion (20 mg/kg ip qd)+Naltrexone (1 mg/kg ip) vs Naltrexone | 181 | 0.241 |
| Day 9 | RDC5 (0.6 mg/kg po)+Bupropion (20 mg/kg ip qd) vs RDC5 | 119 | 0.486 |
|  | RDC5 (0.6 mg/kg po)+Naltrexone (1 mg/kg ip) vs RDC5 | 165 | 0.336 |
|  | RDC5 (0.6 mg/kg po)+Bupropion (20 mg/kg ip qd) vs Bupropion | -347 | 0.047* |
|  | Bupropion (20 mg/kg ip qd)+Naltrexone (1 mg/kg ip) vs Bupropion | 106 | 0.534 |
|  | RDC5 (0.6 mg/kg po)+Naltrexone (1 mg/kg ip) vs Naltrexone | -173 | 0.312 |
|  | Bupropion (20 mg/kg ip qd)+Naltrexone (1 mg/kg ip) vs Naltrexone | 235 | 0.172 |
| Day 10 | RDC5 (0.6 mg/kg po)+Bupropion (20 mg/kg ip qd) vs RDC5 | 161 | 0.402 |
|  | RDC5 (0.6 mg/kg po)+Naltrexone (1 mg/kg ip) vs RDC5 | 226 | 0.239 |
|  | RDC5 (0.6 mg/kg po)+Bupropion (20 mg/kg ip qd) vs Bupropion | -363 | 0.063 |
|  | Bupropion (20 mg/kg ip qd)+Naltrexone (1 mg/kg ip) vs Bupropion | 153 | 0.426 |
|  | RDC5 (0.6 mg/kg po)+Naltrexone (1 mg/kg ip) vs Naltrexone | -173 | 0.367 |
|  | Bupropion (20 mg/kg ip qd)+Naltrexone (1 mg/kg ip) vs Naltrexone | 278 | 0.150 |
| Day 11 | RDC5 (0.6 mg/kg po)+Bupropion (20 mg/kg ip qd) vs RDC5 | 139 | 0.513 |
|  | RDC5 (0.6 mg/kg po)+Naltrexone (1 mg/kg ip) vs RDC5 | 250 | 0.240 |
|  | RDC5 (0.6 mg/kg po)+Bupropion (20 mg/kg ip qd) vs Bupropion | -470 | 0.030* |
|  | Bupropion (20 mg/kg ip qd)+Naltrexone (1 mg/kg ip) vs Bupropion | 148 | 0.486 |
|  | RDC5 (0.6 mg/kg po)+Naltrexone (1 mg/kg ip) vs Naltrexone | -247 | 0.247 |
|  | Bupropion (20 mg/kg ip qd)+Naltrexone (1 mg/kg ip) vs Naltrexone | 260 | 0.221 |
| Day 12 | RDC5 (0.6 mg/kg po)+Bupropion (20 mg/kg ip qd) vs RDC5 | 117 | 0.610 |
|  | RDC5 (0.6 mg/kg po)+Naltrexone (1 mg/kg ip) vs RDC5 | 230 | 0.313 |
|  | RDC5 (0.6 mg/kg po)+Bupropion (20 mg/kg ip qd) vs Bupropion | -554 | 0.018* |
|  | Bupropion (20 mg/kg ip qd)+Naltrexone (1 mg/kg ip) vs Bupropion | 160 | 0.483 |
|  | RDC5 (0.6 mg/kg po)+Naltrexone (1 mg/kg ip) vs Naltrexone | -314 | 0.171 |
|  | Bupropion (20 mg/kg ip qd)+Naltrexone (1 mg/kg ip) vs Naltrexone | 287 | 0.211 |
| Day 13 | RDC5 (0.6 mg/kg po)+Bupropion (20 mg/kg ip qd) vs RDC5 | 141 | 0.566 |
|  | RDC5 (0.6 mg/kg po)+Naltrexone (1 mg/kg ip) vs RDC5 | 276 | 0.263 |
|  | RDC5 (0.6 mg/kg po)+Bupropion (20 mg/kg ip qd) vs Bupropion | -614 | 0.015* |
|  | Bupropion (20 mg/kg ip qd)+Naltrexone (1 mg/kg ip) vs Bupropion | 164 | 0.504 |
|  | RDC5 (0.6 mg/kg po)+Naltrexone (1 mg/kg ip) vs Naltrexone | -325 | 0.188 |
|  | Bupropion (20 mg/kg ip qd)+Naltrexone (1 mg/kg ip) vs Naltrexone | 318 | 0.197 |
| Day 14 | RDC5 (0.6 mg/kg po)+Bupropion (20 mg/kg ip qd) vs RDC5 | 195 | 0.463 |
|  | RDC5 (0.6 mg/kg po)+Naltrexone (1 mg/kg ip) vs RDC5 | 384 | 0.149 |
|  | RDC5 (0.6 mg/kg po)+Bupropion (20 mg/kg ip qd) vs Bupropion | -635 | 0.019* |
|  | Bupropion (20 mg/kg ip qd)+Naltrexone (1 mg/kg ip) vs Bupropion | 193 | 0.467 |
|  | RDC5 (0.6 mg/kg po)+Naltrexone (1 mg/kg ip) vs Naltrexone | -285 | 0.283 |
|  | Bupropion (20 mg/kg ip qd)+Naltrexone (1 mg/kg ip) vs Naltrexone | 353 | 0.184 |

Multiple comparisons are by the multiple t test. *p<0.05.

| Time | Comparison | Difference | p |
| --- | --- | --- | --- |
| Day 15 | RDC5 (0.6 mg/kg po)+Bupropion (20 mg/kg ip qd) vs RDC5 | 186 | 0.506 |
|  | RDC5 (0.6 mg/kg po)+Naltrexone (1 mg/kg ip) vs RDC5 | 423 | 0.133 |
|  | RDC5 (0.6 mg/kg po)+Bupropion (20 mg/kg ip qd) vs Bupropion | -707 | 0.014* |
|  | Bupropion (20 mg/kg ip qd)+Naltrexone (1 mg/kg ip) vs Bupropion | 146 | 0.602 |
|  | RDC5 (0.6 mg/kg po)+Naltrexone (1 mg/kg ip) vs Naltrexone | -308 | 0.271 |
|  | Bupropion (20 mg/kg ip qd)+Naltrexone (1 mg/kg ip) vs Naltrexone | 308 | 0.271 |
| Day 16 | RDC5 (0.6 mg/kg po)+Bupropion (20 mg/kg ip qd) vs RDC5 | 138 | 0.639 |
|  | RDC5 (0.6 mg/kg po)+Naltrexone (1 mg/kg ip) vs RDC5 | 423 | 0.154 |
|  | RDC5 (0.6 mg/kg po)+Bupropion (20 mg/kg ip qd) vs Bupropion | -761 | 0.012* |
|  | Bupropion (20 mg/kg ip qd)+Naltrexone (1 mg/kg ip) vs Bupropion | 184 | 0.534 |
|  | RDC5 (0.6 mg/kg po)+Naltrexone (1 mg/kg ip) vs Naltrexone | -367 | 0.216 |
|  | Bupropion (20 mg/kg ip qd)+Naltrexone (1 mg/kg ip) vs Naltrexone | 293 | 0.321 |
| Day 17 | RDC5 (0.6 mg/kg po)+Bupropion (20 mg/kg ip qd) vs RDC5 | 170 | 0.594 |
|  | RDC5 (0.6 mg/kg po)+Naltrexone (1 mg/kg ip) vs RDC5 | 495 | 0.123 |
|  | RDC5 (0.6 mg/kg po)+Bupropion (20 mg/kg ip qd) vs Bupropion | -777 | 0.017* |
|  | Bupropion (20 mg/kg ip qd)+Naltrexone (1 mg/kg ip) vs Bupropion | 275 | 0.389 |
|  | RDC5 (0.6 mg/kg po)+Naltrexone (1 mg/kg ip) vs Naltrexone | -322 | 0.313 |
|  | Bupropion (20 mg/kg ip qd)+Naltrexone (1 mg/kg ip) vs Naltrexone | 406 | 0.204 |
| Day 18 | RDC5 (0.6 mg/kg po)+Bupropion (20 mg/kg ip qd) vs RDC5 | 184 | 0.579 |
|  | RDC5 (0.6 mg/kg po)+Naltrexone (1 mg/kg ip) vs RDC5 | 555 | 0.098 |
|  | RDC5 (0.6 mg/kg po)+Bupropion (20 mg/kg ip qd) vs Bupropion | -857 | 0.012* |
|  | Bupropion (20 mg/kg ip qd)+Naltrexone (1 mg/kg ip) vs Bupropion | 266 | 0.425 |
|  | RDC5 (0.6 mg/kg po)+Naltrexone (1 mg/kg ip) vs Naltrexone | -334 | 0.316 |
|  | Bupropion (20 mg/kg ip qd)+Naltrexone (1 mg/kg ip) vs Naltrexone | 418 | 0.210 |
| Day 19 | RDC5 (0.6 mg/kg po)+Bupropion (20 mg/kg ip qd) vs RDC5 | 164 | 0.632 |
|  | RDC5 (0.6 mg/kg po)+Naltrexone (1 mg/kg ip) vs RDC5 | 591 | 0.088 |
|  | RDC5 (0.6 mg/kg po)+Bupropion (20 mg/kg ip qd) vs Bupropion | -950 | 0.007** |
|  | Bupropion (20 mg/kg ip qd)+Naltrexone (1 mg/kg ip) vs Bupropion | 219 | 0.524 |
|  | RDC5 (0.6 mg/kg po)+Naltrexone (1 mg/kg ip) vs Naltrexone | -343 | 0.319 |
|  | Bupropion (20 mg/kg ip qd)+Naltrexone (1 mg/kg ip) vs Naltrexone | 399 | 0.247 |
| Day 20 | RDC5 (0.6 mg/kg po)+Bupropion (20 mg/kg ip qd) vs RDC5 | 145 | 0.681 |
|  | RDC5 (0.6 mg/kg po)+Naltrexone (1 mg/kg ip) vs RDC5 | 584 | 0.101 |
|  | RDC5 (0.6 mg/kg po)+Bupropion (20 mg/kg ip qd) vs Bupropion | -982 | 0.007** |
|  | Bupropion (20 mg/kg ip qd)+Naltrexone (1 mg/kg ip) vs Bupropion | 208 | 0.557 |
|  | RDC5 (0.6 mg/kg po)+Naltrexone (1 mg/kg ip) vs Naltrexone | -362 | 0.308 |
|  | Bupropion (20 mg/kg ip qd)+Naltrexone (1 mg/kg ip) vs Naltrexone | 390 | 0.271 |
| Day 21 | RDC5 (0.6 mg/kg po)+Bupropion (20 mg/kg ip qd) vs RDC5 | 129 | 0.725 |
|  | RDC5 (0.6 mg/kg po)+Naltrexone (1 mg/kg ip) vs RDC5 | 587 | 0.113 |
|  | RDC5 (0.6 mg/kg po)+Bupropion (20 mg/kg ip qd) vs Bupropion | -978 | 0.010** |
|  | Bupropion (20 mg/kg ip qd)+Naltrexone (1 mg/kg ip) vs Bupropion | 241 | 0.513 |
|  | RDC5 (0.6 mg/kg po)+Naltrexone (1 mg/kg ip) vs Naltrexone | -361 | 0.327 |
|  | Bupropion (20 mg/kg ip qd)+Naltrexone (1 mg/kg ip) vs Naltrexone | 399 | 0.279 |

Multiple comparisons are by the multiple t test. *p<0.05, **p<0.01.

| Time | Comparison | Difference | p |
| --- | --- | --- | --- |
| Day 22 | RDC5 (0.6 mg/kg po)+Bupropion (20 mg/kg ip qd) vs RDC5 | 157 | 0.681 |
|  | RDC5 (0.6 mg/kg po)+Naltrexone (1 mg/kg ip) vs RDC5 | 629 | 0.102 |
|  | RDC5 (0.6 mg/kg po)+Bupropion (20 mg/kg ip qd) vs Bupropion | -977 | 0.013* |
|  | Bupropion (20 mg/kg ip qd)+Naltrexone (1 mg/kg ip) vs Bupropion | 222 | 0.560 |
|  | RDC5 (0.6 mg/kg po)+Naltrexone (1 mg/kg ip) vs Naltrexone | -351 | 0.359 |
|  | Bupropion (20 mg/kg ip qd)+Naltrexone (1 mg/kg ip) vs Naltrexone | 377 | 0.324 |
| Day 23 | RDC5 (0.6 mg/kg po)+Bupropion (20 mg/kg ip qd) vs RDC5 | 160 | 0.683 |
|  | RDC5 (0.6 mg/kg po)+Naltrexone (1 mg/kg ip) vs RDC5 | 642 | 0.105 |
|  | RDC5 (0.6 mg/kg po)+Bupropion (20 mg/kg ip qd) vs Bupropion | -1012 | 0.012* |
|  | Bupropion (20 mg/kg ip qd)+Naltrexone (1 mg/kg ip) vs Bupropion | 192 | 0.626 |
|  | RDC5 (0.6 mg/kg po)+Naltrexone (1 mg/kg ip) vs Naltrexone | -392 | 0.319 |
|  | Bupropion (20 mg/kg ip qd)+Naltrexone (1 mg/kg ip) vs Naltrexone | 330 | 0.402 |
| Day 24 | RDC5 (0.6 mg/kg po)+Bupropion (20 mg/kg ip qd) vs RDC5 | 189 | 0.639 |
|  | RDC5 (0.6 mg/kg po)+Naltrexone (1 mg/kg ip) vs RDC5 | 641 | 0.115 |
|  | RDC5 (0.6 mg/kg po)+Bupropion (20 mg/kg ip qd) vs Bupropion | -1039 | 0.012* |
|  | Bupropion (20 mg/kg ip qd)+Naltrexone (1 mg/kg ip) vs Bupropion | 180 | 0.656 |
|  | RDC5 (0.6 mg/kg po)+Naltrexone (1 mg/kg ip) vs Naltrexone | -397 | 0.327 |
|  | Bupropion (20 mg/kg ip qd)+Naltrexone (1 mg/kg ip) vs Naltrexone | 370 | 0.360 |
| Day 25 | RDC5 (0.6 mg/kg po)+Bupropion (20 mg/kg ip qd) vs RDC5 | 200 | 0.631 |
|  | RDC5 (0.6 mg/kg po)+Naltrexone (1 mg/kg ip) vs RDC5 | 606 | 0.149 |
|  | RDC5 (0.6 mg/kg po)+Bupropion (20 mg/kg ip qd) vs Bupropion | -1056 | 0.014* |
|  | Bupropion (20 mg/kg ip qd)+Naltrexone (1 mg/kg ip) vs Bupropion | 166 | 0.691 |
|  | RDC5 (0.6 mg/kg po)+Naltrexone (1 mg/kg ip) vs Naltrexone | -449 | 0.283 |
|  | Bupropion (20 mg/kg ip qd)+Naltrexone (1 mg/kg ip) vs Naltrexone | 368 | 0.378 |
| Day 26 | RDC5 (0.6 mg/kg po)+Bupropion (20 mg/kg ip bid) vs RDC5 | 220 | 0.613 |
|  | RDC5 (0.6 mg/kg po)+Naltrexone (1 mg/kg ip) vs RDC5 | 615 | 0.159 |
|  | RDC5 (0.6 mg/kg po)+Bupropion (20 mg/kg ip bid) vs Bupropion | -1056 | 0.018* |
|  | Bupropion (20 mg/kg ip bid)+Naltrexone (1 mg/kg ip) vs Bupropion | 149 | 0.732 |
|  | RDC5 (0.6 mg/kg po)+Naltrexone (1 mg/kg ip) vs Naltrexone | -478 | 0.273 |
|  | Bupropion (20 mg/kg ip bid)+Naltrexone (1 mg/kg ip) vs Naltrexone | 332 | 0.445 |
| Day 27 | RDC5 (0.6 mg/kg po)+Bupropion (20 mg/kg ip bid) vs RDC5 | 259 | 0.563 |
|  | RDC5 (0.6 mg/kg po)+Naltrexone (1 mg/kg ip) vs RDC5 | 682 | 0.131 |
|  | RDC5 (0.6 mg/kg po)+Bupropion (20 mg/kg ip bid) vs Bupropion | -1081 | 0.019* |
|  | Bupropion (20 mg/kg ip bid)+Naltrexone (1 mg/kg ip) vs Bupropion | 95 | 0.833 |
|  | RDC5 (0.6 mg/kg po)+Naltrexone (1 mg/kg ip) vs Naltrexone | -471 | 0.295 |
|  | Bupropion (20 mg/kg ip bid)+Naltrexone (1 mg/kg ip) vs Naltrexone | 283 | 0.528 |
| Day 28 | RDC5 (0.6 mg/kg po)+Bupropion (20 mg/kg ip bid) vs RDC5 | 258 | 0.573 |
|  | RDC5 (0.6 mg/kg po)+Naltrexone (1 mg/kg ip) vs RDC5 | 698 | 0.129 |
|  | RDC5 (0.6 mg/kg po)+Bupropion (20 mg/kg ip bid) vs Bupropion | -1082 | 0.021* |
|  | Bupropion (20 mg/kg ip bid)+Naltrexone (1 mg/kg ip) vs Bupropion | 102 | 0.823 |
|  | RDC5 (0.6 mg/kg po)+Naltrexone (1 mg/kg ip) vs Naltrexone | -477 | 0.298 |
|  | Bupropion (20 mg/kg ip bid)+Naltrexone (1 mg/kg ip) vs Naltrexone | 267 | 0.560 |

Multiple comparisons are by the multiple t test. *p<0.05.

| Time | Comparison | Difference | p |
| --- | --- | --- | --- |
| Day 29 | RDC5 (0.6 mg/kg po)+Bupropion (20 mg/kg ip bid) vs RDC5 | 283 | 0.544 |
|  | RDC5 (0.6 mg/kg po)+Naltrexone (1 mg/kg ip) vs RDC5 | 653 | 0.163 |
|  | RDC5 (0.6 mg/kg po)+Bupropion (20 mg/kg ip bid) vs Bupropion | -1062 | 0.026* |
|  | Bupropion (20 mg/kg ip bid)+Naltrexone (1 mg/kg ip) vs Bupropion | 115 | 0.805 |
|  | RDC5 (0.6 mg/kg po)+Naltrexone (1 mg/kg ip) vs Naltrexone | -488 | 0.296 |
|  | Bupropion (20 mg/kg ip bid)+Naltrexone (1 mg/kg ip) vs Naltrexone | 318 | 0.495 |
| Day 30 | RDC5 (0.6 mg/kg po)+Bupropion (20 mg/kg ip bid) vs RDC5 | 290 | 0.550 |
|  | RDC5 (0.6 mg/kg po)+Naltrexone (1 mg/kg ip) vs RDC5 | 686 | 0.160 |
|  | RDC5 (0.6 mg/kg po)+Bupropion (20 mg/kg ip bid) vs Bupropion | -1049 | 0.035* |
|  | Bupropion (20 mg/kg ip bid)+Naltrexone (1 mg/kg ip) vs Bupropion | 119 | 0.807 |
|  | RDC5 (0.6 mg/kg po)+Naltrexone (1 mg/kg ip) vs Naltrexone | -447 | 0.358 |
|  | Bupropion (20 mg/kg ip bid)+Naltrexone (1 mg/kg ip) vs Naltrexone | 325 | 0.503 |
| Day 31 | RDC5 (0.6 mg/kg po)+Bupropion (20 mg/kg ip bid) vs RDC5 | 343 | 0.499 |
|  | RDC5 (0.6 mg/kg po)+Naltrexone (1 mg/kg ip) vs RDC5 | 771 | 0.131 |
|  | RDC5 (0.6 mg/kg po)+Bupropion (20 mg/kg ip bid) vs Bupropion | -1051 | 0.043* |
|  | Bupropion (20 mg/kg ip bid)+Naltrexone (1 mg/kg ip) vs Bupropion | 132 | 0.795 |
|  | RDC5 (0.6 mg/kg po)+Naltrexone (1 mg/kg ip) vs Naltrexone | -416 | 0.413 |
|  | Bupropion (20 mg/kg ip bid)+Naltrexone (1 mg/kg ip) vs Naltrexone | 339 | 0.504 |
| Day 32 | RDC5 (0.6 mg/kg po)+Bupropion (20 mg/kg ip bid) vs RDC5 | 390 | 0.459 |
|  | RDC5 (0.6 mg/kg po)+Naltrexone (1 mg/kg ip) vs RDC5 | 852 | 0.107 |
|  | RDC5 (0.6 mg/kg po)+Bupropion (20 mg/kg ip bid) vs Bupropion | -1066 | 0.047* |
|  | Bupropion (20 mg/kg ip bid)+Naltrexone (1 mg/kg ip) vs Bupropion | 109 | 0.836 |
|  | RDC5 (0.6 mg/kg po)+Naltrexone (1 mg/kg ip) vs Naltrexone | -422 | 0.422 |
|  | Bupropion (20 mg/kg ip bid)+Naltrexone (1 mg/kg ip) vs Naltrexone | 290 | 0.581 |
| Day 33 | RDC5 (0.6 mg/kg po)+Bupropion (20 mg/kg ip bid) vs RDC5 | 390 | 0.473 |
|  | RDC5 (0.6 mg/kg po)+Naltrexone (1 mg/kg ip) vs RDC5 | 877 | 0.109 |
|  | RDC5 (0.6 mg/kg po)+Bupropion (20 mg/kg ip bid) vs Bupropion | -1080 | 0.052 |
|  | Bupropion (20 mg/kg ip bid)+Naltrexone (1 mg/kg ip) vs Bupropion | 87 | 0.873 |
|  | RDC5 (0.6 mg/kg po)+Naltrexone (1 mg/kg ip) vs Naltrexone | -395 | 0.467 |
|  | Bupropion (20 mg/kg ip bid)+Naltrexone (1 mg/kg ip) vs Naltrexone | 285 | 0.600 |
| Day 34 | RDC5 (0.6 mg/kg po)+Bupropion (20 mg/kg ip bid) vs RDC5 | 417 | 0.457 |
|  | RDC5 (0.6 mg/kg po)+Naltrexone (1 mg/kg ip) vs RDC5 | 872 | 0.121 |
|  | RDC5 (0.6 mg/kg po)+Bupropion (20 mg/kg ip bid) vs Bupropion | -1049 | 0.066 |
|  | Bupropion (20 mg/kg ip bid)+Naltrexone (1 mg/kg ip) vs Bupropion | 81 | 0.885 |
|  | RDC5 (0.6 mg/kg po)+Naltrexone (1 mg/kg ip) vs Naltrexone | -329 | 0.556 |
|  | Bupropion (20 mg/kg ip bid)+Naltrexone (1 mg/kg ip) vs Naltrexone | 345 | 0.537 |
| Day 35 | RDC5 (0.6 mg/kg po)+Bupropion (20 mg/kg ip bid) vs RDC5 | 391 | 0.495 |
|  | RDC5 (0.6 mg/kg po)+Naltrexone (1 mg/kg ip) vs RDC5 | 871 | 0.131 |
|  | RDC5 (0.6 mg/kg po)+Bupropion (20 mg/kg ip bid) vs Bupropion | -1030 | 0.077 |
|  | Bupropion (20 mg/kg ip bid)+Naltrexone (1 mg/kg ip) vs Bupropion | 39 | 0.945 |
|  | RDC5 (0.6 mg/kg po)+Naltrexone (1 mg/kg ip) vs Naltrexone | -290 | 0.612 |
|  | Bupropion (20 mg/kg ip bid)+Naltrexone (1 mg/kg ip) vs Naltrexone | 300 | 0.600 |

Multiple comparisons are by the multiple t test. *p<0.05.

| Time | Comparison | Difference | p |
| --- | --- | --- | --- |
| Day 36 | RDC5 (0.6 mg/kg po)+Bupropion (20 mg/kg ip bid) vs RDC5 | 387 | 0.502 |
|  | RDC5 (0.6 mg/kg po)+Naltrexone (1 mg/kg ip) vs RDC5 | 891 | 0.124 |
|  | RDC5 (0.6 mg/kg po)+Bupropion (20 mg/kg ip bid) vs Bupropion | -1034 | 0.078 |
|  | Bupropion (20 mg/kg ip bid)+Naltrexone (1 mg/kg ip) vs Bupropion | 12 | 0.983 |
|  | RDC5 (0.6 mg/kg po)+Naltrexone (1 mg/kg ip) vs Naltrexone | -250 | 0.665 |
|  | Bupropion (20 mg/kg ip bid)+Naltrexone (1 mg/kg ip) vs Naltrexone | 292 | 0.612 |
| Day 37 | RDC5 (0.6 mg/kg po)+Bupropion (20 mg/kg ip bid) vs RDC5 | 362 | 0.537 |
|  | RDC5 (0.6 mg/kg po)+Naltrexone (1 mg/kg ip) vs RDC5 | 914 | 0.121 |
|  | RDC5 (0.6 mg/kg po)+Bupropion (20 mg/kg ip bid) vs Bupropion | -1061 | 0.076 |
|  | Bupropion (20 mg/kg ip bid)+Naltrexone (1 mg/kg ip) vs Bupropion | 9 | 0.988 |
|  | RDC5 (0.6 mg/kg po)+Naltrexone (1 mg/kg ip) vs Naltrexone | -209 | 0.721 |
|  | Bupropion (20 mg/kg ip bid)+Naltrexone (1 mg/kg ip) vs Naltrexone | 309 | 0.598 |
| Day 38 | RDC5 (0.6 mg/kg po)+Bupropion (20 mg/kg ip bid) vs RDC5 | 346 | 0.560 |
|  | RDC5 (0.6 mg/kg po)+Naltrexone (1 mg/kg ip) vs RDC5 | 920 | 0.123 |
|  | RDC5 (0.6 mg/kg po)+Bupropion (20 mg/kg ip bid) vs Bupropion | -1086 | 0.072 |
|  | Bupropion (20 mg/kg ip bid)+Naltrexone (1 mg/kg ip) vs Bupropion | -6 | 0.992 |
|  | RDC5 (0.6 mg/kg po)+Naltrexone (1 mg/kg ip) vs Naltrexone | -175 | 0.767 |
|  | Bupropion (20 mg/kg ip bid)+Naltrexone (1 mg/kg ip) vs Naltrexone | 331 | 0.577 |
| Day 39 | RDC5 (0.6 mg/kg po)+Bupropion (20 mg/kg ip bid) vs RDC5 | 311 | 0.605 |
|  | RDC5 (0.6 mg/kg po)+Naltrexone (1 mg/kg ip) vs RDC5 | 960 | 0.113 |
|  | RDC5 (0.6 mg/kg po)+Bupropion (20 mg/kg ip bid) vs Bupropion | -1112 | 0.070 |
|  | Bupropion (20 mg/kg ip bid)+Naltrexone (1 mg/kg ip) vs Bupropion | -38 | 0.949 |
|  | RDC5 (0.6 mg/kg po)+Naltrexone (1 mg/kg ip) vs Naltrexone | -100 | 0.868 |
|  | Bupropion (20 mg/kg ip bid)+Naltrexone (1 mg/kg ip) vs Naltrexone | 325 | 0.589 |
| Day 40 | RDC5 (0.6 mg/kg po)+Bupropion (20 mg/kg ip bid) vs RDC5 | 215 | 0.724 |
|  | RDC5 (0.6 mg/kg po)+Naltrexone (1 mg/kg ip) vs RDC5 | 955 | 0.120 |
|  | RDC5 (0.6 mg/kg po)+Bupropion (20 mg/kg ip bid) vs Bupropion | -1176 | 0.059 |
|  | Bupropion (20 mg/kg ip bid)+Naltrexone (1 mg/kg ip) vs Bupropion | -76 | 0.900 |
|  | RDC5 (0.6 mg/kg po)+Naltrexone (1 mg/kg ip) vs Naltrexone | -86 | 0.887 |
|  | Bupropion (20 mg/kg ip bid)+Naltrexone (1 mg/kg ip) vs Naltrexone | 273 | 0.654 |
| Day 41 | RDC5 (0.6 mg/kg po)+Bupropion (20 mg/kg ip bid) vs RDC5 | 112 | 0.855 |
|  | RDC5 (0.6 mg/kg po)+Naltrexone (1 mg/kg ip) vs RDC5 | 931 | 0.133 |
|  | RDC5 (0.6 mg/kg po)+Bupropion (20 mg/kg ip bid) vs Bupropion | -1240 | 0.049* |
|  | Bupropion (20 mg/kg ip bid)+Naltrexone (1 mg/kg ip) vs Bupropion | -93 | 0.880 |
|  | RDC5 (0.6 mg/kg po)+Naltrexone (1 mg/kg ip) vs Naltrexone | -80 | 0.896 |
|  | Bupropion (20 mg/kg ip bid)+Naltrexone (1 mg/kg ip) vs Naltrexone | 247 | 0.687 |
| Day 42 | RDC5 (0.6 mg/kg po)+Bupropion (20 mg/kg ip bid) vs RDC5 | 85 | 0.891 |
|  | RDC5 (0.6 mg/kg po)+Naltrexone (1 mg/kg ip) vs RDC5 | 951 | 0.127 |
|  | RDC5 (0.6 mg/kg po)+Bupropion (20 mg/kg ip bid) vs Bupropion | -1251 | 0.048* |
|  | Bupropion (20 mg/kg ip bid)+Naltrexone (1 mg/kg ip) vs Bupropion | -83 | 0.894 |
|  | RDC5 (0.6 mg/kg po)+Naltrexone (1 mg/kg ip) vs Naltrexone | -53 | 0.931 |
|  | Bupropion (20 mg/kg ip bid)+Naltrexone (1 mg/kg ip) vs Naltrexone | 249 | 0.687 |

Multiple comparisons are by the multiple t test. *p<0.05.

#### their individual constituents (Baseline, Weeks 1 to 6) Water intake (g) on each day (Baseline)

Treatment n Mean SEM Difference % change p from vehicle

| Day -6 | Vehicle 4 ml/kg po +2 ml/kg ip | 12 | 28.0 | 0.9 |  | | |
| --- | --- | --- | --- | --- | --- | --- | --- |
|  | RDC5 0.6 mg/kg po | 10 | 25.6 | 3.0 | -2.4 | -8.5 | 0.114 |
|  | Bupropion 20 mg/kg ip qd | 10 | 24.4 | 1.1 | -3.6 | -12.8 | 0.018* |
|  | Naltrexone 1 mg/kg ip | 10 | 27.9 | 1.3 | -0.1 | -0.3 | 0.959 |
|  | RDC5 0.6 mg/kg po+Bupropion 20 mg/kg ip qd | 10 | 25.2 | 1.5 | -2.8 | -10.1 | 0.062 |
|  | RDC5 0.6 mg/kg po+Naltrexone 1 mg/kg ip | 10 | 24.8 | 0.7 | -3.1 | -11.2 | 0.037* |
|  | Bupropion 20 mg/kg ip qd+Naltrexone 1 mg/kg ip | 10 | 27.1 | 1.0 | -0.9 | -3.2 | 0.545 |
|  | Sibutramine 5 mg/kg po | 10 | 24.1 | 1.1 | -3.9 | -13.8 | 0.011* |
| Day -5 | Vehicle 4 ml/kg po +2 ml/kg ip | 12 | 25.1 | 1.0 |  |  |  |
|  | RDC5 0.6 mg/kg po | 10 | 24.5 | 0.6 | -0.6 | -2.5 | 0.686 |
|  | Bupropion 20 mg/kg ip qd | 10 | 24.1 | 1.2 | -1.0 | -4.0 | 0.521 |
|  | Naltrexone 1 mg/kg ip | 10 | 24.7 | 1.4 | -0.4 | -1.8 | 0.775 |
|  | RDC5 0.6 mg/kg po+Bupropion 20 mg/kg ip qd | 10 | 24.9 | 1.1 | -0.2 | -0.9 | 0.885 |
|  | RDC5 0.6 mg/kg po+Naltrexone 1 mg/kg ip | 10 | 25.7 | 0.7 | 0.6 | 2.2 | 0.724 |
|  | Bupropion 20 mg/kg ip qd+Naltrexone 1 mg/kg ip | 10 | 26.2 | 1.8 | 1.1 | 4.4 | 0.483 |
|  | Sibutramine 5 mg/kg po | 10 | 24.7 | 1.0 | -0.4 | -1.8 | 0.775 |
| Day -4 | Vehicle 4 ml/kg po +2 ml/kg ip | 12 | 26.1 | 1.0 |  |  |  |
|  | RDC5 0.6 mg/kg po | 10 | 26.2 | 0.6 | 0.1 | 0.5 | 0.922 |
|  | Bupropion 20 mg/kg ip qd | 10 | 27.8 | 0.7 | 1.7 | 6.5 | 0.228 |
|  | Naltrexone 1 mg/kg ip | 10 | 25.0 | 1.4 | -1.1 | -4.4 | 0.415 |
|  | RDC5 0.6 mg/kg po+Bupropion 20 mg/kg ip qd | 10 | 26.6 | 0.7 | 0.5 | 1.9 | 0.718 |
|  | RDC5 0.6 mg/kg po+Naltrexone 1 mg/kg ip | 10 | 26.4 | 1.1 | 0.3 | 1.0 | 0.853 |
|  | Bupropion 20 mg/kg ip qd+Naltrexone 1 mg/kg ip | 10 | 27.4 | 1.4 | 1.3 | 4.9 | 0.358 |
|  | Sibutramine 5 mg/kg po | 10 | 26.6 | 1.0 | 0.5 | 2.1 | 0.700 |
| Day -3 | Vehicle 4 ml/kg po +2 ml/kg ip | 12 | 25.6 | 0.9 |  |  |  |
|  | RDC5 0.6 mg/kg po | 10 | 25.7 | 0.9 | 0.1 | 0.4 | 0.948 |
|  | Bupropion 20 mg/kg ip qd | 10 | 24.8 | 1.5 | -0.8 | -3.2 | 0.590 |
|  | Naltrexone 1 mg/kg ip | 10 | 27.4 | 2.3 | 1.8 | 7.2 | 0.233 |
|  | RDC5 0.6 mg/kg po+Bupropion 20 mg/kg ip qd | 10 | 25.9 | 1.1 | 0.4 | 1.4 | 0.819 |
|  | RDC5 0.6 mg/kg po+Naltrexone 1 mg/kg ip | 10 | 24.8 | 1.2 | -0.8 | -3.1 | 0.604 |
|  | Bupropion 20 mg/kg ip qd+Naltrexone 1 mg/kg ip | 10 | 23.2 | 0.9 | -2.3 | -9.1 | 0.130 |
|  | Sibutramine 5 mg/kg po | 10 | 24.0 | 1.6 | -1.6 | -6.3 | 0.298 |
| Day -2 | Vehicle 4 ml/kg po +2 ml/kg ip | 12 | 25.6 | 1.1 |  |  |  |
|  | RDC5 0.6 mg/kg po | 10 | 25.4 | 1.4 | -0.2 | -0.6 | 0.916 |
|  | Bupropion 20 mg/kg ip qd | 10 | 24.7 | 1.6 | -0.9 | -3.6 | 0.529 |
|  | Naltrexone 1 mg/kg ip | 10 | 25.0 | 2.1 | -0.6 | -2.3 | 0.689 |
|  | RDC5 0.6 mg/kg po+Bupropion 20 mg/kg ip qd | 10 | 25.1 | 1.2 | -0.4 | -1.7 | 0.771 |
|  | RDC5 0.6 mg/kg po+Naltrexone 1 mg/kg ip | 10 | 24.3 | 1.2 | -1.3 | -5.1 | 0.373 |
|  | Bupropion 20 mg/kg ip qd+Naltrexone 1 mg/kg ip | 10 | 24.0 | 0.5 | -1.6 | -6.2 | 0.285 |
|  | Sibutramine 5 mg/kg po | 9 | 26.2 | 1.5 | 0.7 | 2.6 | 0.666 |
| Day -1 | Vehicle 4 ml/kg po +2 ml/kg ip | 12 | 24.2 | 1.2 |  |  |  |
|  | RDC5 0.6 mg/kg po | 10 | 25.1 | 0.6 | 0.9 | 3.6 | 0.551 |
|  | Bupropion 20 mg/kg ip qd | 10 | 27.5 | 0.8 | 3.3 | 13.7 | 0.024* |
|  | Naltrexone 1 mg/kg ip | 10 | 25.2 | 1.0 | 1.0 | 4.1 | 0.494 |
|  | RDC5 0.6 mg/kg po+Bupropion 20 mg/kg ip qd | 10 | 24.7 | 1.0 | 0.5 | 2.1 | 0.719 |
|  | RDC5 0.6 mg/kg po+Naltrexone 1 mg/kg ip | 10 | 28.3 | 1.7 | 4.1 | 16.8 | 0.006** |
|  | Bupropion 20 mg/kg ip qd+Naltrexone 1 mg/kg ip | 10 | 24.7 | 1.2 | 0.5 | 1.9 | 0.750 |
|  | Sibutramine 5 mg/kg po | 9 | 29.7 | 1.1 | 5.5 | 22.7 | <0.001*** |
| Day 0 | Vehicle 4 ml/kg po +2 ml/kg ip | 12 | 24.2 | 2.3 |  |  |  |
|  | RDC5 0.6 mg/kg po | 10 | 25.2 | 1.3 | 1.0 | 4.2 | 0.685 |
|  | Bupropion 20 mg/kg ip qd | 10 | 28.0 | 1.1 | 3.8 | 15.7 | 0.127 |
|  | Naltrexone 1 mg/kg ip | 10 | 25.6 | 1.1 | 1.4 | 5.9 | 0.568 |
|  | RDC5 0.6 mg/kg po+Bupropion 20 mg/kg ip qd | 10 | 26.8 | 1.9 | 2.6 | 10.6 | 0.303 |
|  | RDC5 0.6 mg/kg po+Naltrexone 1 mg/kg ip | 10 | 25.4 | 2.0 | 1.2 | 5.1 | 0.617 |
|  | Bupropion 20 mg/kg ip qd+Naltrexone 1 mg/kg ip | 10 | 26.8 | 2.3 | 2.6 | 10.7 | 0.297 |
|  | Sibutramine 5 mg/kg po | 10 | 25.5 | 2.5 | 1.3 | 5.5 | 0.594 |

Means are adjusted for differences between the treatment groups at baseline (average of Days -6 to 0). SEM are calculated from the residuals of the statistical model. Multiple comparisons against vehicle are by the multiple t test. *p<0.05, **p<0.01, ***p<0.001.

Treatment n Mean SEM Difference % change p

from vehicle

| Day 1 | Vehicle 4 ml/kg po +2 ml/kg ip | 12 | 24.5 | 1.2 | | | |
| --- | --- | --- | --- | --- | --- | --- | --- |
|  | RDC5 0.6 mg/kg po | 10 | 20.8 | 1.7 | -3.7 | -15.2 | 0.081 |
|  | Bupropion 20 mg/kg ip qd | 10 | 26.2 | 1.7 | 1.8 | 7.3 | 0.401 |
|  | Naltrexone 1 mg/kg ip | 10 | 23.8 | 1.1 | -0.6 | -2.6 | 0.759 |
|  | RDC5 0.6 mg/kg po+Bupropion 20 mg/kg ip qd | 10 | 23.9 | 1.3 | -0.6 | -2.3 | 0.789 |
|  | RDC5 0.6 mg/kg po+Naltrexone 1 mg/kg ip | 10 | 19.0 | 1.3 | -5.5 | -22.3 | 0.012* |
|  | Bupropion 20 mg/kg ip qd+Naltrexone 1 mg/kg ip | 10 | 27.5 | 1.9 | 3.1 | 12.5 | 0.150 |
|  | Sibutramine 5 mg/kg po | 10 | 10.6 | 1.9 | -13.8 | -56.5 | <0.001*** |
| Day 2 | Vehicle 4 ml/kg po +2 ml/kg ip | 12 | 23.7 | 1.8 |  |  |  |
|  | RDC5 0.6 mg/kg po | 10 | 20.1 | 1.1 | -3.6 | -15.2 | 0.078 |
|  | Bupropion 20 mg/kg ip qd | 10 | 29.3 | 1.5 | 5.6 | 23.7 | 0.007** |
|  | Naltrexone 1 mg/kg ip | 10 | 24.2 | 1.0 | 0.5 | 2.1 | 0.808 |
|  | RDC5 0.6 mg/kg po+Bupropion 20 mg/kg ip qd | 10 | 21.1 | 1.2 | -2.6 | -10.8 | 0.209 |
|  | RDC5 0.6 mg/kg po+Naltrexone 1 mg/kg ip | 10 | 19.4 | 1.6 | -4.3 | -18.0 | 0.039* |
|  | Bupropion 20 mg/kg ip qd+Naltrexone 1 mg/kg ip | 10 | 26.8 | 0.9 | 3.1 | 12.9 | 0.133 |
|  | Sibutramine 5 mg/kg po | 10 | 18.3 | 2.2 | -5.4 | -22.8 | 0.009** |
| Day 3 | Vehicle 4 ml/kg po +2 ml/kg ip | 12 | 25.9 | 1.0 |  |  |  |
|  | RDC5 0.6 mg/kg po | 10 | 20.7 | 1.9 | -5.2 | -20.1 | 0.022* |
|  | Bupropion 20 mg/kg ip qd | 10 | 26.8 | 1.8 | 0.9 | 3.3 | 0.700 |
|  | Naltrexone 1 mg/kg ip | 10 | 23.7 | 1.2 | -2.2 | -8.5 | 0.328 |
|  | RDC5 0.6 mg/kg po+Bupropion 20 mg/kg ip qd | 10 | 22.4 | 1.3 | -3.5 | -13.6 | 0.119 |
|  | RDC5 0.6 mg/kg po+Naltrexone 1 mg/kg ip | 10 | 22.3 | 1.2 | -3.6 | -13.9 | 0.112 |
|  | Bupropion 20 mg/kg ip qd+Naltrexone 1 mg/kg ip | 10 | 27.0 | 1.9 | 1.1 | 4.2 | 0.627 |
|  | Sibutramine 5 mg/kg po | 10 | 23.1 | 2.6 | -2.9 | -11.0 | 0.204 |
| Day 4 | Vehicle 4 ml/kg po +2 ml/kg ip | 12 | 26.0 | 1.5 |  |  |  |
|  | RDC5 0.6 mg/kg po | 10 | 22.6 | 1.0 | -3.5 | -13.3 | 0.135 |
|  | Bupropion 20 mg/kg ip qd | 10 | 27.9 | 1.6 | 1.9 | 7.1 | 0.422 |
|  | Naltrexone 1 mg/kg ip | 10 | 22.8 | 1.7 | -3.2 | -12.2 | 0.169 |
|  | RDC5 0.6 mg/kg po+Bupropion 20 mg/kg ip qd | 10 | 24.4 | 1.7 | -1.6 | -6.1 | 0.488 |
|  | RDC5 0.6 mg/kg po+Naltrexone 1 mg/kg ip | 10 | 25.0 | 1.9 | -1.0 | -3.8 | 0.671 |
|  | Bupropion 20 mg/kg ip qd+Naltrexone 1 mg/kg ip | 10 | 25.2 | 1.4 | -0.8 | -3.1 | 0.724 |
|  | Sibutramine 5 mg/kg po | 10 | 23.4 | 1.8 | -2.6 | -10.0 | 0.263 |
| Day 5 | Vehicle 4 ml/kg po +2 ml/kg ip | 12 | 26.1 | 0.7 |  |  |  |
|  | RDC5 0.6 mg/kg po | 10 | 21.8 | 1.0 | -4.3 | -16.6 | 0.026* |
|  | Bupropion 20 mg/kg ip qd | 10 | 30.2 | 1.9 | 4.1 | 15.6 | 0.036* |
|  | Naltrexone 1 mg/kg ip | 10 | 23.1 | 1.0 | -3.1 | -11.8 | 0.111 |
|  | RDC5 0.6 mg/kg po+Bupropion 20 mg/kg ip qd | 10 | 24.2 | 1.2 | -2.0 | -7.5 | 0.308 |
|  | RDC5 0.6 mg/kg po+Naltrexone 1 mg/kg ip | 10 | 23.0 | 1.2 | -3.1 | -11.8 | 0.109 |
|  | Bupropion 20 mg/kg ip qd+Naltrexone 1 mg/kg ip | 10 | 24.6 | 1.3 | -1.5 | -5.8 | 0.427 |
|  | Sibutramine 5 mg/kg po | 10 | 25.6 | 2.2 | -0.5 | -2.0 | 0.781 |
| Day 6 | Vehicle 4 ml/kg po +2 ml/kg ip | 12 | 26.6 | 1.3 |  |  |  |
|  | RDC5 0.6 mg/kg po | 10 | 21.6 | 1.1 | -4.9 | -18.6 | 0.046* |
|  | Bupropion 20 mg/kg ip qd | 10 | 27.2 | 1.8 | 0.6 | 2.3 | 0.804 |
|  | Naltrexone 1 mg/kg ip | 10 | 24.7 | 2.5 | -1.9 | -7.0 | 0.447 |
|  | RDC5 0.6 mg/kg po+Bupropion 20 mg/kg ip qd | 10 | 23.4 | 2.3 | -3.2 | -11.9 | 0.198 |
|  | RDC5 0.6 mg/kg po+Naltrexone 1 mg/kg ip | 10 | 24.2 | 1.4 | -2.3 | -8.7 | 0.347 |
|  | Bupropion 20 mg/kg ip qd+Naltrexone 1 mg/kg ip | 10 | 24.7 | 1.4 | -1.8 | -6.9 | 0.452 |
|  | Sibutramine 5 mg/kg po | 10 | 24.1 | 3.8 | -2.4 | -9.2 | 0.319 |
| Day 7 | Vehicle 4 ml/kg po +2 ml/kg ip | 12 | 26.3 | 1.0 |  |  |  |
|  | RDC5 0.6 mg/kg po | 10 | 22.2 | 1.1 | -4.1 | -15.7 | 0.053 |
|  | Bupropion 20 mg/kg ip qd | 9 | 30.4 | 1.9 | 4.1 | 15.5 | 0.064 |
|  | Naltrexone 1 mg/kg ip | 10 | 24.1 | 1.4 | -2.3 | -8.6 | 0.288 |
|  | RDC5 0.6 mg/kg po+Bupropion 20 mg/kg ip qd | 10 | 23.7 | 2.2 | -2.7 | -10.2 | 0.208 |
|  | RDC5 0.6 mg/kg po+Naltrexone 1 mg/kg ip | 10 | 22.2 | 0.9 | -4.1 | -15.7 | 0.054 |
|  | Bupropion 20 mg/kg ip qd+Naltrexone 1 mg/kg ip | 10 | 24.1 | 1.6 | -2.2 | -8.4 | 0.299 |
|  | Sibutramine 5 mg/kg po | 10 | 29.0 | 4.4 | 2.7 | 10.3 | 0.204 |

Means are adjusted for differences between the treatment groups at baseline (average of Days -6 to 0). SEM are calculated from the residuals of the statistical model. Multiple comparisons against vehicle are by the multiple t test. *p<0.05, **p<0.01,

***p<0.001.

Treatment n Mean SEM Difference % change p

from vehicle

| Day 8 | Vehicle 4 ml/kg po +2 ml/kg ip | 12 | 25.7 | 1.4 |  |  |  |
| --- | --- | --- | --- | --- | --- | --- | --- |
|  | RDC5 0.6 mg/kg po | 10 | 22.5 | 1.4 | -3.2 | -12.5 | 0.199 |
|  | Bupropion 20 mg/kg ip qd | 10 | 26.1 | 1.9 | 0.4 | 1.7 | 0.860 |
|  | Naltrexone 1 mg/kg ip | 10 | 25.1 | 1.0 | -0.6 | -2.4 | 0.803 |
|  | RDC5 0.6 mg/kg po+Bupropion 20 mg/kg ip qd | 10 | 20.4 | 2.4 | -5.3 | -20.7 | 0.035* |
|  | RDC5 0.6 mg/kg po+Naltrexone 1 mg/kg ip | 10 | 23.6 | 1.9 | -2.1 | -8.3 | 0.391 |
|  | Bupropion 20 mg/kg ip qd+Naltrexone 1 mg/kg ip | 10 | 26.0 | 1.2 | 0.3 | 1.3 | 0.893 |
|  | Sibutramine 5 mg/kg po | 10 | 29.3 | 3.5 | 3.6 | 13.9 | 0.152 |
| Day 9 | Vehicle 4 ml/kg po +2 ml/kg ip | 12 | 26.8 | 1.6 |  |  |  |
|  | RDC5 0.6 mg/kg po | 10 | 21.0 | 1.4 | -5.8 | -21.5 | 0.006** |
|  | Bupropion 20 mg/kg ip qd | 10 | 27.5 | 0.8 | 0.8 | 2.9 | 0.700 |
|  | Naltrexone 1 mg/kg ip | 10 | 22.6 | 1.4 | -4.1 | -15.5 | 0.044* |
|  | RDC5 0.6 mg/kg po+Bupropion 20 mg/kg ip qd | 10 | 21.3 | 2.1 | -5.4 | -20.3 | 0.009** |
|  | RDC5 0.6 mg/kg po+Naltrexone 1 mg/kg ip | 10 | 23.6 | 2.0 | -3.2 | -11.9 | 0.120 |
|  | Bupropion 20 mg/kg ip qd+Naltrexone 1 mg/kg ip | 10 | 26.3 | 1.6 | -0.5 | -1.8 | 0.817 |
|  | Sibutramine 5 mg/kg po | 10 | 29.4 | 3.1 | 2.6 | 9.7 | 0.202 |
| Day 10 | Vehicle 4 ml/kg po +2 ml/kg ip | 12 | 26.9 | 2.3 |  |  |  |
|  | RDC5 0.6 mg/kg po | 10 | 23.9 | 1.1 | -3.0 | -11.1 | 0.189 |
|  | Bupropion 20 mg/kg ip qd | 10 | 27.5 | 1.1 | 0.6 | 2.2 | 0.796 |
|  | Naltrexone 1 mg/kg ip | 10 | 22.5 | 1.0 | -4.4 | -16.5 | 0.052 |
|  | RDC5 0.6 mg/kg po+Bupropion 20 mg/kg ip qd | 10 | 23.4 | 1.9 | -3.5 | -13.0 | 0.124 |
|  | RDC5 0.6 mg/kg po+Naltrexone 1 mg/kg ip | 10 | 23.3 | 1.6 | -3.6 | -13.4 | 0.116 |
|  | Bupropion 20 mg/kg ip qd+Naltrexone 1 mg/kg ip | 10 | 24.5 | 1.7 | -2.4 | -8.8 | 0.298 |
|  | Sibutramine 5 mg/kg po | 10 | 30.9 | 2.3 | 3.9 | 14.7 | 0.084 |
| Day 11 | Vehicle 4 ml/kg po +2 ml/kg ip | 12 | 27.8 | 1.8 |  |  |  |
|  | RDC5 0.6 mg/kg po | 10 | 26.1 | 1.7 | -1.6 | -5.9 | 0.486 |
|  | Bupropion 20 mg/kg ip qd | 10 | 29.4 | 2.0 | 1.6 | 5.8 | 0.494 |
|  | Naltrexone 1 mg/kg ip | 10 | 22.7 | 0.6 | -5.1 | -18.2 | 0.034* |
|  | RDC5 0.6 mg/kg po+Bupropion 20 mg/kg ip qd | 10 | 21.7 | 1.3 | -6.1 | -21.8 | 0.012* |
|  | RDC5 0.6 mg/kg po+Naltrexone 1 mg/kg ip | 10 | 22.9 | 1.8 | -4.8 | -17.3 | 0.044* |
|  | Bupropion 20 mg/kg ip qd+Naltrexone 1 mg/kg ip | 10 | 26.1 | 2.1 | -1.7 | -6.0 | 0.482 |
|  | Sibutramine 5 mg/kg po | 10 | 29.4 | 4.4 | 1.7 | 6.0 | 0.483 |
| Day 12 | Vehicle 4 ml/kg po +2 ml/kg ip | 12 | 25.5 | 1.8 |  |  |  |
|  | RDC5 0.6 mg/kg po | 10 | 23.9 | 1.1 | -1.6 | -6.2 | 0.548 |
|  | Bupropion 20 mg/kg ip qd | 10 | 28.5 | 1.9 | 3.1 | 12.1 | 0.241 |
|  | Naltrexone 1 mg/kg ip | 10 | 22.0 | 1.5 | -3.4 | -13.5 | 0.191 |
|  | RDC5 0.6 mg/kg po+Bupropion 20 mg/kg ip qd | 10 | 23.7 | 2.0 | -1.8 | -7.1 | 0.491 |
|  | RDC5 0.6 mg/kg po+Naltrexone 1 mg/kg ip | 10 | 24.5 | 3.1 | -1.0 | -3.8 | 0.714 |
|  | Bupropion 20 mg/kg ip qd+Naltrexone 1 mg/kg ip | 10 | 27.0 | 1.5 | 1.5 | 6.1 | 0.554 |
|  | Sibutramine 5 mg/kg po | 10 | 29.9 | 3.4 | 4.5 | 17.6 | 0.088 |
| Day 13 | Vehicle 4 ml/kg po +2 ml/kg ip | 12 | 27.7 | 1.4 |  |  |  |
|  | RDC5 0.6 mg/kg po | 10 | 21.9 | 1.9 | -5.8 | -20.8 | 0.041* |
|  | Bupropion 20 mg/kg ip qd | 10 | 28.0 | 2.2 | 0.3 | 1.1 | 0.911 |
|  | Naltrexone 1 mg/kg ip | 10 | 22.6 | 1.8 | -5.1 | -18.6 | 0.067 |
|  | RDC5 0.6 mg/kg po+Bupropion 20 mg/kg ip qd | 10 | 24.6 | 2.6 | -3.1 | -11.2 | 0.265 |
|  | RDC5 0.6 mg/kg po+Naltrexone 1 mg/kg ip | 10 | 23.8 | 2.1 | -3.9 | -14.1 | 0.162 |
|  | Bupropion 20 mg/kg ip qd+Naltrexone 1 mg/kg ip | 10 | 25.5 | 1.3 | -2.2 | -7.8 | 0.435 |
|  | Sibutramine 5 mg/kg po | 10 | 30.3 | 4.2 | 2.6 | 9.5 | 0.346 |
| Day 14 | Vehicle 4 ml/kg po +2 ml/kg ip | 12 | 30.2 | 2.0 |  |  |  |
|  | RDC5 0.6 mg/kg po | 10 | 22.1 | 1.4 | -8.1 | -26.9 | 0.005** |
|  | Bupropion 20 mg/kg ip qd | 10 | 28.2 | 1.6 | -2.0 | -6.6 | 0.480 |
|  | Naltrexone 1 mg/kg ip | 10 | 20.5 | 1.1 | -9.7 | -32.2 | <0.001*** |
|  | RDC5 0.6 mg/kg po+Bupropion 20 mg/kg ip qd | 10 | 22.7 | 2.2 | -7.5 | -24.7 | 0.010** |
|  | RDC5 0.6 mg/kg po+Naltrexone 1 mg/kg ip | 10 | 23.9 | 2.2 | -6.3 | -20.8 | 0.028* |
|  | Bupropion 20 mg/kg ip qd+Naltrexone 1 mg/kg ip | 10 | 27.2 | 3.9 | -3.0 | -10.0 | 0.285 |
|  | Sibutramine 5 mg/kg po | 10 | 31.9 | 3.4 | 1.7 | 5.7 | 0.545 |

Means are adjusted for differences between the treatment groups at baseline (average of Days -6 to 0). SEM are calculated from the residuals of the statistical model. Multiple comparisons against vehicle are by the multiple t test. *p<0.05, **p<0.01,

***p<0.001.

Treatment n Mean SEM Difference % change p

from vehicle

| Day 15 | Vehicle 4 ml/kg po +2 ml/kg ip | 12 | 26.2 | 2.0 |  |  |  |
| --- | --- | --- | --- | --- | --- | --- | --- |
|  | RDC5 0.6 mg/kg po | 10 | 23.9 | 2.4 | -2.2 | -8.5 | 0.414 |
|  | Bupropion 20 mg/kg ip qd | 10 | 27.0 | 2.4 | 0.8 | 3.2 | 0.763 |
|  | Naltrexone 1 mg/kg ip | 10 | 22.6 | 1.7 | -3.6 | -13.7 | 0.190 |
|  | RDC5 0.6 mg/kg po+Bupropion 20 mg/kg ip qd | 10 | 20.8 | 3.1 | -5.3 | -20.3 | 0.054 |
|  | RDC5 0.6 mg/kg po+Naltrexone 1 mg/kg ip | 10 | 21.9 | 1.7 | -4.2 | -16.2 | 0.123 |
|  | Bupropion 20 mg/kg ip qd+Naltrexone 1 mg/kg ip | 10 | 23.6 | 0.7 | -2.5 | -9.6 | 0.360 |
|  | Sibutramine 5 mg/kg po | 10 | 30.4 | 4.2 | 4.3 | 16.3 | 0.121 |
| Day 16 | Vehicle 4 ml/kg po +2 ml/kg ip | 12 | 24.1 | 1.3 |  |  |  |
|  | RDC5 0.6 mg/kg po | 10 | 22.5 | 1.9 | -1.6 | -6.6 | 0.535 |
|  | Bupropion 20 mg/kg ip qd | 10 | 28.2 | 1.8 | 4.1 | 16.9 | 0.113 |
|  | Naltrexone 1 mg/kg ip | 10 | 21.6 | 1.8 | -2.5 | -10.3 | 0.333 |
|  | RDC5 0.6 mg/kg po+Bupropion 20 mg/kg ip qd | 10 | 21.5 | 2.0 | -2.6 | -10.9 | 0.306 |
|  | RDC5 0.6 mg/kg po+Naltrexone 1 mg/kg ip | 10 | 23.7 | 2.5 | -0.3 | -1.4 | 0.897 |
|  | Bupropion 20 mg/kg ip qd+Naltrexone 1 mg/kg ip | 10 | 24.0 | 1.0 | -0.1 | -0.4 | 0.970 |
|  | Sibutramine 5 mg/kg po | 10 | 27.6 | 4.8 | 3.5 | 14.5 | 0.173 |
| Day 17 | Vehicle 4 ml/kg po +2 ml/kg ip | 12 | 25.0 | 1.8 |  |  |  |
|  | RDC5 0.6 mg/kg po | 10 | 20.1 | 2.0 | -4.9 | -19.5 | 0.104 |
|  | Bupropion 20 mg/kg ip qd | 10 | 29.8 | 2.1 | 4.8 | 19.1 | 0.111 |
|  | Naltrexone 1 mg/kg ip | 10 | 23.2 | 1.6 | -1.8 | -7.2 | 0.547 |
|  | RDC5 0.6 mg/kg po+Bupropion 20 mg/kg ip qd | 10 | 24.0 | 1.1 | -1.0 | -3.9 | 0.746 |
|  | RDC5 0.6 mg/kg po+Naltrexone 1 mg/kg ip | 10 | 25.2 | 3.1 | 0.2 | 0.7 | 0.956 |
|  | Bupropion 20 mg/kg ip qd+Naltrexone 1 mg/kg ip | 9 | 26.5 | 1.1 | 1.5 | 6.1 | 0.622 |
|  | Sibutramine 5 mg/kg po | 10 | 31.7 | 5.3 | 6.7 | 26.9 | 0.027* |
| Day 18 | Vehicle 4 ml/kg po +2 ml/kg ip | 12 | 27.6 | 1.4 |  |  |  |
|  | RDC5 0.6 mg/kg po | 10 | 23.4 | 2.1 | -4.2 | -15.2 | 0.207 |
|  | Bupropion 20 mg/kg ip qd | 10 | 30.9 | 2.0 | 3.4 | 12.2 | 0.310 |
|  | Naltrexone 1 mg/kg ip | 10 | 23.6 | 1.7 | -4.0 | -14.5 | 0.229 |
|  | RDC5 0.6 mg/kg po+Bupropion 20 mg/kg ip qd | 10 | 22.5 | 2.8 | -5.0 | -18.2 | 0.132 |
|  | RDC5 0.6 mg/kg po+Naltrexone 1 mg/kg ip | 10 | 25.3 | 1.8 | -2.3 | -8.3 | 0.491 |
|  | Bupropion 20 mg/kg ip qd+Naltrexone 1 mg/kg ip | 10 | 27.4 | 5.9 | -0.2 | -0.7 | 0.953 |
|  | Sibutramine 5 mg/kg po | 10 | 30.0 | 3.6 | 2.4 | 8.9 | 0.461 |
| Day 19 | Vehicle 4 ml/kg po +2 ml/kg ip | 12 | 25.0 | 1.5 |  |  |  |
|  | RDC5 0.6 mg/kg po | 10 | 26.0 | 2.7 | 1.0 | 4.0 | 0.713 |
|  | Bupropion 20 mg/kg ip qd | 10 | 29.5 | 1.5 | 4.5 | 18.0 | 0.101 |
|  | Naltrexone 1 mg/kg ip | 10 | 24.0 | 1.5 | -1.1 | -4.3 | 0.695 |
|  | RDC5 0.6 mg/kg po+Bupropion 20 mg/kg ip qd | 10 | 22.5 | 2.1 | -2.5 | -10.1 | 0.356 |
|  | RDC5 0.6 mg/kg po+Naltrexone 1 mg/kg ip | 10 | 23.9 | 2.6 | -1.1 | -4.5 | 0.682 |
|  | Bupropion 20 mg/kg ip qd+Naltrexone 1 mg/kg ip | 10 | 25.3 | 3.5 | 0.2 | 1.0 | 0.930 |
|  | Sibutramine 5 mg/kg po | 10 | 29.4 | 5.9 | 4.3 | 17.3 | 0.115 |
| Day 20 | Vehicle 4 ml/kg po +2 ml/kg ip | 12 | 26.2 | 1.0 |  |  |  |
|  | RDC5 0.6 mg/kg po | 10 | 25.9 | 1.8 | -0.3 | -1.0 | 0.905 |
|  | Bupropion 20 mg/kg ip qd | 10 | 29.6 | 1.0 | 3.5 | 13.2 | 0.117 |
|  | Naltrexone 1 mg/kg ip | 10 | 25.7 | 1.7 | -0.5 | -1.7 | 0.834 |
|  | RDC5 0.6 mg/kg po+Bupropion 20 mg/kg ip qd | 10 | 23.5 | 2.5 | -2.7 | -10.3 | 0.220 |
|  | RDC5 0.6 mg/kg po+Naltrexone 1 mg/kg ip | 10 | 26.1 | 2.3 | -0.1 | -0.4 | 0.960 |
|  | Bupropion 20 mg/kg ip qd+Naltrexone 1 mg/kg ip | 10 | 25.6 | 2.1 | -0.6 | -2.2 | 0.789 |
|  | Sibutramine 5 mg/kg po | 10 | 33.2 | 4.8 | 7.0 | 26.8 | 0.002** |
| Day 21 | Vehicle 4 ml/kg po +2 ml/kg ip | 12 | 26.5 | 1.8 |  |  |  |
|  | RDC5 0.6 mg/kg po | 10 | 26.2 | 2.6 | -0.3 | -1.2 | 0.926 |
|  | Bupropion 20 mg/kg ip qd | 10 | 28.6 | 2.2 | 2.1 | 8.0 | 0.533 |
|  | Naltrexone 1 mg/kg ip | 10 | 26.8 | 1.2 | 0.3 | 1.1 | 0.931 |
|  | RDC5 0.6 mg/kg po+Bupropion 20 mg/kg ip qd | 10 | 22.8 | 3.3 | -3.7 | -13.8 | 0.281 |
|  | RDC5 0.6 mg/kg po+Naltrexone 1 mg/kg ip | 10 | 26.8 | 3.1 | 0.3 | 1.0 | 0.937 |
|  | Bupropion 20 mg/kg ip qd+Naltrexone 1 mg/kg ip | 10 | 25.2 | 0.9 | -1.3 | -4.8 | 0.706 |
|  | Sibutramine 5 mg/kg po | 10 | 35.9 | 6.3 | 9.3 | 35.3 | 0.007** |

Means are adjusted for differences between the treatment groups at baseline (average of Days -6 to 0). SEM are calculated from the residuals of the statistical model. Multiple comparisons against vehicle are by the multiple t test. *p<0.05, **p<0.01.

Treatment n Mean SEM Difference % change p

from vehicle

| Day 22 | Vehicle 4 ml/kg po +2 ml/kg ip | 12 | 27.3 | 1.8 |  |  |  |
| --- | --- | --- | --- | --- | --- | --- | --- |
|  | RDC5 0.6 mg/kg po | 10 | 24.5 | 2.9 | -2.8 | -10.3 | 0.351 |
|  | Bupropion 20 mg/kg ip qd | 10 | 28.1 | 1.7 | 0.8 | 3.1 | 0.780 |
|  | Naltrexone 1 mg/kg ip | 10 | 24.6 | 2.7 | -2.7 | -9.9 | 0.369 |
|  | RDC5 0.6 mg/kg po+Bupropion 20 mg/kg ip qd | 10 | 24.4 | 2.2 | -2.8 | -10.4 | 0.344 |
|  | RDC5 0.6 mg/kg po+Naltrexone 1 mg/kg ip | 10 | 24.5 | 3.0 | -2.7 | -10.1 | 0.362 |
|  | Bupropion 20 mg/kg ip qd+Naltrexone 1 mg/kg ip | 10 | 24.9 | 1.3 | -2.4 | -8.8 | 0.426 |
|  | Sibutramine 5 mg/kg po | 10 | 33.0 | 3.4 | 5.8 | 21.2 | 0.057 |
| Day 23 | Vehicle 4 ml/kg po +2 ml/kg ip | 12 | 28.0 | 1.3 |  |  |  |
|  | RDC5 0.6 mg/kg po | 9 | 23.8 | 2.1 | -4.3 | -15.3 | 0.139 |
|  | Bupropion 20 mg/kg ip qd | 10 | 30.0 | 1.8 | 2.0 | 7.0 | 0.479 |
|  | Naltrexone 1 mg/kg ip | 10 | 25.6 | 1.3 | -2.4 | -8.6 | 0.389 |
|  | RDC5 0.6 mg/kg po+Bupropion 20 mg/kg ip qd | 10 | 24.8 | 2.0 | -3.2 | -11.4 | 0.252 |
|  | RDC5 0.6 mg/kg po+Naltrexone 1 mg/kg ip | 10 | 26.6 | 2.1 | -1.4 | -5.2 | 0.604 |
|  | Bupropion 20 mg/kg ip qd+Naltrexone 1 mg/kg ip | 10 | 27.4 | 1.6 | -0.6 | -2.3 | 0.818 |
|  | Sibutramine 5 mg/kg po | 10 | 33.3 | 3.4 | 5.3 | 18.8 | 0.062 |
| Day 24 | Vehicle 4 ml/kg po +2 ml/kg ip | 12 | 28.8 | 2.4 |  |  |  |
|  | RDC5 0.6 mg/kg po | 10 | 24.6 | 1.0 | -4.3 | -14.8 | 0.133 |
|  | Bupropion 20 mg/kg ip qd | 10 | 29.7 | 2.4 | 0.9 | 3.0 | 0.760 |
|  | Naltrexone 1 mg/kg ip | 10 | 22.9 | 1.6 | -5.9 | -20.6 | 0.038* |
|  | RDC5 0.6 mg/kg po+Bupropion 20 mg/kg ip qd | 10 | 25.1 | 2.4 | -3.7 | -12.8 | 0.193 |
|  | RDC5 0.6 mg/kg po+Naltrexone 1 mg/kg ip | 10 | 25.6 | 2.0 | -3.2 | -11.2 | 0.254 |
|  | Bupropion 20 mg/kg ip qd+Naltrexone 1 mg/kg ip | 10 | 26.4 | 1.9 | -2.5 | -8.5 | 0.385 |
|  | Sibutramine 5 mg/kg po | 10 | 34.9 | 4.4 | 6.1 | 21.1 | 0.034* |
| Day 25 | Vehicle 4 ml/kg po +2 ml/kg ip | 11 | 28.6 | 2.6 |  |  |  |
|  | RDC5 0.6 mg/kg po | 10 | 24.6 | 1.8 | -4.0 | -13.9 | 0.248 |
|  | Bupropion 20 mg/kg ip qd | 10 | 28.1 | 2.2 | -0.5 | -1.7 | 0.889 |
|  | Naltrexone 1 mg/kg ip | 10 | 24.6 | 1.5 | -3.9 | -13.8 | 0.250 |
|  | RDC5 0.6 mg/kg po+Bupropion 20 mg/kg ip qd | 10 | 24.0 | 2.1 | -4.6 | -16.1 | 0.181 |
|  | RDC5 0.6 mg/kg po+Naltrexone 1 mg/kg ip | 10 | 27.1 | 2.3 | -1.5 | -5.3 | 0.659 |
|  | Bupropion 20 mg/kg ip qd+Naltrexone 1 mg/kg ip | 10 | 26.5 | 1.7 | -2.1 | -7.2 | 0.547 |
|  | Sibutramine 5 mg/kg po | 10 | 35.9 | 4.7 | 7.3 | 25.7 | 0.035* |
| Day 26 | Vehicle 4 ml/kg po +2 ml/kg ip | 11 | 28.3 | 1.8 |  |  |  |
|  | RDC5 0.6 mg/kg po | 9 | 24.0 | 1.5 | -4.3 | -15.1 | 0.181 |
|  | Bupropion 20 mg/kg ip bid | 10 | 29.5 | 2.2 | 1.2 | 4.4 | 0.689 |
|  | Naltrexone 1 mg/kg ip | 10 | 24.7 | 1.9 | -3.6 | -12.7 | 0.245 |
|  | RDC5 0.6 mg/kg po+Bupropion 20 mg/kg ip bid | 10 | 25.8 | 2.4 | -2.5 | -8.8 | 0.419 |
|  | RDC5 0.6 mg/kg po+Naltrexone 1 mg/kg ip | 10 | 27.0 | 4.1 | -1.2 | -4.3 | 0.691 |
|  | Bupropion 20 mg/kg ip bid+Naltrexone 1 mg/kg ip | 10 | 26.8 | 1.4 | -1.4 | -5.1 | 0.641 |
|  | Sibutramine 5 mg/kg po | 10 | 31.0 | 4.3 | 2.7 | 9.6 | 0.378 |
| Day 27 | Vehicle 4 ml/kg po +2 ml/kg ip | 11 | 28.4 | 1.8 |  |  |  |
|  | RDC5 0.6 mg/kg po | 10 | 20.8 | 0.8 | -7.6 | -26.8 | 0.007** |
|  | Bupropion 20 mg/kg ip bid | 10 | 28.1 | 1.5 | -0.4 | -1.3 | 0.892 |
|  | Naltrexone 1 mg/kg ip | 10 | 23.9 | 1.9 | -4.6 | -16.0 | 0.099 |
|  | RDC5 0.6 mg/kg po+Bupropion 20 mg/kg ip bid | 10 | 23.6 | 1.3 | -4.8 | -16.9 | 0.082 |
|  | RDC5 0.6 mg/kg po+Naltrexone 1 mg/kg ip | 10 | 25.7 | 2.2 | -2.8 | -9.8 | 0.310 |
|  | Bupropion 20 mg/kg ip bid+Naltrexone 1 mg/kg ip | 10 | 26.0 | 2.5 | -2.5 | -8.7 | 0.366 |
|  | Sibutramine 5 mg/kg po | 10 | 30.0 | 8.5 | 1.6 | 5.5 | 0.566 |
| Day 28 | Vehicle 4 ml/kg po +2 ml/kg ip | 12 | 28.4 | 1.5 |  |  |  |
|  | RDC5 0.6 mg/kg po | 10 | 27.7 | 1.4 | -0.7 | -2.4 | 0.778 |
|  | Bupropion 20 mg/kg ip bid | 10 | 31.4 | 2.7 | 3.1 | 10.9 | 0.200 |
|  | Naltrexone 1 mg/kg ip | 10 | 24.0 | 1.2 | -4.4 | -15.4 | 0.071 |
|  | RDC5 0.6 mg/kg po+Bupropion 20 mg/kg ip bid | 10 | 27.1 | 1.7 | -1.3 | -4.5 | 0.592 |
|  | RDC5 0.6 mg/kg po+Naltrexone 1 mg/kg ip | 10 | 28.8 | 2.2 | 0.4 | 1.5 | 0.855 |
|  | Bupropion 20 mg/kg ip bid+Naltrexone 1 mg/kg ip | 10 | 28.6 | 1.5 | 0.2 | 0.8 | 0.925 |
|  | Sibutramine 5 mg/kg po | 10 | 31.7 | 6.7 | 3.3 | 11.6 | 0.171 |

Means are adjusted for differences between the treatment groups at baseline (average of Days -6 to 0). SEM are calculated from the residuals of the statistical model. Multiple comparisons against vehicle are by the multiple t test. *p<0.05, **p<0.01.

Treatment n Mean SEM Difference % change p

from vehicle

| Day 29 | Vehicle 4 ml/kg po +2 ml/kg ip | 12 | 29.3 | 1.8 |  |  |  |
| --- | --- | --- | --- | --- | --- | --- | --- |
|  | RDC5 0.6 mg/kg po | 10 | 26.1 | 1.7 | -3.2 | -11.0 | 0.188 |
|  | Bupropion 20 mg/kg ip bid | 10 | 29.2 | 1.2 | -0.1 | -0.5 | 0.957 |
|  | Naltrexone 1 mg/kg ip | 10 | 22.1 | 1.7 | -7.2 | -24.6 | 0.004** |
|  | RDC5 0.6 mg/kg po+Bupropion 20 mg/kg ip bid | 10 | 24.9 | 1.4 | -4.4 | -15.0 | 0.073 |
|  | RDC5 0.6 mg/kg po+Naltrexone 1 mg/kg ip | 10 | 25.6 | 1.9 | -3.7 | -12.7 | 0.129 |
|  | Bupropion 20 mg/kg ip bid+Naltrexone 1 mg/kg ip | 10 | 27.2 | 1.6 | -2.0 | -7.0 | 0.403 |
|  | Sibutramine 5 mg/kg po | 10 | 33.6 | 4.7 | 4.3 | 14.8 | 0.078 |
| Day 30 | Vehicle 4 ml/kg po +2 ml/kg ip | 12 | 26.7 | 1.5 |  |  |  |
|  | RDC5 0.6 mg/kg po | 10 | 24.6 | 1.2 | -2.1 | -8.0 | 0.402 |
|  | Bupropion 20 mg/kg ip bid | 10 | 29.9 | 1.4 | 3.2 | 12.0 | 0.208 |
|  | Naltrexone 1 mg/kg ip | 10 | 22.4 | 1.5 | -4.3 | -16.1 | 0.092 |
|  | RDC5 0.6 mg/kg po+Bupropion 20 mg/kg ip bid | 10 | 23.8 | 1.9 | -2.9 | -10.8 | 0.256 |
|  | RDC5 0.6 mg/kg po+Naltrexone 1 mg/kg ip | 10 | 28.6 | 2.2 | 1.9 | 7.2 | 0.452 |
|  | Bupropion 20 mg/kg ip bid+Naltrexone 1 mg/kg ip | 10 | 27.4 | 1.8 | 0.7 | 2.8 | 0.772 |
|  | Sibutramine 5 mg/kg po | 10 | 34.5 | 3.2 | 7.8 | 29.1 | 0.003** |
| Day 31 | Vehicle 4 ml/kg po +2 ml/kg ip | 12 | 29.6 | 1.8 |  |  |  |
|  | RDC5 0.6 mg/kg po | 10 | 24.2 | 2.0 | -5.3 | -18.1 | 0.057 |
|  | Bupropion 20 mg/kg ip bid | 10 | 28.1 | 1.7 | -1.5 | -5.0 | 0.595 |
|  | Naltrexone 1 mg/kg ip | 10 | 22.9 | 0.9 | -6.7 | -22.6 | 0.018* |
|  | RDC5 0.6 mg/kg po+Bupropion 20 mg/kg ip bid | 10 | 25.6 | 2.1 | -3.9 | -13.2 | 0.162 |
|  | RDC5 0.6 mg/kg po+Naltrexone 1 mg/kg ip | 10 | 25.5 | 2.2 | -4.0 | -13.6 | 0.153 |
|  | Bupropion 20 mg/kg ip bid+Naltrexone 1 mg/kg ip | 10 | 27.6 | 1.4 | -2.0 | -6.6 | 0.482 |
|  | Sibutramine 5 mg/kg po | 10 | 32.9 | 3.5 | 3.3 | 11.2 | 0.236 |
| Day 32 | Vehicle 4 ml/kg po +2 ml/kg ip | 12 | 27.4 | 2.0 |  |  |  |
|  | RDC5 0.6 mg/kg po | 10 | 24.0 | 3.4 | -3.4 | -12.5 | 0.212 |
|  | Bupropion 20 mg/kg ip bid | 10 | 25.5 | 2.2 | -1.9 | -6.9 | 0.490 |
|  | Naltrexone 1 mg/kg ip | 10 | 23.6 | 1.3 | -3.8 | -13.7 | 0.171 |
|  | RDC5 0.6 mg/kg po+Bupropion 20 mg/kg ip bid | 10 | 23.8 | 2.2 | -3.6 | -13.0 | 0.196 |
|  | RDC5 0.6 mg/kg po+Naltrexone 1 mg/kg ip | 10 | 27.3 | 1.9 | -0.1 | -0.3 | 0.980 |
|  | Bupropion 20 mg/kg ip bid+Naltrexone 1 mg/kg ip | 10 | 25.2 | 4.3 | -2.2 | -7.9 | 0.427 |
|  | Sibutramine 5 mg/kg po | 10 | 33.0 | 4.4 | 5.6 | 20.4 | 0.043* |
| Day 33 | Vehicle 4 ml/kg po +2 ml/kg ip | 12 | 27.0 | 2.7 |  |  |  |
|  | RDC5 0.6 mg/kg po | 10 | 24.7 | 2.5 | -2.3 | -8.5 | 0.462 |
|  | Bupropion 20 mg/kg ip bid | 10 | 25.8 | 2.4 | -1.2 | -4.4 | 0.702 |
|  | Naltrexone 1 mg/kg ip | 10 | 23.1 | 1.6 | -3.8 | -14.2 | 0.222 |
|  | RDC5 0.6 mg/kg po+Bupropion 20 mg/kg ip bid | 10 | 25.4 | 2.2 | -1.6 | -5.8 | 0.616 |
|  | RDC5 0.6 mg/kg po+Naltrexone 1 mg/kg ip | 10 | 26.4 | 4.2 | -0.6 | -2.2 | 0.848 |
|  | Bupropion 20 mg/kg ip bid+Naltrexone 1 mg/kg ip | 10 | 22.4 | 5.4 | -4.6 | -17.0 | 0.145 |
|  | Sibutramine 5 mg/kg po | 10 | 30.1 | 6.0 | 3.2 | 11.7 | 0.314 |
| Day 34 | Vehicle 4 ml/kg po +2 ml/kg ip | 12 | 27.1 | 2.6 |  |  |  |
|  | RDC5 0.6 mg/kg po | 10 | 26.8 | 3.1 | -0.3 | -1.3 | 0.916 |
|  | Bupropion 20 mg/kg ip bid | 10 | 26.7 | 1.4 | -0.4 | -1.6 | 0.893 |
|  | Naltrexone 1 mg/kg ip | 10 | 22.7 | 1.6 | -4.4 | -16.2 | 0.176 |
|  | RDC5 0.6 mg/kg po+Bupropion 20 mg/kg ip bid | 10 | 24.1 | 2.4 | -3.0 | -11.2 | 0.346 |
|  | RDC5 0.6 mg/kg po+Naltrexone 1 mg/kg ip | 10 | 27.5 | 4.7 | 0.4 | 1.3 | 0.913 |
|  | Bupropion 20 mg/kg ip bid+Naltrexone 1 mg/kg ip | 10 | 23.7 | 3.5 | -3.4 | -12.6 | 0.290 |
|  | Sibutramine 5 mg/kg po | 10 | 31.9 | 3.1 | 4.8 | 17.6 | 0.141 |
| Day 35 | Vehicle 4 ml/kg po +2 ml/kg ip | 12 | 28.5 | 1.3 |  |  |  |
|  | RDC5 0.6 mg/kg po | 10 | 27.0 | 4.1 | -1.5 | -5.4 | 0.569 |
|  | Bupropion 20 mg/kg ip bid | 10 | 29.4 | 1.3 | 0.8 | 2.9 | 0.755 |
|  | Naltrexone 1 mg/kg ip | 10 | 22.6 | 2.6 | -6.0 | -20.9 | 0.029* |
|  | RDC5 0.6 mg/kg po+Bupropion 20 mg/kg ip bid | 10 | 26.7 | 1.6 | -1.8 | -6.5 | 0.492 |
|  | RDC5 0.6 mg/kg po+Naltrexone 1 mg/kg ip | 10 | 28.2 | 1.4 | -0.4 | -1.3 | 0.893 |
|  | Bupropion 20 mg/kg ip bid+Naltrexone 1 mg/kg ip | 10 | 26.2 | 4.3 | -2.3 | -8.0 | 0.396 |
|  | Sibutramine 5 mg/kg po | 10 | 33.8 | 3.4 | 5.2 | 18.3 | 0.055 |

Means are adjusted for differences between the treatment groups at baseline (average of Days -6 to 0). SEM are calculated from the residuals of the statistical model. Multiple comparisons against vehicle are by the multiple t test. *p<0.05, **p<0.01.

Treatment n Mean SEM Difference % change p

from vehicle

| Day 36 | Vehicle 4 ml/kg po +2 ml/kg ip | 12 | 29.3 | 1.6 |  |  |  |
| --- | --- | --- | --- | --- | --- | --- | --- |
|  | RDC5 0.6 mg/kg po | 10 | 23.8 | 2.5 | -5.5 | -18.8 | 0.100 |
|  | Bupropion 20 mg/kg ip bid | 10 | 25.1 | 2.4 | -4.2 | -14.4 | 0.207 |
|  | Naltrexone 1 mg/kg ip | 10 | 19.9 | 1.7 | -9.4 | -32.1 | 0.006** |
|  | RDC5 0.6 mg/kg po+Bupropion 20 mg/kg ip bid | 10 | 24.3 | 1.9 | -5.0 | -17.1 | 0.135 |
|  | RDC5 0.6 mg/kg po+Naltrexone 1 mg/kg ip | 10 | 20.1 | 1.9 | -9.2 | -31.6 | 0.007** |
|  | Bupropion 20 mg/kg ip bid+Naltrexone 1 mg/kg ip | 10 | 28.8 | 4.2 | -0.6 | -1.9 | 0.867 |
|  | Sibutramine 5 mg/kg po | 10 | 32.4 | 4.3 | 3.1 | 10.6 | 0.350 |
| Day 37 | Vehicle 4 ml/kg po +2 ml/kg ip | 12 | 25.5 | 1.0 |  |  |  |
|  | RDC5 0.6 mg/kg po | 10 | 28.0 | 3.3 | 2.5 | 9.8 | 0.415 |
|  | Bupropion 20 mg/kg ip bid | 10 | 28.9 | 3.0 | 3.4 | 13.3 | 0.269 |
|  | Naltrexone 1 mg/kg ip | 10 | 23.7 | 1.2 | -1.8 | -7.0 | 0.561 |
|  | RDC5 0.6 mg/kg po+Bupropion 20 mg/kg ip bid | 10 | 27.7 | 1.3 | 2.2 | 8.8 | 0.465 |
|  | RDC5 0.6 mg/kg po+Naltrexone 1 mg/kg ip | 10 | 26.4 | 1.6 | 1.0 | 3.8 | 0.750 |
|  | Bupropion 20 mg/kg ip bid+Naltrexone 1 mg/kg ip | 10 | 31.6 | 3.2 | 6.1 | 24.1 | 0.048* |
|  | Sibutramine 5 mg/kg po | 10 | 31.9 | 3.5 | 6.4 | 25.1 | 0.040* |
| Day 38 | Vehicle 4 ml/kg po +2 ml/kg ip | 12 | 25.9 | 1.2 |  |  |  |
|  | RDC5 0.6 mg/kg po | 10 | 26.0 | 2.1 | 0.0 | 0.1 | 0.990 |
|  | Bupropion 20 mg/kg ip bid | 10 | 29.8 | 2.3 | 3.9 | 15.0 | 0.166 |
|  | Naltrexone 1 mg/kg ip | 10 | 22.2 | 1.6 | -3.7 | -14.3 | 0.187 |
|  | RDC5 0.6 mg/kg po+Bupropion 20 mg/kg ip bid | 10 | 25.6 | 1.5 | -0.4 | -1.5 | 0.892 |
|  | RDC5 0.6 mg/kg po+Naltrexone 1 mg/kg ip | 10 | 27.2 | 4.6 | 1.2 | 4.8 | 0.658 |
|  | Bupropion 20 mg/kg ip bid+Naltrexone 1 mg/kg ip | 10 | 28.7 | 5.5 | 2.7 | 10.6 | 0.327 |
|  | Sibutramine 5 mg/kg po | 10 | 30.4 | 4.6 | 4.5 | 17.2 | 0.113 |
| Day 39 | Vehicle 4 ml/kg po +2 ml/kg ip | 11 | 29.5 | 2.4 |  |  |  |
|  | RDC5 0.6 mg/kg po | 10 | 24.7 | 2.8 | -4.8 | -16.4 | 0.169 |
|  | Bupropion 20 mg/kg ip bid | 10 | 25.9 | 1.7 | -3.6 | -12.1 | 0.308 |
|  | Naltrexone 1 mg/kg ip | 10 | 25.3 | 2.3 | -4.2 | -14.1 | 0.238 |
|  | RDC5 0.6 mg/kg po+Bupropion 20 mg/kg ip bid | 10 | 28.5 | 1.5 | -1.0 | -3.2 | 0.785 |
|  | RDC5 0.6 mg/kg po+Naltrexone 1 mg/kg ip | 10 | 26.6 | 5.7 | -2.9 | -9.9 | 0.405 |
|  | Bupropion 20 mg/kg ip bid+Naltrexone 1 mg/kg ip | 10 | 27.5 | 3.1 | -2.0 | -6.7 | 0.574 |
|  | Sibutramine 5 mg/kg po | 10 | 28.9 | 5.7 | -0.6 | -2.2 | 0.856 |
| Day 40 | Vehicle 4 ml/kg po +2 ml/kg ip | 11 | 27.3 | 2.6 |  |  |  |
|  | RDC5 0.6 mg/kg po | 10 | 27.6 | 2.9 | 0.3 | 1.1 | 0.913 |
|  | Bupropion 20 mg/kg ip bid | 10 | 25.6 | 2.0 | -1.8 | -6.4 | 0.537 |
|  | Naltrexone 1 mg/kg ip | 10 | 25.8 | 2.1 | -1.6 | -5.7 | 0.582 |
|  | RDC5 0.6 mg/kg po+Bupropion 20 mg/kg ip bid | 10 | 27.5 | 1.3 | 0.1 | 0.5 | 0.961 |
|  | RDC5 0.6 mg/kg po+Naltrexone 1 mg/kg ip | 10 | 25.4 | 1.9 | -1.9 | -6.9 | 0.505 |
|  | Bupropion 20 mg/kg ip bid+Naltrexone 1 mg/kg ip | 10 | 28.2 | 1.4 | 0.9 | 3.3 | 0.751 |
|  | Sibutramine 5 mg/kg po | 10 | 32.0 | 3.8 | 4.6 | 16.9 | 0.106 |
| Day 41 | Vehicle 4 ml/kg po +2 ml/kg ip | 12 | 25.5 | 1.8 |  |  |  |
|  | RDC5 0.6 mg/kg po | 10 | 27.2 | 2.7 | 1.7 | 6.9 | 0.585 |
|  | Bupropion 20 mg/kg ip bid | 10 | 26.8 | 1.5 | 1.3 | 5.2 | 0.677 |
|  | Naltrexone 1 mg/kg ip | 10 | 24.7 | 1.8 | -0.8 | -3.0 | 0.811 |
|  | RDC5 0.6 mg/kg po+Bupropion 20 mg/kg ip bid | 10 | 26.2 | 3.2 | 0.7 | 2.9 | 0.817 |
|  | RDC5 0.6 mg/kg po+Naltrexone 1 mg/kg ip | 10 | 28.6 | 2.5 | 3.2 | 12.4 | 0.325 |
|  | Bupropion 20 mg/kg ip bid+Naltrexone 1 mg/kg ip | 10 | 27.3 | 1.5 | 1.8 | 7.1 | 0.574 |
|  | Sibutramine 5 mg/kg po | 10 | 32.0 | 4.3 | 6.5 | 25.6 | 0.045* |
| Day 42 | Vehicle 4 ml/kg po +2 ml/kg ip | 12 | 28.6 | 1.3 |  |  |  |
|  | RDC5 0.6 mg/kg po | 10 | 27.0 | 3.4 | -1.6 | -5.7 | 0.627 |
|  | Bupropion 20 mg/kg ip bid | 10 | 29.5 | 2.2 | 0.9 | 3.0 | 0.797 |
|  | Naltrexone 1 mg/kg ip | 10 | 26.0 | 2.2 | -2.7 | -9.3 | 0.425 |
|  | RDC5 0.6 mg/kg po+Bupropion 20 mg/kg ip bid | 10 | 29.9 | 5.5 | 1.3 | 4.5 | 0.696 |
|  | RDC5 0.6 mg/kg po+Naltrexone 1 mg/kg ip | 10 | 27.6 | 2.5 | -1.0 | -3.5 | 0.760 |
|  | Bupropion 20 mg/kg ip bid+Naltrexone 1 mg/kg ip | 10 | 30.5 | 2.1 | 1.9 | 6.5 | 0.577 |
|  | Sibutramine 5 mg/kg po | 10 | 32.2 | 4.5 | 3.6 | 12.5 | 0.285 |

Means are adjusted for differences between the treatment groups at baseline (average of Days -6 to 0). SEM are calculated from the residuals of the statistical model. Multiple comparisons against vehicle are by the multiple t test. *p<0.05, **p<0.01.

| Time | Comparison | Difference | p |
| --- | --- | --- | --- |
| Day -6 | RDC5 (0.6 mg/kg po)+Bupropion (20 mg/kg ip qd) vs RDC5 | -0.4 | 0.773 |
|  | RDC5 (0.6 mg/kg po)+Naltrexone (1 mg/kg ip) vs RDC5 | -0.8 | 0.618 |
|  | RDC5 (0.6 mg/kg po)+Bupropion (20 mg/kg ip qd) vs Bupropion | 0.8 | 0.620 |
|  | Bupropion (20 mg/kg ip qd)+Naltrexone (1 mg/kg ip) vs Bupropion | 2.7 | 0.087 |
|  | RDC5 (0.6 mg/kg po)+Naltrexone (1 mg/kg ip) vs Naltrexone | -3.1 | 0.051 |
|  | Bupropion (20 mg/kg ip qd)+Naltrexone (1 mg/kg ip) vs Naltrexone | -0.8 | 0.596 |
| Day -5 | RDC5 (0.6 mg/kg po)+Bupropion (20 mg/kg ip qd) vs RDC5 | 0.4 | 0.804 |
|  | RDC5 (0.6 mg/kg po)+Naltrexone (1 mg/kg ip) vs RDC5 | 1.2 | 0.469 |
|  | RDC5 (0.6 mg/kg po)+Bupropion (20 mg/kg ip qd) vs Bupropion | 0.8 | 0.634 |
|  | Bupropion (20 mg/kg ip qd)+Naltrexone (1 mg/kg ip) vs Bupropion | 2.1 | 0.200 |
|  | RDC5 (0.6 mg/kg po)+Naltrexone (1 mg/kg ip) vs Naltrexone | 1.0 | 0.541 |
|  | Bupropion (20 mg/kg ip qd)+Naltrexone (1 mg/kg ip) vs Naltrexone | 1.5 | 0.345 |
| Day -4 | RDC5 (0.6 mg/kg po)+Bupropion (20 mg/kg ip qd) vs RDC5 | 0.4 | 0.801 |
|  | RDC5 (0.6 mg/kg po)+Naltrexone (1 mg/kg ip) vs RDC5 | 0.1 | 0.933 |
|  | RDC5 (0.6 mg/kg po)+Bupropion (20 mg/kg ip qd) vs Bupropion | -1.2 | 0.417 |
|  | Bupropion (20 mg/kg ip qd)+Naltrexone (1 mg/kg ip) vs Bupropion | -0.4 | 0.782 |
|  | RDC5 (0.6 mg/kg po)+Naltrexone (1 mg/kg ip) vs Naltrexone | 1.4 | 0.339 |
|  | Bupropion (20 mg/kg ip qd)+Naltrexone (1 mg/kg ip) vs Naltrexone | 2.4 | 0.099 |
| Day -3 | RDC5 (0.6 mg/kg po)+Bupropion (20 mg/kg ip qd) vs RDC5 | 0.3 | 0.875 |
|  | RDC5 (0.6 mg/kg po)+Naltrexone (1 mg/kg ip) vs RDC5 | -0.9 | 0.576 |
|  | RDC5 (0.6 mg/kg po)+Bupropion (20 mg/kg ip qd) vs Bupropion | 1.2 | 0.462 |
|  | Bupropion (20 mg/kg ip qd)+Naltrexone (1 mg/kg ip) vs Bupropion | -1.5 | 0.347 |
|  | RDC5 (0.6 mg/kg po)+Naltrexone (1 mg/kg ip) vs Naltrexone | -2.6 | 0.103 |
|  | Bupropion (20 mg/kg ip qd)+Naltrexone (1 mg/kg ip) vs Naltrexone | -4.2 | 0.011* |
| Day -2 | RDC5 (0.6 mg/kg po)+Bupropion (20 mg/kg ip qd) vs RDC5 | -0.3 | 0.858 |
|  | RDC5 (0.6 mg/kg po)+Naltrexone (1 mg/kg ip) vs RDC5 | -1.2 | 0.452 |
|  | RDC5 (0.6 mg/kg po)+Bupropion (20 mg/kg ip qd) vs Bupropion | 0.5 | 0.746 |
|  | Bupropion (20 mg/kg ip qd)+Naltrexone (1 mg/kg ip) vs Bupropion | -0.7 | 0.672 |
|  | RDC5 (0.6 mg/kg po)+Naltrexone (1 mg/kg ip) vs Naltrexone | -0.7 | 0.637 |
|  | Bupropion (20 mg/kg ip qd)+Naltrexone (1 mg/kg ip) vs Naltrexone | -1.0 | 0.520 |
| Day -1 | RDC5 (0.6 mg/kg po)+Bupropion (20 mg/kg ip qd) vs RDC5 | -0.3 | 0.820 |
|  | RDC5 (0.6 mg/kg po)+Naltrexone (1 mg/kg ip) vs RDC5 | 3.2 | 0.037* |
|  | RDC5 (0.6 mg/kg po)+Bupropion (20 mg/kg ip qd) vs Bupropion | -2.8 | 0.067 |
|  | Bupropion (20 mg/kg ip qd)+Naltrexone (1 mg/kg ip) vs Bupropion | -2.9 | 0.062 |
|  | RDC5 (0.6 mg/kg po)+Naltrexone (1 mg/kg ip) vs Naltrexone | 3.1 | 0.044* |
|  | Bupropion (20 mg/kg ip qd)+Naltrexone (1 mg/kg ip) vs Naltrexone | -0.5 | 0.726 |
| Day 0 | RDC5 (0.6 mg/kg po)+Bupropion (20 mg/kg ip qd) vs RDC5 | 1.6 | 0.548 |
|  | RDC5 (0.6 mg/kg po)+Naltrexone (1 mg/kg ip) vs RDC5 | 0.2 | 0.928 |
|  | RDC5 (0.6 mg/kg po)+Bupropion (20 mg/kg ip qd) vs Bupropion | -1.2 | 0.630 |
|  | Bupropion (20 mg/kg ip qd)+Naltrexone (1 mg/kg ip) vs Bupropion | -1.2 | 0.639 |
|  | RDC5 (0.6 mg/kg po)+Naltrexone (1 mg/kg ip) vs Naltrexone | -0.2 | 0.946 |
|  | Bupropion (20 mg/kg ip qd)+Naltrexone (1 mg/kg ip) vs Naltrexone | 1.2 | 0.649 |

Multiple comparisons are by the multiple t test. *p<0.05.

| Time | Comparison | Difference | p |
| --- | --- | --- | --- |
| Day 1 | RDC5 (0.6 mg/kg po)+Bupropion (20 mg/kg ip qd) vs RDC5 | 3.2 | 0.156 |
|  | RDC5 (0.6 mg/kg po)+Naltrexone (1 mg/kg ip) vs RDC5 | -1.7 | 0.433 |
|  | RDC5 (0.6 mg/kg po)+Bupropion (20 mg/kg ip qd) vs Bupropion | -2.3 | 0.290 |
|  | Bupropion (20 mg/kg ip qd)+Naltrexone (1 mg/kg ip) vs Bupropion | 1.3 | 0.561 |
|  | RDC5 (0.6 mg/kg po)+Naltrexone (1 mg/kg ip) vs Naltrexone | -4.8 | 0.032* |
|  | Bupropion (20 mg/kg ip qd)+Naltrexone (1 mg/kg ip) vs Naltrexone | 3.7 | 0.096 |
| Day 2 | RDC5 (0.6 mg/kg po)+Bupropion (20 mg/kg ip qd) vs RDC5 | 1.0 | 0.621 |
|  | RDC5 (0.6 mg/kg po)+Naltrexone (1 mg/kg ip) vs RDC5 | -0.7 | 0.758 |
|  | RDC5 (0.6 mg/kg po)+Bupropion (20 mg/kg ip qd) vs Bupropion | -8.2 | <0.001*** |
|  | Bupropion (20 mg/kg ip qd)+Naltrexone (1 mg/kg ip) vs Bupropion | -2.6 | 0.229 |
|  | RDC5 (0.6 mg/kg po)+Naltrexone (1 mg/kg ip) vs Naltrexone | -4.7 | 0.027* |
|  | Bupropion (20 mg/kg ip qd)+Naltrexone (1 mg/kg ip) vs Naltrexone | 2.6 | 0.225 |
| Day 3 | RDC5 (0.6 mg/kg po)+Bupropion (20 mg/kg ip qd) vs RDC5 | 1.7 | 0.472 |
|  | RDC5 (0.6 mg/kg po)+Naltrexone (1 mg/kg ip) vs RDC5 | 1.6 | 0.493 |
|  | RDC5 (0.6 mg/kg po)+Bupropion (20 mg/kg ip qd) vs Bupropion | -4.4 | 0.064 |
|  | Bupropion (20 mg/kg ip qd)+Naltrexone (1 mg/kg ip) vs Bupropion | 0.2 | 0.923 |
|  | RDC5 (0.6 mg/kg po)+Naltrexone (1 mg/kg ip) vs Naltrexone | -1.4 | 0.550 |
|  | Bupropion (20 mg/kg ip qd)+Naltrexone (1 mg/kg ip) vs Naltrexone | 3.3 | 0.163 |
| Day 4 | RDC5 (0.6 mg/kg po)+Bupropion (20 mg/kg ip qd) vs RDC5 | 1.9 | 0.437 |
|  | RDC5 (0.6 mg/kg po)+Naltrexone (1 mg/kg ip) vs RDC5 | 2.5 | 0.303 |
|  | RDC5 (0.6 mg/kg po)+Bupropion (20 mg/kg ip qd) vs Bupropion | -3.4 | 0.154 |
|  | Bupropion (20 mg/kg ip qd)+Naltrexone (1 mg/kg ip) vs Bupropion | -2.7 | 0.270 |
|  | RDC5 (0.6 mg/kg po)+Naltrexone (1 mg/kg ip) vs Naltrexone | 2.2 | 0.360 |
|  | Bupropion (20 mg/kg ip qd)+Naltrexone (1 mg/kg ip) vs Naltrexone | 2.4 | 0.325 |
| Day 5 | RDC5 (0.6 mg/kg po)+Bupropion (20 mg/kg ip qd) vs RDC5 | 2.4 | 0.236 |
|  | RDC5 (0.6 mg/kg po)+Naltrexone (1 mg/kg ip) vs RDC5 | 1.2 | 0.536 |
|  | RDC5 (0.6 mg/kg po)+Bupropion (20 mg/kg ip qd) vs Bupropion | -6.0 | 0.003** |
|  | Bupropion (20 mg/kg ip qd)+Naltrexone (1 mg/kg ip) vs Bupropion | -5.6 | 0.006** |
|  | RDC5 (0.6 mg/kg po)+Naltrexone (1 mg/kg ip) vs Naltrexone | -0.0 | 0.993 |
|  | Bupropion (20 mg/kg ip qd)+Naltrexone (1 mg/kg ip) vs Naltrexone | 1.6 | 0.437 |
| Day 6 | RDC5 (0.6 mg/kg po)+Bupropion (20 mg/kg ip qd) vs RDC5 | 1.8 | 0.486 |
|  | RDC5 (0.6 mg/kg po)+Naltrexone (1 mg/kg ip) vs RDC5 | 2.6 | 0.303 |
|  | RDC5 (0.6 mg/kg po)+Bupropion (20 mg/kg ip qd) vs Bupropion | -3.8 | 0.143 |
|  | Bupropion (20 mg/kg ip qd)+Naltrexone (1 mg/kg ip) vs Bupropion | -2.4 | 0.339 |
|  | RDC5 (0.6 mg/kg po)+Naltrexone (1 mg/kg ip) vs Naltrexone | -0.4 | 0.862 |
|  | Bupropion (20 mg/kg ip qd)+Naltrexone (1 mg/kg ip) vs Naltrexone | 0.0 | 0.993 |
| Day 7 | RDC5 (0.6 mg/kg po)+Bupropion (20 mg/kg ip qd) vs RDC5 | 1.5 | 0.506 |
|  | RDC5 (0.6 mg/kg po)+Naltrexone (1 mg/kg ip) vs RDC5 | 0.0 | 0.991 |
|  | RDC5 (0.6 mg/kg po)+Bupropion (20 mg/kg ip qd) vs Bupropion | -6.8 | 0.004** |
|  | Bupropion (20 mg/kg ip qd)+Naltrexone (1 mg/kg ip) vs Bupropion | -6.3 | 0.007** |
|  | RDC5 (0.6 mg/kg po)+Naltrexone (1 mg/kg ip) vs Naltrexone | -1.9 | 0.399 |
|  | Bupropion (20 mg/kg ip qd)+Naltrexone (1 mg/kg ip) vs Naltrexone | 0.1 | 0.981 |

Multiple comparisons are by the multiple t test. *p<0.05, **p<0.01, ***p<0.001.

| Time | Comparison | Difference | p |
| --- | --- | --- | --- |
| Day 8 | RDC5 (0.6 mg/kg po)+Bupropion (20 mg/kg ip qd) vs RDC5 | -2.1 | 0.416 |
|  | RDC5 (0.6 mg/kg po)+Naltrexone (1 mg/kg ip) vs RDC5 | 1.1 | 0.680 |
|  | RDC5 (0.6 mg/kg po)+Bupropion (20 mg/kg ip qd) vs Bupropion | -5.8 | 0.029* |
|  | Bupropion (20 mg/kg ip qd)+Naltrexone (1 mg/kg ip) vs Bupropion | -0.1 | 0.968 |
|  | RDC5 (0.6 mg/kg po)+Naltrexone (1 mg/kg ip) vs Naltrexone | -1.5 | 0.560 |
|  | Bupropion (20 mg/kg ip qd)+Naltrexone (1 mg/kg ip) vs Naltrexone | 1.0 | 0.713 |
| Day 9 | RDC5 (0.6 mg/kg po)+Bupropion (20 mg/kg ip qd) vs RDC5 | 0.3 | 0.882 |
|  | RDC5 (0.6 mg/kg po)+Naltrexone (1 mg/kg ip) vs RDC5 | 2.6 | 0.229 |
|  | RDC5 (0.6 mg/kg po)+Bupropion (20 mg/kg ip qd) vs Bupropion | -6.2 | 0.004** |
|  | Bupropion (20 mg/kg ip qd)+Naltrexone (1 mg/kg ip) vs Bupropion | -1.3 | 0.555 |
|  | RDC5 (0.6 mg/kg po)+Naltrexone (1 mg/kg ip) vs Naltrexone | 1.0 | 0.651 |
|  | Bupropion (20 mg/kg ip qd)+Naltrexone (1 mg/kg ip) vs Naltrexone | 3.7 | 0.086 |
| Day 10 | RDC5 (0.6 mg/kg po)+Bupropion (20 mg/kg ip qd) vs RDC5 | -0.5 | 0.825 |
|  | RDC5 (0.6 mg/kg po)+Naltrexone (1 mg/kg ip) vs RDC5 | -0.6 | 0.798 |
|  | RDC5 (0.6 mg/kg po)+Bupropion (20 mg/kg ip qd) vs Bupropion | -4.1 | 0.086 |
|  | Bupropion (20 mg/kg ip qd)+Naltrexone (1 mg/kg ip) vs Bupropion | -2.9 | 0.215 |
|  | RDC5 (0.6 mg/kg po)+Naltrexone (1 mg/kg ip) vs Naltrexone | 0.9 | 0.718 |
|  | Bupropion (20 mg/kg ip qd)+Naltrexone (1 mg/kg ip) vs Naltrexone | 2.1 | 0.378 |
| Day 11 | RDC5 (0.6 mg/kg po)+Bupropion (20 mg/kg ip qd) vs RDC5 | -4.4 | 0.074 |
|  | RDC5 (0.6 mg/kg po)+Naltrexone (1 mg/kg ip) vs RDC5 | -3.2 | 0.199 |
|  | RDC5 (0.6 mg/kg po)+Bupropion (20 mg/kg ip qd) vs Bupropion | -7.7 | 0.002** |
|  | Bupropion (20 mg/kg ip qd)+Naltrexone (1 mg/kg ip) vs Bupropion | -3.3 | 0.186 |
|  | RDC5 (0.6 mg/kg po)+Naltrexone (1 mg/kg ip) vs Naltrexone | 0.2 | 0.919 |
|  | Bupropion (20 mg/kg ip qd)+Naltrexone (1 mg/kg ip) vs Naltrexone | 3.4 | 0.168 |
| Day 12 | RDC5 (0.6 mg/kg po)+Bupropion (20 mg/kg ip qd) vs RDC5 | -0.2 | 0.932 |
|  | RDC5 (0.6 mg/kg po)+Naltrexone (1 mg/kg ip) vs RDC5 | 0.6 | 0.823 |
|  | RDC5 (0.6 mg/kg po)+Bupropion (20 mg/kg ip qd) vs Bupropion | -4.9 | 0.077 |
|  | Bupropion (20 mg/kg ip qd)+Naltrexone (1 mg/kg ip) vs Bupropion | -1.5 | 0.575 |
|  | RDC5 (0.6 mg/kg po)+Naltrexone (1 mg/kg ip) vs Naltrexone | 2.5 | 0.366 |
|  | Bupropion (20 mg/kg ip qd)+Naltrexone (1 mg/kg ip) vs Naltrexone | 5.0 | 0.071 |
| Day 13 | RDC5 (0.6 mg/kg po)+Bupropion (20 mg/kg ip qd) vs RDC5 | 2.7 | 0.360 |
|  | RDC5 (0.6 mg/kg po)+Naltrexone (1 mg/kg ip) vs RDC5 | 1.9 | 0.524 |
|  | RDC5 (0.6 mg/kg po)+Bupropion (20 mg/kg ip qd) vs Bupropion | -3.4 | 0.241 |
|  | Bupropion (20 mg/kg ip qd)+Naltrexone (1 mg/kg ip) vs Bupropion | -2.5 | 0.393 |
|  | RDC5 (0.6 mg/kg po)+Naltrexone (1 mg/kg ip) vs Naltrexone | 1.2 | 0.671 |
|  | Bupropion (20 mg/kg ip qd)+Naltrexone (1 mg/kg ip) vs Naltrexone | 3.0 | 0.306 |
| Day 14 | RDC5 (0.6 mg/kg po)+Bupropion (20 mg/kg ip qd) vs RDC5 | 0.6 | 0.828 |
|  | RDC5 (0.6 mg/kg po)+Naltrexone (1 mg/kg ip) vs RDC5 | 1.8 | 0.536 |
|  | RDC5 (0.6 mg/kg po)+Bupropion (20 mg/kg ip qd) vs Bupropion | -5.5 | 0.066 |
|  | Bupropion (20 mg/kg ip qd)+Naltrexone (1 mg/kg ip) vs Bupropion | -1.0 | 0.727 |
|  | RDC5 (0.6 mg/kg po)+Naltrexone (1 mg/kg ip) vs Naltrexone | 3.4 | 0.247 |
|  | Bupropion (20 mg/kg ip qd)+Naltrexone (1 mg/kg ip) vs Naltrexone | 6.7 | 0.026* |

Multiple comparisons are by the multiple t test. *p<0.05, **p<0.01

| Time | Comparison | Difference | p |
| --- | --- | --- | --- |
| Day 15 | RDC5 (0.6 mg/kg po)+Bupropion (20 mg/kg ip qd) vs RDC5 | -3.1 | 0.280 |
|  | RDC5 (0.6 mg/kg po)+Naltrexone (1 mg/kg ip) vs RDC5 | -2.0 | 0.481 |
|  | RDC5 (0.6 mg/kg po)+Bupropion (20 mg/kg ip qd) vs Bupropion | -6.1 | 0.034* |
|  | Bupropion (20 mg/kg ip qd)+Naltrexone (1 mg/kg ip) vs Bupropion | -3.3 | 0.245 |
|  | RDC5 (0.6 mg/kg po)+Naltrexone (1 mg/kg ip) vs Naltrexone | -0.7 | 0.820 |
|  | Bupropion (20 mg/kg ip qd)+Naltrexone (1 mg/kg ip) vs Naltrexone | 1.1 | 0.703 |
| Day 16 | RDC5 (0.6 mg/kg po)+Bupropion (20 mg/kg ip qd) vs RDC5 | -1.0 | 0.697 |
|  | RDC5 (0.6 mg/kg po)+Naltrexone (1 mg/kg ip) vs RDC5 | 1.2 | 0.639 |
|  | RDC5 (0.6 mg/kg po)+Bupropion (20 mg/kg ip qd) vs Bupropion | -6.7 | 0.014* |
|  | Bupropion (20 mg/kg ip qd)+Naltrexone (1 mg/kg ip) vs Bupropion | -4.2 | 0.120 |
|  | RDC5 (0.6 mg/kg po)+Naltrexone (1 mg/kg ip) vs Naltrexone | 2.1 | 0.422 |
|  | Bupropion (20 mg/kg ip qd)+Naltrexone (1 mg/kg ip) vs Naltrexone | 2.4 | 0.373 |
| Day 17 | RDC5 (0.6 mg/kg po)+Bupropion (20 mg/kg ip qd) vs RDC5 | 3.9 | 0.210 |
|  | RDC5 (0.6 mg/kg po)+Naltrexone (1 mg/kg ip) vs RDC5 | 5.0 | 0.108 |
|  | RDC5 (0.6 mg/kg po)+Bupropion (20 mg/kg ip qd) vs Bupropion | -5.7 | 0.067 |
|  | Bupropion (20 mg/kg ip qd)+Naltrexone (1 mg/kg ip) vs Bupropion | -3.3 | 0.311 |
|  | RDC5 (0.6 mg/kg po)+Naltrexone (1 mg/kg ip) vs Naltrexone | 2.0 | 0.529 |
|  | Bupropion (20 mg/kg ip qd)+Naltrexone (1 mg/kg ip) vs Naltrexone | 3.3 | 0.303 |
| Day 18 | RDC5 (0.6 mg/kg po)+Bupropion (20 mg/kg ip qd) vs RDC5 | -0.8 | 0.811 |
|  | RDC5 (0.6 mg/kg po)+Naltrexone (1 mg/kg ip) vs RDC5 | 1.9 | 0.581 |
|  | RDC5 (0.6 mg/kg po)+Bupropion (20 mg/kg ip qd) vs Bupropion | -8.4 | 0.017* |
|  | Bupropion (20 mg/kg ip qd)+Naltrexone (1 mg/kg ip) vs Bupropion | -3.6 | 0.303 |
|  | RDC5 (0.6 mg/kg po)+Naltrexone (1 mg/kg ip) vs Naltrexone | 1.7 | 0.621 |
|  | Bupropion (20 mg/kg ip qd)+Naltrexone (1 mg/kg ip) vs Naltrexone | 3.8 | 0.274 |
| Day 19 | RDC5 (0.6 mg/kg po)+Bupropion (20 mg/kg ip qd) vs RDC5 | -3.5 | 0.218 |
|  | RDC5 (0.6 mg/kg po)+Naltrexone (1 mg/kg ip) vs RDC5 | -2.1 | 0.458 |
|  | RDC5 (0.6 mg/kg po)+Bupropion (20 mg/kg ip qd) vs Bupropion | -7.0 | 0.015* |
|  | Bupropion (20 mg/kg ip qd)+Naltrexone (1 mg/kg ip) vs Bupropion | -4.3 | 0.136 |
|  | RDC5 (0.6 mg/kg po)+Naltrexone (1 mg/kg ip) vs Naltrexone | -0.0 | 0.987 |
|  | Bupropion (20 mg/kg ip qd)+Naltrexone (1 mg/kg ip) vs Naltrexone | 1.3 | 0.646 |
| Day 20 | RDC5 (0.6 mg/kg po)+Bupropion (20 mg/kg ip qd) vs RDC5 | -2.4 | 0.288 |
|  | RDC5 (0.6 mg/kg po)+Naltrexone (1 mg/kg ip) vs RDC5 | 0.2 | 0.947 |
|  | RDC5 (0.6 mg/kg po)+Bupropion (20 mg/kg ip qd) vs Bupropion | -6.1 | 0.009** |
|  | Bupropion (20 mg/kg ip qd)+Naltrexone (1 mg/kg ip) vs Bupropion | -4.0 | 0.080 |
|  | RDC5 (0.6 mg/kg po)+Naltrexone (1 mg/kg ip) vs Naltrexone | 0.3 | 0.879 |
|  | Bupropion (20 mg/kg ip qd)+Naltrexone (1 mg/kg ip) vs Naltrexone | -0.1 | 0.956 |
| Day 21 | RDC5 (0.6 mg/kg po)+Bupropion (20 mg/kg ip qd) vs RDC5 | -3.4 | 0.345 |
|  | RDC5 (0.6 mg/kg po)+Naltrexone (1 mg/kg ip) vs RDC5 | 0.6 | 0.870 |
|  | RDC5 (0.6 mg/kg po)+Bupropion (20 mg/kg ip qd) vs Bupropion | -5.8 | 0.105 |
|  | Bupropion (20 mg/kg ip qd)+Naltrexone (1 mg/kg ip) vs Bupropion | -3.4 | 0.339 |
|  | RDC5 (0.6 mg/kg po)+Naltrexone (1 mg/kg ip) vs Naltrexone | -0.0 | 0.994 |
|  | Bupropion (20 mg/kg ip qd)+Naltrexone (1 mg/kg ip) vs Naltrexone | -1.6 | 0.657 |

Multiple comparisons are by the multiple t test. *p<0.05, **p<0.01

| Time | Comparison | Difference | p |
| --- | --- | --- | --- |
| Day 22 | RDC5 (0.6 mg/kg po)+Bupropion (20 mg/kg ip qd) vs RDC5 | -0.0 | 0.990 |
|  | RDC5 (0.6 mg/kg po)+Naltrexone (1 mg/kg ip) vs RDC5 | 0.1 | 0.984 |
|  | RDC5 (0.6 mg/kg po)+Bupropion (20 mg/kg ip qd) vs Bupropion | -3.7 | 0.242 |
|  | Bupropion (20 mg/kg ip qd)+Naltrexone (1 mg/kg ip) vs Bupropion | -3.2 | 0.304 |
|  | RDC5 (0.6 mg/kg po)+Naltrexone (1 mg/kg ip) vs Naltrexone | -0.0 | 0.989 |
|  | Bupropion (20 mg/kg ip qd)+Naltrexone (1 mg/kg ip) vs Naltrexone | 0.3 | 0.921 |
| Day 23 | RDC5 (0.6 mg/kg po)+Bupropion (20 mg/kg ip qd) vs RDC5 | 1.1 | 0.720 |
|  | RDC5 (0.6 mg/kg po)+Naltrexone (1 mg/kg ip) vs RDC5 | 2.8 | 0.345 |
|  | RDC5 (0.6 mg/kg po)+Bupropion (20 mg/kg ip qd) vs Bupropion | -5.2 | 0.078 |
|  | Bupropion (20 mg/kg ip qd)+Naltrexone (1 mg/kg ip) vs Bupropion | -2.6 | 0.370 |
|  | RDC5 (0.6 mg/kg po)+Naltrexone (1 mg/kg ip) vs Naltrexone | 1.0 | 0.742 |
|  | Bupropion (20 mg/kg ip qd)+Naltrexone (1 mg/kg ip) vs Naltrexone | 1.8 | 0.545 |
| Day 24 | RDC5 (0.6 mg/kg po)+Bupropion (20 mg/kg ip qd) vs RDC5 | 0.6 | 0.847 |
|  | RDC5 (0.6 mg/kg po)+Naltrexone (1 mg/kg ip) vs RDC5 | 1.0 | 0.727 |
|  | RDC5 (0.6 mg/kg po)+Bupropion (20 mg/kg ip qd) vs Bupropion | -4.6 | 0.125 |
|  | Bupropion (20 mg/kg ip qd)+Naltrexone (1 mg/kg ip) vs Bupropion | -3.3 | 0.262 |
|  | RDC5 (0.6 mg/kg po)+Naltrexone (1 mg/kg ip) vs Naltrexone | 2.7 | 0.361 |
|  | Bupropion (20 mg/kg ip qd)+Naltrexone (1 mg/kg ip) vs Naltrexone | 3.5 | 0.240 |
| Day 25 | RDC5 (0.6 mg/kg po)+Bupropion (20 mg/kg ip qd) vs RDC5 | -0.6 | 0.858 |
|  | RDC5 (0.6 mg/kg po)+Naltrexone (1 mg/kg ip) vs RDC5 | 2.5 | 0.483 |
|  | RDC5 (0.6 mg/kg po)+Bupropion (20 mg/kg ip qd) vs Bupropion | -4.1 | 0.241 |
|  | Bupropion (20 mg/kg ip qd)+Naltrexone (1 mg/kg ip) vs Bupropion | -1.6 | 0.650 |
|  | RDC5 (0.6 mg/kg po)+Naltrexone (1 mg/kg ip) vs Naltrexone | 2.4 | 0.486 |
|  | Bupropion (20 mg/kg ip qd)+Naltrexone (1 mg/kg ip) vs Naltrexone | 1.9 | 0.590 |
| Day 26 | RDC5 (0.6 mg/kg po)+Bupropion (20 mg/kg ip bid) vs RDC5 | 1.8 | 0.583 |
|  | RDC5 (0.6 mg/kg po)+Naltrexone (1 mg/kg ip) vs RDC5 | 3.0 | 0.348 |
|  | RDC5 (0.6 mg/kg po)+Bupropion (20 mg/kg ip bid) vs Bupropion | -3.7 | 0.239 |
|  | Bupropion (20 mg/kg ip bid)+Naltrexone (1 mg/kg ip) vs Bupropion | -2.7 | 0.398 |
|  | RDC5 (0.6 mg/kg po)+Naltrexone (1 mg/kg ip) vs Naltrexone | 2.4 | 0.452 |
|  | Bupropion (20 mg/kg ip bid)+Naltrexone (1 mg/kg ip) vs Naltrexone | 2.2 | 0.493 |
| Day 27 | RDC5 (0.6 mg/kg po)+Bupropion (20 mg/kg ip bid) vs RDC5 | 2.8 | 0.318 |
|  | RDC5 (0.6 mg/kg po)+Naltrexone (1 mg/kg ip) vs RDC5 | 4.8 | 0.088 |
|  | RDC5 (0.6 mg/kg po)+Bupropion (20 mg/kg ip bid) vs Bupropion | -4.4 | 0.116 |
|  | Bupropion (20 mg/kg ip bid)+Naltrexone (1 mg/kg ip) vs Bupropion | -2.1 | 0.452 |
|  | RDC5 (0.6 mg/kg po)+Naltrexone (1 mg/kg ip) vs Naltrexone | 1.8 | 0.529 |
|  | Bupropion (20 mg/kg ip bid)+Naltrexone (1 mg/kg ip) vs Naltrexone | 2.1 | 0.460 |
| Day 28 | RDC5 (0.6 mg/kg po)+Bupropion (20 mg/kg ip bid) vs RDC5 | -0.6 | 0.808 |
|  | RDC5 (0.6 mg/kg po)+Naltrexone (1 mg/kg ip) vs RDC5 | 1.1 | 0.657 |
|  | RDC5 (0.6 mg/kg po)+Bupropion (20 mg/kg ip bid) vs Bupropion | -4.4 | 0.084 |
|  | Bupropion (20 mg/kg ip bid)+Naltrexone (1 mg/kg ip) vs Bupropion | -2.9 | 0.255 |
|  | RDC5 (0.6 mg/kg po)+Naltrexone (1 mg/kg ip) vs Naltrexone | 4.8 | 0.058 |
|  | Bupropion (20 mg/kg ip bid)+Naltrexone (1 mg/kg ip) vs Naltrexone | 4.6 | 0.069 |

Multiple comparisons are by the multiple t test.

| Time | Comparison | Difference | p |
| --- | --- | --- | --- |
| Day 29 | RDC5 (0.6 mg/kg po)+Bupropion (20 mg/kg ip bid) vs RDC5 | -1.2 | 0.641 |
|  | RDC5 (0.6 mg/kg po)+Naltrexone (1 mg/kg ip) vs RDC5 | -0.5 | 0.843 |
|  | RDC5 (0.6 mg/kg po)+Bupropion (20 mg/kg ip bid) vs Bupropion | -4.3 | 0.096 |
|  | Bupropion (20 mg/kg ip bid)+Naltrexone (1 mg/kg ip) vs Bupropion | -1.9 | 0.454 |
|  | RDC5 (0.6 mg/kg po)+Naltrexone (1 mg/kg ip) vs Naltrexone | 3.5 | 0.172 |
|  | Bupropion (20 mg/kg ip bid)+Naltrexone (1 mg/kg ip) vs Naltrexone | 5.2 | 0.044* |
| Day 30 | RDC5 (0.6 mg/kg po)+Bupropion (20 mg/kg ip bid) vs RDC5 | -0.8 | 0.773 |
|  | RDC5 (0.6 mg/kg po)+Naltrexone (1 mg/kg ip) vs RDC5 | 4.0 | 0.131 |
|  | RDC5 (0.6 mg/kg po)+Bupropion (20 mg/kg ip bid) vs Bupropion | -6.1 | 0.024* |
|  | Bupropion (20 mg/kg ip bid)+Naltrexone (1 mg/kg ip) vs Bupropion | -2.5 | 0.352 |
|  | RDC5 (0.6 mg/kg po)+Naltrexone (1 mg/kg ip) vs Naltrexone | 6.2 | 0.021* |
|  | Bupropion (20 mg/kg ip bid)+Naltrexone (1 mg/kg ip) vs Naltrexone | 5.0 | 0.060 |
| Day 31 | RDC5 (0.6 mg/kg po)+Bupropion (20 mg/kg ip bid) vs RDC5 | 1.4 | 0.621 |
|  | RDC5 (0.6 mg/kg po)+Naltrexone (1 mg/kg ip) vs RDC5 | 1.3 | 0.644 |
|  | RDC5 (0.6 mg/kg po)+Bupropion (20 mg/kg ip bid) vs Bupropion | -2.4 | 0.402 |
|  | Bupropion (20 mg/kg ip bid)+Naltrexone (1 mg/kg ip) vs Bupropion | -0.5 | 0.869 |
|  | RDC5 (0.6 mg/kg po)+Naltrexone (1 mg/kg ip) vs Naltrexone | 2.7 | 0.358 |
|  | Bupropion (20 mg/kg ip bid)+Naltrexone (1 mg/kg ip) vs Naltrexone | 4.7 | 0.106 |
| Day 32 | RDC5 (0.6 mg/kg po)+Bupropion (20 mg/kg ip bid) vs RDC5 | -0.1 | 0.963 |
|  | RDC5 (0.6 mg/kg po)+Naltrexone (1 mg/kg ip) vs RDC5 | 3.4 | 0.242 |
|  | RDC5 (0.6 mg/kg po)+Bupropion (20 mg/kg ip bid) vs Bupropion | -1.7 | 0.559 |
|  | Bupropion (20 mg/kg ip bid)+Naltrexone (1 mg/kg ip) vs Bupropion | -0.3 | 0.920 |
|  | RDC5 (0.6 mg/kg po)+Naltrexone (1 mg/kg ip) vs Naltrexone | 3.7 | 0.198 |
|  | Bupropion (20 mg/kg ip bid)+Naltrexone (1 mg/kg ip) vs Naltrexone | 1.6 | 0.578 |
| Day 33 | RDC5 (0.6 mg/kg po)+Bupropion (20 mg/kg ip bid) vs RDC5 | 0.7 | 0.822 |
|  | RDC5 (0.6 mg/kg po)+Naltrexone (1 mg/kg ip) vs RDC5 | 1.7 | 0.603 |
|  | RDC5 (0.6 mg/kg po)+Bupropion (20 mg/kg ip bid) vs Bupropion | -0.4 | 0.909 |
|  | Bupropion (20 mg/kg ip bid)+Naltrexone (1 mg/kg ip) vs Bupropion | -3.4 | 0.301 |
|  | RDC5 (0.6 mg/kg po)+Naltrexone (1 mg/kg ip) vs Naltrexone | 3.2 | 0.324 |
|  | Bupropion (20 mg/kg ip bid)+Naltrexone (1 mg/kg ip) vs Naltrexone | -0.7 | 0.819 |
| Day 34 | RDC5 (0.6 mg/kg po)+Bupropion (20 mg/kg ip bid) vs RDC5 | -2.7 | 0.422 |
|  | RDC5 (0.6 mg/kg po)+Naltrexone (1 mg/kg ip) vs RDC5 | 0.7 | 0.838 |
|  | RDC5 (0.6 mg/kg po)+Bupropion (20 mg/kg ip bid) vs Bupropion | -2.6 | 0.438 |
|  | Bupropion (20 mg/kg ip bid)+Naltrexone (1 mg/kg ip) vs Bupropion | -3.0 | 0.376 |
|  | RDC5 (0.6 mg/kg po)+Naltrexone (1 mg/kg ip) vs Naltrexone | 4.7 | 0.162 |
|  | Bupropion (20 mg/kg ip bid)+Naltrexone (1 mg/kg ip) vs Naltrexone | 1.0 | 0.774 |
| Day 35 | RDC5 (0.6 mg/kg po)+Bupropion (20 mg/kg ip bid) vs RDC5 | -0.3 | 0.911 |
|  | RDC5 (0.6 mg/kg po)+Naltrexone (1 mg/kg ip) vs RDC5 | 1.2 | 0.677 |
|  | RDC5 (0.6 mg/kg po)+Bupropion (20 mg/kg ip bid) vs Bupropion | -2.7 | 0.340 |
|  | Bupropion (20 mg/kg ip bid)+Naltrexone (1 mg/kg ip) vs Bupropion | -3.1 | 0.268 |
|  | RDC5 (0.6 mg/kg po)+Naltrexone (1 mg/kg ip) vs Naltrexone | 5.6 | 0.049* |
|  | Bupropion (20 mg/kg ip bid)+Naltrexone (1 mg/kg ip) vs Naltrexone | 3.7 | 0.193 |

| Time | Comparison | Difference | p |
| --- | --- | --- | --- |
| Day 36 | RDC5 (0.6 mg/kg po)+Bupropion (20 mg/kg ip bid) vs RDC5 | 0.5 | 0.882 |
|  | RDC5 (0.6 mg/kg po)+Naltrexone (1 mg/kg ip) vs RDC5 | -3.7 | 0.284 |
|  | RDC5 (0.6 mg/kg po)+Bupropion (20 mg/kg ip bid) vs Bupropion | -0.8 | 0.820 |
|  | Bupropion (20 mg/kg ip bid)+Naltrexone (1 mg/kg ip) vs Bupropion | 3.7 | 0.293 |
|  | RDC5 (0.6 mg/kg po)+Naltrexone (1 mg/kg ip) vs Naltrexone | 0.1 | 0.967 |
|  | Bupropion (20 mg/kg ip bid)+Naltrexone (1 mg/kg ip) vs Naltrexone | 8.8 | 0.013* |
| Day 37 | RDC5 (0.6 mg/kg po)+Bupropion (20 mg/kg ip bid) vs RDC5 | -0.3 | 0.936 |
|  | RDC5 (0.6 mg/kg po)+Naltrexone (1 mg/kg ip) vs RDC5 | -1.5 | 0.635 |
|  | RDC5 (0.6 mg/kg po)+Bupropion (20 mg/kg ip bid) vs Bupropion | -1.2 | 0.718 |
|  | Bupropion (20 mg/kg ip bid)+Naltrexone (1 mg/kg ip) vs Bupropion | 2.7 | 0.392 |
|  | RDC5 (0.6 mg/kg po)+Naltrexone (1 mg/kg ip) vs Naltrexone | 2.8 | 0.390 |
|  | Bupropion (20 mg/kg ip bid)+Naltrexone (1 mg/kg ip) vs Naltrexone | 7.9 | 0.015* |
| Day 38 | RDC5 (0.6 mg/kg po)+Bupropion (20 mg/kg ip bid) vs RDC5 | -0.4 | 0.887 |
|  | RDC5 (0.6 mg/kg po)+Naltrexone (1 mg/kg ip) vs RDC5 | 1.2 | 0.681 |
|  | RDC5 (0.6 mg/kg po)+Bupropion (20 mg/kg ip bid) vs Bupropion | -4.3 | 0.146 |
|  | Bupropion (20 mg/kg ip bid)+Naltrexone (1 mg/kg ip) vs Bupropion | -1.1 | 0.695 |
|  | RDC5 (0.6 mg/kg po)+Naltrexone (1 mg/kg ip) vs Naltrexone | 4.9 | 0.093 |
|  | Bupropion (20 mg/kg ip bid)+Naltrexone (1 mg/kg ip) vs Naltrexone | 6.4 | 0.029* |
| Day 39 | RDC5 (0.6 mg/kg po)+Bupropion (20 mg/kg ip bid) vs RDC5 | 3.9 | 0.279 |
|  | RDC5 (0.6 mg/kg po)+Naltrexone (1 mg/kg ip) vs RDC5 | 1.9 | 0.592 |
|  | RDC5 (0.6 mg/kg po)+Bupropion (20 mg/kg ip bid) vs Bupropion | 2.6 | 0.464 |
|  | Bupropion (20 mg/kg ip bid)+Naltrexone (1 mg/kg ip) vs Bupropion | 1.6 | 0.653 |
|  | RDC5 (0.6 mg/kg po)+Naltrexone (1 mg/kg ip) vs Naltrexone | 1.2 | 0.732 |
|  | Bupropion (20 mg/kg ip bid)+Naltrexone (1 mg/kg ip) vs Naltrexone | 2.2 | 0.543 |
| Day 40 | RDC5 (0.6 mg/kg po)+Bupropion (20 mg/kg ip bid) vs RDC5 | -0.2 | 0.952 |
|  | RDC5 (0.6 mg/kg po)+Naltrexone (1 mg/kg ip) vs RDC5 | -2.2 | 0.449 |
|  | RDC5 (0.6 mg/kg po)+Bupropion (20 mg/kg ip bid) vs Bupropion | 1.9 | 0.515 |
|  | Bupropion (20 mg/kg ip bid)+Naltrexone (1 mg/kg ip) vs Bupropion | 2.7 | 0.362 |
|  | RDC5 (0.6 mg/kg po)+Naltrexone (1 mg/kg ip) vs Naltrexone | -0.3 | 0.908 |
|  | Bupropion (20 mg/kg ip bid)+Naltrexone (1 mg/kg ip) vs Naltrexone | 2.5 | 0.397 |
| Day 41 | RDC5 (0.6 mg/kg po)+Bupropion (20 mg/kg ip bid) vs RDC5 | -1.0 | 0.763 |
|  | RDC5 (0.6 mg/kg po)+Naltrexone (1 mg/kg ip) vs RDC5 | 1.4 | 0.672 |
|  | RDC5 (0.6 mg/kg po)+Bupropion (20 mg/kg ip bid) vs Bupropion | -0.6 | 0.859 |
|  | Bupropion (20 mg/kg ip bid)+Naltrexone (1 mg/kg ip) vs Bupropion | 0.5 | 0.889 |
|  | RDC5 (0.6 mg/kg po)+Naltrexone (1 mg/kg ip) vs Naltrexone | 3.9 | 0.242 |
|  | Bupropion (20 mg/kg ip bid)+Naltrexone (1 mg/kg ip) vs Naltrexone | 2.6 | 0.444 |
| Day 42 | RDC5 (0.6 mg/kg po)+Bupropion (20 mg/kg ip bid) vs RDC5 | 2.9 | 0.402 |
|  | RDC5 (0.6 mg/kg po)+Naltrexone (1 mg/kg ip) vs RDC5 | 0.6 | 0.862 |
|  | RDC5 (0.6 mg/kg po)+Bupropion (20 mg/kg ip bid) vs Bupropion | 0.4 | 0.898 |
|  | Bupropion (20 mg/kg ip bid)+Naltrexone (1 mg/kg ip) vs Bupropion | 1.0 | 0.773 |
|  | RDC5 (0.6 mg/kg po)+Naltrexone (1 mg/kg ip) vs Naltrexone | 1.6 | 0.637 |
|  | Bupropion (20 mg/kg ip bid)+Naltrexone (1 mg/kg ip) vs Naltrexone | 4.5 | 0.196 |

#### Averages daily water intake (g/day)

Treatment n Mean SEM Difference % change p from vehicle

| Week 1 | Vehicle 4 ml/kg po +2 ml/kg ip | 12 | 25.7 | 0.7 |  | | |
| --- | --- | --- | --- | --- | --- | --- | --- |
| (Day 1-7) | RDC5 0.6 mg/kg po | 10 | 21.3 | 0.8 | -4.4 | -17.1 | 0.006** |
|  | Bupropion 20 mg/kg ip qd | 10 | 28.5 | 1.4 | 2.8 | 10.8 | 0.077 |
|  | Naltrexone 1 mg/kg ip | 10 | 23.8 | 1.0 | -1.9 | -7.2 | 0.233 |
|  | RDC5 0.6 mg/kg po+Bupropion 20 mg/kg ip qd | 10 | 23.0 | 0.9 | -2.7 | -10.3 | 0.091 |
|  | RDC5 0.6 mg/kg po+Naltrexone 1 mg/kg ip | 10 | 22.3 | 1.0 | -3.4 | -13.1 | 0.033* |
|  | Bupropion 20 mg/kg ip qd+Naltrexone 1 mg/kg ip | 10 | 25.7 | 0.8 | 0.0 | 0.0 | 0.997 |
|  | Sibutramine 5 mg/kg po | 10 | 22.7 | 1.7 | -3.0 | -11.6 | 0.057 |
| Week 2 | Vehicle 4 ml/kg po +2 ml/kg ip | 12 | 27.0 | 1.3 |  |  |  |
| (Day 8-14) | RDC5 0.6 mg/kg po qd | 10 | 23.1 | 1.0 | -3.9 | -14.6 | 0.026* |
|  | Bupropion 20 mg/kg ip | 10 | 28.3 | 1.0 | 1.2 | 4.5 | 0.484 |
|  | Naltrexone 1 mg/kg ip | 10 | 22.5 | 0.6 | -4.5 | -16.7 | 0.011* |
|  | RDC5 0.6 mg/kg po+Bupropion 20 mg/kg ip qd | 10 | 22.3 | 1.3 | -4.7 | -17.5 | 0.008** |
|  | RDC5 0.6 mg/kg po+Naltrexone 1 mg/kg ip | 10 | 24.2 | 1.6 | -2.9 | -10.6 | 0.103 |
|  | Bupropion 20 mg/kg ip qd+Naltrexone 1 mg/kg ip | 10 | 26.0 | 1.6 | -1.1 | -3.9 | 0.545 |
|  | Sibutramine 5 mg/kg po | 10 | 30.2 | 3.1 | 3.2 | 11.7 | 0.073 |
| Week 3 | Vehicle 4 ml/kg po +2 ml/kg ip | 12 | 26.0 | 0.9 |  |  |  |
| (Day 15-21) | RDC5 0.6 mg/kg po | 10 | 24.3 | 1.8 | -1.7 | -6.6 | 0.462 |
|  | Bupropion 20 mg/kg ip qd | 10 | 29.3 | 1.4 | 3.3 | 12.7 | 0.162 |
|  | Naltrexone 1 mg/kg ip | 10 | 24.1 | 1.2 | -1.9 | -7.4 | 0.415 |
|  | RDC5 0.6 mg/kg po+Bupropion 20 mg/kg ip qd | 10 | 22.6 | 2.1 | -3.4 | -13.0 | 0.151 |
|  | RDC5 0.6 mg/kg po+Naltrexone 1 mg/kg ip | 10 | 25.0 | 2.2 | -1.0 | -3.9 | 0.665 |
|  | Bupropion 20 mg/kg ip qd+Naltrexone 1 mg/kg ip | 10 | 25.8 | 1.9 | -0.2 | -0.8 | 0.929 |
|  | Sibutramine 5 mg/kg po | 10 | 31.3 | 4.8 | 5.3 | 20.2 | 0.028* |
| Week 4 | Vehicle 4 ml/kg po +2 ml/kg ip | 12 | 27.9 | 1.6 |  |  |  |
| (Day 22-25) | RDC5 0.6 mg/kg po | 10 | 24.6 | 1.7 | -3.3 | -11.8 | 0.207 |
|  | Bupropion 20 mg/kg ip qd | 10 | 28.6 | 1.9 | 0.7 | 2.5 | 0.785 |
|  | Naltrexone 1 mg/kg ip | 10 | 24.7 | 1.2 | -3.3 | -11.6 | 0.212 |
|  | RDC5 0.6 mg/kg po+Bupropion 20 mg/kg ip qd | 10 | 24.7 | 1.8 | -3.3 | -11.8 | 0.208 |
|  | RDC5 0.6 mg/kg po+Naltrexone 1 mg/kg ip | 10 | 26.4 | 2.0 | -1.6 | -5.7 | 0.542 |
|  | Bupropion 20 mg/kg ip qd+Naltrexone 1 mg/kg ip | 10 | 26.4 | 1.1 | -1.6 | -5.7 | 0.541 |
|  | Sibutramine 5 mg/kg po | 10 | 34.0 | 3.4 | 6.1 | 21.9 | 0.021* |
| Week 5 | Vehicle 4 ml/kg po +2 ml/kg ip | 12 | 28.6 | 1.2 |  |  |  |
| (Day 26-35) | RDC5 0.6 mg/kg po | 10 | 25.7 | 1.5 | -2.9 | -10.2 | 0.161 |
|  | Bupropion 20 mg/kg ip bid | 10 | 28.5 | 1.3 | -0.1 | -0.3 | 0.966 |
|  | Naltrexone 1 mg/kg ip | 10 | 23.2 | 1.1 | -5.4 | -18.9 | 0.011* |
|  | RDC5 0.6 mg/kg po+Bupropion 20 mg/kg ip bid | 10 | 25.2 | 1.5 | -3.5 | -12.1 | 0.097 |
|  | RDC5 0.6 mg/kg po+Naltrexone 1 mg/kg ip | 10 | 27.7 | 1.9 | -0.9 | -3.3 | 0.652 |
|  | Bupropion 20 mg/kg ip bid+Naltrexone 1 mg/kg ip | 10 | 26.0 | 2.0 | -2.6 | -9.1 | 0.214 |
|  | Sibutramine 5 mg/kg po | 10 | 31.3 | 4.4 | 2.7 | 9.3 | 0.202 |
| Week 6 | Vehicle 4 ml/kg po +2 ml/kg ip | 12 | 27.3 | 0.8 |  |  |  |
| (Day 36-42) | RDC5 0.6 mg/kg po | 10 | 26.2 | 2.2 | -1.1 | -4.0 | 0.602 |
|  | Bupropion 20 mg/kg ip bid | 10 | 27.9 | 1.3 | 0.6 | 2.2 | 0.776 |
|  | Naltrexone 1 mg/kg ip | 10 | 23.9 | 1.1 | -3.4 | -12.6 | 0.100 |
|  | RDC5 0.6 mg/kg po+Bupropion 20 mg/kg ip bid | 10 | 27.6 | 1.5 | 0.3 | 1.2 | 0.876 |
|  | RDC5 0.6 mg/kg po+Naltrexone 1 mg/kg ip | 10 | 26.9 | 1.8 | -0.4 | -1.4 | 0.849 |
|  | Bupropion 20 mg/kg ip bid+Naltrexone 1 mg/kg ip | 10 | 28.7 | 2.4 | 1.3 | 4.9 | 0.516 |
|  | Sibutramine 5 mg/kg po | 10 | 31.4 | 3.9 | 4.1 | 14.8 | 0.053 |

Means are adjusted for differences between the treatment groups at baseline (average of Days -6 to 0). SEM are calculated from the residuals of the statistical model. Multiple comparisons against vehicle are by the multiple t test. *p<0.05, **p<0.01.

#### Table 7 cont. Average daily water intake (g/day)

Treatment n Mean SEM Difference % change p from vehicle

| Weeks 1-4 Vehicle 4 ml/kg po +2 ml/kg ip | 12 | 26.6 | 0.8 |  | | |
| --- | --- | --- | --- | --- | --- | --- |
| (Day 1-25) RDC5 0.6 mg/kg po | 10 | 23.4 | 1.2 | -3.1 | -11.8 | 0.066 |
| Bupropion 20 mg/kg ip qd | 10 | 28.8 | 1.2 | 2.2 | 8.3 | 0.191 |
| Naltrexone 1 mg/kg ip | 10 | 23.8 | 0.6 | -2.8 | -10.5 | 0.099 |
| RDC5 0.6 mg/kg po+Bupropion 20 mg/kg ip qd | 10 | 23.1 | 1.4 | -3.5 | -13.0 | 0.043* |
| RDC5 0.6 mg/kg po+Naltrexone 1 mg/kg ip | 10 | 24.5 | 1.5 | -2.0 | -7.7 | 0.228 |
| Bupropion 20 mg/kg ip qd+Naltrexone 1 mg/kg ip | 10 | 26.1 | 1.0 | -0.5 | -1.7 | 0.784 |
| Sibutramine 5 mg/kg po | 10 | 29.9 | 3.0 | 3.3 | 12.4 | 0.054 |
| Weeks 5-6 Vehicle 4 ml/kg po +2 ml/kg ip | 12 | 27.9 | 0.9 |  |  |  |
| (Day 26-42) RDC5 0.6 mg/kg po | 10 | 26.0 | 1.8 | -2.0 | -7.0 | 0.276 |
| Bupropion 20 mg/kg ip bid | 10 | 28.4 | 1.2 | 0.5 | 1.7 | 0.789 |
| Naltrexone 1 mg/kg ip | 10 | 23.5 | 1.0 | -4.4 | -15.7 | 0.017* |
| RDC5 0.6 mg/kg po+Bupropion 20 mg/kg ip bid | 10 | 26.0 | 1.1 | -1.9 | -6.8 | 0.295 |
| RDC5 0.6 mg/kg po+Naltrexone 1 mg/kg ip | 10 | 27.5 | 1.8 | -0.4 | -1.6 | 0.804 |
| Bupropion 20 mg/kg ip bid+Naltrexone 1 mg/kg ip | 10 | 27.6 | 1.7 | -0.3 | -1.2 | 0.858 |
| Sibutramine 5 mg/kg po | 10 | 31.3 | 4.1 | 3.3 | 11.9 | 0.067 |
| Weeks 1-5 Vehicle 4 ml/kg po +2 ml/kg ip | 12 | 27.1 | 0.8 |  |  |  |
| (Day 1-35) RDC5 0.6 mg/kg po | 10 | 24.4 | 1.2 | -2.7 | -10.1 | 0.106 |
| Bupropion 20 mg/kg ip qd/bid | 10 | 28.7 | 1.2 | 1.6 | 6.0 | 0.329 |
| Naltrexone 1 mg/kg ip | 10 | 23.7 | 0.7 | -3.4 | -12.5 | 0.045* |
| RDC5 0.6 mg/kg po+Bupropion 20 mg/kg ip qd/bid | 10 | 24.1 | 1.4 | -3.0 | -10.9 | 0.079 |
| RDC5 0.6 mg/kg po+Naltrexone 1 mg/kg ip | 10 | 25.7 | 1.6 | -1.4 | -5.1 | 0.412 |
| Bupropion 20 mg/kg ip qd/bid +Naltrexone 1 mg/kg ip | 10 | 26.6 | 0.9 | -0.5 | -1.9 | 0.758 |
| Sibutramine 5 mg/kg po | 10 | 30.3 | 3.4 | 3.2 | 11.7 | 0.062 |
| Overall Vehicle 4 ml/kg po +2 ml/kg ip | 12 | 27.1 | 0.7 |  |  |  |
| (Day 1-42) RDC5 0.6 mg/kg po | 10 | 24.8 | 1.3 | -2.2 | -8.3 | 0.177 |
| Bupropion 20 mg/kg ip qd/bid | 10 | 28.7 | 1.1 | 1.6 | 5.8 | 0.340 |
| Naltrexone 1 mg/kg ip | 10 | 23.7 | 0.7 | -3.4 | -12.6 | 0.042* |
| RDC5 0.6 mg/kg po+Bupropion 20 mg/kg ip qd/bid | 10 | 24.6 | 1.2 | -2.5 | -9.2 | 0.137 |
| RDC5 0.6 mg/kg po+Naltrexone 1 mg/kg ip | 10 | 26.0 | 1.6 | -1.1 | -4.1 | 0.504 |
| Bupropion 20 mg/kg ip qd/bid +Naltrexone 1 mg/kg ip | 10 | 26.9 | 1.1 | -0.2 | -0.7 | 0.915 |
| Sibutramine 5 mg/kg po | 10 | 30.4 | 3.4 | 3.4 | 12.4 | 0.045* |

Means are adjusted for differences between the treatment groups at baseline (average of Days -6 to 0). SEM are calculated from the residuals of the statistical model. Multiple comparisons against vehicle are by the multiple t test. *p<0.05.

#### Table 7 cont. Average daily water intake (g/day) comparisons of combination treatments to their individual constituents

| Time | Comparison | Difference | p |
| --- | --- | --- | --- |
| Week 1 | RDC5 (0.6 mg/kg po)+Bupropion (20 mg/kg ip qd) vs RDC5 | 1.7 | 0.284 |
| (Day 1-7) | RDC5 (0.6 mg/kg po)+Naltrexone (1 mg/kg ip) vs RDC5 | 1.0 | 0.524 |
|  | RDC5 (0.6 mg/kg po)+Bupropion (20 mg/kg ip qd) vs Bupropion | -5.4 | 0.001** |
|  | Bupropion (20 mg/kg ip qd)+Naltrexone (1 mg/kg ip) vs Bupropion | -2.8 | 0.090 |
|  | RDC5 (0.6 mg/kg po)+Naltrexone (1 mg/kg ip) vs Naltrexone | -1.5 | 0.356 |
|  | Bupropion (20 mg/kg ip qd)+Naltrexone (1 mg/kg ip) vs Naltrexone | 1.9 | 0.252 |
| Week 2 | RDC5 (0.6 mg/kg po)+Bupropion (20 mg/kg ip qd) vs RDC5 | -0.8 | 0.658 |
| (Day 8-14) | RDC5 (0.6 mg/kg po)+Naltrexone (1 mg/kg ip) vs RDC5 | 1.1 | 0.555 |
|  | RDC5 (0.6 mg/kg po)+Bupropion (20 mg/kg ip qd) vs Bupropion | -6.0 | 0.002** |
|  | Bupropion (20 mg/kg ip qd)+Naltrexone (1 mg/kg ip) vs Bupropion | -2.3 | 0.213 |
|  | RDC5 (0.6 mg/kg po)+Naltrexone (1 mg/kg ip) vs Naltrexone | 1.7 | 0.360 |
|  | Bupropion (20 mg/kg ip qd)+Naltrexone (1 mg/kg ip) vs Naltrexone | 3.5 | 0.058 |
| Week 3 | RDC5 (0.6 mg/kg po)+Bupropion (20 mg/kg ip qd) vs RDC5 | -1.7 | 0.498 |
| (Day 15-21) | RDC5 (0.6 mg/kg po)+Naltrexone (1 mg/kg ip) vs RDC5 | 0.7 | 0.772 |
|  | RDC5 (0.6 mg/kg po)+Bupropion (20 mg/kg ip qd) vs Bupropion | -6.7 | 0.008** |
|  | Bupropion (20 mg/kg ip qd)+Naltrexone (1 mg/kg ip) vs Bupropion | -3.5 | 0.155 |
|  | RDC5 (0.6 mg/kg po)+Naltrexone (1 mg/kg ip) vs Naltrexone | 0.9 | 0.714 |
|  | Bupropion (20 mg/kg ip qd)+Naltrexone (1 mg/kg ip) vs Naltrexone | 1.7 | 0.486 |
| Week 4 | RDC5 (0.6 mg/kg po)+Bupropion (20 mg/kg ip qd) vs RDC5 | 0.0 | 0.998 |
| (Day 22-25) | RDC5 (0.6 mg/kg po)+Naltrexone (1 mg/kg ip) vs RDC5 | 1.7 | 0.529 |
|  | RDC5 (0.6 mg/kg po)+Bupropion (20 mg/kg ip qd) vs Bupropion | -4.0 | 0.144 |
|  | Bupropion (20 mg/kg ip qd)+Naltrexone (1 mg/kg ip) vs Bupropion | -2.3 | 0.398 |
|  | RDC5 (0.6 mg/kg po)+Naltrexone (1 mg/kg ip) vs Naltrexone | 1.7 | 0.538 |
|  | Bupropion (20 mg/kg ip qd)+Naltrexone (1 mg/kg ip) vs Naltrexone | 1.7 | 0.539 |
| Week 5 | RDC5 (0.6 mg/kg po)+Bupropion (20 mg/kg ip bid) vs RDC5 | -0.5 | 0.802 |
| (Day 26-35) | RDC5 (0.6 mg/kg po)+Naltrexone (1 mg/kg ip) vs RDC5 | 2.0 | 0.360 |
|  | RDC5 (0.6 mg/kg po)+Bupropion (20 mg/kg ip bid) vs Bupropion | -3.4 | 0.121 |
|  | Bupropion (20 mg/kg ip bid)+Naltrexone (1 mg/kg ip) vs Bupropion | -2.5 | 0.250 |
|  | RDC5 (0.6 mg/kg po)+Naltrexone (1 mg/kg ip) vs Naltrexone | 4.5 | 0.043* |
|  | Bupropion (20 mg/kg ip bid)+Naltrexone (1 mg/kg ip) vs Naltrexone | 2.8 | 0.198 |
| Week 6 | RDC5 (0.6 mg/kg po)+Bupropion (20 mg/kg ip bid) vs RDC5 | 1.4 | 0.517 |
| (Day 36-42) | RDC5 (0.6 mg/kg po)+Naltrexone (1 mg/kg ip) vs RDC5 | 0.7 | 0.752 |
|  | RDC5 (0.6 mg/kg po)+Bupropion (20 mg/kg ip bid) vs Bupropion | -0.3 | 0.902 |
|  | Bupropion (20 mg/kg ip bid)+Naltrexone (1 mg/kg ip) vs Bupropion | 0.8 | 0.726 |
|  | RDC5 (0.6 mg/kg po)+Naltrexone (1 mg/kg ip) vs Naltrexone | 3.0 | 0.162 |
|  | Bupropion (20 mg/kg ip bid)+Naltrexone (1 mg/kg ip) vs Naltrexone | 4.8 | 0.029* |

Multiple comparisons are by the multiple t test. *p<0.05.

#### Table 7 cont. Average daily water intake (g/day) comparisons of combination treatments to their individual constituents

| Time | Comparison | Difference | p |
| --- | --- | --- | --- |
| Weeks 1-4 | RDC5 (0.6 mg/kg po)+Bupropion (20 mg/kg ip qd) vs RDC5 | -0.3 | 0.850 |
| (Day 1-25) | RDC5 (0.6 mg/kg po)+Naltrexone (1 mg/kg ip) vs RDC5 | 1.1 | 0.538 |
|  | RDC5 (0.6 mg/kg po)+Bupropion (20 mg/kg ip qd) vs Bupropion | -5.7 | 0.002** |
|  | Bupropion (20 mg/kg ip qd)+Naltrexone (1 mg/kg ip) vs Bupropion | -2.7 | 0.131 |
|  | RDC5 (0.6 mg/kg po)+Naltrexone (1 mg/kg ip) vs Naltrexone | 0.8 | 0.666 |
|  | Bupropion (20 mg/kg ip qd)+Naltrexone (1 mg/kg ip) vs Naltrexone | 2.3 | 0.186 |
| Weeks 5-6 | RDC5 (0.6 mg/kg po)+Bupropion (20 mg/kg ip bid) vs RDC5 | 0.1 | 0.966 |
| (Day 26-42) | RDC5 (0.6 mg/kg po)+Naltrexone (1 mg/kg ip) vs RDC5 | 1.5 | 0.420 |
|  | RDC5 (0.6 mg/kg po)+Bupropion (20 mg/kg ip bid) vs Bupropion | -2.4 | 0.209 |
|  | Bupropion (20 mg/kg ip bid)+Naltrexone (1 mg/kg ip) vs Bupropion | -0.8 | 0.669 |
|  | RDC5 (0.6 mg/kg po)+Naltrexone (1 mg/kg ip) vs Naltrexone | 3.9 | 0.039* |
|  | Bupropion (20 mg/kg ip bid)+Naltrexone (1 mg/kg ip) vs Naltrexone | 4.1 | 0.034* |
| Weeks 1-5 | RDC5 (0.6 mg/kg po)+Bupropion (20 mg/kg ip qd/bid) vs RDC5 | -0.2 | 0.892 |
| (Day 1-35) | RDC5 (0.6 mg/kg po)+Naltrexone (1 mg/kg ip) vs RDC5 | 1.4 | 0.440 |
|  | RDC5 (0.6 mg/kg po)+Bupropion (20 mg/kg ip qd/bid) vs Bupropion | -4.6 | 0.010* |
|  | Bupropion (20 mg/kg ip qd/bid)+Naltrexone (1 mg/kg ip) vs Bupropion | -2.2 | 0.220 |
|  | RDC5 (0.6 mg/kg po)+Naltrexone (1 mg/kg ip) vs Naltrexone | 2.0 | 0.249 |
|  | Bupropion (20 mg/kg ip qd/bid)+Naltrexone (1 mg/kg ip) vs Naltrexone | 2.9 | 0.102 |
| Overall | RDC5 (0.6 mg/kg po)+Bupropion (20 mg/kg ip qd/bid) vs RDC5 | -0.2 | 0.894 |
| (Day 1-42) | RDC5 (0.6 mg/kg po)+Naltrexone (1 mg/kg ip) vs RDC5 | 1.1 | 0.511 |
|  | RDC5 (0.6 mg/kg po)+Bupropion (20 mg/kg ip qd/bid) vs Bupropion | -4.1 | 0.021* |
|  | Bupropion (20 mg/kg ip qd/bid)+Naltrexone (1 mg/kg ip) vs Bupropion | -1.8 | 0.311 |
|  | RDC5 (0.6 mg/kg po)+Naltrexone (1 mg/kg ip) vs Naltrexone | 2.3 | 0.186 |
|  | Bupropion (20 mg/kg ip qd/bid)+Naltrexone (1 mg/kg ip) vs Naltrexone | 3.2 | 0.064 |

Multiple comparisons are by the multiple t test. *p<0.05, **p<0.01.

| **Plasma glucose (mM)**  Group | B1 mean SEM p | | | B2 mean SEM p | | | 10 min mean SEM p | | | 20 min mean SEM p | | |
| --- | --- | --- | --- | --- | --- | --- | --- | --- | --- | --- | --- | --- |
| A: Vehicle po + Vehicle ip | 6.38 | 0.35 |  | 5.81 | 0.20 |  | 9.26 | 0.46 |  | 11.24 | 0.49 |  |
| B: RDC5 0.6 mg/kg po | 5.74 | 0.22 | 0.138 | 5.62 | 0.18 | 0.552 | 7.99 | 0.46 | 0.084 | 10.07 | 0.78 | 0.214 |
| C: Bupropion 20 mg/kg ip qd 1-25/bid 26-43 | 6.94 | 0.29 | 0.233 | 6.02 | 0.29 | 0.543 | 9.78 | 0.64 | 0.518 | 12.28 | 0.64 | 0.311 |
| D: Naltrexone 1 mg/kg ip | 6.15 | 0.60 | 0.610 | 5.82 | 0.17 | >0.999 | 8.69 | 0.35 | 0.456 | 9.75 | 0.46 | 0.109 |
| E: RDC5 0.6 mg/kg po + Bupropion 20 mg/kg ip qd 1-25/bid 26-43 | 6.10 | 0.22 | 0.527 | 5.44 | 0.23 | 0.252 | 8.34 | 0.38 | 0.218 | 8.94 | 0.55 | 0.011* ### |
| F: RDC5 0.6 mg/kg po + Naltrexone 1 mg/kg ip | 6.27 | 0.31 | 0.814 | 5.76 | 0.25 | 0.866 | 8.32 | 0.71 | 0.209 | 9.26 | 0.82 | 0.029* |
| G: Bupropion 20 mg/kg ip qd 1-25/bid 26-43 + Naltrexone 1 mg/kg ip | 6.85 | 0.34 | 0.310 | 6.59 | 0.21 | 0.031* ^†^ | 9.18 | 0.54 | 0.923 | 10.66 | 0.66 | 0.545 |
| H: Sibutramine 5 mg/kg po | 5.78 | 0.34 | 0.166 | 4.86 | 0.25 | 0.002** | 8.51 | 0.44 | 0.322 | 9.57 | 0.62 | 0.069 |
| **Plasma glucose (mM)** | 30 min | | | 45 min | | | 60 min | | | 120 min | | |
| Group | mean SEM p | | | mean SEM p | | | mean SEM p | | | mean SEM p | | |
| A: Vehicle po + Vehicle ip | 11.26 | 0.62 |  | 10.09 | 0.41 |  | 10.05 | 0.36 |  | 6.51 | 0.24 |  |
| B: RDC5 0.6 mg/kg po | 11.20 | 0.86 | 0.949 | 10.72 | 0.94 | 0.362 | 10.99 | 0.91 | 0.177 | 8.19 | 0.31 | <0.001*** |
| C: Bupropion 20 mg/kg ip qd 1-25/bid 26-43 | 12.01 | 0.67 | 0.433 | 10.17 | 0.44 | 0.903 | 9.89 | 0.42 | 0.815 | 6.94 | 0.32 | 0.233 |
| D: Naltrexone 1 mg/kg ip | 10.27 | 0.66 | 0.259 | 8.98 | 0.34 | 0.082 | 8.82 | 0.20 | 0.051 | 6.73 | 0.17 | 0.539 |
| E: RDC5 0.6 mg/kg po + Bupropion 20 mg/kg ip qd 1-25/bid 26-43 | 10.31 | 0.56 | 0.281 | 9.14 | 0.56 | 0.137^Ψ^ | 9.03 | 0.37 | 0.109^ΨΨ^ | 7.13 | 0.39 | 0.092^Ψ^ |
| F: RDC5 0.6 mg/kg po + Naltrexone 1 mg/kg ip | 10.15 | 0.50 | 0.205 | 9.31 | 0.47 | 0.226^Ψ^ | 8.83 | 0.62 | 0.052^ΨΨ^ | 8.24 | 0.58 | <0.001*** ^†††^ |
| G: Bupropion 20 mg/kg ip qd 1-25/bid 26-43 + Naltrexone 1 mg/kg ip | 11.06 | 0.79 | 0.822 | 9.55 | 0.55 | 0.406 | 9.60 | 0.44 | 0.488 | 7.21 | 0.24 | 0.062 |
| H: Sibutramine 5 mg/kg po | 9.75 | 0.42 | 0.079 | 8.47 | 0.37 | 0.010** | 8.68 | 0.33 | 0.029* | 6.37 | 0.23 | 0.704 |

**Plasma glucose (mM.h)**

AUC (0-60 min) AUCB2 (0-60 min) AUC (0-120 min) AUCB2 (0-120 min)

Group mean SEM p mean SEM p mean SEM p mean SEM p

| A: Vehicle po + Vehicle ip | 10.03 | 0.28 |  | 4.20 | 0.25 |  | 18.41 | 0.48 |  | 6.74 | 0.44 |  |
| --- | --- | --- | --- | --- | --- | --- | --- | --- | --- | --- | --- | --- |
| B: RDC5 0.6 mg/kg po | 9.88 | 0.71 | 0.796 | 4.34 | 0.66 | 0.805 | 19.56 | 1.17 | 0.248 | 8.32 | 1.08 | 0.114 |
| C: Bupropion 20 mg/kg ip qd 1-25/bid 26-43 | 10.47 | 0.41 | 0.485 | 4.65 | 0.54 | 0.421 | 18.99 | 0.56 | 0.551 | 7.17 | 0.90 | 0.664 |
| D: Naltrexone 1 mg/kg ip | 9.14 | 0.32 | 0.130 | 3.37 | 0.35 | 0.136 | 16.93 | 0.45 | 0.111 | 5.41 | 0.55 | 0.183 |
| E: RDC5 0.6 mg/kg po + Bupropion 20 mg/kg ip qd 1-25/bid 26-43 | 9.01 | 0.43 | 0.082^#^ | 3.52 | 0.33 | 0.217 | 17.10 | 0.69 | 0.159^Ψ^ | 6.06 | 0.59 | 0.4931^Ψ^ |
| F: RDC5 0.6 mg/kg po + Naltrexone 1 mg/kg ip | 9.10 | 0.49 | 0.113 | 3.39 | 0.36 | 0.145 | 18.04 | 0.98 | 0.691 | 6.66 | 0.80 | 0.939 |
| G: Bupropion 20 mg/kg ip qd 1-25/bid 26-43 + Naltrexone 1 mg/kg ip | 9.71 | 0.50 | 0.593 | 3.14 | 0.38 | 0.057^#^ | 18.20 | 0.77 | 0.821 | 4.97 | 0.59 | 0.078^#^ |
| H: Sibutramine 5 mg/kg po | 8.64 | 0.35 | 0.017* | 3.89 | 0.36 | 0.564 | 16.10 | 0.55 | 0.011* | 6.61 | 0.63 | 0.896 |

Animals were fasted for 16 h prior to an OGTT performed on Day 37. Samples were analysed for plasma glucose and area under the curve (AUC) and area above the Baseline 2 (AUCB2) calculated for 0-60 and 0-120 min. Analysis was by robust regression including treatment and assay cohort as factors and Day 1 body weight and bleeding order as covariates. A log transformation was used except for the AUCB2 calculations. Data are shown as adjusted means (n=10-12) and SEM were calculated from the residuals of the statistical models. Comparisons to vehicle were by the multiple t test and significances are denoted by *p<0.05, **p<0.01 and ***p<0.001. The p values shown above are from the comparison to vehicle. Comparisons of combination groups to single treatments were also by the multiple t test and significances against RDC5 alone are denoted by Ψp<0.05 and by ΨΨp<0.01, significances against bupropion alone are denoted by #p<0.05 and by ###p<0.001, whilst significances against naltrexone alone are denoted by †p<0.05 and †††p<0.001.

**Plasma insulin (ng/ml)**

B1 B2 10 min 20 min

Group mean SEM p mean SEM p mean SEM p mean SEM p

| A: Vehicle po + Vehicle ip | 0.83 | 0.15 |  | 0.62 | 0.11 |  | 6.31 | 0.86 |  | 5.07 | 0.76 |  |
| --- | --- | --- | --- | --- | --- | --- | --- | --- | --- | --- | --- | --- |
| B: RDC5 0.6 mg/kg po | 0.50 | 0.11 | 0.019* | 0.50 | 0.14 | 0.300 | 2.55 | 0.33 | <0.001*** | 3.28 | 0.61 | 0.068 |
| C: Bupropion 20 mg/kg ip qd 1-25/bid 26-43 | 0.82 | 0.09 | 0.927 | 0.57 | 0.07 | 0.661 | 4.87 | 0.82 | 0.272 | 6.19 | 0.60 | 0.403 |
| D: Naltrexone 1 mg/kg ip | 0.79 | 0.11 | 0.817 | 0.71 | 0.09 | 0.544 | 4.84 | 0.66 | 0.258 | 5.05 | 0.91 | 0.983 |
| E: RDC5 0.6 mg/kg po + Bupropion 20 mg/kg ip qd 1-25/bid 26-43 | 0.70 | 0.08 | 0.417 | 0.63 | 0.07 | 0.962 | 2.88 | 0.55 | 0.001** ^#^ | 3.01 | 0.61 | 0.030* ^##^ |
| F: RDC5 0.6 mg/kg po + Naltrexone 1 mg/kg ip | 0.65 | 0.09 | 0.245 | 0.61 | 0.08 | 0.922 | 3.40 | 0.79 | 0.010* | 3.49 | 0.61 | 0.118 |
| G: Bupropion 20 mg/kg ip qd 1-25/bid 26-43 + Naltrexone 1 mg/kg ip | 0.83 | 0.08 | >0.999 | 0.68 | 0.09 | 0.708 | 4.46 | 0.62 | 0.142 | 5.26 | 0.96 | 0.881 |
| H: Sibutramine 5 mg/kg po | 0.64 | 0.10 | 0.238 | 0.53 | 0.08 | 0.417 | 3.51 | 0.93 | 0.014* | 3.38 | 0.59 | 0.091 |

**Plasma insulin (ng/ml)**

30 min 45 min 60 min 120 min

Group mean SEM p mean SEM p mean SEM p mean SEM p

| A: Vehicle po + Vehicle ip | 4.29 | 0.34 |  | 3.31 | 0.37 |  | 3.12 | 0.25 |  | 1.56 | 0.17 |  |
| --- | --- | --- | --- | --- | --- | --- | --- | --- | --- | --- | --- | --- |
| B: RDC5 0.6 mg/kg po | 2.78 | 0.59 | 0.052 | 1.99 | 0.47 | 0.021* | 1.93 | 0.41 | 0.009** | 1.66 | 0.34 | 0.767 |
| C: Bupropion 20 mg/kg ip qd 1-25/bid 26-43 | 3.60 | 0.56 | 0.429 | 2.78 | 0.50 | 0.422 | 2.79 | 0.35 | 0.527 | 1.52 | 0.12 | 0.890 |
| D: Naltrexone 1 mg/kg ip | 3.59 | 0.60 | 0.422 | 2.76 | 0.37 | 0.407 | 2.94 | 0.28 | 0.736 | 1.67 | 0.28 | 0.730 |
| E: RDC5 0.6 mg/kg po + Bupropion 20 mg/kg ip qd 1-25/bid 26-43 | 2.03 | 0.24 | 0.001** ^#^ | 1.83 | 0.21 | 0.008** | 2.14 | 0.24 | 0.037* | 1.63 | 0.21 | 0.831 |
| F: RDC5 0.6 mg/kg po + Naltrexone 1 mg/kg ip | 2.84 | 0.63 | 0.066 | 1.92 | 0.30 | 0.015* | 1.81 | 0.33 | 0.003** ^†^ | 1.92 | 0.18 | 0.297 |
| G: Bupropion 20 mg/kg ip qd 1-25/bid 26-43 + Naltrexone 1 mg/kg ip | 3.23 | 0.75 | 0.201 | 2.36 | 0.43 | 0.122 | 2.86 | 0.45 | 0.621 | 1.54 | 0.21 | 0.937 |
| H: Sibutramine 5 mg/kg po | 2.04 | 0.41 | 0.001** | 1.94 | 0.25 | 0.017* | 1.84 | 0.15 | 0.004** | 1.30 | 0.19 | 0.357 |

**Plasma insulin (ng.h/ml)**

AUC (0-60 min) AUCB2 (0-60 min) AUC (0-120 min) AUCB2 (0-120 min)

Group mean SEM p mean SEM p mean SEM p mean SEM p

| A: Vehicle po + Vehicle ip | 4.28 | 0.31 |  | 3.82 | 0.34 |  | 6.65 | 0.48 |  | 5.61 | 0.56 |  |
| --- | --- | --- | --- | --- | --- | --- | --- | --- | --- | --- | --- | --- |
| B: RDC5 0.6 mg/kg po | 2.33 | 0.39 | 0.002** | 2.02 | 0.43 | 0.003** | 4.08 | 0.72 | 0.005** | 3.44 | 0.92 | 0.013* |
| C: Bupropion 20 mg/kg ip qd 1-25/bid 26-43 | 3.79 | 0.42 | 0.531 | 3.33 | 0.50 | 0.398 | 6.04 | 0.56 | 0.564 | 5.06 | 0.69 | 0.514 |
| D: Naltrexone 1 mg/kg ip | 3.62 | 0.42 | 0.393 | 3.01 | 0.47 | 0.165 | 6.04 | 0.55 | 0.569 | 4.74 | 0.53 | 0.307 |
| E: RDC5 0.6 mg/kg po + Bupropion 20 mg/kg ip qd 1-25/bid 26-43 | 2.28 | 0.28 | 0.002** ^#^ | 1.75 | 0.35 | <0.001*** ^#^ | 4.21 | 0.49 | 0.008** ^#^ | 3.15 | 0.62 | 0.005** ^#^ |
| F: RDC5 0.6 mg/kg po + Naltrexone 1 mg/kg ip | 2.57 | 0.45 | 0.011* | 2.02 | 0.34 | 0.003** | 4.43 | 0.55 | 0.018* | 3.30 | 0.47 | 0.008** |
| G: Bupropion 20 mg/kg ip qd 1-25/bid 26-43 + Naltrexone 1 mg/kg ip | 3.42 | 0.50 | 0.253 | 2.96 | 0.56 | 0.138 | 5.87 | 0.77 | 0.455 | 4.77 | 0.83 | 0.323 |
| H: Sibutramine 5 mg/kg po | 2.39 | 0.32 | 0.004** | 2.01 | 0.37 | 0.002** | 3.97 | 0.46 | 0.003** | 3.10 | 0.48 | 0.004** |

Animals were fasted for 16 h prior to an OGTT performed on Day 37. Samples were analysed for plasma insulin and area under the curve (AUC) and area above the Baseline 2 (AUCB2) calculated for 0-60 and 0-120 min. Analysis was by robust regression including treatment and cohort as factors and Day 1 body weight and bleeding order as covariates. A log transformation was used except for the AUCB2 calculations. Data are shown as adjusted means (n=10-12) and SEM were calculated from the residuals of the statistical models. Comparisons to vehicle were by the multiple t test and significances are denoted by *p<0.05, **p<0.01 and ***p<0.001. The p values shown above are from the comparison to vehicle. Comparisons of combination groups to single treatments were also by the multiple t test and significances against bupropion alone are denoted by #p<0.05 and ##p<0.01, signficances against naltrexone alone are denoted by †p<0.05 and there were no significant differences against RDC5 alone.

#### Table 10 Terminal plasma glucose and insulin levels and comparisons of combination treatments to their individual constituents

**Terminal plasma glucose and insulin levels**

| Treatment | n | Mean | SEM | % of vehicle | p |
| --- | --- | --- | --- | --- | --- |
| Glucose (mM) |  |  |  |  |  |
| Vehicle (4 ml/kg po +2 ml/kg ip) | 12 | 13.75 | 0.39 |  |  |
| RDC5 0.6 mg/kg po | 10 | 14.45 | 0.69 | 105.1 | 0.330 |
| Bupropion 20 mg/kg ip qd/bid | 10 | 13.25 | 0.50 | 96.4 | 0.469 |
| Naltrexone 1 mg/kg ip | 10 | 13.43 | 0.50 | 97.7 | 0.652 |
| RDC5 0.6 mg/kg po+Bupropion 20 mg/kg ip | 10 | 14.22 | 0.42 | 103.4 | 0.509 |
| RDC5 0.6 mg/kg po+Naltrexone 1 mg/kg ip | 10 | 14.00 | 0.58 | 101.8 | 0.720 |
| Bupropion 20 mg/kg ip+Naltrexone 1 mg/kg ip | 10 | 14.82 | 0.55 | 107.8 | 0.142 |
| Sibutramine 5 mg/kg po | 10 | 12.76 | 0.38 | 92.8 | 0.147 |
| Insulin (ng/ml) |  |  |  |  |  |
| Vehicle (4 ml/kg po +2 ml/kg ip) | 12 | 6.31 | 0.73 |  |  |
| RDC5 0.6 mg/kg po | 10 | 4.14 | 0.62 | 65.6 | 0.046* |
| Bupropion 20 mg/kg ip qd/bid | 10 | 5.16 | 0.57 | 81.8 | 0.337 |
| Naltrexone 1 mg/kg ip | 10 | 3.49 | 0.81 | 55.3 | 0.006** |
| RDC5 0.6 mg/kg po+Bupropion 20 mg/kg ip | 10 | 4.90 | 0.71 | 77.6 | 0.227 |
| RDC5 0.6 mg/kg po+Naltrexone 1 mg/kg ip | 10 | 3.84 | 0.46 | 60.8 | 0.019* |
| Bupropion 20 mg/kg ip+Naltrexone 1 mg/kg ip | 10 | 3.60 | 0.51 | 57.0 | 0.009** |
| Sibutramine 5 mg/kg po | 10 | 5.57 | 0.84 | 88.2 | 0.548 |

Means are back-transformed and adjusted for differences between the treatment groups in body weight at baseline (Day

1) and bleeding order. SEM are calculated from the residuals of the statistical model. Multiple comparisons against vehicle are by the multiple test. *p<0.05, **p<0.01

#### Terminal plasma glucose and insulin levels comparisons of combination treatments to their individual constituents

| Comparison | % | p |
| --- | --- | --- |
| Glucose (mM) |  |  |
| RDC5 (0.6 mg/kg po)+Bupropion (20 mg/kg ip qd/bid) vs RDC5 | 98.4 | 0.763 |
| RDC5 (0.6 mg/kg po)+Bupropion (20 mg/kg ip qd/bid) vs Bupropion | 107.3 | 0.187 |
| RDC5 (0.6 mg/kg po)+Naltrexone (1 mg/kg ip) vs RDC5 | 96.9 | 0.554 |
| RDC5 (0.6 mg/kg po)+Naltrexone (1 mg/kg ip) vs Naltrexone | 104.2 | 0.440 |
| Bupropion (20 mg/kg ip qd/bid)+Naltrexone (1 mg/kg ip) vs Bupropion | 111.9 | 0.038* |
| Bupropion (20 mg/kg ip qd/bid)+Naltrexone (1 mg/kg ip) vs Naltrexone | 110.3 | 0.068 |
| Insulin (ng/ml) |  |  |
| RDC5 (0.6 mg/kg po)+Bupropion (20 mg/kg ip qd/bid) vs RDC5 | 118.3 | 0.441 |
| RDC5 (0.6 mg/kg po)+Bupropion (20 mg/kg ip qd/bid) vs Bupropion | 94.9 | 0.809 |
| RDC5 (0.6 mg/kg po)+Naltrexone (1 mg/kg ip) vs RDC5 | 92.7 | 0.729 |
| RDC5 (0.6 mg/kg po)+Naltrexone (1 mg/kg ip) vs Naltrexone | 109.9 | 0.665 |
| Bupropion (20 mg/kg ip qd/bid)+Naltrexone (1 mg/kg ip) vs Bupropion | 69.7 | 0.100 |
| Bupropion (20 mg/kg ip qd/bid)+Naltrexone (1 mg/kg ip) vs Naltrexone | 103.0 | 0.893 |
| Multiple comparisons are by the multiple test. *p<0.05 |  |  |

**Table 11 Pancreatic insulin levels and comparisons of combination treatments to their individual constituents**

**Pancreatic insulin levels (μg insulin/g tissue)**

| Treatment | n | Mean | SEM | % of vehicle | p |
| --- | --- | --- | --- | --- | --- |
| Vehicle (4 ml/kg po +2 ml/kg ip) | 6 | 39.8 | 4.7 |  |  |
| RDC5 0.6 mg/kg po | 5 | 22.0 | 2.5 | 55.2 | 0.006** |
| Bupropion 20 mg/kg ip qd/bid | 5 | 34.1 | 6.7 | 85.7 | 0.448 |
| Naltrexone 1 mg/kg ip | 5 | 47.8 | 5.4 | 120.1 | 0.369 |
| RDC5 0.6 mg/kg po+Bupropion 20 mg/kg ip | 5 | 21.4 | 3.9 | 53.8 | 0.005** |
| RDC5 0.6 mg/kg po+Naltrexone 1 mg/kg ip | 5 | 17.8 | 2.9 | 44.6 | <0.001*** |
| Bupropion 20 mg/kg ip+Naltrexone 1 mg/kg ip | 5 | 47.2 | 4.3 | 118.5 | 0.405 |

Means are back-transformed. SEM are calculated from the residuals of the statistical model. Multiple comparisons against vehicle are by the multiple test. **p<0.01, ***p<0.001

#### Pancreatic insulin levels (μg insulin/g tissue) comparisons of combination treatments to their individual constituents

| Comparison | % | p |
| --- | --- | --- |
| RDC5 (0.6 mg/kg po)+Bupropion (20 mg/kg ip qd/bid) vs RDC5 | 97.4 | 0.902 |
| RDC5 (0.6 mg/kg po)+Bupropion (20 mg/kg ip qd/bid) vs Bupropion | 62.8 | 0.034* |
| RDC5 (0.6 mg/kg po)+Naltrexone (1 mg/kg ip) vs RDC5 | 80.8 | 0.321 |
| RDC5 (0.6 mg/kg po)+Naltrexone (1 mg/kg ip) vs Naltrexone | 37.1 | <0.001*** |
| Bupropion (20 mg/kg ip qd/bid)+Naltrexone (1 mg/kg ip) vs Bupropion | 138.3 | 0.135 |
| Bupropion (20 mg/kg ip qd/bid)+Naltrexone (1 mg/kg ip) vs Naltrexone | 98.7 | 0.950 |
| Multiple comparisons are by the multiple test. *p<0.05, ***p<0.001 |  |  |

| Treatment | n | Mean | SEM | Difference | % change | p |
| --- | --- | --- | --- | --- | --- | --- |
| Retroperitoneal fat pads |  |  |  |  |  |  |
| Vehicle 4 ml/kg po +2 ml/kg ip | 12 | 11.5 | 0.8 |  |  |  |
| RDC5 0.6 mg/kg po | 10 | 9.9 | 0.5 | -1.6 | -13.8 | 0.218 |
| Bupropion 20 mg/kg ip qd/bid | 10 | 11.2 | 0.7 | -0.3 | -2.9 | 0.797 |
| Naltrexone 1 mg/kg ip | 10 | 13.7 | 1.3 | 2.2 | 19.0 | 0.092 |
| RDC5 0.6 mg/kg po+Bupropion 20 mg/kg ip qd/bid | 10 | 9.0 | 0.9 | -2.5 | -22.1 | 0.051 |
| RDC5 0.6 mg/kg po+Naltrexone 1 mg/kg ip | 10 | 11.5 | 1.2 | -0.1 | -0.5 | 0.961 |
| Bupropion 20 mg/kg ip qd/bid+Naltrexone 1 mg/kg ip | 10 | 12.2 | 0.9 | 0.7 | 5.8 | 0.605 |
| Liver |  |  |  |  |  |  |
| Vehicle 4 ml/kg po +2 ml/kg ip | 12 | 12.8 | 0.5 |  |  |  |
| RDC5 0.6 mg/kg po | 10 | 13.2 | 0.3 | 0.4 | 3.4 | 0.495 |
| Bupropion 20 mg/kg ip qd/bid | 10 | 14.0 | 0.5 | 1.2 | 9.6 | 0.057 |
| Naltrexone 1 mg/kg ip | 10 | 12.6 | 0.6 | -0.2 | -1.5 | 0.761 |
| RDC5 0.6 mg/kg po+Bupropion 20 mg/kg ip qd/bid | 10 | 13.9 | 0.4 | 1.1 | 9.0 | 0.075 |
| RDC5 0.6 mg/kg po+Naltrexone 1 mg/kg ip | 10 | 12.9 | 0.4 | 0.2 | 1.3 | 0.794 |
| Bupropion 20 mg/kg ip qd/bid+Naltrexone 1 mg/kg ip | 10 | 13.6 | 0.5 | 0.8 | 6.6 | 0.190 |
| Pancreas |  |  |  |  |  |  |
| Vehicle 4 ml/kg po +2 ml/kg ip | 12 | 0.880 | 0.039 |  |  |  |
| RDC5 0.6 mg/kg po | 10 | 0.955 | 0.070 | 0.074 | 8.4 | 0.295 |
| Bupropion 20 mg/kg ip qd/bid | 10 | 0.884 | 0.049 | 0.004 | 0.4 | 0.958 |
| Naltrexone 1 mg/kg ip | 10 | 0.973 | 0.052 | 0.093 | 10.5 | 0.192 |
| RDC5 0.6 mg/kg po+Bupropion 20 mg/kg ip qd/bid | 10 | 0.925 | 0.064 | 0.044 | 5.0 | 0.530 |
| RDC5 0.6 mg/kg po+Naltrexone 1 mg/kg ip | 10 | 0.916 | 0.036 | 0.035 | 4.0 | 0.617 |
| Bupropion 20 mg/kg ip qd/bid+Naltrexone 1 mg/kg ip | 10 | 0.911 | 0.036 | 0.031 | 3.5 | 0.666 |

Means are adjusted for differences between the treatment groups in Day 1 body weight. SEM are calculated from the residuals of the statistical model. Multiple comparisons against vehicle are by the multiple t test.

#### Tissue weights (g) comparisons of combination treatments to their individual constituents

| Comparison | Difference | p |
| --- | --- | --- |
| Retroperitoneal fat pads |  |  |
| RDC5 (0.6 mg/kg po)+Bupropion (20 mg/kg ip qd/bid) vs RDC5 | -1.0 | 0.478 |
| RDC5 (0.6 mg/kg po)+Naltrexone (1 mg/kg ip) vs RDC5 | 1.5 | 0.257 |
| RDC5 (0.6 mg/kg po)+Bupropion (20 mg/kg ip qd/bid) vs Bupropion | -2.2 | 0.103 |
| Bupropion (20 mg/kg ip qd/bid)+Naltrexone (1 mg/kg ip) vs Bupropion | 1.0 | 0.459 |
| RDC5 (0.6 mg/kg po)+Naltrexone (1 mg/kg ip) vs Naltrexone | -2.3 | 0.097 |
| Bupropion (20 mg/kg ip qd/bid)+Naltrexone (1 mg/kg ip) vs Naltrexone | -1.5 | 0.259 |
| Liver |  |  |
| RDC5 (0.6 mg/kg po)+Bupropion (20 mg/kg ip qd/bid) vs RDC5 | 0.7 | 0.286 |
| RDC5 (0.6 mg/kg po)+Naltrexone (1 mg/kg ip) vs RDC5 | -0.3 | 0.686 |
| RDC5 (0.6 mg/kg po)+Bupropion (20 mg/kg ip qd/bid) vs Bupropion | -0.1 | 0.903 |
| Bupropion (20 mg/kg ip qd/bid)+Naltrexone (1 mg/kg ip) vs Bupropion | -0.4 | 0.561 |
| RDC5 (0.6 mg/kg po)+Naltrexone (1 mg/kg ip) vs Naltrexone | 0.4 | 0.590 |
| Bupropion (20 mg/kg ip qd/bid)+Naltrexone (1 mg/kg ip) vs Naltrexone | 1.0 | 0.124 |
| Pancreas |  |  |
| RDC5 (0.6 mg/kg po)+Bupropion (20 mg/kg ip qd/bid) vs RDC5 | -0.030 | 0.686 |
| RDC5 (0.6 mg/kg po)+Naltrexone (1 mg/kg ip) vs RDC5 | -0.039 | 0.598 |
| RDC5 (0.6 mg/kg po)+Bupropion (20 mg/kg ip qd/bid) vs Bupropion | 0.041 | 0.582 |
| Bupropion (20 mg/kg ip qd/bid)+Naltrexone (1 mg/kg ip) vs Bupropion | 0.027 | 0.717 |
| RDC5 (0.6 mg/kg po)+Naltrexone (1 mg/kg ip) vs Naltrexone | -0.057 | 0.438 |
| Bupropion (20 mg/kg ip qd/bid)+Naltrexone (1 mg/kg ip) vs Naltrexone | -0.062 | 0.401 |
| Multiple comparisons are by the multiple t test. |  |  |

| Treatment | n | Mean | SEM | Difference | % change | p |
| --- | --- | --- | --- | --- | --- | --- |
| Retroperitoneal fat pads |  |  |  |  |  |  |
| Vehicle 4 ml/kg po +2 ml/kg ip | 12 | 10.9 | 0.7 |  |  |  |
| RDC5 0.6 mg/kg po | 10 | 10.5 | 0.4 | -0.4 | -3.3 | 0.746 |
| Bupropion 20 mg/kg ip qd/bid | 10 | 10.8 | 0.4 | -0.0 | -0.4 | 0.973 |
| Naltrexone 1 mg/kg ip | 10 | 12.9 | 1.2 | 2.1 | 19.2 | 0.064 |
| RDC5 0.6 mg/kg po+Bupropion 20 mg/kg ip qd/bid | 10 | 10.1 | 0.8 | -0.8 | -7.2 | 0.487 |
| RDC5 0.6 mg/kg po+Naltrexone 1 mg/kg ip | 10 | 11.6 | 1.0 | 0.7 | 6.5 | 0.529 |
| Bupropion 20 mg/kg ip qd/bid+Naltrexone 1 mg/kg ip | 10 | 12.5 | 0.7 | 1.6 | 15.1 | 0.145 |
| Liver |  |  |  |  |  |  |
| Vehicle 4 ml/kg po +2 ml/kg ip | 12 | 12.4 | 0.5 |  |  |  |
| RDC5 0.6 mg/kg po | 10 | 13.5 | 0.2 | 1.0 | 8.3 | 0.092 |
| Bupropion 20 mg/kg ip qd/bid | 10 | 13.8 | 0.4 | 1.4 | 10.9 | 0.026* |
| Naltrexone 1 mg/kg ip | 10 | 12.2 | 0.6 | -0.3 | -2.1 | 0.665 |
| RDC5 0.6 mg/kg po+Bupropion 20 mg/kg ip qd/bid | 10 | 14.4 | 0.3 | 2.0 | 16.1 | 0.002** |
| RDC5 0.6 mg/kg po+Naltrexone 1 mg/kg ip | 10 | 13.0 | 0.3 | 0.5 | 4.3 | 0.372 |
| Bupropion 20 mg/kg ip qd/bid+Naltrexone 1 mg/kg ip | 10 | 13.8 | 0.6 | 1.3 | 10.5 | 0.032* |
| Pancreas |  |  |  |  |  |  |
| Vehicle 4 ml/kg po +2 ml/kg ip | 12 | 0.883 | 0.039 |  |  |  |
| RDC5 0.6 mg/kg po | 10 | 0.953 | 0.069 | 0.070 | 7.9 | 0.329 |
| Bupropion 20 mg/kg ip qd/bid | 10 | 0.886 | 0.049 | 0.003 | 0.3 | 0.969 |
| Naltrexone 1 mg/kg ip | 10 | 0.976 | 0.051 | 0.093 | 10.6 | 0.190 |
| RDC5 0.6 mg/kg po+Bupropion 20 mg/kg ip qd/bid | 10 | 0.921 | 0.064 | 0.038 | 4.3 | 0.598 |
| RDC5 0.6 mg/kg po+Naltrexone 1 mg/kg ip | 10 | 0.915 | 0.037 | 0.033 | 3.7 | 0.646 |
| Bupropion 20 mg/kg ip qd/bid+Naltrexone 1 mg/kg ip | 10 | 0.910 | 0.037 | 0.027 | 3.0 | 0.704 |

Means are adjusted for differences between the treatment groups in final carcass weight. SEM are calculated from the residuals of the statistical model. Multiple comparisons against vehicle are by the multiple t test. *p<0.05, **p<0.01

#### Tissue weights (g) comparisons of combination treatments to their individual constituents

| Comparison | Difference | p |
| --- | --- | --- |
| Retroperitoneal fat pads |  |  |
| RDC5 (0.6 mg/kg po)+Bupropion (20 mg/kg ip qd/bid) vs RDC5 | -0.4 | 0.716 |
| RDC5 (0.6 mg/kg po)+Naltrexone (1 mg/kg ip) vs RDC5 | 1.1 | 0.360 |
| RDC5 (0.6 mg/kg po)+Bupropion (20 mg/kg ip qd/bid) vs Bupropion | -0.7 | 0.523 |
| Bupropion (20 mg/kg ip qd/bid)+Naltrexone (1 mg/kg ip) vs Bupropion | 1.7 | 0.151 |
| RDC5 (0.6 mg/kg po)+Naltrexone (1 mg/kg ip) vs Naltrexone | -1.4 | 0.237 |
| Bupropion (20 mg/kg ip qd/bid)+Naltrexone (1 mg/kg ip) vs Naltrexone | -0.4 | 0.703 |
| Liver |  |  |
| RDC5 (0.6 mg/kg po)+Bupropion (20 mg/kg ip qd/bid) vs RDC5 | 1.0 | 0.123 |
| RDC5 (0.6 mg/kg po)+Naltrexone (1 mg/kg ip) vs RDC5 | -0.5 | 0.433 |
| RDC5 (0.6 mg/kg po)+Bupropion (20 mg/kg ip qd/bid) vs Bupropion | 0.6 | 0.311 |
| Bupropion (20 mg/kg ip qd/bid)+Naltrexone (1 mg/kg ip) vs Bupropion | -0.0 | 0.939 |
| RDC5 (0.6 mg/kg po)+Naltrexone (1 mg/kg ip) vs Naltrexone | 0.8 | 0.206 |
| Bupropion (20 mg/kg ip qd/bid)+Naltrexone (1 mg/kg ip) vs Naltrexone | 1.6 | 0.014* |
| Pancreas |  |  |
| RDC5 (0.6 mg/kg po)+Bupropion (20 mg/kg ip qd/bid) vs RDC5 | -0.032 | 0.666 |
| RDC5 (0.6 mg/kg po)+Naltrexone (1 mg/kg ip) vs RDC5 | -0.037 | 0.614 |
| RDC5 (0.6 mg/kg po)+Bupropion (20 mg/kg ip qd/bid) vs Bupropion | 0.035 | 0.637 |
| Bupropion (20 mg/kg ip qd/bid)+Naltrexone (1 mg/kg ip) vs Bupropion | 0.024 | 0.744 |
| RDC5 (0.6 mg/kg po)+Naltrexone (1 mg/kg ip) vs Naltrexone | -0.061 | 0.413 |
| Bupropion (20 mg/kg ip qd/bid)+Naltrexone (1 mg/kg ip) vs Naltrexone | -0.066 | 0.372 |
| Multiple comparisons are by the multiple t test. *p<0.05 |  |  |

**Figure 1 Effects of RDC5, bupropion and naltrexone, alone and in combination, on body weight in dietary-induced obese, female Wistar rats**

460


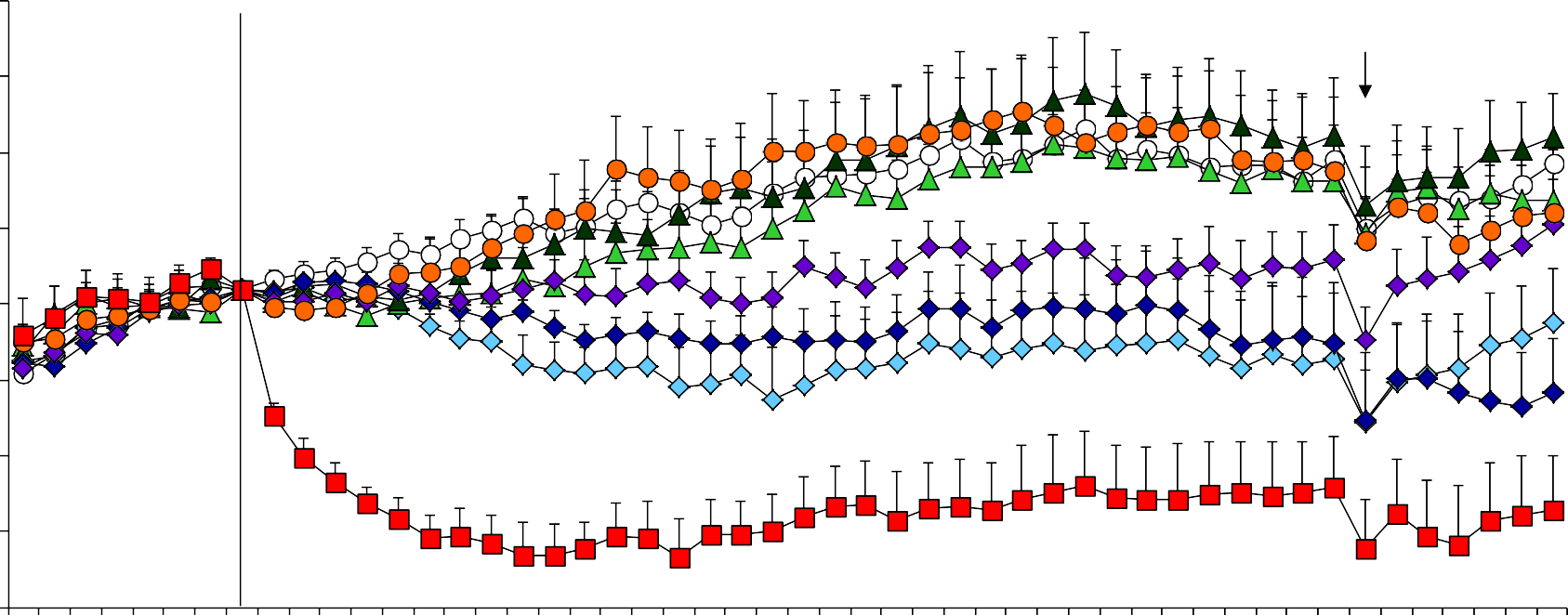


Baseline

OGTT

**Ψ**

**† †**

**†**

**†**

**† †**

**†**

**†**

*** * ***

*****

***** *** * ***

*** ** *** ****

*** ****

*****

*****

******

*****

******

**** ** * *** ** ** *** *** ** *** *** *** * ***

***#***

**# *#**

**#**

**# # #**

**#**

**#**

**#**

******

*** * ***

*****

**# ****

*** ***

*******

******

******

**†**

*******

*** ****

**#**

*****

**# #**

**#**

**#**

**#**

***#**

*******

******

***# *** * * *****

**#**

**# *#*# #*#* ##**

*******

**********
[truncated: 27,328 more chars]
